# Epigenetic Clock in Bears: A Simple Cost‐Effective Blood DNA Methylation‐Based Age Estimation Method Applicable to Multiple Bear Species

**DOI:** 10.1002/ece3.71424

**Published:** 2025-05-06

**Authors:** Michito Shimozuru, Shiori Nakamura, Jumpei Yamazaki, Yojiro Yanagawa, Hiroo Tamatani, Misako Kuroe, Koji Yamazaki, Shinsuke Koike, Yusuke Goto, Tomoko Naganuma, Kahoko Tochigi, Akino Inagaki, Naoki Takekoshi, Seungyun Baek, Nobutaka Sato, Yusuke Honda, Toshio Tsubota, Hideyuki Ito

**Affiliations:** ^1^ Faculty of Veterinary Medicine Hokkaido University Sapporo Hokkaido Japan; ^2^ One Health Research Center Hokkaido University Sapporo Hokkaido Japan; ^3^ Department of Bear Management Picchio Wildlife Research Center Nagano Japan; ^4^ Nagano Environmental Conservation Research Institute Nagano Japan; ^5^ Tokyo University of Agriculture Tokyo Japan; ^6^ Zoological Laboratory Ibaraki Nature Museum Ibaraki Japan; ^7^ Tokyo University of Agriculture and Technology Tokyo Japan; ^8^ Obihiro University of Agriculture and Veterinary Medicine Obihiro Hokkaido Japan; ^9^ Asahikawa City Asahiyama Zoo Asahikawa Hokkaido Japan; ^10^ Noichi Zoological Park of Kochi Prefecture Konan Kochi Japan; ^11^ Wildlife Research Center Kyoto University Kyoto Japan; ^12^ Kyoto City Zoo Kyoto Japan

**Keywords:** age estimation, aging, bear, carnivore, DNA methylation, epigenetic clock

## Abstract

Age is an essential factor to understand the life history and demographic parameters of wildlife. Previously, we established an age estimation method for brown bears based on blood DNA methylation level. In this study, we first applied the brown bear‐specific age estimation model to other bear species, including Asian black, polar, sun, and Andean bears. Using blood DNA, we performed bisulfite pyrosequencing to determine the methylation levels at four cytosine‐phosphate‐guanine (CpG) sites adjacent to a single gene, *SLC12A5*. The best model specific to brown bears estimated their ages with satisfactory accuracy, with mean absolute error (MAE) of 1.5, 2.1, 2.2, and 0.4 years for Asian black (52 samples from 16 captive and 36 wild bears), polar (27 samples from 21 captive bears), sun bears (11 samples from 8 captive bears), and Andean bears (one captive bear), respectively. Then, we established an Asian black bear‐specific age estimation model and a common age estimation model applicable for other bear species (i.e., a pan‐bear model) using the methylation levels of the four CpG sites. The best model specific to Asian black bears had high accuracy with MAE of 1.1 after leave‐one‐out cross‐validation (LOOCV). In addition, the best pan‐bear model achieved accuracy with MAE of 1.3, 1.2, 2.1, and 2.2 years after LOOCV for brown, Asian black, polar, and sun bears, respectively. The results suggested that the pan‐bear age estimation model using the aging marker (CpG sites adjacent to *SLC12A5*) is a simple, highly accurate, and cost‐effective tool that is applicable to Ursidae.

## Graphical abstract

Age is an essential factor to understand the life history and demographic parameters of wildlife. In this study, we built a common epigenetic clock model for multiple bear species, including brown, Asian black, polar, sun, and Andean bears. The model will contribute to ecological research, conservation, and management of bear species.

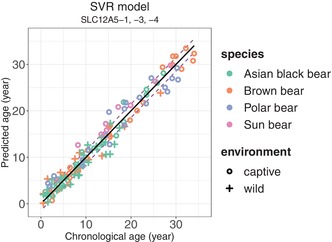

## 1 Introduction

Age information is very important to understand the life history and demographic parameters of wild animals, including age of sexual maturity, reproductive rate, reproductive senescence, age‐dependent survival rate, and lifespan, all of which are necessary for planning wildlife conservation and management. However, it is difficult to obtain age information for wildlife. Long‐term studies with continuous animal tracking have provided important scientific data to clarify the life histories of various species (Sæther et al. 1998; Schwartz et al. 2006). However, these approaches are time‐consuming and costly, especially for long‐lived wildlife species. As an alternative, age estimation methods have been established that are specific for different species (Zhang et al. 2024). Methods involving counting the cementum annuli of the teeth have been used in many species, including marine (Laws 1952; Stewart et al. 1996) and terrestrial mammals (Asmus and Weckerly 2011; Thomas 1977). However, this method has several disadvantages, including invasiveness for captured live animals, the requirement for experienced observers (McLaughlin et al. 1990), and limited accuracy, especially in older individuals (Costello et al. 2004).

Over the past two decades, molecular markers for aging have been established as alternative, less‐invasive means of age estimation (Bocklandt et al. 2011; Haussmann and Mauck 2008). Among them, DNA methylation, an epigenetic mark involving the formation of 5‐methylcytosine via the transfer of a methyl group usually onto the C5 position of cytosine at cytosine‐phosphate‐guanine dinucleotide (CpG) sites, has been used as a marker of aging. DNA methylation level was reported to be correlated with age and was initially applied to age estimation in humans, mainly in forensic research (Horvath 2013), using various biological samples, including blood and muscle tissue. This technique, known as the epigenetic clock, has now been applied to many other species, including laboratory animals (Lowe et al. 2020; Stubbs et al. 2017), companion animals (Horvath et al. 2022; Qi et al. 2021; Raj et al. 2021; Thompson et al. 2017), and wild animals (Czajka et al. 2024; Le Clercq et al. 2024; Lemaitre et al. 2022; Polanowski et al. 2014; Thompson et al. 2017). For epigenetic clock development, some studies applied the mammalian methylation microarray (HorvathMammalMethylChip40; Arneson et al. 2022), which has the potential to screen methylation changes in more than 37,000 highly conserved CpG sites with high coverage. This DNA methylation array is highly innovative and technically excellent in that it provides highly accurate values and is applicable to various animal species and multiple DNA sources, including blood, skin biopsy samples, liver, etc. For example, Lu et al. (2023) developed a high‐accuracy epigenetic clock applicable to 59 tissue types across 185 mammalian species (i.e., universal pan‐mammalian clocks) using the HorvathMammalMethylChip40 microarray. While chip‐based methods offer easy data processing through available R packages and include epigenetic clock calibration in the fee, their significantly higher costs remain a limitation. A more convenient and cost‐effective method is required for studies with limited budgets and/or with large numbers of samples. Recently, Qi et al. (2024) successfully developed an epigenetic clock in domestic cats by targeting 23 selected CpG sites from five gene regions using various machine learning techniques. After generating barcoded DNA sequencing libraries for each gene amplicon from 334 bisulfited DNA samples, they pooled them to make a single library. Then they conducted next‐generation sequencing to reduce the cost of bisulfite sequencing. The model had MAE of 2.0 years, which was slightly inferior to the value (0.79 years) in the above study using the HorvathMammalMethylChip40 (Raj et al. 2021) but was less than 10% of the maximum lifespan (20–30 years old, O'neill et al. 2014; Human Ageing Genomic Resources 2023), so sufficiently accurate. Furthermore, they established age estimation models with the same protocol for other felid species—the Tsushima leopard cat ( *Prionailurus bengalensis euptilurus* ) and *Panthera* spp.—with satisfactory accuracy (the MAE values were 1.5 and 1.6 years, respectively). This suggested that it is possible to establish cost‐effective and readily applicable age estimation models for research, conservation, and management purposes for related animal species.

Recently, we established a novel age estimation method for brown bears ( *Ursus arctos* ) both living in captivity and in the wild based on DNA methylation levels using blood DNA (Nakamura et al. 2023). We applied bisulfite pyrosequencing to obtain methylation levels at 39 CpG sites adjacent to 12 genes, of which 13 CpGs adjacent to four genes showed significant correlations with age. The best model was based on just four CpG sites adjacent to a single gene, solute carrier family 12 member 5 (*SLC12A5*), with MAE of 1.3 years, which was highly accurate as compared to the lifespan of the animals (20–30 years; Bunnell and Trait 1981). The methylation levels of CpG sites adjacent to *SLC12A5* have been reported to be correlated with age in other carnivores (Horvath et al. 2022; Ito et al. 2017; Raj et al. 2021; Robeck et al. 2023), suggesting that this estimation model may be applicable to other bear species. With the exception of brown bears and American black bears ( *Ursus americanus* ), most bear species have received conservation attention, mainly due to habitat loss and human–bear conflicts (Can et al. 2014), and are listed as vulnerable species in the International Union for Conservation of Nature (IUCN) Red List (https://www.iucnredlist.org/). If this method was applied to other bear species, it would contribute to conservation and management as well as ecological research of bear species for which it is difficult to obtain adequate samples. This study was performed to examine whether epigenetic markers and the model established for brown bears could be applied to other bear species, including Asian black bears ( *Ursus thibetanus* ), polar bears ( *Ursus maritimus* ), sun bears ( *Helarctos malayanus* ), and Andean bears ( *Tremarctos ornatus* ). In addition, we attempted to establish an age estimation model commonly applicable to multiple bear species (i.e., a pan‐bear model), as well as a model specific to the Asian black bear for which we were able to obtain a sufficient number of samples.

## 2 Materials and Methods

### 2.1 Ethics Statement

All procedures involved in sample collection from captive bears were conducted in accordance with the Guidelines for Animal Care and Use, Hokkaido University, and were approved by the Animal Care and Use Committee of the Graduate School of Veterinary Medicine, Hokkaido University (Approval Number: JU8114, JU8134, JU9124, 17,006, 18–0179, 20–0146, and 23–0144). All procedures involved in sample collection from wild bears in the Ashio area in Tochigi Prefecture were conducted with the Guidelines Concerning Animal Experimentation of the Tokyo University of Agriculture and the Mammal Society of Japan (https://www.mammalogy.jp/en/guideline.pdf) and were approved by the Animal Experiment Committee of Tokyo University of Agriculture (Approval Number: #156206). All procedures involved in sample collection from wild bears in the Karuizawa area in Nagano Prefecture were conducted with the Guidelines Concerning Animal Experimentation of the Mammal Society of Japan and were approved by the Ministry of the Environment and Nagano Prefecture.

### 2.2 Study Area, Animals, and Blood Collection

#### 2.2.1 Captive Bears

Samples and detailed information on blood sampling (bear ID, species, sex, area, age, date of blood collection, etc.) are shown in Table 1 and Table S_M1, respectively. Dates of birth were available for all captive bears except two sun bears, which were born in 1994 in China and transported to Japan in 1995. As birth occurs year‐round in the sun bear (Frederick et al. 2012), we assumed that both were born in the middle of the year (i.e., July 2). Blood samples were obtained from 16 Asian black bears (14 Japanese black bears, *Ursus thibetanus japonicus* , 4 males and 10 females; 2 Himalayan black bears, *Ursus thibetanus laniger* , one male and one female), 21 polar bears (27 samples in total from 7 males and 14 females), eight sun bears (11 samples in total from 4 males and 4 females), and one male Andean bear. Blood was collected from the jugular or medial saphenous vein under anesthesia or via the dorsal venous network of the hand or the cephalic vein in the carpal joint without anesthesia during husbandry procedures (Otaki et al. 2015), except for the Andean bear from which blood was collected during necropsy after death. Bears were immobilized using blow darts with intramuscular administration of medetomidine HCl (40 μg/kg; Dorbene; Kyoritsu Seiyaku, Tokyo, Japan) and a 1:1 mixture of zolazepam HCl and tiletamine HCl (2.0–4.0 mg/kg; Zoletil 100; Virbac, Carros, France). After blood sampling was completed, atipamezole HCl (200 μg; Atipame; Kyoritsu Seiyaku, Tokyo, Japan) was injected intramuscularly to aid recovery from anesthesia. Blood samples were collected into vacuum tubes containing ethylenediaminetetraacetic acid disodium (EDTA‐2Na) as an anticoagulant. The collected blood samples were stored at −80°C as whole blood or buffy coat until genomic DNA extraction. Buffy coat samples were obtained by centrifuging blood samples at 1880 × g for 10 min.

**TABLE 1:** Samples from four bear species used in this study.

Species	Environment	Area	No. samples	No. bears	No. females	Min. age	Max. age	Ave. age
Asian black bears	Captive	—	16	16^a^	10	2.6	28.1	13.2
	Wild	Tochigi	20	20	11	1.4	17.5	8.6
	Wild	Nagano	16	16	9	0.6	16.4	7.2
Polar bears	Captive	—	27	21	14	1.6	30.5	16.1
Sun bears	Captive	—	11	8	4	8.2	28.8	19.2
Andean bears	Captive	—	1	1	0	33.0^b^	—^b^	—^b^

^a^ Fourteen Japanese black bears and two Himalayan black bears are included.

^b^ Only one Andean bear was included.

#### 2.2.2 Wild Bears

We collected blood samples from 36 wild Japanese black bears captured from a wide area around the Ashio area in Tochigi Prefecture (9 males and 11 females) and Karuizawa area in Nagano Prefecture (7 males and 9 females) (Table 1 and Table S_M1). In both areas, long‐term bear surveys, including mark and release studies, have been conducted from 2003 to the present. We used blood samples collected between 2015 and 2021 from bears with ages estimated by counting tooth cementum annuli (Tochigi et al. 2018) at the first capture between 2006 and 2020, except for one cub‐of‐the‐year the age of which was identified based on body size. In general, cementum annuli become more difficult to read in older individuals (Costello et al. 2004). Czajka et al. (2024) successfully established an epigenetic clock in American black bears based on bears that were estimated to be between 1 and 19 years old according to examinations of their teeth. However, the accuracy of such age assessment has never been verified scientifically for Asian black bears. Therefore, in this study, we limited bears to those with an estimated age of less than 6 years at first capture. Pregnant female Japanese black bears give birth from late January to early February during winter hibernation (Iibuchi et al. 2009), so we assumed that all bears were born on February 1. Finally, the age at the time of blood sampling (ranging from 0.6 to 17.4 years; Table S_M1) was determined based on tooth examinations on the date of first capture and the date of recapture when blood was sampled. Bears were captured in barrel traps using honey and honeycombs as attractants. The protocols for anesthesia, blood collection, and storage were the same as for captive bears.

### 2.3 DNA Extraction and Bisulfite Conversion

Genomic DNA was extracted using the DNeasy Blood & Tissue Kit (Qiagen, Venlo, Netherlands), according to the manufacturer's protocol. The concentration of extracted DNA was measured using the NanoDrop 2000c spectrophotometer (Thermo Fisher Scientific, Waltham, MA, USA), and extracted genomic DNA was stored at −30°C until bisulfite conversion using the EZ DNA Methylation‐Gold Kit (Zymo Research, Irvine, CA, USA) according to the manufacturer's instructions.

### 2.4 Target Genes, Polymerase Chain Reaction, and Pyrosequencing

We targeted genomic locations adjacent to a single gene, solute carrier family 12 member 5 (*SLC12A5*), which includes four target CpG sites (i.e., SL‐1, ‐2, ‐3, and ‐4). In our previous study (Nakamura et al. 2023), we generated three age estimation models, including a single regression model and two multiple regression models (elastic net regression and support vector regression [SVR]) using 13 CpG sites adjacent to four genes. Among them, SVR using the methylation levels of all four CpGs adjacent to *SLC12A5* showed the best performance (MAE = 1.304), whereas the single regression model and elastic net regression model, using the methylation levels of one CpG (SL‐4) or all four CpGs (SL‐1, ‐2, ‐3, and ‐4), respectively, showed comparable performance in age estimation of brown bears (the MAE values were 1.582 and 1.593 years, respectively). We identified homologous sequences in the genomic regions of the four bear species containing the target CpG sites using the Basic Local Alignment Search Tool (BLAST) provided by the National Center for Biotechnology Information (NCBI) (Table 2).

**TABLE 2:** Genomic locations of the target CpG sites in each bear species.

Bear species	GenBank accession ID	DNA positions of the target CpG sites			
		SL‐1	SL‐2	SL‐3	SL‐4
Brown bears	NW_020626136.1	29,314,689	29,314,692	29,314,694	29,314,698
Asian black bears	BMBK01035068.1	182,094	182,097	182,099	182,103
	WEIE01000139.1	1447098^a^	1447095^a^	1447093^a^	1447089^a^
Polar bear	NW_024424675.1	33,687,964	33,687,967	33,687,969	33,687,973
Sun bears	JAPYYF010000022.1	30,912,650	30,912,653	30,912,655	30,912,359
Andean bears	WMLG01006021.1	837796^a^	837793^a^	837791^a^	837787^a^
	JAPYXY010000006.1	13993862^a^	13993859^a^	13993857^a^	13993853^a^

^a^ Denotes the reverse complement.

Polymerase chain reaction (PCR) and subsequent pyrosequencing were conducted following our previous reports (Nakamura et al. 2023; Yamazaki et al. 2021). PCR was conducted in two steps, using the TaKaRa EpiTaq HS (Takara Bio Inc., Shiga, Japan). The first step was performed in a total volume of 15 μL containing 0.75 μL of the genomic DNA sample (diluted to contain 3.75 ng DNA), 0.075 μL PCR Taq, 1.5 μL 10 × PCR Buffer (Mg^2+^ free), 1.5 μL MgCl_2_, 1.8 μL dNTP mixture, 0.3 μL each of the forward (5’‐GTTTTAGGGTTAGAGGTAGAGYG‐3′) and reverse primers (5’‐CTACCCCCRAACTATAAACAAT‐3′) (10 μmol/L), and 8.775 μL molecular‐grade water (Nippon Gene, Tokyo, Japan). Each sample was run in duplicate. The second PCR step was performed in a total volume of 15.13 μL containing 0.1 μL of the first PCR product, 0.075 μL Taq, 1.5 μL 10 × PCR Buffer (Mg^2+^ free), 1.5 μL MgCl_2_, 1.8 μL dNTP mixture, 0.3 μL of the forward primer (5’‐GTTTTAGGGTTAGAGGTAGAGYG‐3′, 10 μmol/L), 0.06 μL of the reverse primer (5’‐GGGACACCGCTGATCGTTTACTACCCCCRAACTATAAACAAT‐3′, 10 μmol/L), 0.27 μL of the biotin‐modified primers (5’‐GGGACACCGCTGATCGTTTA‐3′, 10 μmol/L), and 9.525 μL molecular‐grade water (Nippon Gene). The PCR conditions were 98°C for 1 min, followed by 35 cycles of 98°C for 10 s, 56°C (the first PCR) or 58°C (the second PCR) for 30 s, and 72°C for 30 s. We electrophoresed 2.5 μL of each PCR product with agarose gel to confirm that the target was amplified.

To determine the methylation levels of the target CpGs, pyrosequencing was performed using PyroMark Q48 (Qiagen) with PyroMark Q48 Advanced Reagents (Qiagen) according to the manufacturer's instructions. The mean methylation levels determined from duplicate reactions were used in the analysis.

### 2.5 Age Estimation Using the Model Established for Brown Bears

We applied three models—a single regression model with SL‐4 and two multiple regression models (i.e., elastic net regression and SVR, both with the four CpGs in *SLC12A5*)—to the dataset obtained from the four bear species. The R scripts for each model used to estimate age are available in the Supporting Information of Nakamura et al. (2023).

### 2.6 Establishment of Age Estimation Model for Asian Black Bears

The establishment of age estimation models for Asian black bears was conducted according to the method described in our previous studies in brown bears (Nakamura et al. 2023). Briefly, we built three models, that is, a single regression model and two multiple regression models (elastic net regression and SVR), based on the methylation levels determined by pyrosequencing. We selected these models for simplicity (i.e., a single regression model), frequency of use, and accuracy. Elastic net models, a type of penalized regression, have frequently been used in age‐estimation models (Horvath 2013; Lu et al. 2023; Raj et al. 2021; Thompson et al. 2017), whereas SVR has been reported to produce high estimation accuracy (Nakamura et al. 2023; Qi et al. 2021; Xu et al. 2015). We used data obtained from adjacent CpGs that tend to show the same methylation trends (Affinito et al. 2020), and the four selected CpGs highly correlated in this study (Figures S_M1 and M2). In order to avoid the effects of this multicollinearity, we used principal components analysis to transform the data obtained from four CpGs to one principal component (i.e., first principal component score), with which we additionally built one more single regression model (i.e., principal component regression). It is important to note that multicollinearity has an effect on the explanatory interpretation but does not necessarily affect results in multiple regression analysis for predictive purposes (Shmueli 2010). In addition, the elastic net model can mitigate the effects of multicollinearity (Chan et al. 2022) and the SVR was shown to sufficiently regulate multicollinearity in some studies (e.g., Farrell et al. 2019). Accordingly, multiple models were used to evaluate estimation accuracy. Age and DNA methylation levels were standardized prior to model construction. Single regression, elastic net regression, and SVR models were generated using the R function *lm* and the R packages glmnet and e1071, respectively. All models were validated by leave‐one‐out cross‐validation (LOOCV).

To evaluate factors capable of affecting the deviation of the age estimation model, we generated linear regressions that included Δage (predicted age − chronological age) and |Δage| (absolute difference between predicted age and chronological age) as dependent variables and three factors (age, sex, and growth environment [i.e., captive or wild condition]) as well as the interactions among each factor pair as explanatory variables.

### 2.7 Establishment of a Pan‐Bear Age Estimation Model

Similar to the Asian black bear‐specific age estimation model, we established a pan‐bear age estimation model based on the methylation levels obtained from brown, Asian black, polar, and sun bears. To establish an age estimation model, we used only one sample per individual animal to avoid overfitting of the model to the methylation changes of individuals that were sampled multiple times. For polar and sun bears sampled multiple times (6 and 3 bears, respectively), samples were selected to include as wide an age range as possible, and some were selected randomly (Table S_M1). Methylation data for brown bears were from our previous study (Nakamura et al. 2023) (available on Dryad at doi:10.5061/dryad.9w0vt4bm0). The data included a total of 49 brown bears, including 34 captive (17 females and 17 males) and 15 wild (14 females and 1 male) bears. The Andean bear (*n* = 1) was not included in the model establishment.

To evaluate factors capable of affecting the deviation of the age estimation model, we generated linear regressions that included Δage and |Δage| as dependent variables and four factors (species [i.e., brown, Asian black, polar, and sun bears], age, sex, and growth environment [i.e., captive or wild condition]) as well as the interactions among each factor pair as explanatory variables.

## 3 Results

### 3.1 Correlation Between DNA Methylation Level and Chronological Age

**FIGURE 1:**
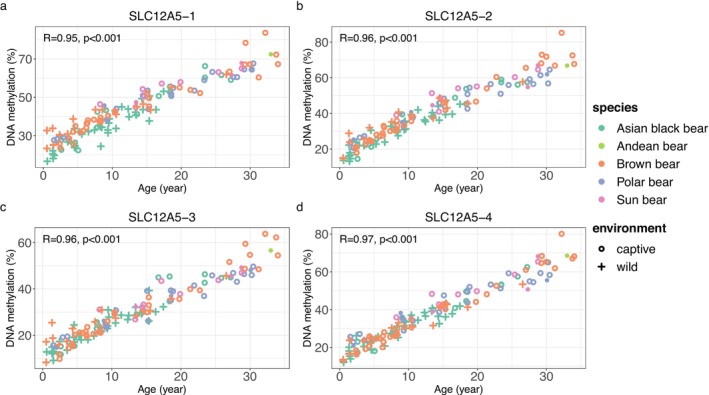
Scatter plots of age versus DNA methylation level (%) of four CpGs (a: SL‐1; b: SL‐2; c: SL‐3; d: SL‐4) in five bear species. Brown bears data were from Nakamura et al. (2023). The closed circles indicate the data which were excluded from pan‐bear age estimation model to avoid duplicate samples of the same individuals.

There were significant correlations between methylation levels of all four target CpGs adjacent to *SLC12A5* and chronological age in all three bear species examined, except for the Andean bear (*n* = 1) (Figure 1). The data, including correlation coefficients and *p* values for each CpG for each bear species, are shown in Table S_R1.

### 3.2 Application of Age Estimation Model Established for Brown Bears

We applied three brown bear‐specific age estimation models, including single regression, elastic net regression, and SVR models, to Asian black, polar, and sun bears. The MAE, median absolute error (MedAE), and root mean square error (RMSE) values are presented in Table 3. The elastic net regression model was selected as the most accurate model for Asian black bears (MAE = 1.197) and sun bears (MAE = 1.921), whereas the SVR model was selected as the most accurate model for polar bears (MAE = 2.143). The one Andean bear (33.0 years old) sample examined had an estimated age of 31.6 years in the single regression model, 30.9 years in the elastic net regression model, and 32.6 years in the SVR model.

**TABLE 3:** Application of brown bear‐specific age estimation model to Asian black, polar, and sun bears. Mean absolute error (MAE), median absolute error (MedAE), and root mean square error (RMSE) values were presented for each model for each species.

Model	Used CpGs	Asian black bears			Polar bears			Sun bears			Brown bears^a^		
		MAE	MedAE	RMSE	MAE	MedAE	RMSE	MAE	MedAE	RMSE	MAE	MedAE	RMSE
Single regression	SL‐4	1.396	1.161	1.720	2.737	2.649	3.181	2.639	2.277	3.327	1.735	1.735	2.178
Elastic net regression	SL‐1, ‐2, ‐3, ‐4	**1.197**	**0.941**	**1.520**	2.459	2.341	2.872	**1.921**	**1.139**	**2.539**	1.630	1.537	**1.988**
Support vector regression	SL‐1, ‐2, ‐3, ‐4	1.533	1.157	1.970	**2.143**	**1.948**	**2.550**	2.172	1.858	2.601	**1.610**	**1.440**	2.029

*Note:* Each bold value denotes the minimum value for each indicator.

^a^ Values of brown bears were generated using wild brown bear samples that were not included in the brown bear‐specific model establishment in Nakamura et al. (2023). Those values were different from the values generated by LOOCV in Nakamura et al. (2023).

### 3.3 Age Estimation Model for Asian Black Bears

We constructed a total of 17 age estimation models (4 for single regression model, 1 for principal component regression, 1 for elastic net regression, and 11 for SVR models). Values of MAE, MedAE, and RMSE for elastic net regression and SVR models are presented in Table 4. Since the SVR model does not reduce features during its construction process, all possible combinations of the four CpGs were examined. Regarding principal component regression, the formula for the first principal component score is as follows:

$$\begin{array}{cc} \text{PC1}=\; & \left(-0.49916\times \text{standardized methylation level of}\;\mathrm{SL}-1\right)\\ & +\left(-0.50367\times \text{standardized methylation level of}\;\mathrm{SL}-2\right)\\ & +\left(-0.49917\times \text{standardized methylation level of}\;\mathrm{SL}-3\right)\\ & +\left(-0.49798\times \text{standardized methylation level of}\;\mathrm{SL}-4\right). \end{array}$$

**TABLE 4:** Mean absolute error (MAE), median absolute error (MedAE), and root mean square error (RMSE) values for Asian black bear‐specific age estimation model.

Model	Used CpGs	LOOCV model		
		MAE	MedAE	RMSE
Single regression	SL‐1	1.794	1.317	2.321
	SL‐2	1.580	1.419	1.970
	SL‐3	1.769	1.339	2.340
	SL‐4	**1.406**	**1.316**	**1.746**
Principal component regression	PC1	**1.337**	**1.167**	**1.699**
Elastic net regression	SL‐1, ‐2, ‐3, ‐4	**1.308**	**1.078**	**1.648**
Support vector regression	SL‐1, ‐2, ‐3, ‐4	1.163	0.819	1.557
	SL‐1, ‐2, ‐3	1.438	1.105	1.889
	SL‐1, ‐2, ‐4	**1.136**	**0.709**	1.529
	SL‐1, ‐3, ‐4	1.169	0.856	1.514
	SL‐2, ‐3, ‐4	1.421	1.073	1.804
	SL‐1, ‐2	1.669	1.145	2.113
	SL‐1, ‐3	1.464	1.014	1.917
	SL‐1, ‐4	1.183	0.800	1.557
	SL‐2, ‐3	1.467	1.080	1.833
	SL‐2, ‐4	1.160	0.848	1.523
	SL‐3, ‐4	1.227	0.862	**1.507**

*Note:* Each bold value denotes the minimum value for each model.

**FIGURE 2:**
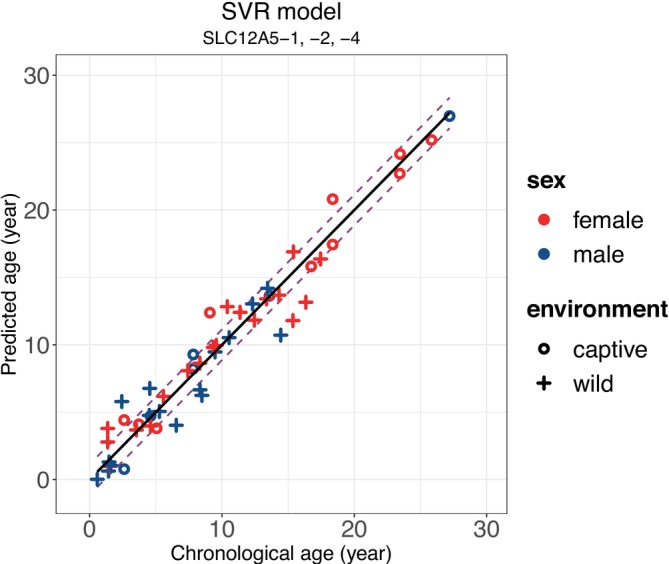
Scatter plots of predicted age and chronological age in the best age estimation model for Asian black bears (SVR model using SL‐1, ‐2, and ‐4) after LOOCV. The solid line represents predicted age = chronological age; distance between the dotted line and solid line represents the MAE of the model after LOOCV.

This first principal component explained 96.5% of the total variance in the data based on its cumulative proportion. Therefore, we constructed a principal component regression model using only this component. The SVR model using methylation levels of SL‐1, ‐2, and ‐4 was selected as the best model according to the values of MAE (1.136) and MedAE (0.709) after LOOCV (Figure 2), whereas RMSE (1.529) was the fourth lowest among the 17 models. Linear regression analysis revealed that none of the explanatory variables (i.e., age, sex, and growth environment [i.e., captive or wild condition], or their interactions) were statistically significant factors affecting Δage or |Δage| in the best model (Table S_R2). The R script used to construct models, estimate age, and evaluate factors capable of affecting the deviation of the age estimation model is available in Supporting Information. Details of the parameters used in the elastic net regression and SVR models are presented in Table S_R3.

### 3.4 Pan‐Bear Age Estimation Model

Similar to the model for Asian black bears, we constructed a total of 17 age estimation models (4 for single regression model, 1 for principal component regression, 1 for elastic net regression, and 11 for support vector regression models). Values of MAE, MedAE, and RMSE for elastic net regression and SVR models are presented in Table 5. Regarding principal component regression, the formula for the first principal component score is as follows:

$$\begin{array}{cc} \text{PC1}=\; & \left(0.49861\times \text{standardized methylation level of}\;\mathrm{SL}-1\right)\\ & +\left(0.50239\times \text{standardized methylation level of}\;\mathrm{SL}-2\right)\\ & +\left(0.49996\times \text{standardized methylation level of}\;\mathrm{SL}-3\right)\\ & +\left(0.49902\times \text{standardized methylation level of}\;\mathrm{SL}-4\right). \end{array}$$

**TABLE 5:** Mean absolute error (MAE), median absolute error (MedAE), and root mean square error (RMSE) values for a pan‐bear age estimation model.

Model	Used CpGs	LOOCV model		
		MAE	MedAE	RMSE
Single regression	SL‐1	2.294	1.959	2.873
	SL‐2	1.898	1.589	2.445
	SL‐3	2.075	1.515	2.683
	SL‐4	**1.663**	**1.310**	**2.149**
Principal component regression	PC1	**1.669**	**1.267**	**2.130**
Elastic net regression	SL‐1, ‐2, ‐3, ‐4	**1.573**	**1.172**	**2.044**
Support vector regression	SL‐1, ‐2, ‐3, ‐4	1.475	1.192	1.864
	SL‐1, ‐2, ‐3	1.654	1.383	2.076
	SL‐1, ‐2, ‐4	1.462	**1.044**	1.874
	SL‐1, ‐3, ‐4	**1.455**	1.183	**1.807**
	SL‐2, ‐3, ‐4	1.469	1.121	1.893
	SL‐1, ‐2	1.667	1.425	2.103
	SL‐1, ‐3	1.755	1.383	2.267
	SL‐1, ‐4	1.492	1.204	1.898
	SL‐2, ‐3	1.757	1.439	2.252
	SL‐2, ‐4	1.478	1.190	1.916
	SL‐3, ‐4	1.516	1.194	1.918

*Note:* Each bold value denotes the minimum value for each model.

**FIGURE 3:**
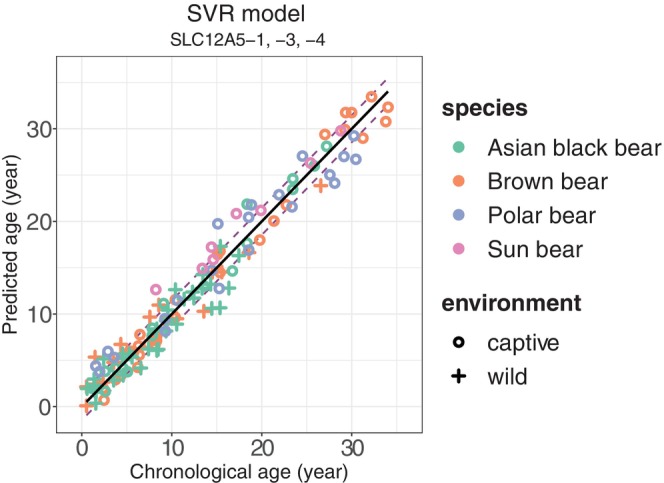
Scatter plots of predicted age and chronological age in the best pan‐bear age estimation model (SVR model using SL‐1, ‐3, and ‐4) after LOOCV. Solid line represents predicted age = chronological age; distance between the dotted line and solid line represents the MAE of the model after LOOCV.

This first principal component explained 97.4% of the total variance in the data based on its cumulative proportion. Therefore, we constructed a principal component regression model using only this component. The SVR model using methylation levels of SL‐1, ‐3, and ‐4 was selected as the best model according to the values of MAE (1.455) and RMSE (1.807) after LOOCV (Figure 3), whereas MedAE (1.183) was third lowest among the 17 models. The MAE, MedAE, and RMSE values after LOOCV in the best model are presented by species in Table 6. The one Andean bear (33.0 years old) had an estimated age of 31.4 years in the single regression model, 31.0 years in the principal component regression model, 31.4 years in the elastic net regression model, and 32.5 years in the SVR model.

**TABLE 6:** Mean absolute error (MAE), median absolute error (MedAE), and root mean square error (RMSE) values after leave‐one‐out cross‐validation for the pan‐bear best model.

Species	*N*	MAE	MedAE	RMSE
Brown bears	49	1.342	1.174	1.592
Asian black bears	52	1.199	0.967	1.590
Polar bears	21	2.107	1.905	2.398
Sun bears	8	2.111	1.413	2.458
Andean bears	1	0.521^a^	—^a^	—^a^

Abbreviation: *N*, number of samples.

^a^ Only one Andean bear was included. The MAE of the Andean bear was calculated based on predicted age by the pan‐bear age estimation model.

Linear regression analysis showed that some explanatory variables were statistically significant factors affecting Δage or |Δage| in this best model (Table 7). When Δage was used as the dependent variable, the best regression model included age, growth environment, species, and their interactions as explanatory variables (adjusted R^2^ = 0.2807) (Table 7). Among these variables, species (sun bears) had a significant positive effect, whereas growth environment (wild), the interaction between age and growth environment (wild), and the interaction between age and species (polar bears) had significant negative effects on Δage. When |Δage| was used as the dependent variable, the best regression model included growth environment, sex, and species as explanatory variables (adjusted R^2^ = 0.1796) (Table 7). Among these variables, growth environment (wild) and species (polar and sun bears) had significant positive effects, while the interaction between age and species (sun bears) had a significant negative effect on |Δage|. To focus on species differences, the values of Δage and |Δage|, generated by the SVR model using methylation levels of SL‐1, ‐3, and ‐4, were compared among the four species. This showed that Δage was significantly higher in sun bears than in the other three species (Figure S_R1; *p* < 0.05, Tukey's multiple comparison test), while |Δage| was significantly higher in polar bears than in brown and Asian black bears (*p* < 0.05, Tukey's multiple comparison test).

**TABLE 7:** Coefficient values and *p*‐values for the linear regression of Δage or |Δage| in the best pan‐bear age estimation model (the SVR model using SL‐1, ‐3, and ‐4).

	Estimate	*p*
Δage		
(Intercept)	0.03215	
Age	0.03051	0.40450
Growth environment (wild)	−0.13671	**0.00957**
Species (brown bears)	−0.05355	0.27253
Species (polar bears)	0.06217	0.26455
Species (sun bears)	0.26361	**0.00210**
Age: Growth environment (wild)	−0.17393	**0.00005**
Age: Species (brown bears)	−0.01602	0.68601
Age: Species (polar bears)	−0.17326	**0.00062**
Age: Species (sun bears)	−0.15708	0.07876
Growth environment (wild): Species (brown bears)	0.12308	0.09125
|Δage|		
(Intercept)	0.10550	
Age	0.02649	0.20231
Growth environment (wild)	0.05239	**0.03349**
Sex (male)	−0.02975	0.14216
Species (brown bears)	0.02143	0.35216
Species (polar bears)	0.12120	**0.00043**
Species (sun bears)	0.20236	**0.00021**
Age: Species (brown bears)	−0.01336	0.57560
Age: Species (polar bears)	−0.00868	0.77325
Age: Species (sun bears)	−0.14839	**0.01048**

*Note:* Bold values denote statistical significance (*p* < 0.05).

## 4 Discussion

The results of the present study showed that an epigenetic clock established for brown bears is also applicable to other bear species belonging to the subfamily Ursinae, including Asian black bears, polar bears, and sun bears, and probably with Andean bears belonging to the subfamily Tremarctinae. These bear species vary widely in body size (e.g., body mass: 27–65 kg in adult sun bears vs. > 500 kg in adult male polar bears), feeding ecology (e.g., omnivores and insectivorous in sun bears vs. carnivorous in polar bears), and habitat (e.g., tropical forests in sun bears vs. Arctic marine habitat in polar bears) (Rode et al. 2021; Scotson et al. 2021). One possible factor enabling them to share the same model is their similarity in lifespan. Although information on their lifespan in the wild is very limited, the five bear species examined here generally live 20–30 years in captivity with the oldest records of 40, 39.2, 43.8, 35.9, and 39 years for brown, Asian black, polar, sun, and Andean bears, respectively (AnAge). Three other bear species have similar lifespans, with oldest records of 34, 33, and 36.8 years for American black bears, sloth bears, and giant pandas, respectively (AnAge), further suggesting that the brown bear model would be applicable to all bear species.

Another, more likely factor enabling the use of the same model is the high degree of evolutionary conservation of target CpGs adjacent to *SLC12A5* (hereafter, simply referred to as *SLC12A5*) as markers of aging. *SLC12A5* blood DNA methylation level has been reported to show an extremely strong correlation with age and can be used as a marker of aging in Carnivora, including domestic dogs (Horvath et al. 2022; Ito et al. 2017), domestic cats (Raj et al. 2021), Pacific walrus ( *Odobenus rosmarus divergens* ), and harbor seals ( *Phoca vitulina* ) (Robeck et al. 2023), as well as in humans (Florath et al. 2014; Horvath et al. 2022). In fact, more than 10,000 methylation sites are conserved and can be used as age markers among mammals with highly different life histories (i.e., universal pan‐mammalian epigenetic clock; Lu et al. 2023). Among these age markers, an advantage of *SLC12A5* is the high rate of increase in methylation with aging; in our previous study in the brown bear, the methylation levels of the four CpGs in *SLC12A5* showed changes of 1.34%–1.68% with age, whereas another nine CpGs adjacent to three genes that also showed correlations with age but were excluded from the best model showed changes of 0.36%–0.93% with age (Nakamura et al. 2023). The four CpGs, with high rates of age‐related changes in methylation level, provided a robust age estimation model by counteracting potential differences in pace of aging between related species and the effects of measurement errors of a few percent, which are inevitable with pyrosequencing. Taken together, our results imply that the age‐estimation model that targets only one DNA region can be widely applied as a highly accurate and cost‐effective tool in other mammals, especially those of the Carnivora. Furthermore, it can be used to establish a simple, cost‐effective epigenetic clock that can be shared without calibration among related species with similar life spans, once good age markers, such as *SLC12A5*, are obtained.

Among the Asian black, polar, and sun bears, the age estimation model for brown bears showed the best fit for Asian black bears, and the worst fit, although still satisfactory, for polar bears. The MAE in Asian black bears was comparable to or less than in brown bears in three models, whereas those in polar bears were 0.6–0.9 years higher than in brown bears. These results were paradoxical compared to the phylogenetic relationships among bear species. Phylogenomic analysis suggested that polar bears are most closely related to brown bears and are grouped within the same clade as American black bears, while Asian black bears and sun bears are grouped in a different clade along with sloth bears (Kumar et al. 2017). One possible reason is that aged individuals comprised a large proportion of the polar bear dataset. Older individuals tended to have larger |Δage| in Felidae species (Qi et al. 2024), and specifically, many previous studies (El Khoury et al. 2019; Prado et al. 2021; Qi et al. 2024; Raj et al. 2021) reported that the age estimation model based on DNA methylation tended to underestimate the age of older individuals. This was also supported by the pan‐bear age estimation model established in this study. Indeed, nine of the 27 polar bear samples were over 20 years old, and |Δage| and Δage of these samples in the SVR model were 2.78 and −1.91 on average, respectively, whereas those of samples under 20 years old were 1.83 and 1.41 on average, respectively. Such sampling bias also existed for sun bears and is inevitable in captive animals in zoos, which may have affected the MAE in this study.

We established the first epigenetic clock specific to Asian black bears. Evaluation based on the MAE of LOOCV suggested that the SVR model using 3 CpGs (i.e., SL‐1, ‐2, and ‐4) is the best model for this species. The MAE and MedAE values of LOOCV (1.136 and 0.709, respectively) were comparable or superior to those in the best model in brown bears (1.304 and 1.000, respectively), and also to age estimation models for other carnivores with different lifespans, including dogs (Horvath et al. 2022), cats (Raj et al. 2021), suggesting that the model was highly accurate. In addition, sex and environmental conditions (i.e., captive or wild) had no effect on Δage or |Δage| in the best model. Furthermore, only two Himalayan black bears were included in the study, but they showed low MAE values. These observations suggested that the established model can be applied to other subspecies of Asian black bears regardless of sex and environmental conditions. In Japan, a large number of Japanese black bears are killed annually (1224–7650 bears per year during 2014–2023; Ministry of the Environment Japan, 2024: https://www.env.go.jp/nature/choju/effort/effort12/effort12.html) mainly for management purposes. However, monitoring of demographic trends has not been carried out in most areas. The method outlined here can provide an understanding of the age structure of harvested bears, which will help to establish appropriate management plans. In contrast, on the eastern Asian continent, Asian black bears are threatened by human activities, such as poaching and habitat fragmentation (Kozakai et al. 2021). Our method will be helpful to provide appropriate health care according to age for bears rescued from the wild or poor captive conditions, such as bile farms that are still common in East and Southeast Asia (Kalogeropoulu et al. 2023).

Finally, we established the first pan‐bear epigenetic clock model across all targeted bear species, except for Andean bears. The best model was constructed using three CpGs (i.e., SL‐1, ‐3, and ‐4) in the SVR model, which was different from the best brown bear‐specific model (4 CpGs in the SVR model) and that in the Asian black bear‐specific model (3 CpGs [SL‐1, ‐2, and ‐4] in the SVR model). However, the performance of the common model was still good enough and was comparable even to these species‐specific models (i.e., the MAE values after LOOCV were 1.342 and 1.199 in the pan‐bear model and 1.304 and 1.136 in the species‐specific models for brown and Asian black bears, respectively). Furthermore, in polar bears and sun bears in which species‐specific models could not be constructed due to small sample sizes, the MAE decreased slightly from the values in the brown bear‐specific model (2.144 and 2.164 for polar and sun bears, respectively, in the SVR model) to those in the common model (2.107 and 2.111 after LOOCV for polar and sun bears, respectively). These observations suggested that the brown bear‐specific model is still useful, but the pan‐bear model is recommended for age estimation of polar and sun bears as well as other Ursidae species, including American black, sloth, and Andean bears and giant pandas.

In the best pan‐bear model, linear regression analysis revealed that species is one of the factors affecting Δage or |Δage|; sun bears had higher Δage than the other three bear species, while polar bears had larger |Δage| than brown and Asian black bears. Indeed, Δage values of all sun bears were positive, suggesting overestimation of their ages. This may indicate subtle species differences in the baseline methylation levels and/or in the progression of methylation levels with age. However, in this study, the numbers of sun bears and polar bears included in model construction were smaller than for the other two species. In addition, as mentioned above, polar bear samples were biased toward older individuals that tended to have greater |Δage| in the studies of other species (Qi et al. 2024). Therefore, such bias in sample size and age may have had a greater impact on our results. It was also suggested that the growth environment (i.e., captive or wild conditions) was another factor affecting Δage or |Δage|. However, wild bears were included only in brown bears and Asian black bears in this study. Likewise, the current dataset contained significant species‐specific bias in age and sex class and growth environment (captive or wild), as well as differences in the number of samples by species, making it difficult to identify environmental factors affecting epigenetic aging. Further studies are needed to clarify this issue.

In conclusion, we confirmed that the epigenetic clock established for brown bears was applicable to other bear species, including Asian black bears, polar bears, and sun bears, and likely also to Andean bears. Furthermore, we built a common epigenetic clock model for these bear species, suggesting that a pan‐bear model would also be useful for other bear species not included in this study. To our knowledge, this is the only epigenetic clock for wildlife capable of providing accurate and precise age estimation by targeting only one DNA region including four CpGs. The current method is superior to other methylation‐based models, such as the HorvathMammalMethylChip40 mammalian DNA methylation microarray (Arneson et al. 2022) in terms of simplicity of methodology and cost‐effectiveness, which is beneficial for studies with larger sample sizes and/or budget limitations. Moreover, it is likely that the current method will also be applicable to other carnivores as a simple, accurate, and less costly means of estimating age, although calibration may be needed according to the lifespan of target species. However, it is necessary to develop epigenetic clocks using samples that can be collected noninvasively, such as feces, because collecting blood samples from wild carnivores is difficult and invasive. As most large carnivores are threatened with extinction and have declining population trends (Wolf and Ripple 2018), the method outlined here will contribute to ecological research, conservation, and management of these species.

## Author Contributions

**Michito Shimozuru:** conceptualization (lead), data curation (equal), formal analysis (equal), investigation (equal), methodology (equal), project administration (lead), visualization (equal), writing – original draft (equal). **Shiori Nakamura:** conceptualization (supporting), data curation (lead), formal analysis (equal), investigation (equal), methodology (equal), validation (equal), visualization (equal), writing – original draft (equal). **Jumpei Yamazaki:** funding acquisition (equal), methodology (equal), resources (equal), writing – review and editing (equal). **Yojiro Yanagawa:** investigation (equal), resources (equal), writing – review and editing (supporting). **Hiroo Tamatani:** resources (equal), writing – review and editing (supporting). **Misako Kuroe:** resources (equal), writing – review and editing (supporting). **Koji Yamazaki:** investigation (equal), resources (equal), writing – review and editing (equal). **Shinsuke Koike:** funding acquisition (equal), investigation (equal), resources (equal), writing – review and editing (equal). **Yusuke Goto:** investigation (supporting), resources (supporting), writing – review and editing (supporting). **Tomoko Naganuma:** investigation (supporting), resources (supporting), writing – review and editing (supporting). **Kahoko Tochigi:** investigation (supporting), resources (supporting), writing – review and editing (supporting). **Akino Inagaki:** investigation (supporting), resources (supporting), writing – review and editing (supporting). **Naoki Takekoshi:** investigation (supporting), resources (supporting), writing – review and editing (supporting). **Seungyun Baek:** investigation (supporting), resources (supporting), writing – review and editing (supporting). **Nobutaka Sato:** resources (equal). **Yusuke Honda:** resources (equal). **Toshio Tsubota:** supervision (equal), writing – review and editing (equal). **Hideyuki Ito:** conceptualization (equal), funding acquisition (equal), supervision (equal), writing – review and editing (equal).

## Conflicts of Interest

The authors declare no conflicts of interest.

## Supporting information


Appendix S1.

Appendix S2.

Appendix S3.

## Data Availability

Additional supporting information are uploaded as [Link], [Link], [Link]. The data obtained from pyrosequencing analyses are available in Dryad at: DOI: 10.5061/dryad.b5mkkwhqt.
