# Supplementary material for: Epigenetic Clock in Bears: A Simple Cost‐Effective Blood DNA Methylation‐Based Age Estimation Method Applicable to Multiple Bear Species
Source: Ecol Evol. 2025 May 6;15(5):e71424. doi: 10.1002/ece3.71424 (PMC12055220; doi:10.1002/ece3.71424)
Supplement: Supplementary file 1 — Appendix S1. [file ECE3-15-e71424-s001.docx]

Supplementary file_R script_Asian black bear

**A Simple Cost-Effective Blood DNA Methylation-Based Age Estimation Method Applicable to Multiple Bear Species**

Michito Shimozuru, Shiori Nakamura, Jumpei Yamazaki, Yojiro Yanagawa, Hiroo Tamatani, Misako Kuroe, Koji Yamazaki, Shinsuke Koike, Yusuke Goto, Tomoko Naganuma, Kahoko Tochigi, Akino Inagaki, Naoki Takekoshi, Seungyun Baek, Nobutaka Sato, Yusuke Honda, Toshio Tsubota, Hideyuki Ito

Principal component regression (PC1)

- [Load in packages](#Load_in_packages)
- [Data input](#Data_input)
- [The correlation between methylation level and age](#The_correlation_between_methyltion_level)
- [Age estimation model 【Single regression】](#Single_regression)
  - [Single regression (SLC12A5-1)](#S_1)
  - [Single regression (SLC12A5-2)](#S_2)
  - [Single regression (SLC12A5-3)](#S_3)
  - [Single regression (SLC12A5-4)](#S_4)
- [Age estimation model 【Principal component regression (PC1)】](#Age_estimation_model_【PCR】)
  - [Principal component regression (PC1)](#PC1)
- [Age estimation model 【Elastic net regression】](#Age_estimation_model_【Elastic_net_regres)

- - [Elastic net regression (SLC12A5-1, -2, -3, -4)](#EN_1234)
- [Age estimation model 【Support vector regression】](#Age_estimation_model_【Suport_vector_regr)
  - [Support vector regression (SLC12A5-1, -2, -3, -4)](#SVR_1234)
  - [Support vector regression (SLC12A5-1, -2, -3)](#SVR_123)
  - [Support vector regression (SLC12A5-1, -2, -4)](#SVR_124)
  - [Support vector regression (SLC12A5-1, -3, -4)](#SVR_134)
  - [Support vector regression (SLC12A5-2, -3, -4)](#SVR_234)
  - [Support vector regression (SLC12A5-1, -2)](#SVR_12)
  - [Support vector regression (SLC12A5-1, -3)](#SVR_13)
  - [Support vector regression (SLC12A5-1, -4)](#SVR_14)
  - [Support vector regression (SLC12A5-2, -3)](#SVR_23)
  - [Support vector regression (SLC12A5-2, -4)](#SVR_24)
  - [Support vector regression (SLC12A5-3, -4)](#SVR_34)
- [Influences of interaction among age, sex, and growth environment](#Influences_of_interaction_among_age_sex)
  - [Single regression (SLC12A5-4)](#I_S)
  - [Principal component regression (PC1)](#I_PC1)
  - [Elastic net regression (SLC12A5-1, -2, -3, -4)](#I_EN3)
  - [Support vector regression (SLC12A5-1, -2, -4)](#I_SVR3)
  - [Support vector regression (SLC12A5-3, -4)](#I_SVR2)
- [How to apply to the models](#How_to_apply_to_the_models)
  - [Single regression (SLC12A5-4)](#H_S)
  - [Principal component regression (PC1)](#H_PC1)
  - [Elastic net regression (SLC12A5-1, -2, -3, -4)](#H_E)
  - [Support vector regression (SLC12A5-1, -2, -4)](#H_SVR)
  - [Output to a csv file](#Output_to_a_csv_file)

Load in packages

library(dplyr)

library(MuMIn)

library(glmnet)

library(e1071)

library(ggplot2)

library(car)

Data input

ABBB<-read.csv("Asian_black_bear_blood.csv")

ABBBS<-read.csv("Asian_black_bear_blood_standardized.csv")

The correlation between methylation level and age

#SLC12A5-1

cor.test(ABBB$age,ABBB$SLC12A5_1_methylation_rate_ave)

Pearson's product-moment correlation

data: ABBB$age and ABBB$SLC12A5_1_methylation_rate_ave

t = 19.995, df = 50, p-value < 2.2e-16

alternative hypothesis: true correlation is not equal to 0

95 percent confidence interval:

0.9019420 0.9669121

sample estimates:

cor

0.942785

SLC1<-ggplot(ABBB,aes(x=age,y=SLC12A5_1_methylation_rate_ave))+theme_bw()+

geom_point(aes(shape=environment,color=sex),size=2,stroke=2)+

labs(x="Age (year)",y="DNA methylation (%)")+

scale_shape_manual(name="environment",labels=c("Captive"="captive","Wild"="wild"),values=c("Captive"=1, "Wild"=3))+

scale_color_manual(name="sex",labels=c("F"="female","M"="male"),values=c("F"="firebrick2","M"="dodgerblue4"))+

theme(axis.text.x=element_text(size=20),axis.text.y=element_text(size=20))+

theme(axis.title.x=element_text(size=17),axis.title.y=element_text(size=17))+

annotate("text",size=6,x=-Inf,y=Inf,hjust=-.1,vjust=2,label="R=0.94, p<0.001")+

labs (title="SLC12A5-1")+

theme(plot.title=element_text(size=20,hjust = 0.5))


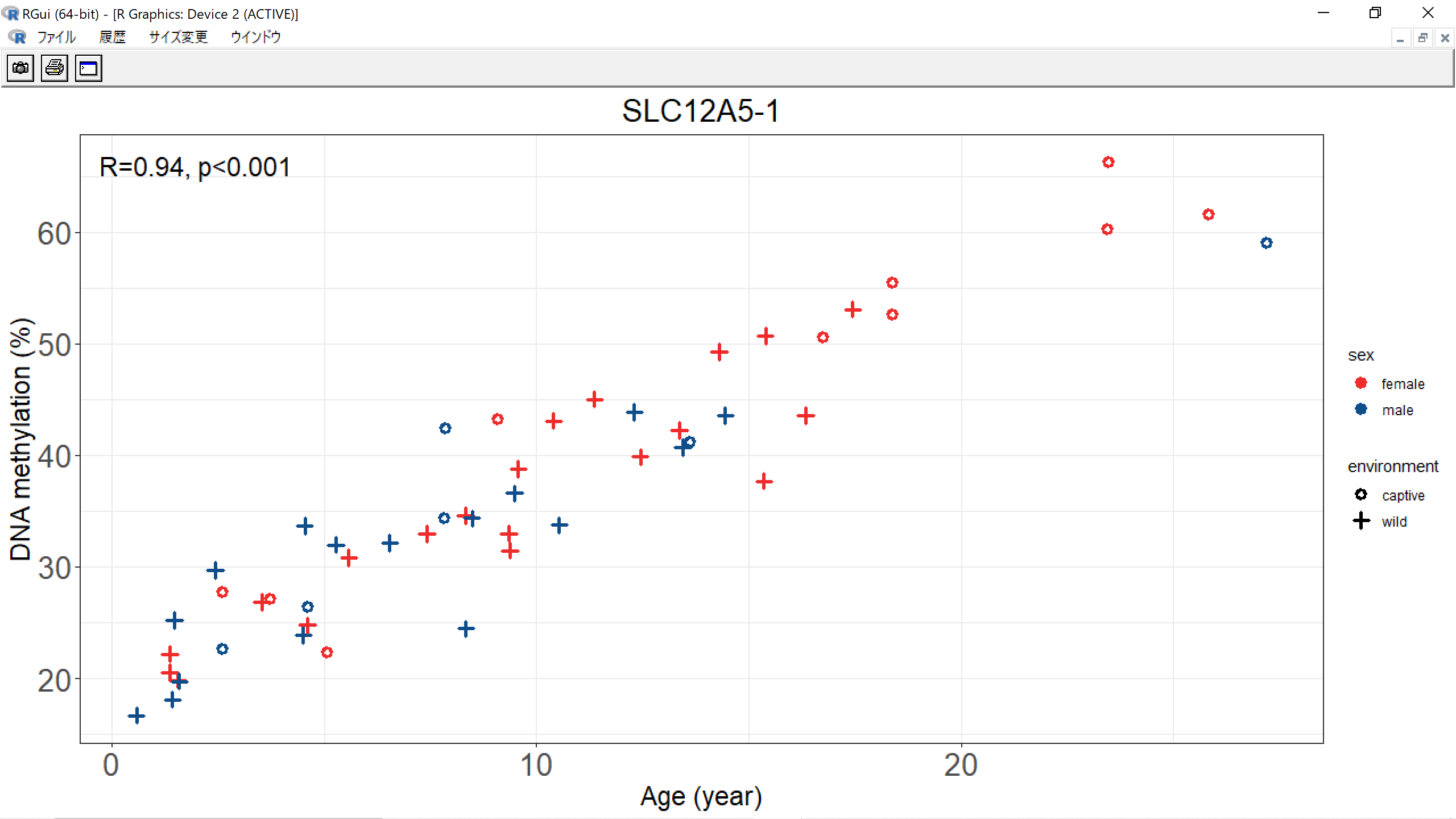


#SLC12A5-2

cor.test(ABBB$age,ABBB$SLC12A5_2_methylation_rate_ave)

Pearson's product-moment correlation

data: ABBB$age and ABBB$SLC12A5_2_methylation_rate_ave

t = 23.76, df = 50, p-value < 2.2e-16

alternative hypothesis: true correlation is not equal to 0

95 percent confidence interval:

0.9283866 0.9760559

sample estimates:

cor

0.9584555

SLC2<-ggplot(ABBB,aes(x=age,y=SLC12A5_2_methylation_rate_ave))+theme_bw()+

geom_point(aes(shape=environment,color=sex),size=2,stroke=2)+

labs(x="Age (year)",y="DNA methylation (%)")+

scale_shape_manual(name="environment",labels=c("Captive"="captive","Wild"="wild"),values=c("Captive"=1, "Wild"=3))+

scale_color_manual(name="sex",labels=c("F"="female","M"="male"),values=c("F"="firebrick2","M"="dodgerblue4"))+

theme(axis.text.x=element_text(size=20),axis.text.y=element_text(size=20))+

theme(axis.title.x=element_text(size=17),axis.title.y=element_text(size=17))+

annotate("text",size=6,x=-Inf,y=Inf,hjust=-.1,vjust=2,label="R=0.96, p<0.001")+

labs (title="SLC12A5-2")+

theme(plot.title=element_text(size=20,hjust = 0.5))


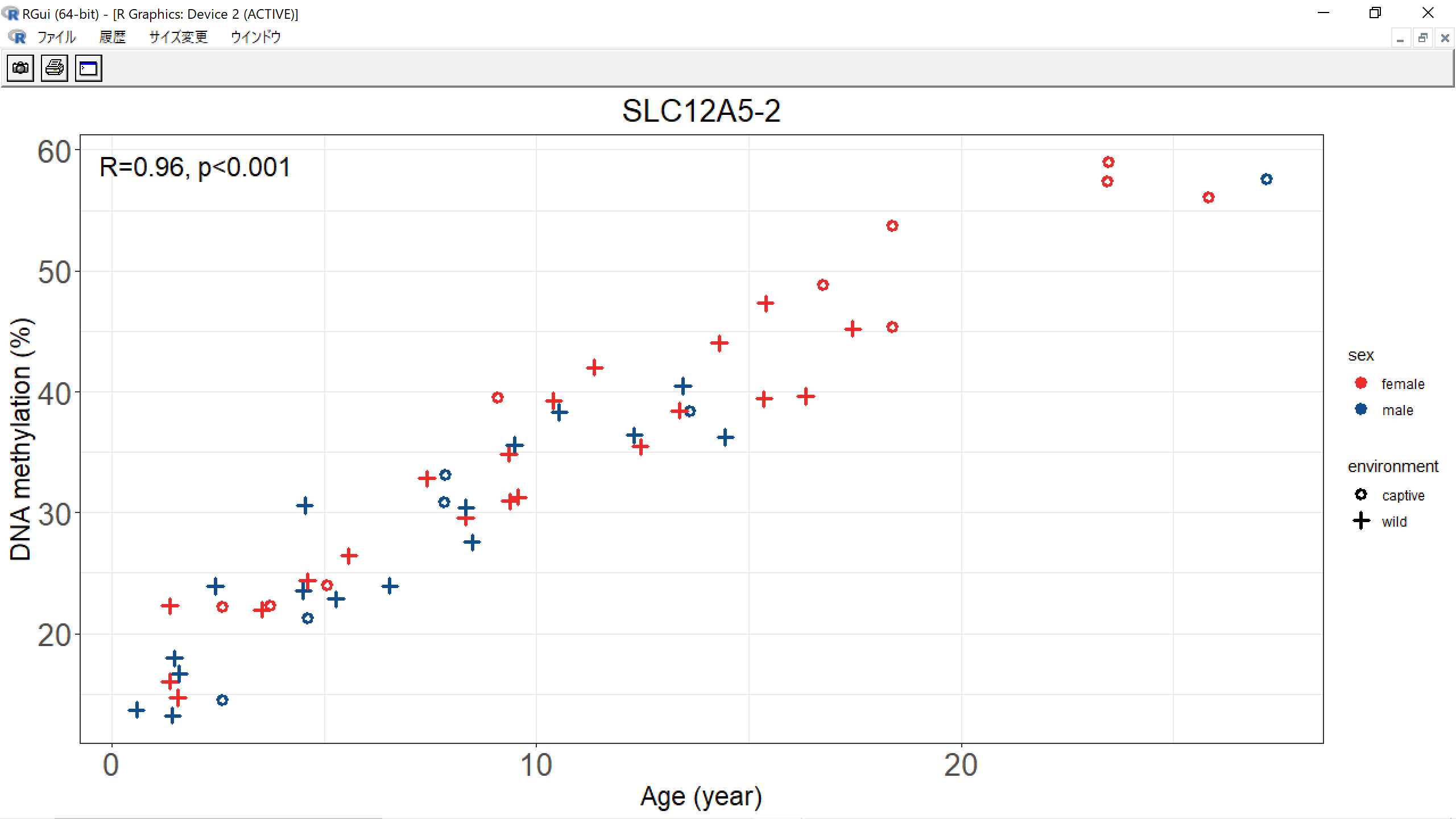


#SLC12A5-3

cor.test(ABBB$age,ABBB$SLC12A5_3_methylation_rate_ave)

Pearson's product-moment correlation

data: ABBB$age and ABBB$SLC12A5_3_methylation_rate_ave

t = 20.115, df = 50, p-value < 2.2e-16

alternative hypothesis: true correlation is not equal to 0

95 percent confidence interval:

0.9029855 0.9672760

sample estimates:

cor

0.9434067

SLC3<-ggplot(ABBB,aes(x=age,y=SLC12A5_3_methylation_rate_ave))+theme_bw()+

geom_point(aes(shape=environment,color=sex),size=2,stroke=2)+

labs(x="Age (year)",y="DNA methylation (%)")+

scale_shape_manual(name="environment",labels=c("Captive"="captive","Wild"="wild"),values=c("Captive"=1, "Wild"=3))+

scale_color_manual(name="sex",labels=c("F"="female","M"="male"),values=c("F"="firebrick2","M"="dodgerblue4"))+

theme(axis.text.x=element_text(size=20),axis.text.y=element_text(size=20))+

theme(axis.title.x=element_text(size=17),axis.title.y=element_text(size=17))+

annotate("text",size=6,x=-Inf,y=Inf,hjust=-.1,vjust=2,label="R=0.94, p<0.001")+

labs (title="SLC12A5-3")+

theme(plot.title=element_text(size=20,hjust = 0.5))


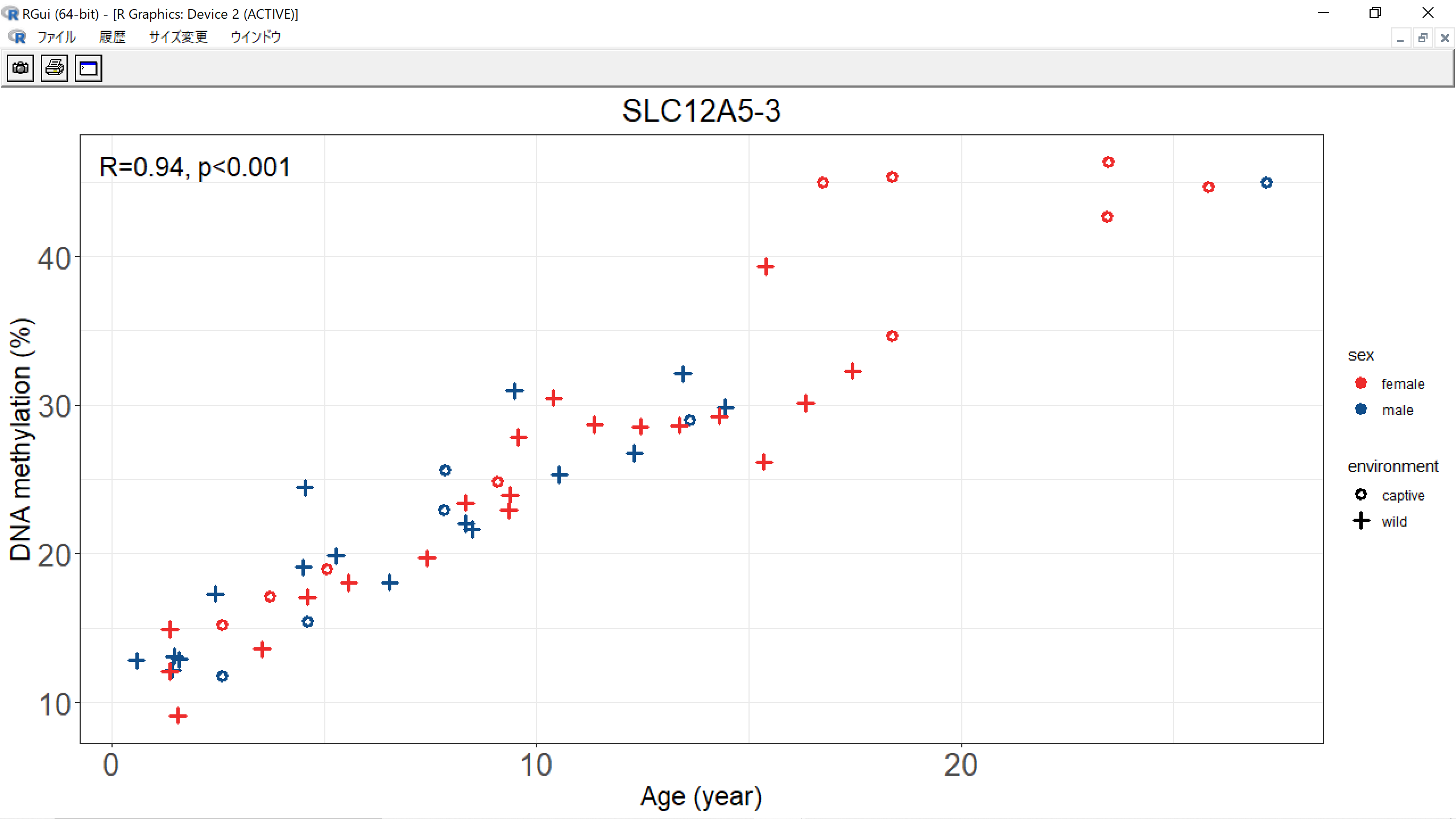


#SLC12A5-4

cor.test(ABBB$age,ABBB$SLC12A5_4_methylation_rate_ave)

Pearson's product-moment correlation

data: ABBB$age and ABBB$SLC12A5_4_methylation_rate_ave

t = 26.846, df = 50, p-value < 2.2e-16

alternative hypothesis: true correlation is not equal to 0

95 percent confidence interval:

0.9429683 0.9810268

sample estimates:

cor

0.9670194

SLC4<-ggplot(ABBB,aes(x=age,y=SLC12A5_4_methylation_rate_ave))+theme_bw()+

geom_point(aes(shape=environment,color=sex),size=2,stroke=2)+

labs(x="Age (year)",y="DNA methylation (%)")+

scale_shape_manual(name="environment",labels=c("Captive"="captive","Wild"="wild"),values=c("Captive"=1, "Wild"=3))+

scale_color_manual(name="sex",labels=c("F"="female","M"="male"),values=c("F"="firebrick2","M"="dodgerblue4"))+

theme(axis.text.x=element_text(size=20),axis.text.y=element_text(size=20))+

theme(axis.title.x=element_text(size=17),axis.title.y=element_text(size=17))+

annotate("text",size=6,x=-Inf,y=Inf,hjust=-.1,vjust=2,label="R=0.97, p<0.001")+

labs (title="SLC12A5-4")+

theme(plot.title=element_text(size=20,hjust = 0.5))


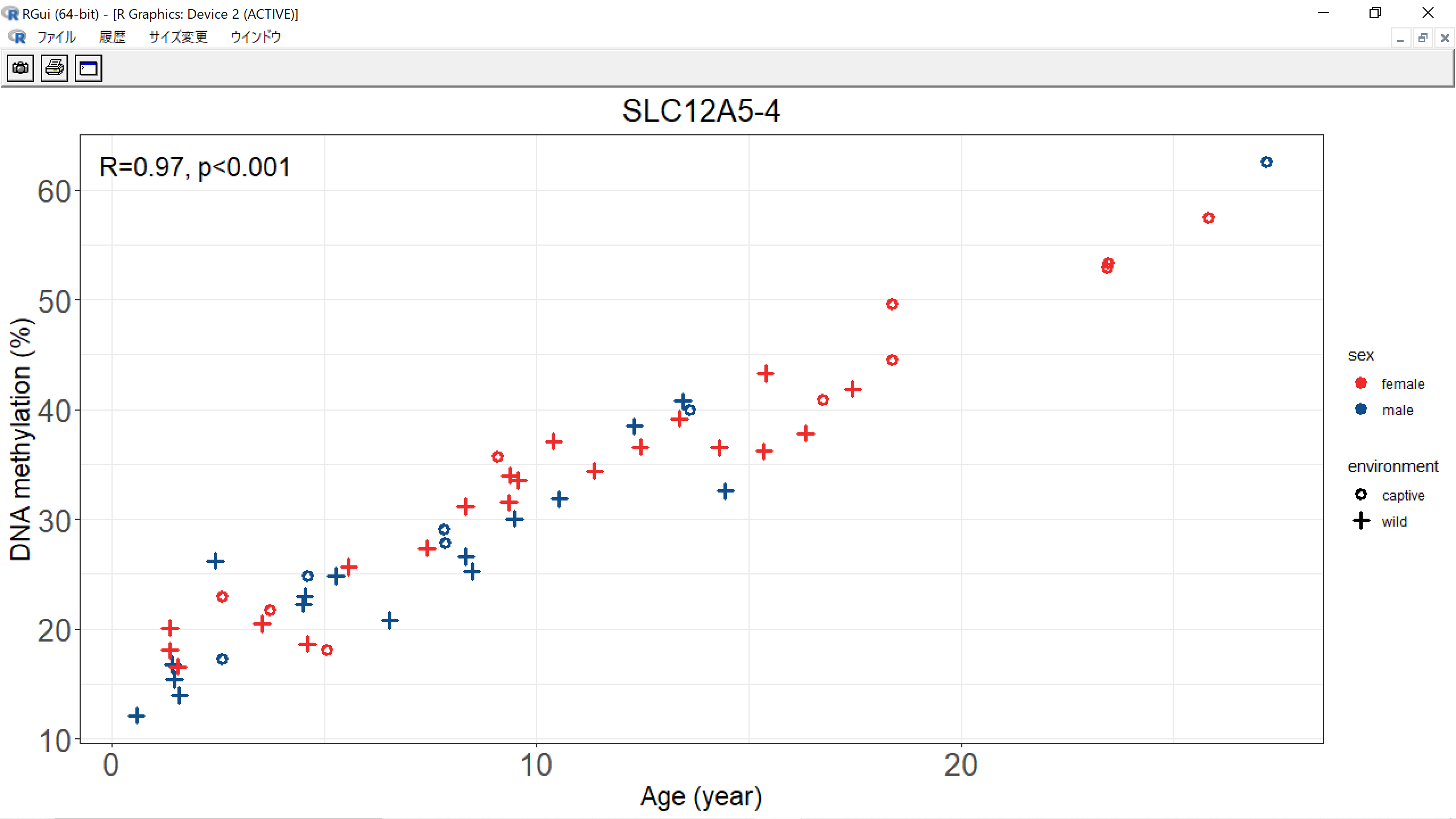


Age estimation model 【Single regression】

YS <- ABBBS$age

slc1S <- ABBBS$SLC12A5_1_methylation_rate_ave

slc2S <- ABBBS$SLC12A5_2_methylation_rate_ave

slc3S <- ABBBS$SLC12A5_3_methylation_rate_ave

slc4S <- ABBBS$SLC12A5_4_methylation_rate_ave

AGE <- ABBB$age

#RemoveOne function

removeOne <- function(dat,x) {

if(x<dat){

list=seq(1,dat)

x1=x-1;x2=x+1

v1=c(list[0:x1]);v2=c(list[x2:dat])

data=c(v1,v2)}

else {data=seq(1,dat-1)}

return (data)}

Single regression (SLC12A5-1)

SRM_SLC_1<-lm(formula=YS~slc1S,data=ABBBS)

coef(SRM_SLC_1)

(Intercept) slc1S

-3.071178e-10 9.427850e-01

#LOOCV

nSamples<-nrow(ABBBS)

predict_SRM_SLC_1_loocv<-numeric(nSamples)

for (z in 1:nSamples){

indices<-removeOne(nSamples,z)

dr<-data.frame(ABBBS$age[indices],ABBBS$SLC12A5_1_methylation_rate_ave[indices])

colnames(dr)<-c("age","methylslc_1")

bestmodel_SRM_SLC_1_loocv<-lm(age~methylslc_1,data=dr)

newdata<-data.frame(methylslc_1=ABBBS$SLC12A5_1_methylation_rate_ave[z])

p<-predict(bestmodel_SRM_SLC_1_loocv,newdata)*sd(AGE)+mean(AGE)

if (p<0){p=0}

predict_SRM_SLC_1_loocv[z]<-p}

ABBB_predict_SRM_SLC_1_loocv<-cbind(ABBB,predict_SRM_SLC_1_loocv)

MAE_SRM_SLC_1_loocv<-mean(abs(ABBB_predict_SRM_SLC_1_loocv$predict_SRM_SLC_1_loocv-ABBB_predict_SRM_SLC_1_loocv$age))

MedianAE_SRM_SLC_1_loocv<-median(abs(ABBB_predict_SRM_SLC_1_loocv$predict_SRM_SLC_1_loocv-ABBB_predict_SRM_SLC_1_loocv$age))

RMSE_SRM_SLC_1_loocv<-sqrt(mean((ABBB_predict_SRM_SLC_1_loocv$predict_SRM_SLC_1_loocv-ABBB_predict_SRM_SLC_1_loocv$age)^2))

cat("MAE:", MAE_SRM_SLC_1_loocv, "\nMed AE:", MedianAE_SRM_SLC_1_loocv, "\nRMSE:", RMSE_SRM_SLC_1_loocv, "\n")

MAE: 1.793596

Med AE: 1.317445

RMSE: 2.321244

g_SRM_SLC_1_loocv<-ggplot(ABBB_predict_SRM_SLC_1_loocv,aes(age,predict_SRM_SLC_1_loocv))+theme_bw()+

annotate("segment",x=min(ABBB$age),xend=max(ABBB$age),y=min(ABBB$age)+1.793596,yend=max(ABBB$age)+1.793596,colour="orchid4",linetype=2, linewidth =0.7)+

annotate("segment",x=min(ABBB$age),xend=max(ABBB$age),y=min(ABBB$age)-1.793596,yend=max(ABBB$age)-1.793596,colour="orchid4",linetype=2, linewidth =0.7)+

geom_point(aes(shape=environment,color=sex),size=2,stroke=2)+

labs(x="Chronological age (year)",y="Predicted age (year)")+

scale_shape_manual(name="environment",labels=c("Captive"="captive","Wild"="wild"),values=c("Captive"=1, "Wild"=3))+

scale_color_manual(name="sex",labels=c("F"="female","M"="male"),values=c("F"="firebrick2","M"="dodgerblue4"))+

theme(axis.text.x=element_text(size=20),axis.text.y=element_text(size=20))+

theme(axis.title.x=element_text(size=17),axis.title.y=element_text(size=17))+

geom_line(aes(y =age), linewidth=1)+

labs(title="single regression model")+

theme(title=element_text(size=17),plot.title=element_text(hjust=0.5))+

scale_y_continuous(limits=c(-5,40))+

scale_x_continuous(limits=c(-5,40))+

labs(subtitle="SLC12A5-1")+

theme(plot.subtitle=element_text(size=15,hjust=0.5))


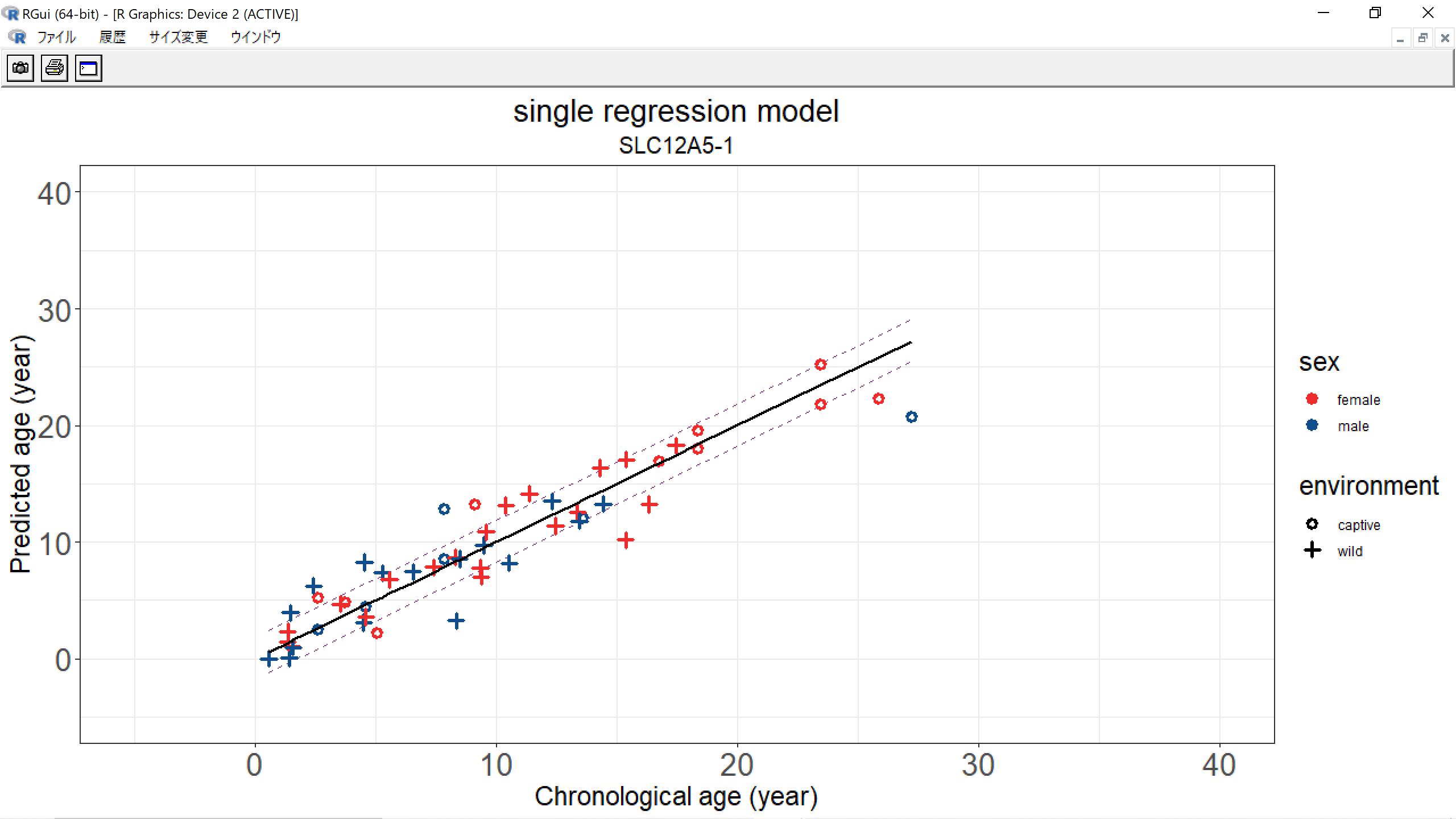


Single regression (SLC12A5-2)

SRM_SLC_2<-lm(formula=YS~slc2S,data=ABBBS)

coef(SRM_SLC_2)

(Intercept) slc2S

7.988770e-13 9.584555e-01

#LOOCV

nSamples<-nrow(ABBBS)

predict_SRM_SLC_2_loocv<-numeric(nSamples)

for (z in 1:nSamples){

indices<-removeOne(nSamples,z)

dr<-data.frame(ABBBS$age[indices],ABBBS$SLC12A5_2_methylation_rate_ave[indices])

colnames(dr)<-c("age","methylslc_2")

bestmodel_SRM_SLC_2_loocv<-lm(age~methylslc_2,data=dr)

newdata<-data.frame(methylslc_2=ABBBS$SLC12A5_2_methylation_rate_ave[z])

p<-predict(bestmodel_SRM_SLC_2_loocv,newdata)*sd(AGE)+mean(AGE)

if (p<0){p=0}

predict_SRM_SLC_2_loocv[z]<-p}

ABBB_predict_SRM_SLC_2_loocv<-cbind(ABBB,predict_SRM_SLC_2_loocv)

MAE_SRM_SLC_2_loocv<-mean(abs(ABBB_predict_SRM_SLC_2_loocv$predict_SRM_SLC_2_loocv-ABBB_predict_SRM_SLC_2_loocv$age))

MedianAE_SRM_SLC_2_loocv<-median(abs(ABBB_predict_SRM_SLC_2_loocv$predict_SRM_SLC_2_loocv-ABBB_predict_SRM_SLC_2_loocv$age))

RMSE_SRM_SLC_2_loocv<-sqrt(mean((ABBB_predict_SRM_SLC_2_loocv$predict_SRM_SLC_2_loocv-ABBB_predict_SRM_SLC_2_loocv$age)^2))

cat("MAE:", MAE_SRM_SLC_2_loocv, "\nMed AE:", MedianAE_SRM_SLC_2_loocv, "\nRMSE:", RMSE_SRM_SLC_2_loocv, "\n")

MAE: 1.579626

Med AE: 1.419156

RMSE: 1.969631

g_SRM_SLC_2_loocv<-ggplot(ABBB_predict_SRM_SLC_2_loocv,aes(age,predict_SRM_SLC_2_loocv))+theme_bw()+

annotate("segment",x=min(ABBB$age),xend=max(ABBB$age),y=min(ABBB$age)+1.579626,yend=max(ABBB$age)+1.579626,colour="orchid4",linetype=2, linewidth =0.7)+

annotate("segment",x=min(ABBB$age),xend=max(ABBB$age),y=min(ABBB$age)-1.579626,yend=max(ABBB$age)-1.579626,colour="orchid4",linetype=2, linewidth =0.7)+

geom_point(aes(shape=environment,color=sex),size=2,stroke=2)+

labs(x="Chronological age (year)",y="Predicted age (year)")+

scale_shape_manual(name="environment",labels=c("Captive"="captive","Wild"="wild"),values=c("Captive"=1, "Wild"=3))+

scale_color_manual(name="sex",labels=c("F"="female","M"="male"),values=c("F"="firebrick2","M"="dodgerblue4"))+

theme(axis.text.x=element_text(size=20),axis.text.y=element_text(size=20))+

theme(axis.title.x=element_text(size=17),axis.title.y=element_text(size=17))+

geom_line(aes(y =age), linewidth=1)+

labs(title="single regression model")+

theme(title=element_text(size=17),plot.title=element_text(hjust=0.5))+

scale_y_continuous(limits=c(-5,40))+

scale_x_continuous(limits=c(-5,40))+

labs(subtitle="SLC12A5-2")+

theme(plot.subtitle=element_text(size=15,hjust=0.5))


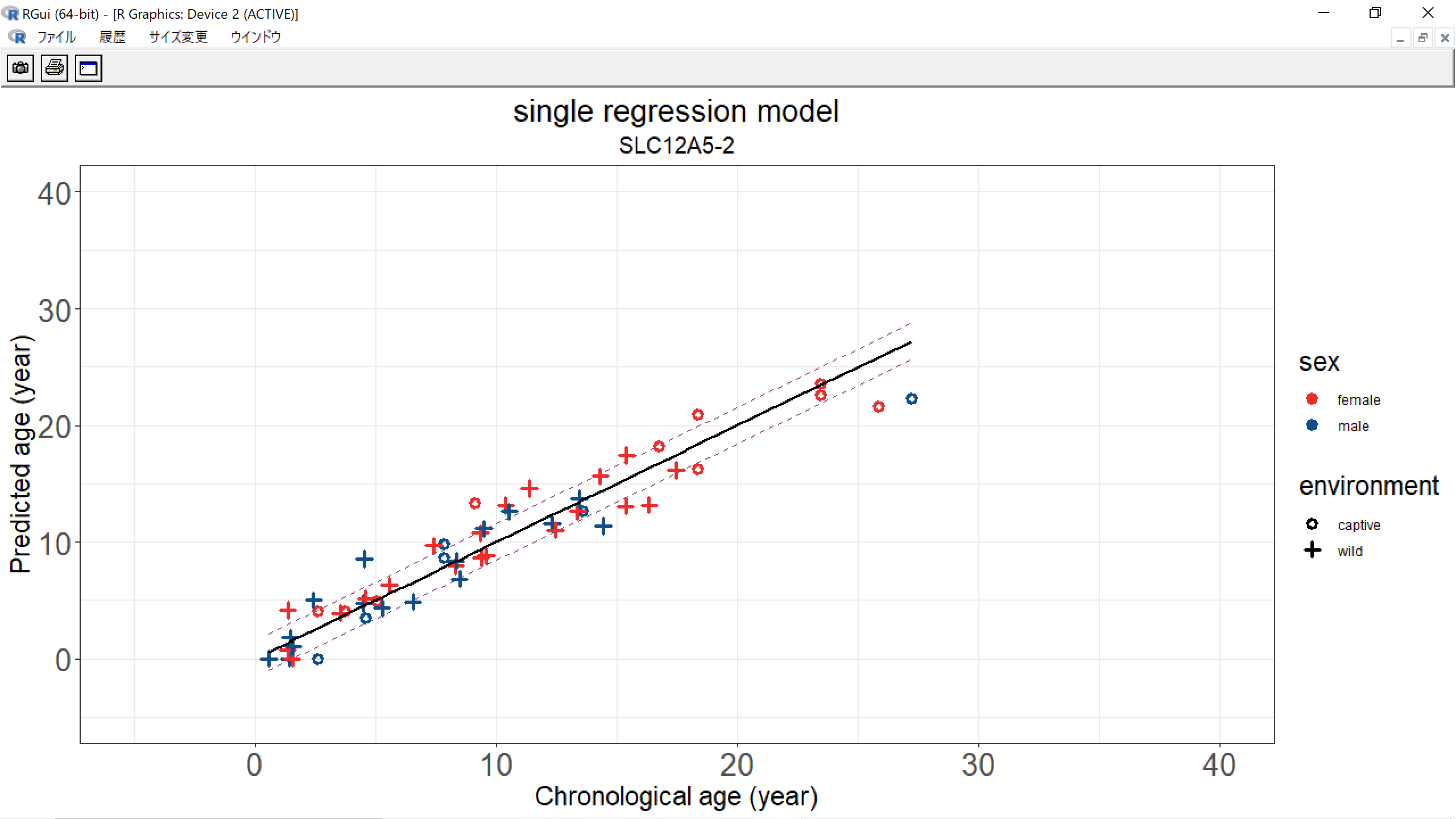


Single regression (SLC12A5-3)

SRM_SLC_3<-lm(formula=YS~slc3S,data=ABBBS)

coef(SRM_SLC_3)

(Intercept) slc3S

3.737342e-11 9.434067e-01

#LOOCV

nSamples<-nrow(ABBBS)

predict_SRM_SLC_3_loocv<-numeric(nSamples)

for (z in 1:nSamples){

indices<-removeOne(nSamples,z)

dr<-data.frame(ABBBS$age[indices],ABBBS$SLC12A5_3_methylation_rate_ave[indices])

colnames(dr)<-c("age","methylslc_3")

bestmodel_SRM_SLC_3_loocv<-lm(age~methylslc_3,data=dr)

newdata<-data.frame(methylslc_3=ABBBS$SLC12A5_3_methylation_rate_ave[z])

p<-predict(bestmodel_SRM_SLC_3_loocv,newdata)*sd(AGE)+mean(AGE)

if (p<0){p=0}

predict_SRM_SLC_3_loocv[z]<-p}

ABBB_predict_SRM_SLC_3_loocv<-cbind(ABBB,predict_SRM_SLC_3_loocv)

MAE_SRM_SLC_3_loocv<-mean(abs(ABBB_predict_SRM_SLC_3_loocv$predict_SRM_SLC_3_loocv-ABBB_predict_SRM_SLC_3_loocv$age))

MedianAE_SRM_SLC_3_loocv<-median(abs(ABBB_predict_SRM_SLC_3_loocv$predict_SRM_SLC_3_loocv-ABBB_predict_SRM_SLC_3_loocv$age))

RMSE_SRM_SLC_3_loocv<-sqrt(mean((ABBB_predict_SRM_SLC_3_loocv$predict_SRM_SLC_3_loocv-ABBB_predict_SRM_SLC_3_loocv$age)^2))

cat("MAE:", MAE_SRM_SLC_3_loocv, "\nMed AE:", MedianAE_SRM_SLC_3_loocv, "\nRMSE:", RMSE_SRM_SLC_3_loocv, "\n")

MAE: 1.768868

Med AE: 1.338683

RMSE: 2.340213

g_SRM_SLC_3_loocv<-ggplot(ABBB_predict_SRM_SLC_3_loocv,aes(age,predict_SRM_SLC_3_loocv))+theme_bw()+

annotate("segment",x=min(ABBB$age),xend=max(ABBB$age),y=min(ABBB$age)+1.768868,yend=max(ABBB$age)+1.768868,colour="orchid4",linetype=2, linewidth =0.7)+

annotate("segment",x=min(ABBB$age),xend=max(ABBB$age),y=min(ABBB$age)-1.768868,yend=max(ABBB$age)-1.768868,colour="orchid4",linetype=2, linewidth =0.7)+

geom_point(aes(shape=environment,color=sex),size=2,stroke=2)+

labs(x="Chronological age (year)",y="Predicted age (year)")+

scale_shape_manual(name="environment",labels=c("Captive"="captive","Wild"="wild"),values=c("Captive"=1, "Wild"=3))+

scale_color_manual(name="sex",labels=c("F"="female","M"="male"),values=c("F"="firebrick2","M"="dodgerblue4"))+

theme(axis.text.x=element_text(size=20),axis.text.y=element_text(size=20))+

theme(axis.title.x=element_text(size=17),axis.title.y=element_text(size=17))+

geom_line(aes(y =age), linewidth=1)+

labs(title="single regression model")+

theme(title=element_text(size=17),plot.title=element_text(hjust=0.5))+

scale_y_continuous(limits=c(-5,40))+

scale_x_continuous(limits=c(-5,40))+

labs(subtitle="SLC12A5-3")+

theme(plot.subtitle=element_text(size=15,hjust=0.5))


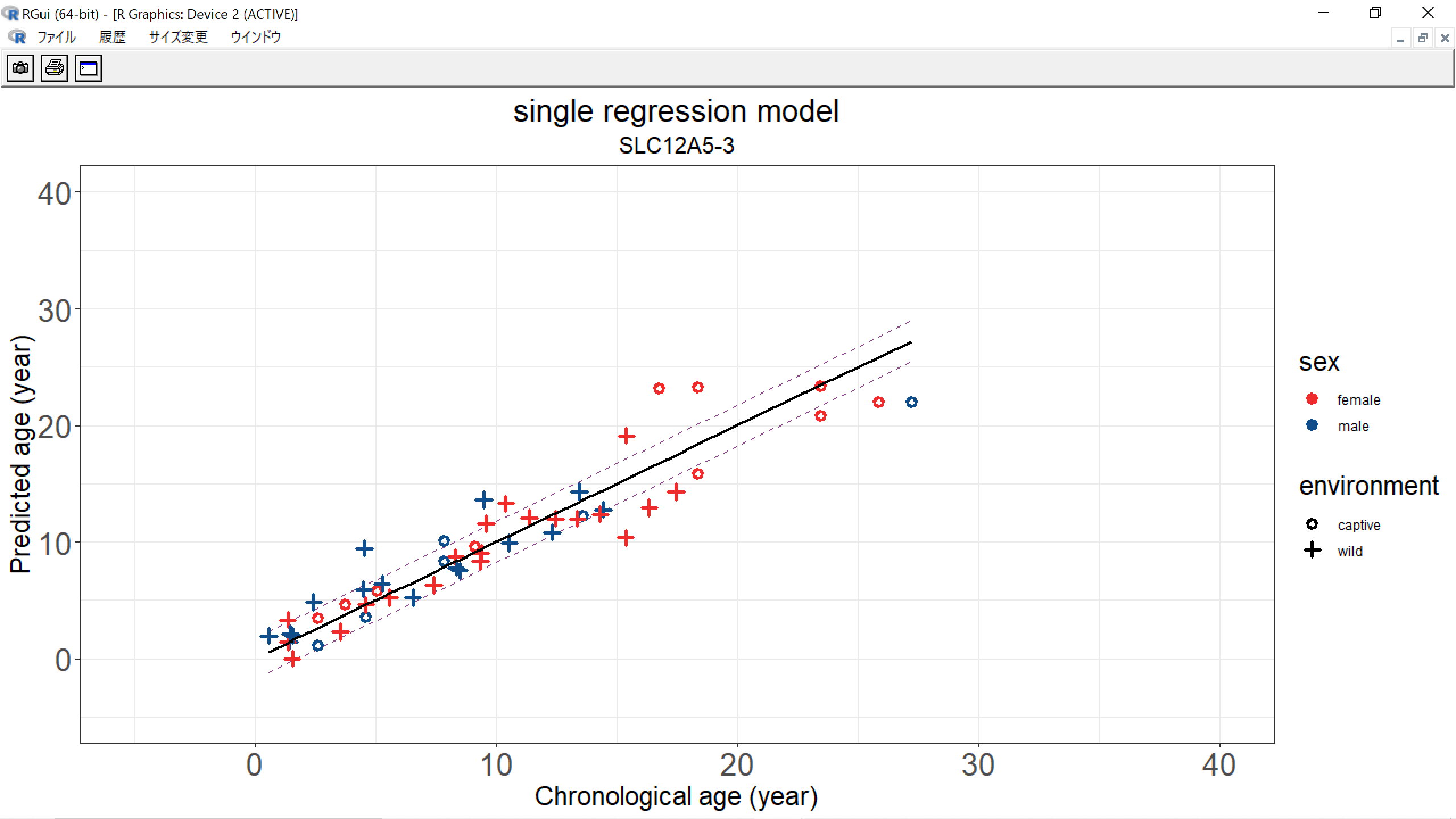


Single regression (SLC12A5-4)

SRM_SLC_4<-lm(formula=YS~slc4S,data=ABBBS)

coef(SRM_SLC_4)

(Intercept) slc4S

1.923086e-11 9.670194e-01

#LOOCV

nSamples<-nrow(ABBBS)

predict_SRM_SLC_4_loocv<-numeric(nSamples)

for (z in 1:nSamples){

indices<-removeOne(nSamples,z)

dr<-data.frame(ABBBS$age[indices],ABBBS$SLC12A5_4_methylation_rate_ave[indices])

colnames(dr)<-c("age","methylslc_4")

bestmodel_SRM_SLC_4_loocv<-lm(age~methylslc_4,data=dr)

newdata<-data.frame(methylslc_4=ABBBS$SLC12A5_4_methylation_rate_ave[z])

p<-predict(bestmodel_SRM_SLC_4_loocv,newdata)*sd(AGE)+mean(AGE)

if (p<0){p=0}

predict_SRM_SLC_4_loocv[z]<-p}

ABBB_predict_SRM_SLC_4_loocv<-cbind(ABBB,predict_SRM_SLC_4_loocv)

MAE_SRM_SLC_4_loocv<-mean(abs(ABBB_predict_SRM_SLC_4_loocv$predict_SRM_SLC_4_loocv-ABBB_predict_SRM_SLC_4_loocv$age))

MedianAE_SRM_SLC_4_loocv<-median(abs(ABBB_predict_SRM_SLC_4_loocv$predict_SRM_SLC_4_loocv-ABBB_predict_SRM_SLC_4_loocv$age))

RMSE_SRM_SLC_4_loocv<-sqrt(mean((ABBB_predict_SRM_SLC_4_loocv$predict_SRM_SLC_4_loocv-ABBB_predict_SRM_SLC_4_loocv$age)^2))

cat("MAE:", MAE_SRM_SLC_4_loocv, "\nMed AE:", MedianAE_SRM_SLC_4_loocv, "\nRMSE:", RMSE_SRM_SLC_4_loocv, "\n")

MAE: 1.405935

Med AE: 1.315636

RMSE: 1.746293

g_SRM_SLC_4_loocv<-ggplot(ABBB_predict_SRM_SLC_4_loocv,aes(age,predict_SRM_SLC_4_loocv))+theme_bw()+

annotate("segment",x=min(ABBB$age),xend=max(ABBB$age),y=min(ABBB$age)+1.405935,yend=max(ABBB$age)+1.405935,colour="orchid4",linetype=2, linewidth =0.7)+

annotate("segment",x=min(ABBB$age),xend=max(ABBB$age),y=min(ABBB$age)-1.405935,yend=max(ABBB$age)-1.405935,colour="orchid4",linetype=2, linewidth =0.7)+

geom_point(aes(shape=environment,color=sex),size=2,stroke=2)+

labs(x="Chronological age (year)",y="Predicted age (year)")+

scale_shape_manual(name="environment",labels=c("Captive"="captive","Wild"="wild"),values=c("Captive"=1, "Wild"=3))+

scale_color_manual(name="sex",labels=c("F"="female","M"="male"),values=c("F"="firebrick2","M"="dodgerblue4"))+

theme(axis.text.x=element_text(size=20),axis.text.y=element_text(size=20))+

theme(axis.title.x=element_text(size=17),axis.title.y=element_text(size=17))+

geom_line(aes(y =age), linewidth=1)+

labs(title="single regression model")+

theme(title=element_text(size=17),plot.title=element_text(hjust=0.5))+

scale_y_continuous(limits=c(-5,40))+

scale_x_continuous(limits=c(-5,40))+

labs(subtitle="SLC12A5-4")+

theme(plot.subtitle=element_text(size=15,hjust=0.5))


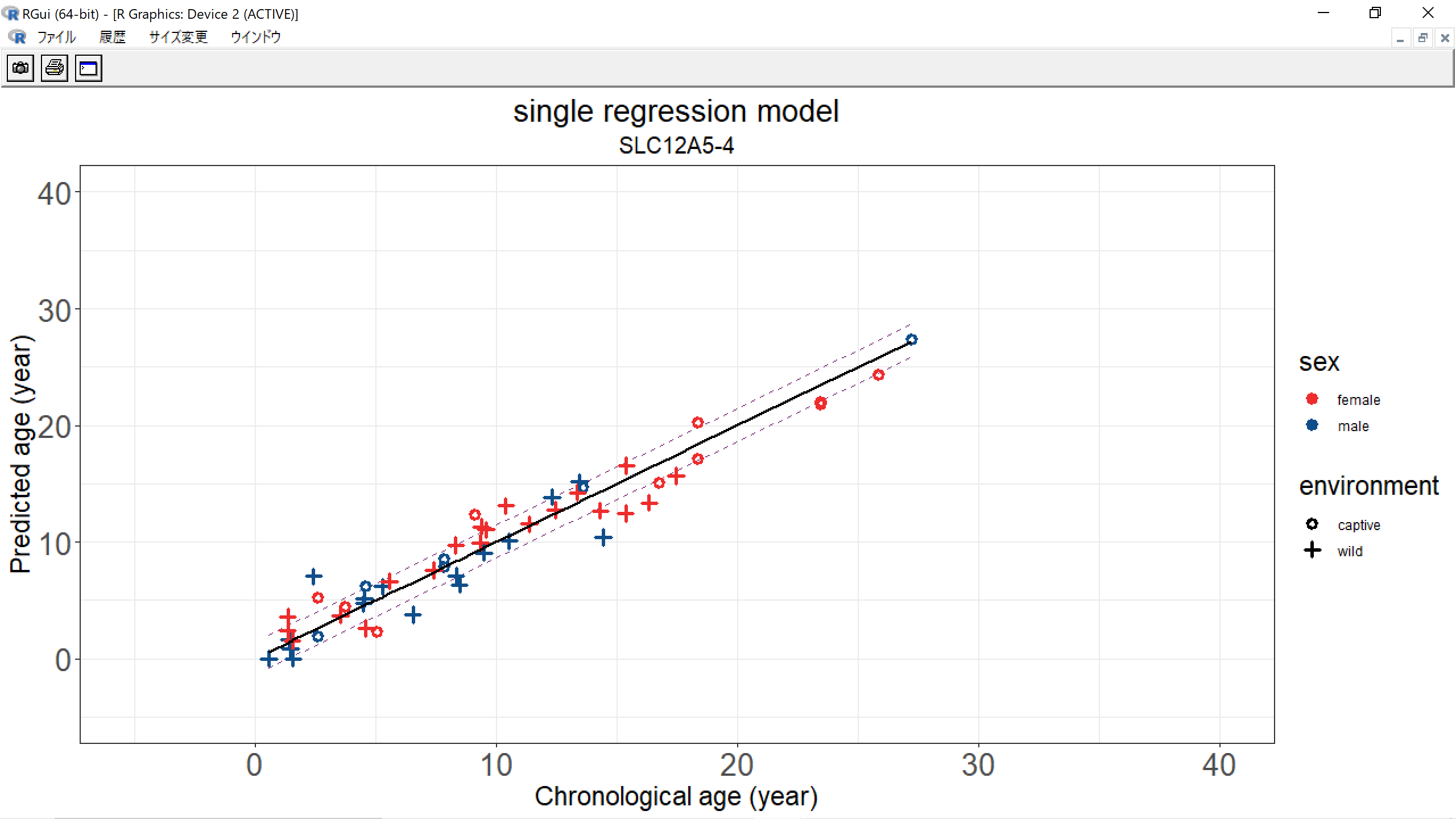


Age estimation model 【Principal component regression】

AGE <- ABBB$age

#RemoveOne function

removeOne <- function(dat,x) {

if(x<dat){

list=seq(1,dat)

x1=x-1;x2=x+1

v1=c(list[0:x1]);v2=c(list[x2:dat])

data=c(v1,v2)}

else {data=seq(1,dat-1)}

return (data)}

pca_slc <- prcomp(ABBBS[, c("SLC12A5_1_methylation_rate_ave",

"SLC12A5_2_methylation_rate_ave",

"SLC12A5_3_methylation_rate_ave",

"SLC12A5_4_methylation_rate_ave")],

scale = FALSE)

pca_slc

Standard deviations (1, .., p=4):

[1] 1.9649387 0.2452374 0.2287991 0.1628668

Rotation (n x k) = (4 x 4):

PC1 PC2 PC3

SLC12A5_1_methylation_rate_ave -0.4991565 0.06588438 -0.83362958

SLC12A5_2_methylation_rate_ave -0.5036685 -0.13925681 0.05888886

SLC12A5_3_methylation_rate_ave -0.4991723 -0.65943050 0.36355737

SLC12A5_4_methylation_rate_ave -0.4979838 0.73581126 0.41160645

PC4

SLC12A5_1_methylation_rate_ave -0.2270767

SLC12A5_2_methylation_rate_ave 0.8505631

SLC12A5_3_methylation_rate_ave -0.4287242

SLC12A5_4_methylation_rate_ave -0.2029139

screeplot(pca_slc, type = "lines", main = "Scree plot")


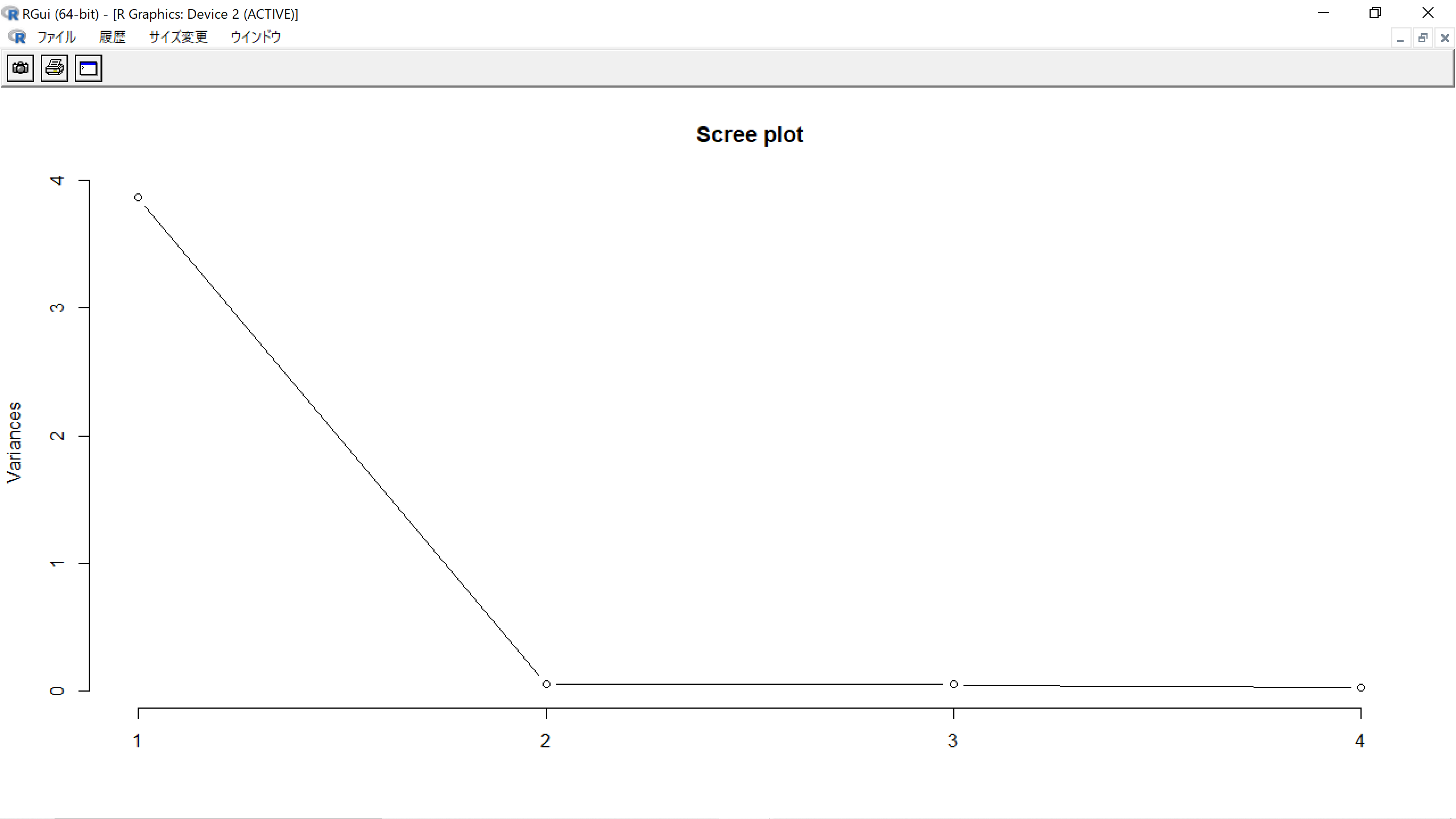


summary(pca_slc)

Importance of components:

PC1 PC2 PC3 PC4

Standard deviation 1.9649 0.24524 0.22880 0.16287

Proportion of Variance 0.9653 0.01504 0.01309 0.00663

Cumulative Proportion 0.9653 0.98028 0.99337 1.00000

Principal component regression (PC1)

ABBBS$SLC12A5_PC1 <- pca_slc$x[,1]

PCRM_SLC <- lm(formula = age ~ SLC12A5_PC1, data = ABBBS)

coef(PCRM_SLC)

(Intercept) SLC12A5_PC1

1.923074e-11 -4.936109e-01

#LOOCV

nSamples <- nrow(ABBBS)

predict_PCRM_SLC_loocv <- numeric(nSamples)

for (z in 1:nSamples) {

indices <- removeOne(nSamples, z)

dr <- data.frame(ABBBS$age[indices], ABBBS$SLC12A5_PC1[indices])

colnames(dr) <- c("age", "methylslc_pc1")

bestmodel_PCRM_SLC_loocv <- lm(age ~ methylslc_pc1, data = dr)

newdata <- data.frame(methylslc_pc1 = ABBBS$SLC12A5_PC1[z])

p <- predict(bestmodel_PCRM_SLC_loocv, newdata) * sd(AGE) + mean(AGE)

if (p < 0) { p = 0 }

predict_PCRM_SLC_loocv[z] <- p

}

ABBB_predict_PCRM_SLC_loocv <- cbind(ABBB, predict_PCRM_SLC_loocv)

MAE_PCRM_SLC_loocv <- mean(abs(ABBB_predict_PCRM_SLC_loocv$predict_PCRM_SLC_loocv - ABBB_predict_PCRM_SLC_loocv$age))

MedianAE_PCRM_SLC_loocv <- median(abs(ABBB_predict_PCRM_SLC_loocv$predict_PCRM_SLC_loocv - ABBB_predict_PCRM_SLC_loocv$age))

RMSE_PCRM_SLC_loocv <- sqrt(mean((ABBB_predict_PCRM_SLC_loocv$predict_PCRM_SLC_loocv - ABBB_predict_PCRM_SLC_loocv$age)^2))

cat("MAE:", MAE_PCRM_SLC_loocv, "\nMed AE:", MedianAE_PCRM_SLC_loocv, "\nRMSE:", RMSE_PCRM_SLC_loocv, "\n")

MAE: 1.33657

Med AE: 1.166756

RMSE: 1.698863

g_PCRM_SLC_loocv<-ggplot(ABBB_predict_PCRM_SLC_loocv,aes(age,predict_PCRM_SLC_loocv))+theme_bw()+

annotate("segment",x=min(ABBB$age),xend=max(ABBB$age),y=min(ABBB$age)+1.33657,yend=max(ABBB$age)+1.33657,colour="orchid4",linetype=2, linewidth =0.7)+

annotate("segment",x=min(ABBB$age),xend=max(ABBB$age),y=min(ABBB$age)-1.33657

,yend=max(ABBB$age)-1.33657,colour="orchid4",linetype=2, linewidth =0.7)+

geom_point(aes(shape=environment,color=sex),size=2,stroke=2)+

labs(x="Chronological age (year)",y="Predicted age (year)")+

scale_shape_manual(name="environment",labels=c("Captive"="captive","Wild"="wild"),values=c("Captive"=1, "Wild"=3))+

scale_color_manual(name="sex",labels=c("F"="female","M"="male"),values=c("F"="firebrick2","M"="dodgerblue4"))+

theme(axis.text.x=element_text(size=20),axis.text.y=element_text(size=20))+

theme(axis.title.x=element_text(size=17),axis.title.y=element_text(size=17))+

geom_line(aes(y =age), linewidth=1)+

labs(title="principal component regression model")+

theme(title=element_text(size=17),plot.title=element_text(hjust=0.5))+

scale_y_continuous(limits=c(-5,40))+

scale_x_continuous(limits=c(-5,40))+

labs(subtitle="SLC12A5-PC1")+

theme(plot.subtitle=element_text(size=15,hjust=0.5))


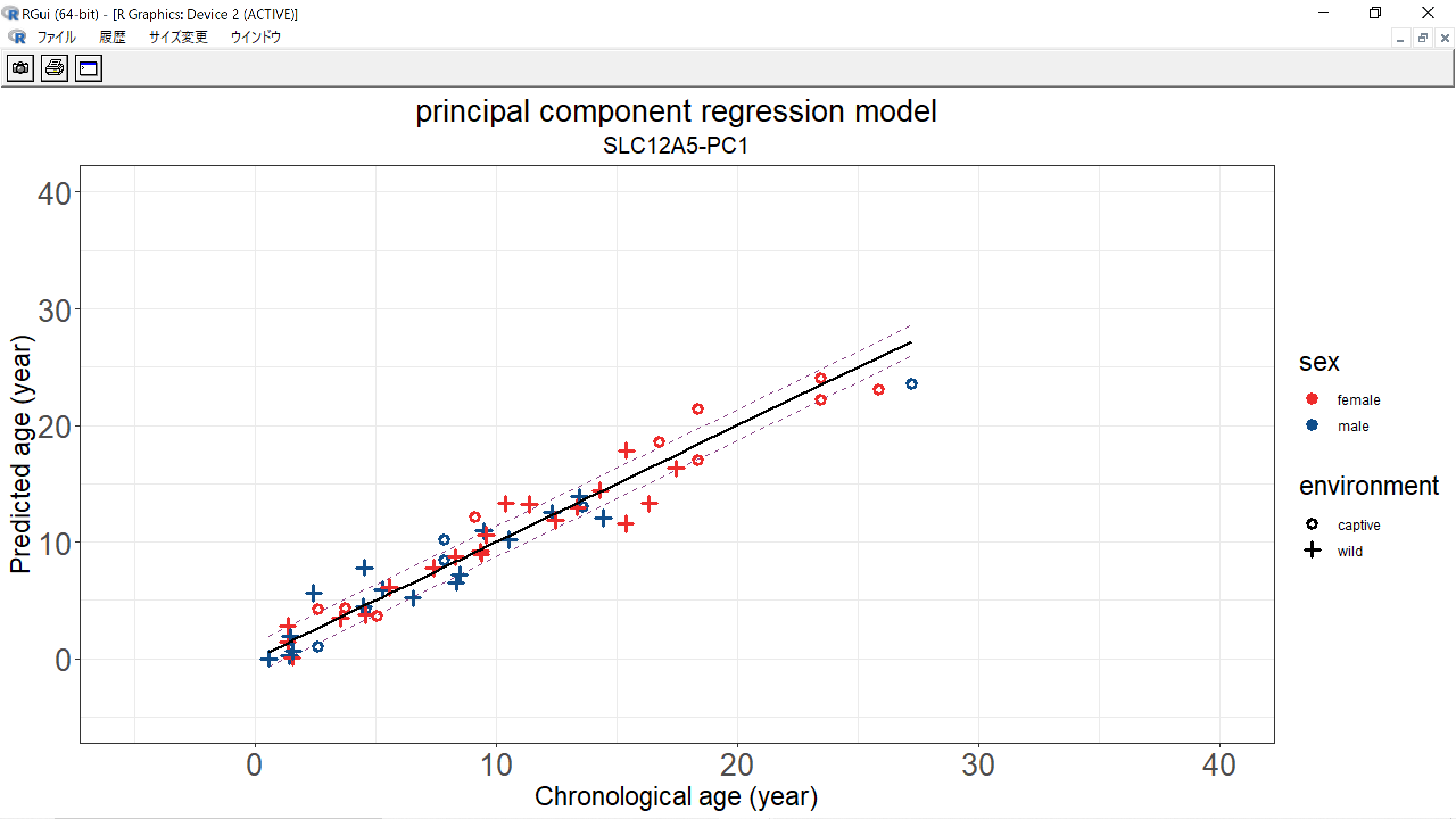


Age estimation model 【Elastic net regression】

YS <- ABBBS$age

slc1S <- ABBBS$SLC12A5_1_methylation_rate_ave

slc2S <- ABBBS$SLC12A5_2_methylation_rate_ave

slc3S <- ABBBS$SLC12A5_3_methylation_rate_ave

slc4S <- ABBBS$SLC12A5_4_methylation_rate_ave

AGE <- ABBB$age

#RemoveOne function

removeOne <- function(dat,x) {

if(x<dat){

list=seq(1,dat)

x1=x-1;x2=x+1

v1=c(list[0:x1]);v2=c(list[x2:dat])

data=c(v1,v2)}

else {data=seq(1,dat-1)}

return (data)}

Elastic net regression (SLC12A5-1, -2, -3, -4)

XS <- cbind(slc1S, slc2S, slc3S, slc4S)

set.seed(1)

alpha <- seq(0.01, 0.99, 0.01)

repeats <- 20

alpha.cvm.df <- NULL

for (i in 1:length(alpha)) {

alpha.repeats <- numeric(repeats)

for (j in 1:repeats) {

set.seed(j)

m <- cv.glmnet(x = XS, y = YS, family = "gaussian", alpha = alpha[i], standardize = FALSE)

alpha.repeats[j] <- min(m$cvm)

}

alpha.cvm.df <- rbind(alpha.cvm.df, data.frame(alpha = alpha[i], mincvm = mean(alpha.repeats)))

}

best.alpha <- alpha.cvm.df$alpha[which.min(alpha.cvm.df$mincvm)]

lambda.cvm.df <- NULL

lambda.list <- numeric(repeats)

cv.errors <- numeric(repeats)

for (i in 1:repeats) {

set.seed(i)

m <- cv.glmnet(x = XS, y = YS, family = "gaussian", alpha = best.alpha, standardize = FALSE)

lambda.cvm.df <- rbind(lambda.cvm.df, data.frame(lambda = m$lambda.min, mincvm = min(m$cvm)))

}

lambda_counts <- lambda.cvm.df %>%

group_by(lambda) %>%

summarise(count = n(), .groups = 'drop')

max_count <- max(lambda_counts$count)

candidates <- lambda_counts$lambda[lambda_counts$count == max_count]

best.lambda <- lambda.cvm.df %>%

filter(lambda %in% candidates) %>%

slice(which.min(mincvm)) %>%

pull(lambda)

cat("Best alpha:", best.alpha, "\nBest lambda:", best.lambda, "\n")

Best alpha: 0.01

Best lambda: 0.009484228

ENM1 <- glmnet(x = XS, y = YS, family = "gaussian", lambda = best.lambda, alpha = best.alpha, standardize = FALSE)

coef(ENM1,s=best.lambda)

5 x 1 sparse Matrix of class "dgCMatrix"

s1

(Intercept) -1.877875e-11

slc1S 1.047886e-01

slc2S 2.288183e-01

slc3S 1.385184e-01

slc4S 5.131129e-01

#LOOCV

nSamples<-nrow(ABBBS)

predict_ENM_loocv<-numeric(nSamples)

for (z in 1:nSamples){

indices<-removeOne(nSamples,z)

X1 <-cbind(ABBBS$SLC12A5_1_methylation_rate_ave[indices],ABBBS$SLC12A5_2_methylation_rate_ave[indices],ABBBS$SLC12A5_3_methylation_rate_ave[indices],ABBBS$SLC12A5_4_methylation_rate_ave[indices])

Y1 <- ABBBS$age[indices]

ENM2 <- glmnet(x = X1, y = Y1, family = "gaussian", lambda = best.lambda, alpha = best.alpha, standardize = FALSE)

Xnew <- cbind(ABBBS$SLC12A5_1_methylation_rate_ave[z],ABBBS$SLC12A5_2_methylation_rate_ave[z],ABBBS$SLC12A5_3_methylation_rate_ave[z],ABBBS$SLC12A5_4_methylation_rate_ave[z])

p<-predict(ENM2,Xnew,s=best.lambda) *sd(AGE)+mean(AGE)

if (p<0){p=0}

predict_ENM_loocv[z]<-p}

ABBB_predict_ENM_loocv<-cbind(ABBB,predict_ENM_loocv)

MAE_ENM_loocv<-mean(abs(ABBB_predict_ENM_loocv$predict_ENM_loocv-ABBB_predict_ENM_loocv$age))

MedianAE_ENM_loocv<-median(abs(ABBB_predict_ENM_loocv$predict_ENM_loocv-ABBB_predict_ENM_loocv$age))

RMSE_ENM_loocv<- sqrt(mean((ABBB_predict_ENM_loocv$predict_ENM_loocv-ABBB_predict_ENM_loocv$age)^2))

cat("MAE:", MAE_ENM_loocv, "\nMed AE:", MedianAE_ENM_loocv, "\nRMSE:", RMSE_ENM_loocv, "\n")

MAE: 1.307885

Med AE: 1.077722

RMSE: 1.647679

g_ENM_loocv<-ggplot(ABBB_predict_ENM_loocv,aes(age,predict_ENM_loocv))+theme_bw()+

annotate("segment",x=min(ABBB$age),xend=max(ABBB$age),y=min(ABBB$age)+1.307885,yend=max(ABBB$age)+1.307885,colour="orchid4",linetype=2,linewidth =0.7)+

annotate("segment",x=min(ABBB$age),xend=max(ABBB$age),y=min(ABBB$age)-1.307885,yend=max(ABBB$age)-1.307885,colour="orchid4",linetype=2,linewidth =0.7)+

geom_point(aes(shape=environment,color=sex),size=2,stroke=2)+

labs(x="Chronological age (year)",y="Predicted age (year)")+

scale_shape_manual(name="environment",labels=c("Captive"="captive","Wild"="wild"),values=c("Captive"=1, "Wild"=3))+

scale_color_manual(name="sex",labels=c("F"="female","M"="male"),values=c("F"="firebrick2","M"="dodgerblue4"))+

theme(axis.text.x=element_text(size=20),axis.text.y=element_text(size=20))+

theme(axis.title.x=element_text(size=17),axis.title.y=element_text(size=17))+

geom_line(aes(y =age), linewidth=1)+

labs(title="elastic net regression model")+

theme(title=element_text(size=17),plot.title=element_text(hjust=0.5))+

scale_y_continuous(limits=c(-5,40))+

scale_x_continuous(limits=c(-5,40))+

labs(subtitle="SLC12A5-1, -2, -3, -4")+

theme(plot.subtitle=element_text(size=15,hjust=0.5))


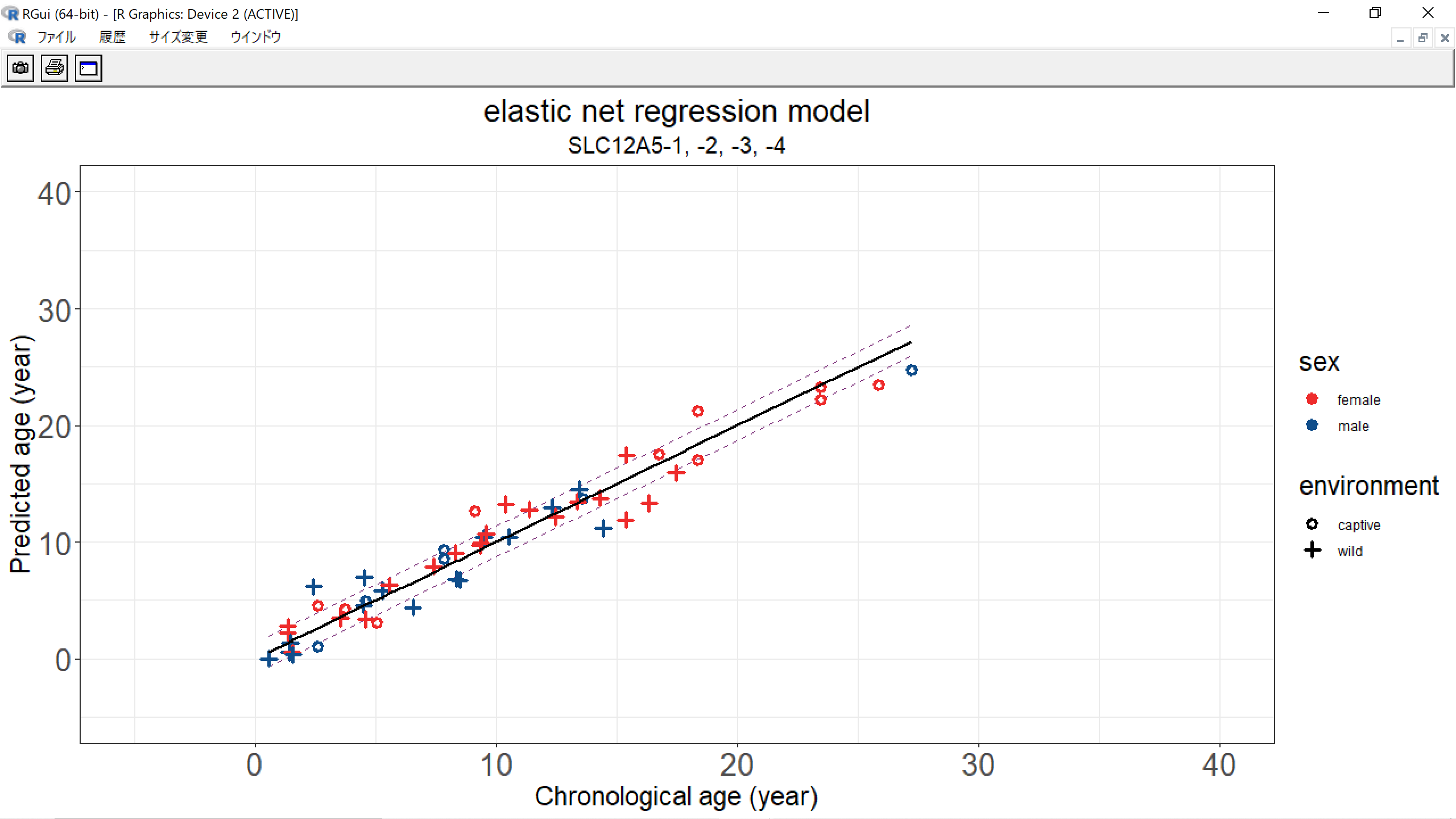


Age estimation model 【Support vector regression】

AGE <- ABBB$age

#RemoveOne function

removeOne <- function(dat,x) {

if(x<dat){

list=seq(1,dat)

x1=x-1;x2=x+1

v1=c(list[0:x1]);v2=c(list[x2:dat])

data=c(v1,v2)}

else {data=seq(1,dat-1)}

return (data)}

Support vector regression (SLC12A5-1, -2, -3, -4)

set.seed(1)

tuneResult<-

tune(svm,age~SLC12A5_1_methylation_rate_ave+SLC12A5_2_methylation_rate_ave+SLC12A5_3_methylation_rate_ave+SLC12A5_4_methylation_rate_ave,data=ABBBS,

ranges=list(cost=10^(seq(-4,5,0.1)),gamma=10^(seq(-5,4,0.1))),

tunecontrol = tune.control(sampling = "cross", cross = 10), scale = FALSE)

tunedModel <- tuneResult$best.model

tunedModel

Call:

best.tune(METHOD = svm, train.x = age ~ SLC12A5_1_methylation_rate_ave +

SLC12A5_2_methylation_rate_ave + SLC12A5_3_methylation_rate_ave +

SLC12A5_4_methylation_rate_ave, data = ABBBS, ranges = list(cost = 10^(seq(-4,

5, 0.1)), gamma = 10^(seq(-5, 4, 0.1))), tunecontrol = tune.control(sampling = "cross",

cross = 10), scale = FALSE)

Parameters:

SVM-Type: eps-regression

SVM-Kernel: radial

cost: 1e+05

gamma: 0.0002511886

epsilon: 0.1

Number of Support Vectors: 28

best.cost <- tunedModel$cost

best.gamma <- tunedModel$gamma

cat("Cost: ", best.cost, "\nGamma: ", best.gamma, "\n")

Cost: 1e+05

Gamma: 0.0002511886

tune_results <- as.data.frame(tuneResult$performances)

tune_results$cost <- log10(tune_results$cost)

tune_results$gamma <- log10(tune_results$gamma)

ggplot(tune_results, aes(x = cost, y = gamma, fill = error)) +

geom_tile() +

geom_point(aes(x = log10(best.cost), y = log10(best.gamma)), color = "blue", size = 1, shape = 21, fill = "blue") +

scale_fill_gradient(low = "white", high = "red") +

labs(title = "SVM Parameter Tuning Results",

x = "Log10(Cost)",

y = "Log10(Gamma)",

fill = "Error") +

theme_minimal()


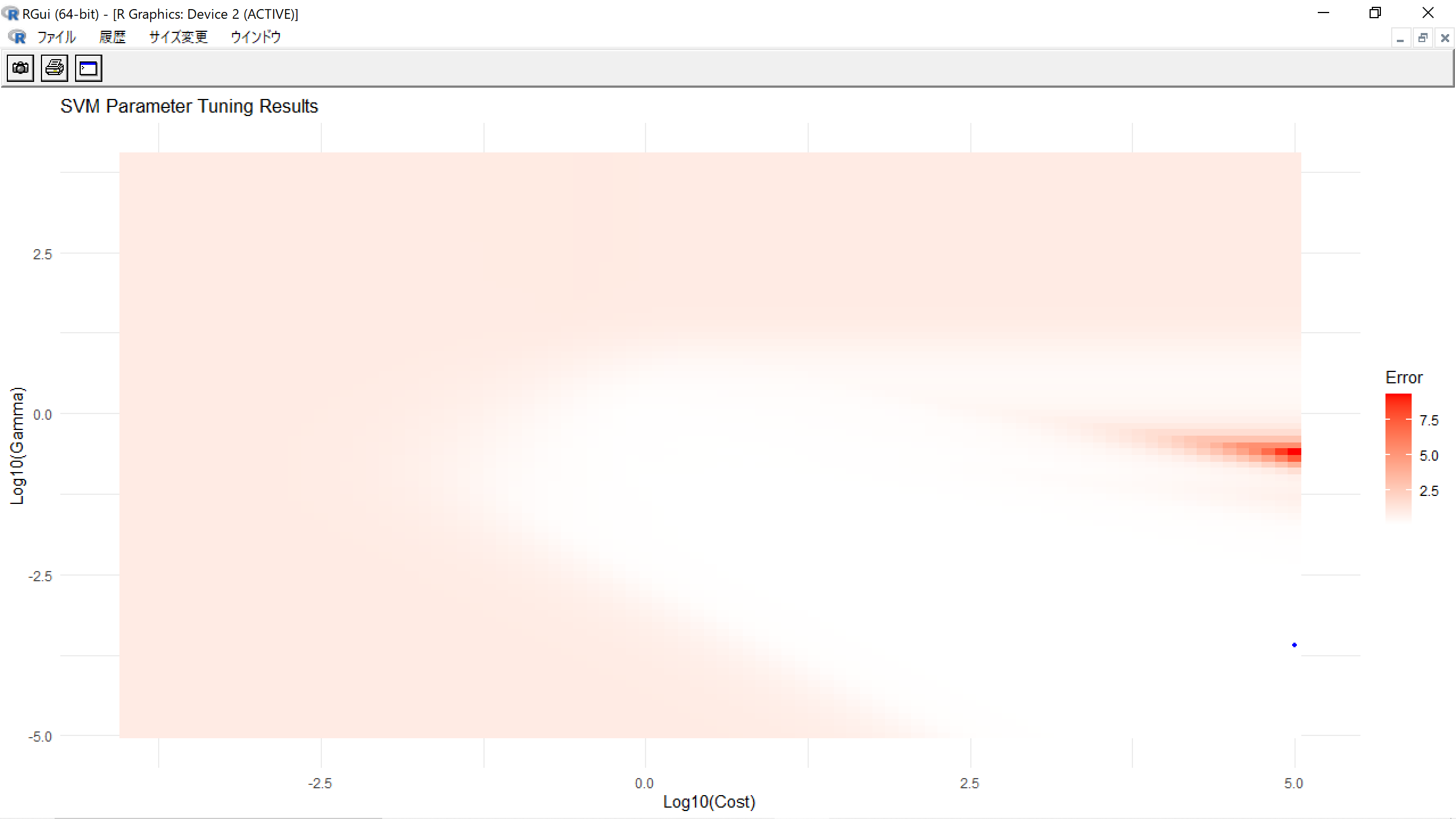


SVRM<-

svm(age~SLC12A5_1_methylation_rate_ave+SLC12A5_2_methylation_rate_ave+SLC12A5_3_methylation_rate_ave+SLC12A5_4_methylation_rate_ave,　data=ABBBS,

cost=best.cost, gamma=best.gamma, epsilon=0.1, scale = FALSE)

#LOOCV

nSamples<-nrow(ABBBS)

predict_SVRM_loocv<-numeric(nSamples)

for (z in 1:nSamples){

indices<-removeOne(nSamples,z)

dr<-data.frame(ABBBS$age[indices],ABBBS$SLC12A5_1_methylation_rate_ave[indices],ABBBS$SLC12A5_2_methylation_rate_ave[indices],ABBBS$SLC12A5_3_methylation_rate_ave[indices],ABBBS$SLC12A5_4_methylation_rate_ave[indices])

colnames(dr)<-c("age","methylslc_1","methylslc_2","methylslc_3","methylslc_4")

bestmodel_SVRM<-svm(age~methylslc_1+methylslc_2+methylslc_3+methylslc_4, data=dr,

cost=best.cost, gamma= best.gamma, epsilon=0.1, scale = FALSE)

newdata<-data.frame(methylslc_1=ABBBS$SLC12A5_1_methylation_rate_ave[z],methylslc_2=ABBBS$SLC12A5_2_methylation_rate_ave[z],methylslc_3=ABBBS$SLC12A5_3_methylation_rate_ave[z],methylslc_4=ABBBS$SLC12A5_4_methylation_rate_ave[z])

p<-predict(bestmodel_SVRM,newdata)*sd(AGE)+mean(AGE)

if (p<0){p=0}

predict_SVRM_loocv[z]<-p}

ABBB_SVRM_loocv<-cbind(ABBB,predict_SVRM_loocv)

MAE_SVRM_loocv<-mean(abs(ABBB_SVRM_loocv$predict_SVRM_loocv-ABBB$age))

MedianAE_SVRM_loocv<-median(abs(ABBB_SVRM_loocv$predict_SVRM_loocv-ABBB$age))

RMSE_SVRM_loocv<- sqrt(mean((ABBB_SVRM_loocv$predict_SVRM_loocv-ABBB$age)^2))

cat("MAE:", MAE_SVRM_loocv, "\nMed AE:", MedianAE_SVRM_loocv, "\nRMSE:", RMSE_SVRM_loocv, "\n")

MAE: 1.162576

Med AE: 0.8189912

RMSE: 1.556881

Support vector regression (SLC12A5-1, -2, -3)

set.seed(1)

tuneResult<-

tune(svm,age~SLC12A5_1_methylation_rate_ave+SLC12A5_2_methylation_rate_ave+SLC12A5_3_methylation_rate_ave,data=ABBBS,

ranges=list(cost=10^(seq(-4,5,0.1)),gamma=10^(seq(-5,4,0.1))),

tunecontrol = tune.control(sampling = "cross", cross = 10), scale = FALSE)

tunedModel <- tuneResult$best.model

tunedModel

Call:

best.tune(METHOD = svm, train.x = age ~ SLC12A5_1_methylation_rate_ave +

SLC12A5_2_methylation_rate_ave + SLC12A5_3_methylation_rate_ave,

data = ABBBS, ranges = list(cost = 10^(seq(-4, 5, 0.1)), gamma = 10^(seq(-5,

4, 0.1))), tunecontrol = tune.control(sampling = "cross",

cross = 10), scale = FALSE)

Parameters:

SVM-Type: eps-regression

SVM-Kernel: radial

cost: 2.511886

gamma: 0.1584893

epsilon: 0.1

Number of Support Vectors: 36

best.cost <- tunedModel$cost

best.gamma <- tunedModel$gamma

cat("Cost: ", best.cost, "\nGamma: ", best.gamma, "\n")

Cost: 2.511886

Gamma: 0.1584893

tune_results <- as.data.frame(tuneResult$performances)

tune_results$cost <- log10(tune_results$cost)

tune_results$gamma <- log10(tune_results$gamma)

ggplot(tune_results, aes(x = cost, y = gamma, fill = error)) +

geom_tile() +

geom_point(aes(x = log10(best.cost), y = log10(best.gamma)), color = "blue", size = 1, shape = 21, fill = "blue") +

scale_fill_gradient(low = "white", high = "red") +

labs(title = "SVM Parameter Tuning Results",

x = "Log10(Cost)",

y = "Log10(Gamma)",

fill = "Error") +

theme_minimal()


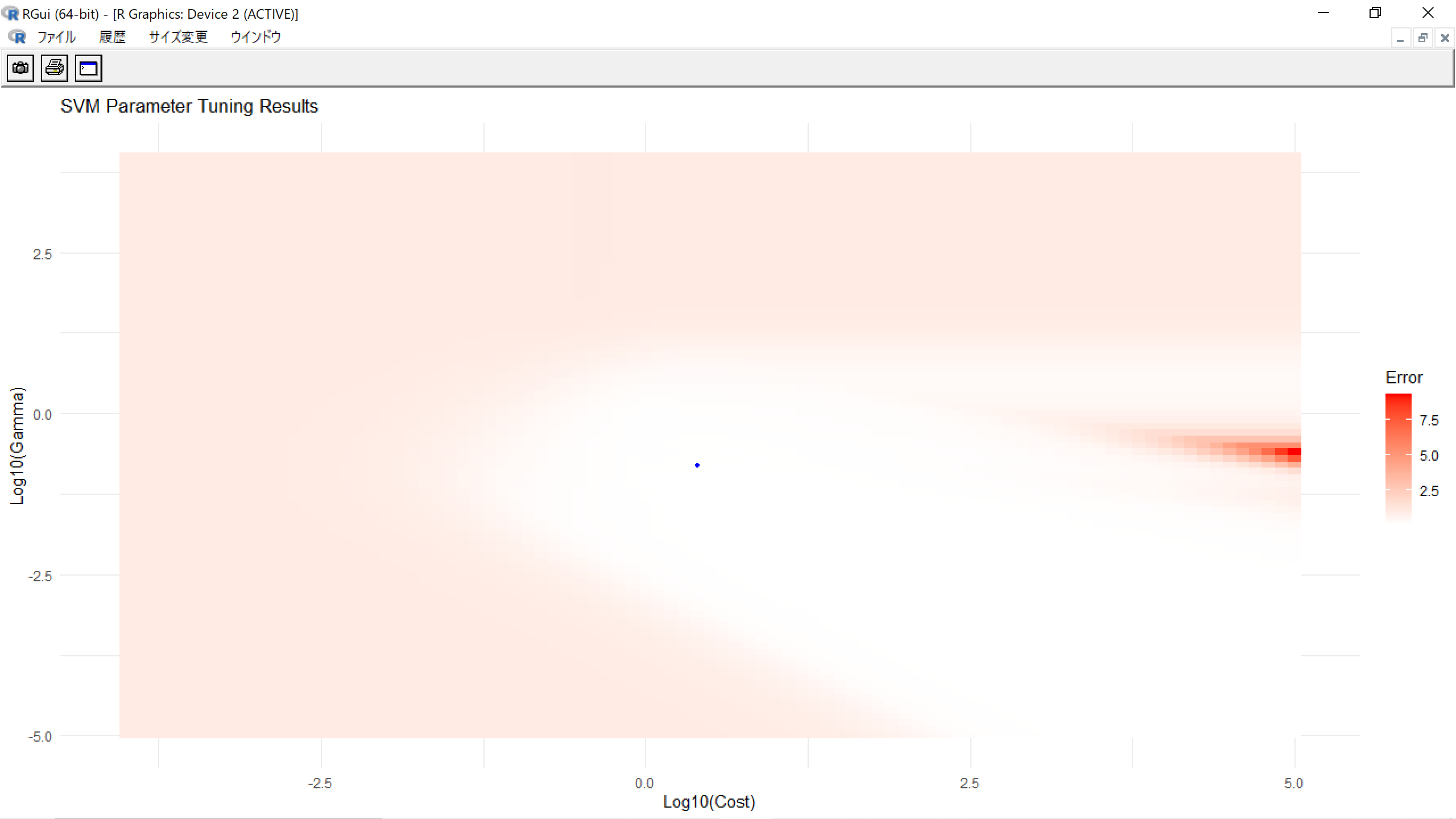


SVRM<-

svm(age~SLC12A5_1_methylation_rate_ave+SLC12A5_2_methylation_rate_ave+SLC12A5_3_methylation_rate_ave,　data=ABBBS,

cost=best.cost, gamma=best.gamma, epsilon=0.1, scale = FALSE)

#LOOCV

nSamples<-nrow(ABBBS)

predict_SVRM_loocv<-numeric(nSamples)

for (z in 1:nSamples){

indices<-removeOne(nSamples,z)

dr<-data.frame(ABBBS$age[indices], ABBBS$SLC12A5_1_methylation_rate_ave[indices],ABBBS$SLC12A5_2_methylation_rate_ave[indices],ABBBS$SLC12A5_3_methylation_rate_ave[indices])

colnames(dr)<-c("age","methylslc_1","methylslc_2","methylslc_3")

bestmodel_SVRM<-svm(age~ methylslc_1+methylslc_2+methylslc_3, data=dr,

cost=best.cost, gamma= best.gamma, epsilon=0.1, scale = FALSE)

newdata<-data.frame(methylslc_1=ABBBS$SLC12A5_1_methylation_rate_ave[z],methylslc_2=ABBBS$SLC12A5_2_methylation_rate_ave[z],methylslc_3=ABBBS$SLC12A5_3_methylation_rate_ave[z])

p<-predict(bestmodel_SVRM,newdata)*sd(AGE)+mean(AGE)

if (p<0){p=0}

predict_SVRM_loocv[z]<-p}

ABBB_SVRM_loocv<-cbind(ABBB,predict_SVRM_loocv)

MAE_SVRM_loocv<-mean(abs(ABBB_SVRM_loocv$predict_SVRM_loocv-ABBB$age))

MedianAE_SVRM_loocv<-median(abs(ABBB_SVRM_loocv$predict_SVRM_loocv-ABBB$age))

RMSE_SVRM_loocv<- sqrt(mean((ABBB_SVRM_loocv$predict_SVRM_loocv-ABBB$age)^2))

cat("MAE:", MAE_SVRM_loocv, "\nMed AE:", MedianAE_SVRM_loocv, "\nRMSE:", RMSE_SVRM_loocv, "\n")

MAE: 1.43753

Med AE: 1.104879

RMSE: 1.888627

Support vector regression (SLC12A5-1, -2, -4)

set.seed(1)

tuneResult<-

tune(svm,age~SLC12A5_1_methylation_rate_ave+SLC12A5_2_methylation_rate_ave+SLC12A5_4_methylation_rate_ave,data=ABBBS,

ranges=list(cost=10^(seq(-4,5,0.1)),gamma=10^(seq(-5,4,0.1))),

tunecontrol = tune.control(sampling = "cross", cross = 10), scale = FALSE)

tunedModel <- tuneResult$best.model

tunedModel

Call:

best.tune(METHOD = svm, train.x = age ~ SLC12A5_1_methylation_rate_ave +

SLC12A5_2_methylation_rate_ave + SLC12A5_4_methylation_rate_ave,

data = ABBBS, ranges = list(cost = 10^(seq(-4, 5, 0.1)), gamma = 10^(seq(-5,

4, 0.1))), tunecontrol = tune.control(sampling = "cross",

cross = 10), scale = FALSE)

Parameters:

SVM-Type: eps-regression

SVM-Kernel: radial

cost: 79432.82

gamma: 0.0007943282

epsilon: 0.1

Number of Support Vectors: 30

best.cost <- tunedModel$cost

best.gamma <- tunedModel$gamma

cat("Cost: ", best.cost, "\nGamma: ", best.gamma, "\n")

Cost: 79432.82

Gamma: 0.0007943282

tune_results <- as.data.frame(tuneResult$performances)

tune_results$cost <- log10(tune_results$cost)

tune_results$gamma <- log10(tune_results$gamma)

ggplot(tune_results, aes(x = cost, y = gamma, fill = error)) +

geom_tile() +

geom_point(aes(x = log10(best.cost), y = log10(best.gamma)), color = "blue", size = 1, shape = 21, fill = "blue") +

scale_fill_gradient(low = "white", high = "red") +

labs(title = "SVM Parameter Tuning Results",

x = "Log10(Cost)",

y = "Log10(Gamma)",

fill = "Error") +

theme_minimal()


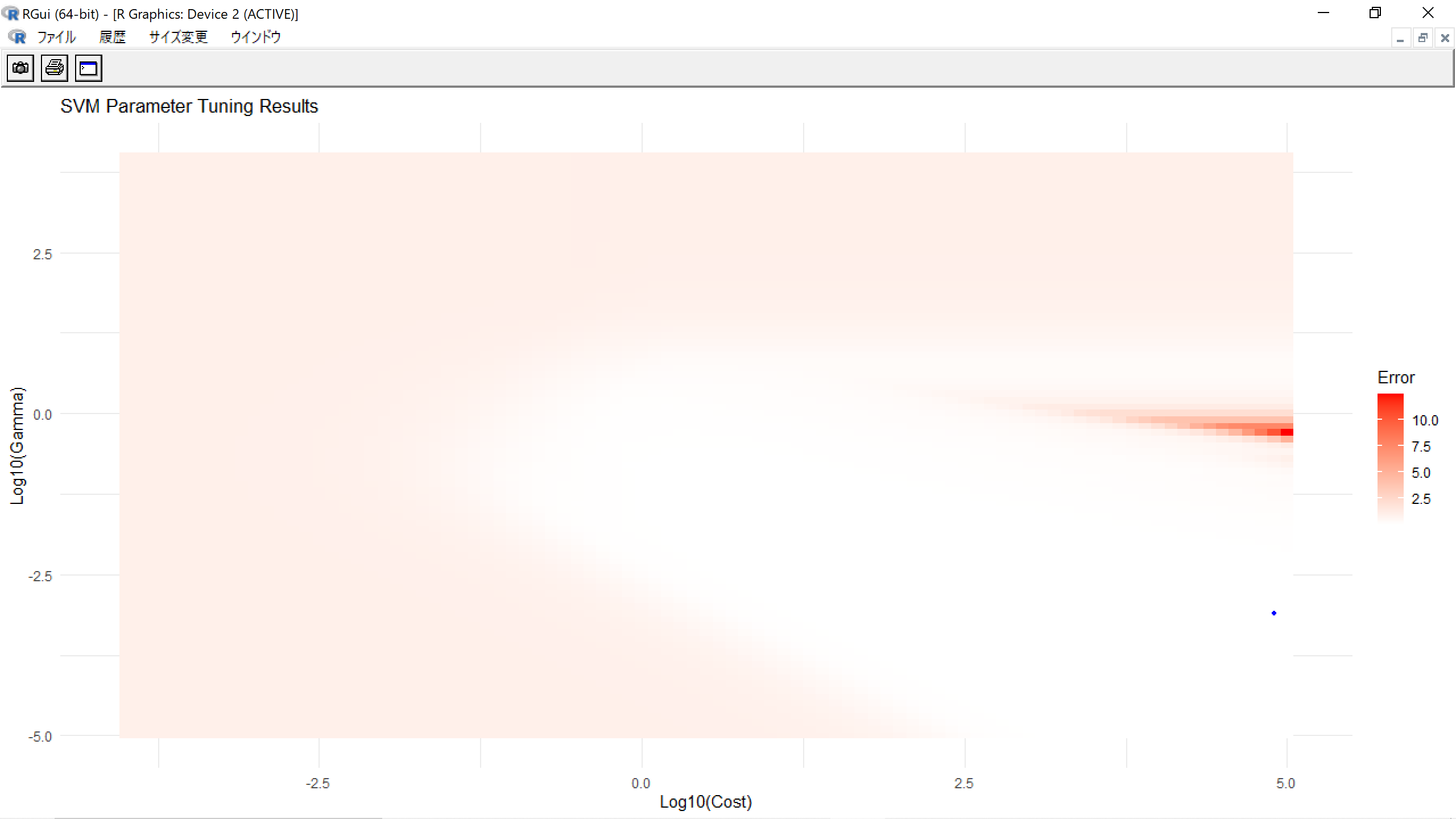


SVRM<-

svm(age~SLC12A5_1_methylation_rate_ave+SLC12A5_2_methylation_rate_ave+SLC12A5_4_methylation_rate_ave,　data=ABBBS,

cost=best.cost, gamma=best.gamma, epsilon=0.1, scale = FALSE)

#LOOCV

nSamples<-nrow(ABBBS)

predict_SVRM_loocv<-numeric(nSamples)

for (z in 1:nSamples){

indices<-removeOne(nSamples,z)

dr<-data.frame(ABBBS$age[indices],ABBBS$SLC12A5_1_methylation_rate_ave[indices],ABBBS$SLC12A5_2_methylation_rate_ave[indices],ABBBS$SLC12A5_4_methylation_rate_ave[indices])

colnames(dr)<-c("age","methylslc_1","methylslc_2","methylslc_4")

bestmodel_SVRM<-svm(age~methylslc_1+methylslc_2+methylslc_4, data=dr,

cost=best.cost, gamma= best.gamma, epsilon=0.1, scale = FALSE)

newdata<-data.frame(methylslc_1=ABBBS$SLC12A5_1_methylation_rate_ave[z],methylslc_2=ABBBS$SLC12A5_2_methylation_rate_ave[z],methylslc_4=ABBBS$SLC12A5_4_methylation_rate_ave[z])

p<-predict(bestmodel_SVRM,newdata)*sd(AGE)+mean(AGE)

if (p<0){p=0}

predict_SVRM_loocv[z]<-p}

ABBB_SVRM_loocv<-cbind(ABBB,predict_SVRM_loocv)

MAE_SVRM_loocv<-mean(abs(ABBB_SVRM_loocv$predict_SVRM_loocv-ABBB$age))

MedianAE_SVRM_loocv<-median(abs(ABBB_SVRM_loocv$predict_SVRM_loocv-ABBB$age))

RMSE_SVRM_loocv<- sqrt(mean((ABBB_SVRM_loocv$predict_SVRM_loocv-ABBB$age)^2))

cat("MAE:", MAE_SVRM_loocv, "\nMed AE:", MedianAE_SVRM_loocv, "\nRMSE:", RMSE_SVRM_loocv, "\n")

MAE: 1.135542

Med AE: 0.7089896

RMSE: 1.528604

g_SVRM_loocv<-ggplot(ABBB_SVRM_loocv,aes(age,predict_SVRM_loocv))+theme_bw()+

annotate("segment",x=min(ABBB$age),xend=max(ABBB$age),y=min(ABBB$age)+1.135542,yend=max(ABBB$age)+1.135542,colour="orchid4",linetype=2,linewidth =0.7)+

annotate("segment",x= min(ABBB$age),xend=max(ABBB$age), y=min(ABBB$age)-1.135542,yend=max(ABBB$age)-1.135542,colour="orchid4",linetype=2,linewidth =0.7)+

geom_point(aes(shape=environment,color=sex),size=2,stroke=2)+

labs(x="Chronological age (year)",y="Predicted age (year)")+

scale_shape_manual(name="environment",labels=c("Captive"="captive","Wild"="wild"),values=c("Captive"=1, "Wild"=3))+

scale_color_manual(name="sex",labels=c("F"="female","M"="male"),values=c("F"="firebrick2","M"="dodgerblue4"))+

theme(axis.text.x=element_text(size=20),axis.text.y=element_text(size=20))+

theme(axis.title.x=element_text(size=17),axis.title.y=element_text(size=17))+

geom_line(aes(y =age), linewidth=1)+

labs(title="SVR model")+

theme(title=element_text(size=17),plot.title=element_text(hjust=0.5))+

scale_y_continuous(limits=c(-5,40))+

scale_x_continuous(limits=c(-5,40))+

labs(subtitle="SLC12A5-1, -2, -4")+

theme(plot.subtitle=element_text(size=15,hjust=0.5))


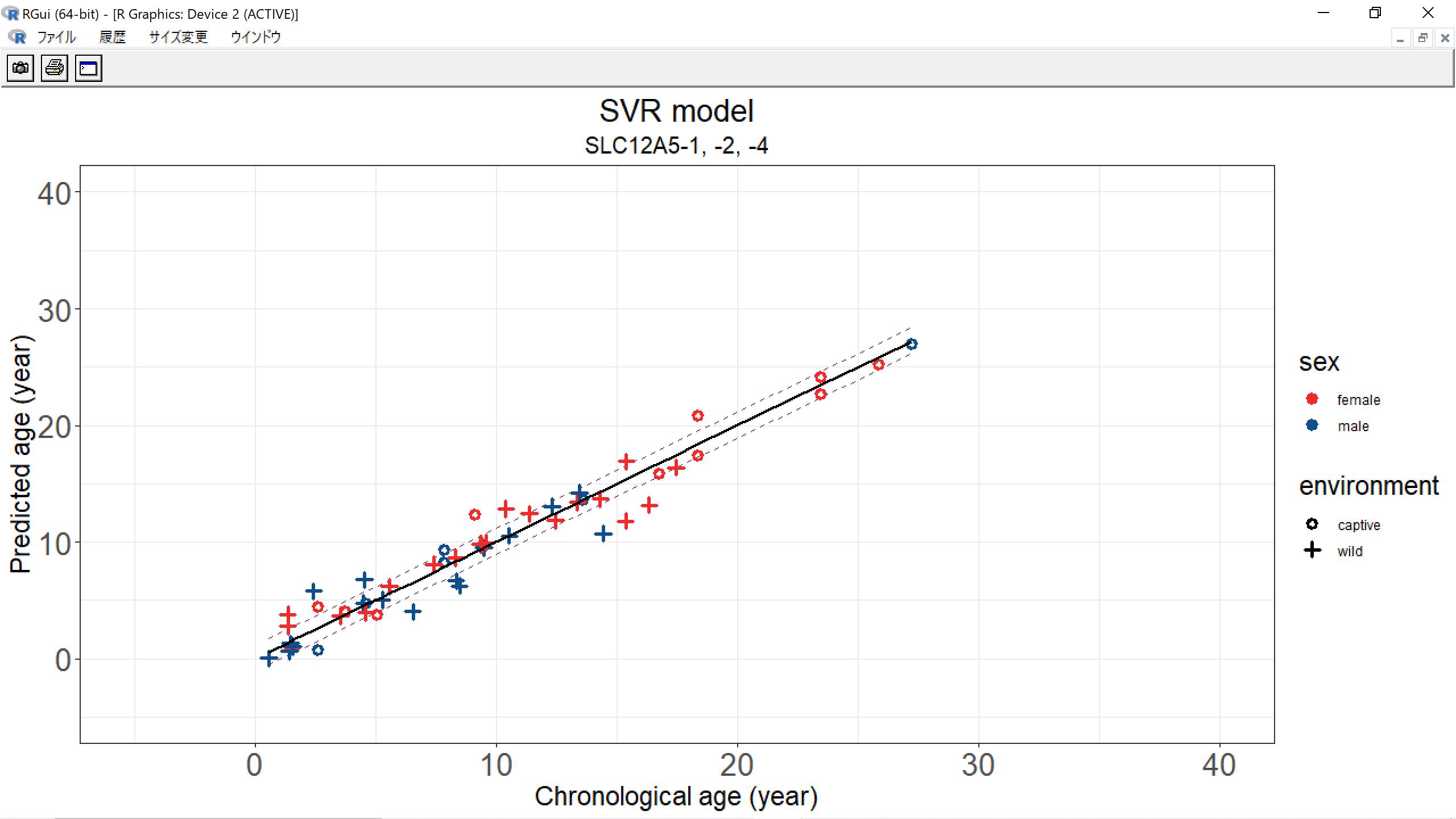


Support vector regression (SLC12A5-1, -3, -4)

set.seed(1)

tuneResult<-tune(svm,age~SLC12A5_1_methylation_rate_ave+SLC12A5_3_methylation_rate_ave+SLC12A5_4_methylation_rate_ave,data=ABBBS,

ranges=list(cost=10^(seq(-4,5,0.1)),gamma=10^(seq(-5,4,0.1))),

tunecontrol = tune.control(sampling = "cross", cross = 10), scale = FALSE)

tunedModel <- tuneResult$best.model

tunedModel

Call:

best.tune(METHOD = svm, train.x = age ~ SLC12A5_1_methylation_rate_ave +

SLC12A5_3_methylation_rate_ave + SLC12A5_4_methylation_rate_ave,

data = ABBBS, ranges = list(cost = 10^(seq(-4, 5, 0.1)), gamma = 10^(seq(-5,

4, 0.1))), tunecontrol = tune.control(sampling = "cross",

cross = 10), scale = FALSE)

Parameters:

SVM-Type: eps-regression

SVM-Kernel: radial

cost: 251.1886

gamma: 0.006309573

epsilon: 0.1

Number of Support Vectors: 31

best.cost <- tunedModel$cost

best.gamma <- tunedModel$gamma

cat("Cost: ", best.cost, "\nGamma: ", best.gamma, "\n")

Cost: 251.1886

Gamma: 0.006309573

tune_results <- as.data.frame(tuneResult$performances)

tune_results$cost <- log10(tune_results$cost)

tune_results$gamma <- log10(tune_results$gamma)

ggplot(tune_results, aes(x = cost, y = gamma, fill = error)) +

geom_tile() +

geom_point(aes(x = log10(best.cost), y = log10(best.gamma)), color = "blue", size = 1, shape = 21, fill = "blue") +

scale_fill_gradient(low = "white", high = "red") +

labs(title = "SVM Parameter Tuning Results",

x = "Log10(Cost)",

y = "Log10(Gamma)",

fill = "Error") +

theme_minimal()


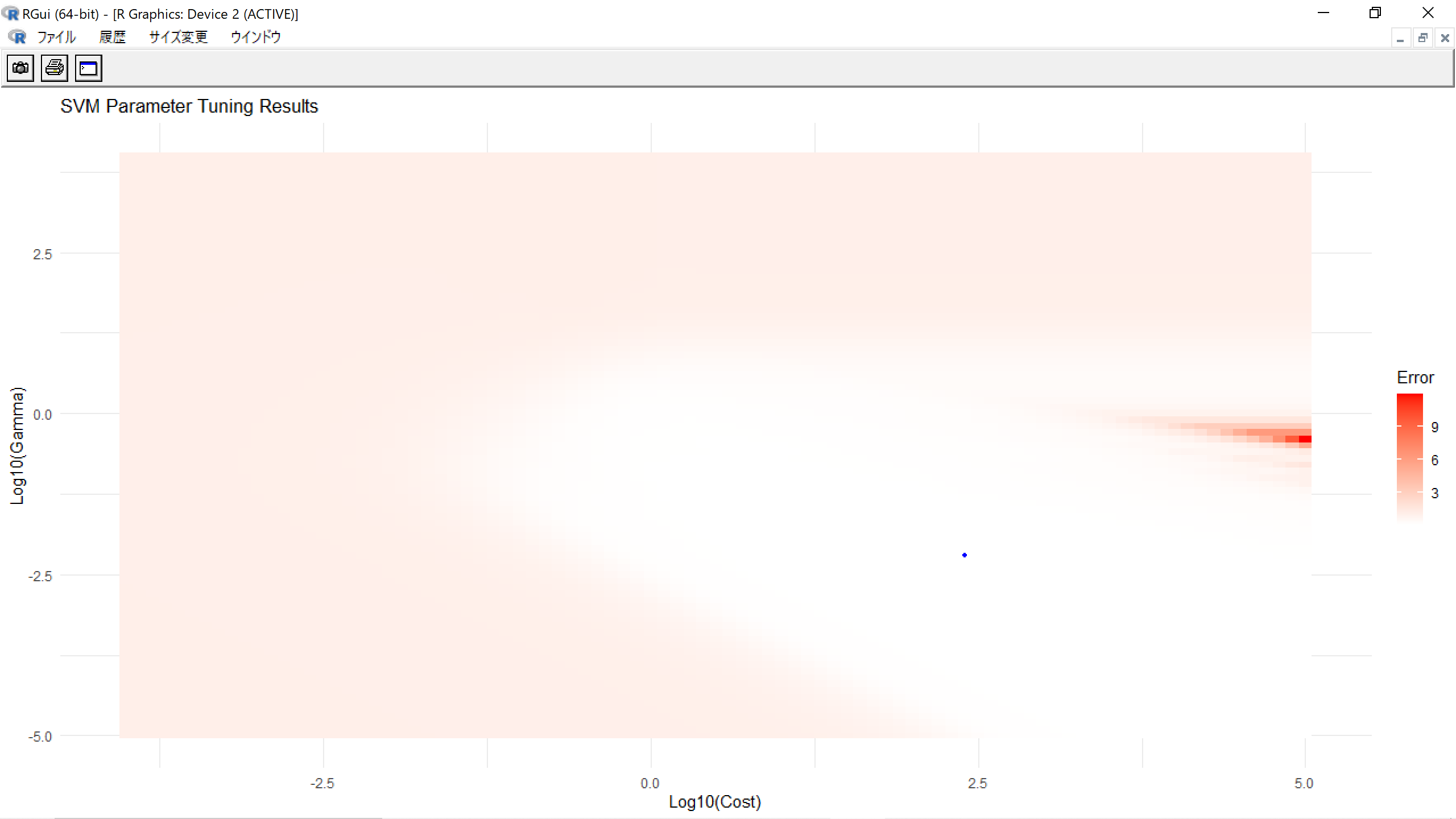


SVRM<-

svm(age~SLC12A5_1_methylation_rate_ave+SLC12A5_3_methylation_rate_ave+SLC12A5_4_methylation_rate_ave,　data=ABBBS,

cost=best.cost, gamma=best.gamma, epsilon=0.1, scale = FALSE)

#LOOCV

nSamples<-nrow(ABBBS)

predict_SVRM_loocv<-numeric(nSamples)

for (z in 1:nSamples){

indices<-removeOne(nSamples,z)

dr<-data.frame(ABBBS$age[indices],ABBBS$SLC12A5_1_methylation_rate_ave[indices],ABBBS$SLC12A5_3_methylation_rate_ave[indices],ABBBS$SLC12A5_4_methylation_rate_ave[indices])

colnames(dr)<-c("age","methylslc_1","methylslc_3","methylslc_4")

bestmodel_SVRM<-svm(age~methylslc_1+methylslc_3+methylslc_4, data=dr,

cost=best.cost, gamma= best.gamma, epsilon=0.1, scale = FALSE)

newdata<-data.frame(methylslc_1=ABBBS$SLC12A5_1_methylation_rate_ave[z],methylslc_3=ABBBS$SLC12A5_3_methylation_rate_ave[z],methylslc_4=ABBBS$SLC12A5_4_methylation_rate_ave[z])

p<-predict(bestmodel_SVRM,newdata)*sd(AGE)+mean(AGE)

if (p<0){p=0}

predict_SVRM_loocv[z]<-p}

ABBB_SVRM_loocv<-cbind(ABBB,predict_SVRM_loocv)

MAE_SVRM_loocv<-mean(abs(ABBB_SVRM_loocv$predict_SVRM_loocv-ABBB$age))

MedianAE_SVRM_loocv<-median(abs(ABBB_SVRM_loocv$predict_SVRM_loocv-ABBB$age))

RMSE_SVRM_loocv<- sqrt(mean((ABBB_SVRM_loocv$predict_SVRM_loocv-ABBB$age)^2))

cat("MAE:", MAE_SVRM_loocv, "\nMed AE:", MedianAE_SVRM_loocv, "\nRMSE:", RMSE_SVRM_loocv, "\n")

MAE: 1.168572

Med AE: 0.8559905

RMSE: 1.51406

Support vector regression (SLC12A5-2, -3, -4)

set.seed(1)

tuneResult<-

tune(svm,age~SLC12A5_2_methylation_rate_ave+SLC12A5_3_methylation_rate_ave+SLC12A5_4_methylation_rate_ave,data=ABBBS,

ranges=list(cost=10^(seq(-4,5,0.1)),gamma=10^(seq(-5,4,0.1))),

tunecontrol = tune.control(sampling = "cross", cross = 10), scale = FALSE)

tunedModel <- tuneResult$best.model

tunedModel

Call:

best.tune(METHOD = svm, train.x = age ~ SLC12A5_2_methylation_rate_ave +

SLC12A5_3_methylation_rate_ave + SLC12A5_4_methylation_rate_ave,

data = ABBBS, ranges = list(cost = 10^(seq(-4, 5, 0.1)), gamma = 10^(seq(-5,

4, 0.1))), tunecontrol = tune.control(sampling = "cross",

cross = 10), scale = FALSE)

Parameters:

SVM-Type: eps-regression

SVM-Kernel: radial

cost: 125.8925

gamma: 0.01584893

epsilon: 0.1

Number of Support Vectors: 30

best.cost <- tunedModel$cost

best.gamma <- tunedModel$gamma

cat("Cost: ", best.cost, "\nGamma: ", best.gamma, "\n")

Cost: 125.8925

Gamma: 0.01584893

tune_results <- as.data.frame(tuneResult$performances)

tune_results$cost <- log10(tune_results$cost)

tune_results$gamma <- log10(tune_results$gamma)

ggplot(tune_results, aes(x = cost, y = gamma, fill = error)) +

geom_tile() +

geom_point(aes(x = log10(best.cost), y = log10(best.gamma)), color = "blue", size = 1, shape = 21, fill = "blue") +

scale_fill_gradient(low = "white", high = "red") +

labs(title = "SVM Parameter Tuning Results",

x = "Log10(Cost)",

y = "Log10(Gamma)",

fill = "Error") +

theme_minimal()


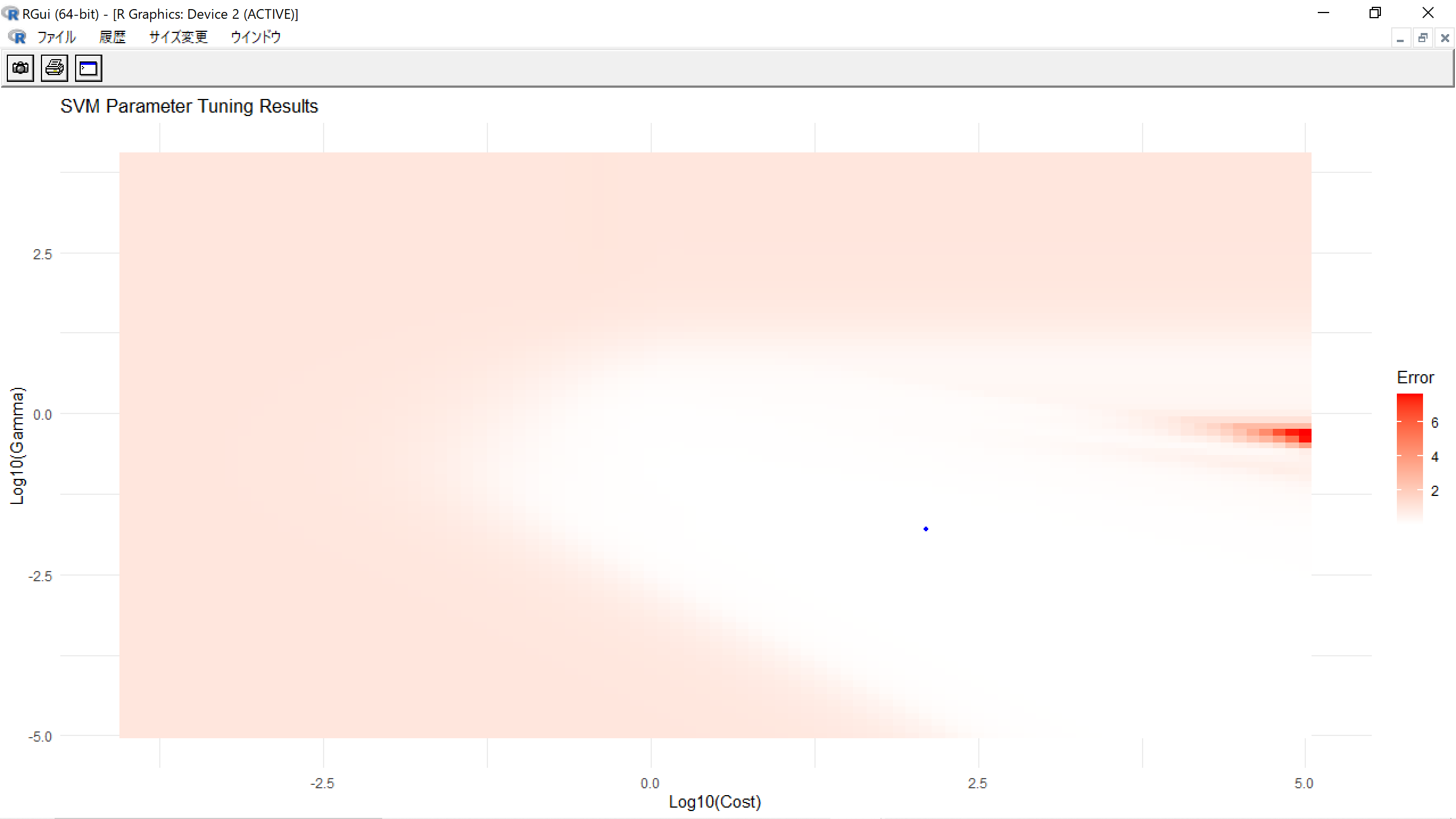


SVRM<-

svm(age~SLC12A5_2_methylation_rate_ave+SLC12A5_3_methylation_rate_ave+SLC12A5_4_methylation_rate_ave,　data=ABBBS,

cost=best.cost, gamma=best.gamma, epsilon=0.1, scale = FALSE)

#LOOCV

nSamples<-nrow(ABBBS)

predict_SVRM_loocv<-numeric(nSamples)

for (z in 1:nSamples){

indices<-removeOne(nSamples,z)

dr<-data.frame(ABBBS$age[indices],ABBBS$age[indices],ABBBS$SLC12A5_2_methylation_rate_ave[indices],ABBBS$SLC12A5_3_methylation_rate_ave[indices],ABBBS$SLC12A5_4_methylation_rate_ave[indices])

colnames(dr)<-c("age","methylslc_2","methylslc_3","methylslc_4")

bestmodel_SVRM<-svm(age~methylslc_2+methylslc_3+methylslc_4, data=dr,

cost=best.cost, gamma= best.gamma, epsilon=0.1, scale = FALSE)

newdata<-data.frame(methylslc_2=ABBBS$SLC12A5_2_methylation_rate_ave[z],methylslc_3=ABBBS$SLC12A5_3_methylation_rate_ave[z],methylslc_4=ABBBS$SLC12A5_4_methylation_rate_ave[z])

p<-predict(bestmodel_SVRM,newdata)*sd(AGE)+mean(AGE)

if (p<0){p=0}

predict_SVRM_loocv[z]<-p}

ABBB_SVRM_loocv<-cbind(ABBB,predict_SVRM_loocv)

MAE_SVRM_loocv<-mean(abs(ABBB_SVRM_loocv$predict_SVRM_loocv-ABBB$age))

MedianAE_SVRM_loocv<-median(abs(ABBB_SVRM_loocv$predict_SVRM_loocv-ABBB$age))

RMSE_SVRM_loocv<- sqrt(mean((ABBB_SVRM_loocv$predict_SVRM_loocv-ABBB$age)^2))

cat("MAE:", MAE_SVRM_loocv, "\nMed AE:", MedianAE_SVRM_loocv, "\nRMSE:", RMSE_SVRM_loocv, "\n")

MAE: 1.421176

Med AE: 1.072741

RMSE: 1.804017

Support vector regression (SLC12A5-1, -2)

set.seed(1)

tuneResult<-

tune(svm,age~SLC12A5_1_methylation_rate_ave+SLC12A5_2_methylation_rate_ave,data=ABBBS,

ranges=list(cost=10^(seq(-4,5,0.1)),gamma=10^(seq(-5,4,0.1))),

tunecontrol = tune.control(sampling = "cross", cross = 10), scale = FALSE)

tunedModel <- tuneResult$best.model

tunedModel

Call:

best.tune(METHOD = svm, train.x = age ~ SLC12A5_1_methylation_rate_ave +

SLC12A5_2_methylation_rate_ave, data = ABBBS, ranges = list(cost = 10^(seq(-4,

5, 0.1)), gamma = 10^(seq(-5, 4, 0.1))), tunecontrol = tune.control(sampling = "cross",

cross = 10), scale = FALSE)

Parameters:

SVM-Type: eps-regression

SVM-Kernel: radial

cost: 50.11872

gamma: 0.002511886

epsilon: 0.1

Number of Support Vectors: 38

best.cost <- tunedModel$cost

best.gamma <- tunedModel$gamma

cat("Cost: ", best.cost, "\nGamma: ", best.gamma, "\n")

Cost: 50.11872

Gamma: 0.002511886

tune_results <- as.data.frame(tuneResult$performances)

tune_results$cost <- log10(tune_results$cost)

tune_results$gamma <- log10(tune_results$gamma)

ggplot(tune_results, aes(x = cost, y = gamma, fill = error)) +

geom_tile() +

geom_point(aes(x = log10(best.cost), y = log10(best.gamma)), color = "blue", size = 1, shape = 21, fill = "blue") +

scale_fill_gradient(low = "white", high = "red") +

labs(title = "SVM Parameter Tuning Results",

x = "Log10(Cost)",

y = "Log10(Gamma)",

fill = "Error") +

theme_minimal()


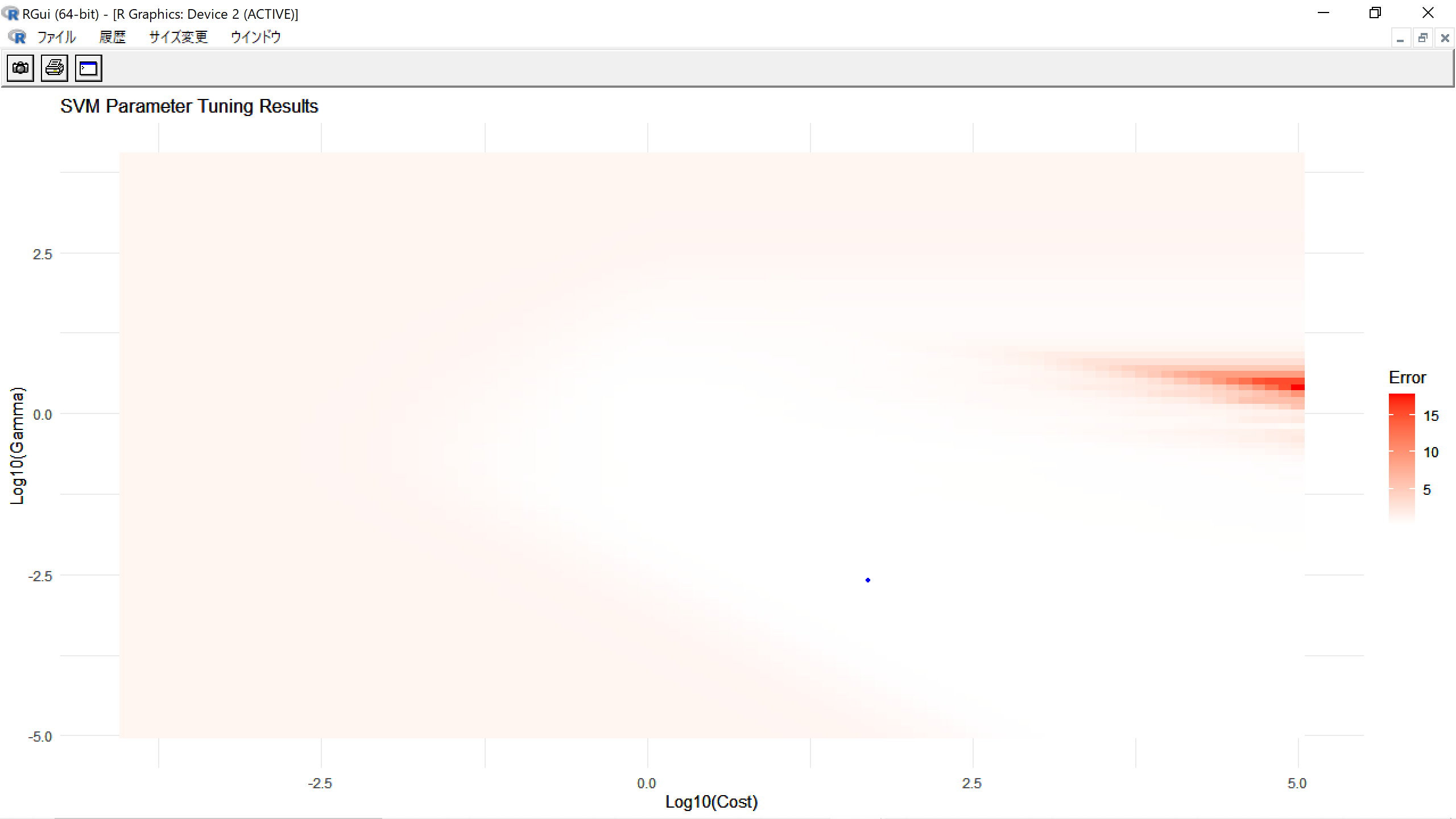


SVRM<-

svm(age~SLC12A5_1_methylation_rate_ave+SLC12A5_2_methylation_rate_ave,　data=ABBBS,

cost=best.cost, gamma=best.gamma, epsilon=0.1, scale = FALSE)

#LOOCV

nSamples<-nrow(ABBBS)

predict_SVRM_loocv<-numeric(nSamples)

for (z in 1:nSamples){

indices<-removeOne(nSamples,z)

dr<-data.frame(ABBBS$age[indices],ABBBS$age[indices],ABBBS$SLC12A5_1_methylation_rate_ave[indices],ABBBS$SLC12A5_2_methylation_rate_ave[indices])

colnames(dr)<-c("age","methylslc_1","methylslc_2")

bestmodel_SVRM<-svm(age~methylslc_1+methylslc_2, data=dr,

cost=best.cost, gamma= best.gamma, epsilon=0.1, scale = FALSE)

newdata<-data.frame(methylslc_1=ABBBS$SLC12A5_1_methylation_rate_ave[z],methylslc_2=ABBBS$SLC12A5_2_methylation_rate_ave[z])

p<-predict(bestmodel_SVRM,newdata)*sd(AGE)+mean(AGE)

if (p<0){p=0}

predict_SVRM_loocv[z]<-p}

ABBB_SVRM_loocv<-cbind(ABBB,predict_SVRM_loocv)

MAE_SVRM_loocv<-mean(abs(ABBB_SVRM_loocv$predict_SVRM_loocv-ABBB$age))

MedianAE_SVRM_loocv<-median(abs(ABBB_SVRM_loocv$predict_SVRM_loocv-ABBB$age))

RMSE_SVRM_loocv<- sqrt(mean((ABBB_SVRM_loocv$predict_SVRM_loocv-ABBB$age)^2))

cat("MAE:", MAE_SVRM_loocv, "\nMed AE:", MedianAE_SVRM_loocv, "\nRMSE:", RMSE_SVRM_loocv, "\n")

MAE: 1.668735

Med AE: 1.145043

RMSE: 2.112639

Support vector regression (SLC12A5-1, -3)

set.seed(1)

tuneResult<-

tune(svm,age~SLC12A5_1_methylation_rate_ave+SLC12A5_3_methylation_rate_ave,data=ABBBS,

ranges=list(cost=10^(seq(-4,5,0.1)),gamma=10^(seq(-5,4,0.1))),

tunecontrol = tune.control(sampling = "cross", cross = 10), scale = FALSE)

tunedModel <- tuneResult$best.model

tunedModel

Call:

best.tune(METHOD = svm, train.x = age ~ SLC12A5_1_methylation_rate_ave +

SLC12A5_3_methylation_rate_ave, data = ABBBS, ranges = list(cost = 10^(seq(-4,

5, 0.1)), gamma = 10^(seq(-5, 4, 0.1))), tunecontrol = tune.control(sampling = "cross",

cross = 10), scale = FALSE)

Parameters:

SVM-Type: eps-regression

SVM-Kernel: radial

cost: 2.511886

gamma: 0.2511886

epsilon: 0.1

Number of Support Vectors: 36

best.cost <- tunedModel$cost

best.gamma <- tunedModel$gamma

cat("Cost: ", best.cost, "\nGamma: ", best.gamma, "\n")

Cost: 2.511886

Gamma: 0.2511886

tune_results <- as.data.frame(tuneResult$performances)

tune_results$cost <- log10(tune_results$cost)

tune_results$gamma <- log10(tune_results$gamma)

ggplot(tune_results, aes(x = cost, y = gamma, fill = error)) +

geom_tile() +

geom_point(aes(x = log10(best.cost), y = log10(best.gamma)), color = "blue", size = 1, shape = 21, fill = "blue") +

scale_fill_gradient(low = "white", high = "red") +

labs(title = "SVM Parameter Tuning Results",

x = "Log10(Cost)",

y = "Log10(Gamma)",

fill = "Error") +

theme_minimal()


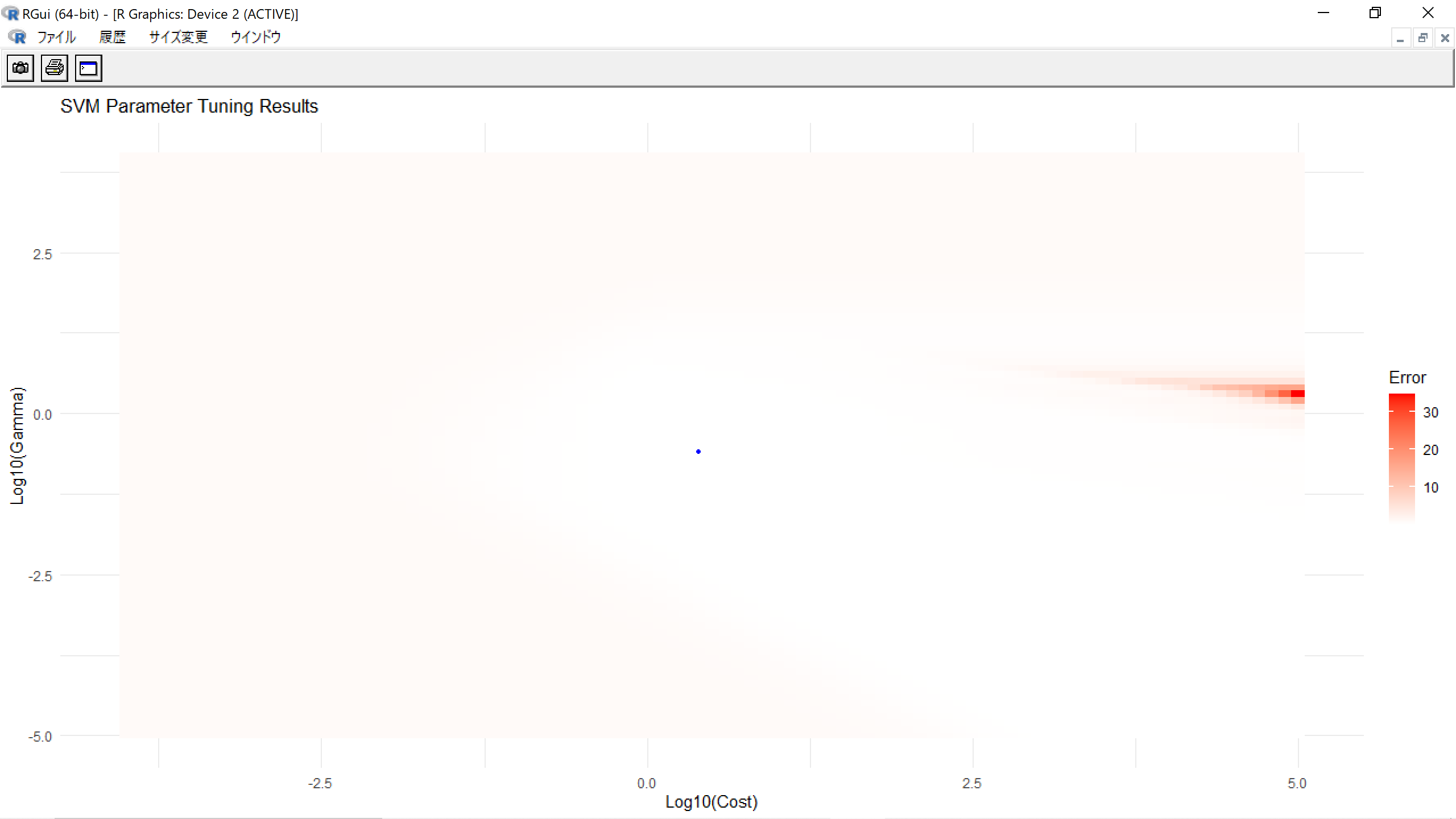


SVRM<-

svm(age~SLC12A5_1_methylation_rate_ave+SLC12A5_3_methylation_rate_ave,　data=ABBBS,

cost=best.cost, gamma=best.gamma, epsilon=0.1, scale = FALSE)

#LOOCV

nSamples<-nrow(ABBBS)

predict_SVRM_loocv<-numeric(nSamples)

for (z in 1:nSamples){

indices<-removeOne(nSamples,z)

dr<-data.frame(ABBBS$age[indices],ABBBS$SLC12A5_1_methylation_rate_ave[indices],ABBBS$SLC12A5_3_methylation_rate_ave[indices])

colnames(dr)<-c("age","methylslc_1","methylslc_3")

bestmodel_SVRM<-svm(age~methylslc_1+methylslc_3, data=dr,

cost=best.cost, gamma= best.gamma, epsilon=0.1, scale = FALSE)

newdata<-data.frame(methylslc_1=ABBBS$SLC12A5_1_methylation_rate_ave[z],methylslc_3=ABBBS$SLC12A5_3_methylation_rate_ave[z])

p<-predict(bestmodel_SVRM,newdata)*sd(AGE)+mean(AGE)

if (p<0){p=0}

predict_SVRM_loocv[z]<-p}

ABBB_SVRM_loocv<-cbind(ABBB,predict_SVRM_loocv)

MAE_SVRM_loocv<-mean(abs(ABBB_SVRM_loocv$predict_SVRM_loocv-ABBB$age))

MedianAE_SVRM_loocv<-median(abs(ABBB_SVRM_loocv$predict_SVRM_loocv-ABBB$age))

RMSE_SVRM_loocv<- sqrt(mean((ABBB_SVRM_loocv$predict_SVRM_loocv-ABBB$age)^2))

cat("MAE:", MAE_SVRM_loocv, "\nMed AE:", MedianAE_SVRM_loocv, "\nRMSE:", RMSE_SVRM_loocv, "\n")

MAE: 1.463754

Med AE: 1.013653

RMSE: 1.91697

Support vector regression (SLC12A5-1, -4)

set.seed(1)

tuneResult<-

tune(svm,age~SLC12A5_1_methylation_rate_ave+SLC12A5_4_methylation_rate_ave,data=ABBBS,

ranges=list(cost=10^(seq(-4,5,0.1)),gamma=10^(seq(-5,4,0.1))),

tunecontrol = tune.control(sampling = "cross", cross = 10), scale = FALSE)

tunedModel <- tuneResult$best.model

tunedModel

Call:

best.tune(METHOD = svm, train.x = age ~ SLC12A5_1_methylation_rate_ave +

SLC12A5_4_methylation_rate_ave, data = ABBBS, ranges = list(cost = 10^(seq(-4,

5, 0.1)), gamma = 10^(seq(-5, 4, 0.1))), tunecontrol = tune.control(sampling = "cross",

cross = 10), scale = FALSE)

Parameters:

SVM-Type: eps-regression

SVM-Kernel: radial

cost: 31.62278

gamma: 0.006309573

epsilon: 0.1

Number of Support Vectors: 33

best.cost <- tunedModel$cost

best.gamma <- tunedModel$gamma

cat("Cost: ", best.cost, "\nGamma: ", best.gamma, "\n")

Cost: 31.62278

Gamma: 0.006309573

tune_results <- as.data.frame(tuneResult$performances)

tune_results$cost <- log10(tune_results$cost)

tune_results$gamma <- log10(tune_results$gamma)

ggplot(tune_results, aes(x = cost, y = gamma, fill = error)) +

geom_tile() +

geom_point(aes(x = log10(best.cost), y = log10(best.gamma)), color = "blue", size = 1, shape = 21, fill = "blue") +

scale_fill_gradient(low = "white", high = "red") +

labs(title = "SVM Parameter Tuning Results",

x = "Log10(Cost)",

y = "Log10(Gamma)",

fill = "Error") +

theme_minimal()


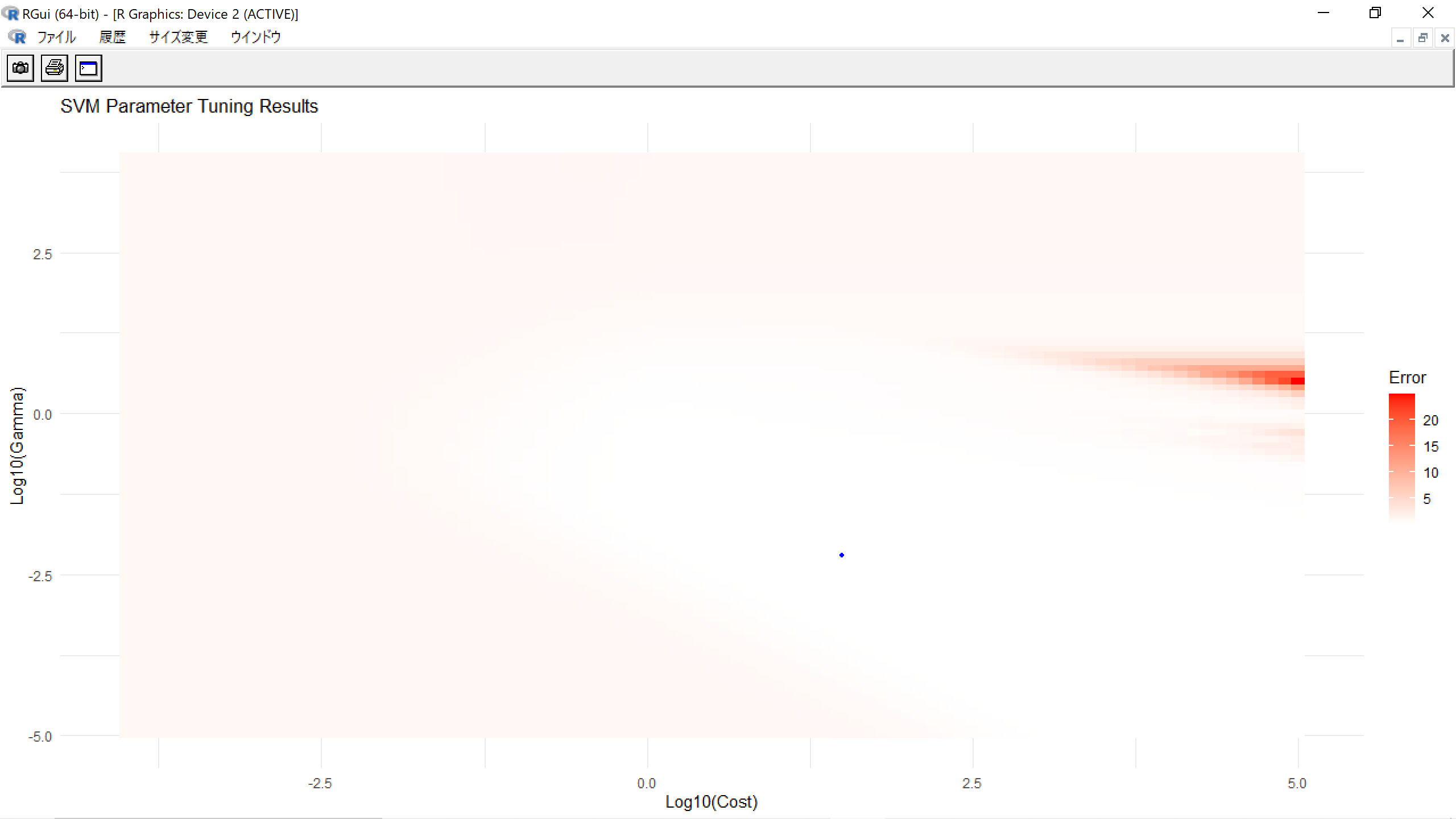


SVRM<-

svm(age~SLC12A5_1_methylation_rate_ave+SLC12A5_4_methylation_rate_ave,　data=ABBBS,

cost=best.cost, gamma=best.gamma, epsilon=0.1, scale = FALSE)

#LOOCV

nSamples<-nrow(ABBBS)

predict_SVRM_loocv<-numeric(nSamples)

for (z in 1:nSamples){

indices<-removeOne(nSamples,z)

dr<-data.frame(ABBBS$age[indices],ABBBS$SLC12A5_1_methylation_rate_ave[indices],ABBBS$SLC12A5_4_methylation_rate_ave[indices])

colnames(dr)<-c("age","methylslc_1","methylslc_4")

bestmodel_SVRM<-svm(age~methylslc_1+methylslc_4, data=dr,

cost=best.cost, gamma= best.gamma, epsilon=0.1, scale = FALSE)

newdata<-data.frame(methylslc_1=ABBBS$SLC12A5_1_methylation_rate_ave[z],methylslc_4=ABBBS$SLC12A5_4_methylation_rate_ave[z])

p<-predict(bestmodel_SVRM,newdata)*sd(AGE)+mean(AGE)

if (p<0){p=0}

predict_SVRM_loocv[z]<-p}

ABBB_SVRM_loocv<-cbind(ABBB,predict_SVRM_loocv)

MAE_SVRM_loocv<-mean(abs(ABBB_SVRM_loocv$predict_SVRM_loocv-ABBB$age))

MedianAE_SVRM_loocv<-median(abs(ABBB_SVRM_loocv$predict_SVRM_loocv-ABBB$age))

RMSE_SVRM_loocv<- sqrt(mean((ABBB_SVRM_loocv$predict_SVRM_loocv-ABBB$age)^2))

cat("MAE:", MAE_SVRM_loocv, "\nMed AE:", MedianAE_SVRM_loocv, "\nRMSE:", RMSE_SVRM_loocv, "\n")

MAE: 1.183381

Med AE: 0.8004908

RMSE: 1.557492

Support vector regression (SLC12A5-2, -3)

set.seed(1)

tuneResult<-

tune(svm,age~SLC12A5_2_methylation_rate_ave+SLC12A5_3_methylation_rate_ave,data=ABBBS,

ranges=list(cost=10^(seq(-4,5,0.1)),gamma=10^(seq(-5,4,0.1))),

tunecontrol = tune.control(sampling = "cross", cross = 10), scale = FALSE)

tunedModel <- tuneResult$best.model

tunedModel

Call:

best.tune(METHOD = svm, train.x = age ~ SLC12A5_2_methylation_rate_ave +

SLC12A5_3_methylation_rate_ave, data = ABBBS, ranges = list(cost = 10^(seq(-4,

5, 0.1)), gamma = 10^(seq(-5, 4, 0.1))), tunecontrol = tune.control(sampling = "cross",

cross = 10), scale = FALSE)

Parameters:

SVM-Type: eps-regression

SVM-Kernel: radial

cost: 63095.73

gamma: 0.003981072

epsilon: 0.1

Number of Support Vectors: 38

best.cost <- tunedModel$cost

best.gamma <- tunedModel$gamma

cat("Cost: ", best.cost, "\nGamma: ", best.gamma, "\n")

Cost: 63095.73

Gamma: 0.003981072

tune_results <- as.data.frame(tuneResult$performances)

tune_results$cost <- log10(tune_results$cost)

tune_results$gamma <- log10(tune_results$gamma)

ggplot(tune_results, aes(x = cost, y = gamma, fill = error)) +

geom_tile() +

geom_point(aes(x = log10(best.cost), y = log10(best.gamma)), color = "blue", size = 1, shape = 21, fill = "blue") +

scale_fill_gradient(low = "white", high = "red") +

labs(title = "SVM Parameter Tuning Results",

x = "Log10(Cost)",

y = "Log10(Gamma)",

fill = "Error") +

theme_minimal()


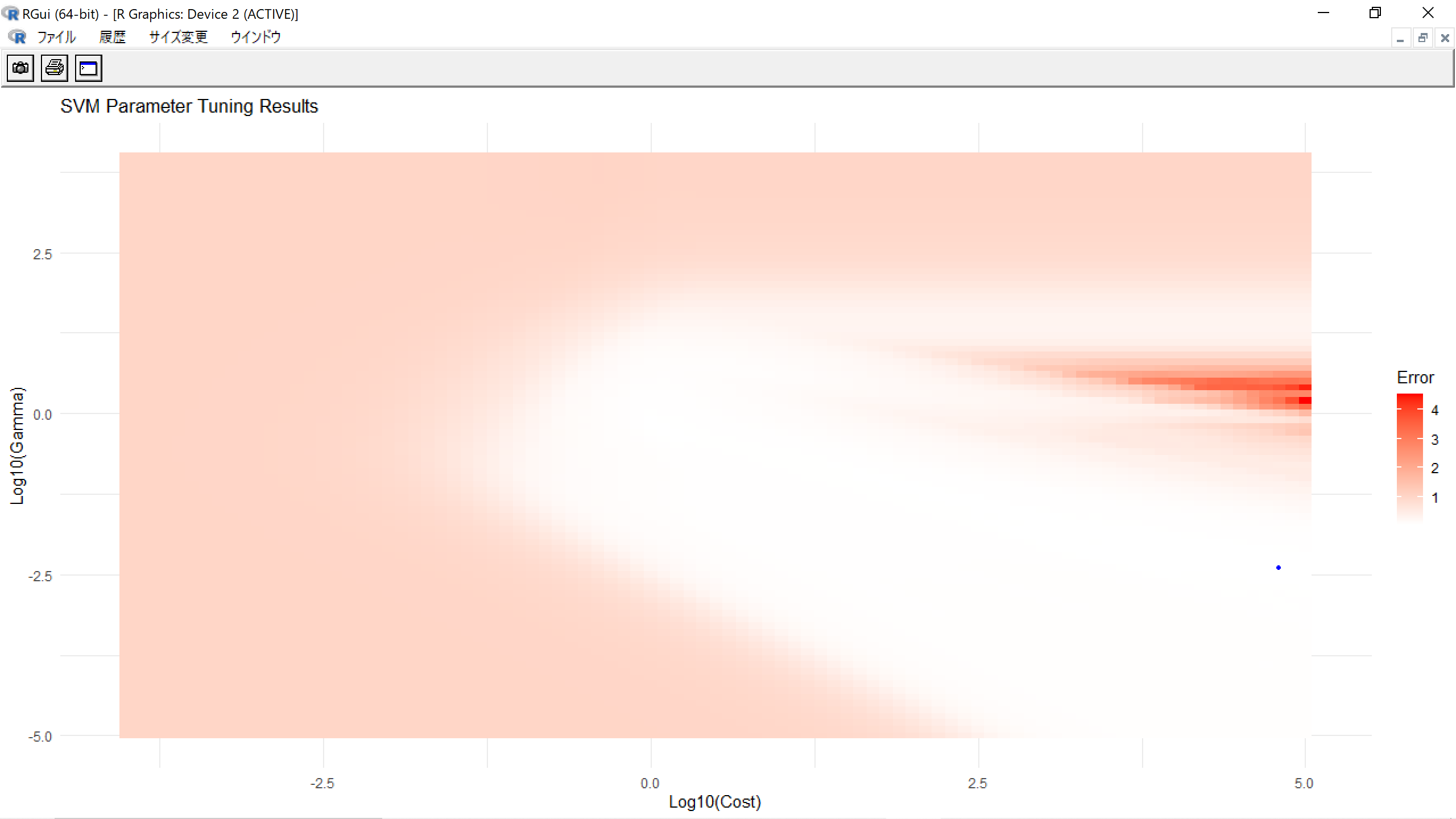


SVRM<-

svm(age~SLC12A5_2_methylation_rate_ave+SLC12A5_3_methylation_rate_ave,　data=ABBBS,

cost=best.cost, gamma=best.gamma, epsilon=0.1, scale = FALSE)

#LOOCV

nSamples<-nrow(ABBBS)

predict_SVRM_loocv<-numeric(nSamples)

for (z in 1:nSamples){

indices<-removeOne(nSamples,z)

dr<-data.frame(ABBBS$age[indices],ABBBS$SLC12A5_2_methylation_rate_ave[indices],ABBBS$SLC12A5_3_methylation_rate_ave[indices])

colnames(dr)<-c("age","methylslc_2","methylslc_3")

bestmodel_SVRM<-svm(age~methylslc_2+methylslc_3, data=dr,

cost=best.cost, gamma= best.gamma, epsilon=0.1, scale = FALSE)

newdata<-data.frame(methylslc_2=ABBBS$SLC12A5_2_methylation_rate_ave[z],methylslc_3=ABBBS$SLC12A5_3_methylation_rate_ave[z])

p<-predict(bestmodel_SVRM,newdata)*sd(AGE)+mean(AGE)

if (p<0){p=0}

predict_SVRM_loocv[z]<-p}

ABBB_SVRM_loocv<-cbind(ABBB,predict_SVRM_loocv)

MAE_SVRM_loocv<-mean(abs(ABBB_SVRM_loocv$predict_SVRM_loocv-ABBB$age))

MedianAE_SVRM_loocv<-median(abs(ABBB_SVRM_loocv$predict_SVRM_loocv-ABBB$age))

RMSE_SVRM_loocv<- sqrt(mean((ABBB_SVRM_loocv$predict_SVRM_loocv-ABBB$age)^2))

cat("MAE:", MAE_SVRM_loocv, "\nMed AE:", MedianAE_SVRM_loocv, "\nRMSE:", RMSE_SVRM_loocv, "\n")

MAE: 1.46688

Med AE: 1.079649

RMSE: 1.833115

Support vector regression (SLC12A5-2, -4)

set.seed(1)

tuneResult<-

tune(svm,age~ SLC12A5_2_methylation_rate_ave+SLC12A5_4_methylation_rate_ave,data=ABBBS,

ranges=list(cost=10^(seq(-4,5,0.1)),gamma=10^(seq(-5,4,0.1))),

tunecontrol = tune.control(sampling = "cross", cross = 10), scale = FALSE)

tunedModel <- tuneResult$best.model

tunedModel

Call:

best.tune(METHOD = svm, train.x = age ~ SLC12A5_2_methylation_rate_ave +

SLC12A5_4_methylation_rate_ave, data = ABBBS, ranges = list(cost = 10^(seq(-4,

5, 0.1)), gamma = 10^(seq(-5, 4, 0.1))), tunecontrol = tune.control(sampling = "cross",

cross = 10), scale = FALSE)

Parameters:

SVM-Type: eps-regression

SVM-Kernel: radial

cost: 31622.78

gamma: 0.01258925

epsilon: 0.1

Number of Support Vectors: 31

best.cost <- tunedModel$cost

best.gamma <- tunedModel$gamma

cat("Cost: ", best.cost, "\nGamma: ", best.gamma, "\n")

Cost: 31622.78

Gamma: 0.01258925

tune_results <- as.data.frame(tuneResult$performances)

tune_results$cost <- log10(tune_results$cost)

tune_results$gamma <- log10(tune_results$gamma)

ggplot(tune_results, aes(x = cost, y = gamma, fill = error)) +

geom_tile() +

geom_point(aes(x = log10(best.cost), y = log10(best.gamma)), color = "blue", size = 1, shape = 21, fill = "blue") +

scale_fill_gradient(low = "white", high = "red") +

labs(title = "SVM Parameter Tuning Results",

x = "Log10(Cost)",

y = "Log10(Gamma)",

fill = "Error") +

theme_minimal()


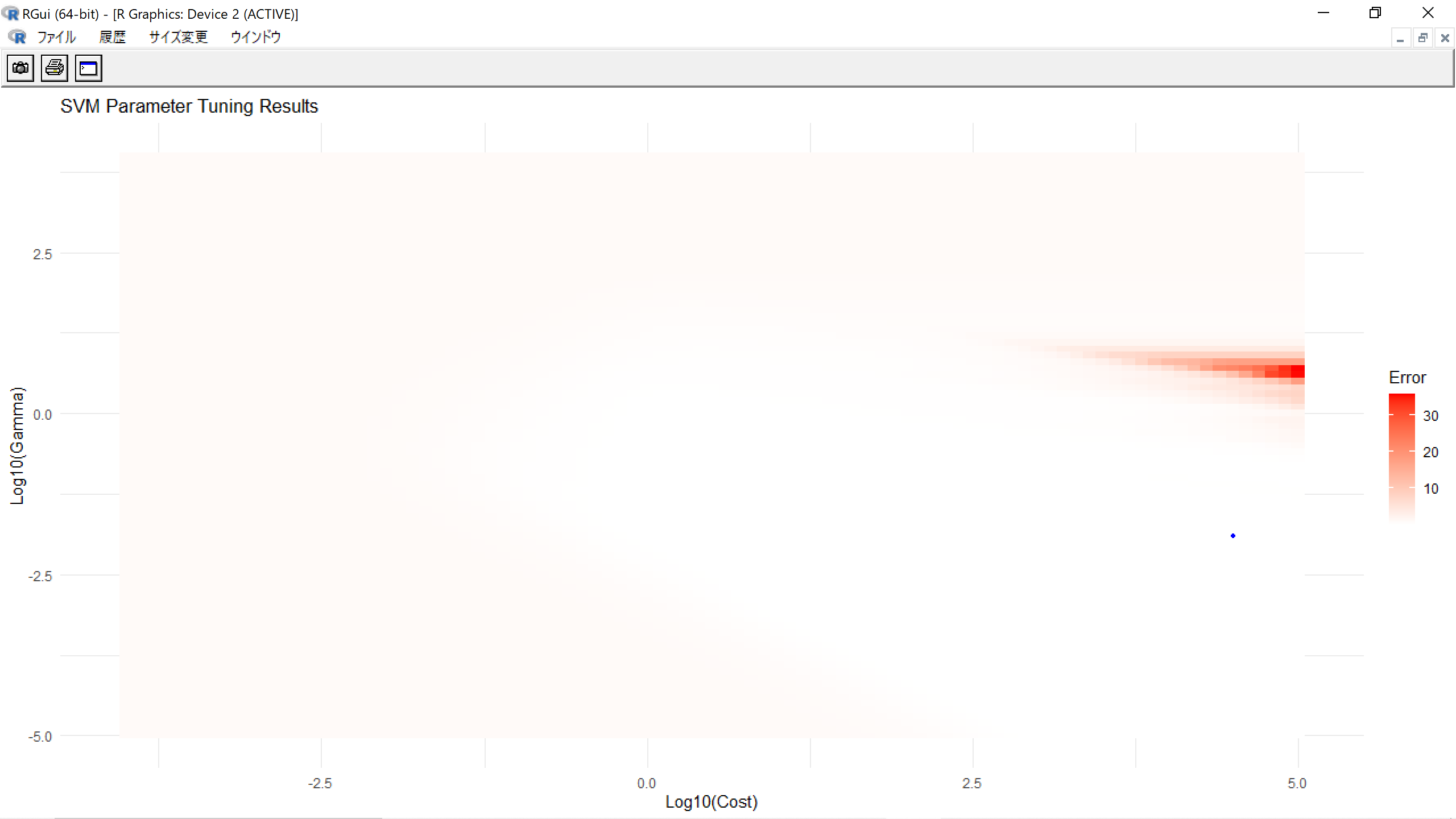


SVRM<-

svm(age~ SLC12A5_2_methylation_rate_ave+SLC12A5_4_methylation_rate_ave,　data=ABBBS,

cost=best.cost, gamma=best.gamma, epsilon=0.1, scale = FALSE)

#LOOCV

nSamples<-nrow(ABBBS)

predict_SVRM_loocv<-numeric(nSamples)

for (z in 1:nSamples){

indices<-removeOne(nSamples,z)

dr<-data.frame(ABBBS$age[indices],ABBBS$SLC12A5_2_methylation_rate_ave[indices],ABBBS$SLC12A5_4_methylation_rate_ave[indices])

colnames(dr)<-c("age","methylslc_2","methylslc_4")

bestmodel_SVRM<-svm(age~methylslc_2+methylslc_4, data=dr,

cost=best.cost, gamma= best.gamma, epsilon=0.1, scale = FALSE)

newdata<-data.frame(methylslc_2=ABBBS$SLC12A5_2_methylation_rate_ave[z],methylslc_4=ABBBS$SLC12A5_4_methylation_rate_ave[z])

p<-predict(bestmodel_SVRM,newdata)*sd(AGE)+mean(AGE)

if (p<0){p=0}

predict_SVRM_loocv[z]<-p}

ABBB_SVRM_loocv<-cbind(ABBB,predict_SVRM_loocv)

MAE_SVRM_loocv<-mean(abs(ABBB_SVRM_loocv$predict_SVRM_loocv-ABBB$age))

MedianAE_SVRM_loocv<-median(abs(ABBB_SVRM_loocv$predict_SVRM_loocv-ABBB$age))

RMSE_SVRM_loocv<- sqrt(mean((ABBB_SVRM_loocv$predict_SVRM_loocv-ABBB$age)^2))

cat("MAE:", MAE_SVRM_loocv, "\nMed AE:", MedianAE_SVRM_loocv, "\nRMSE:", RMSE_SVRM_loocv, "\n")

MAE: 1.160056

Med AE: 0.8483242

RMSE: 1.522913

Support vector regression (SLC12A5-3, -4)

set.seed(1)

tuneResult<-

tune(svm,age~SLC12A5_3_methylation_rate_ave+SLC12A5_4_methylation_rate_ave,data=ABBBS,

ranges=list(cost=10^(seq(-4,5,0.1)),gamma=10^(seq(-5,4,0.1))),

tunecontrol = tune.control(sampling = "cross", cross = 10), scale = FALSE)

tunedModel <- tuneResult$best.model

tunedModel

Call:

best.tune(METHOD = svm, train.x = age ~ SLC12A5_3_methylation_rate_ave +

SLC12A5_4_methylation_rate_ave, data = ABBBS, ranges = list(cost = 10^(seq(-4,

5, 0.1)), gamma = 10^(seq(-5, 4, 0.1))), tunecontrol = tune.control(sampling = "cross",

cross = 10), scale = FALSE)

Parameters:

SVM-Type: eps-regression

SVM-Kernel: radial

cost: 79.43282

gamma: 0.03162278

epsilon: 0.1

Number of Support Vectors: 36

best.cost <- tunedModel$cost

best.gamma <- tunedModel$gamma

cat("Cost: ", best.cost, "\nGamma: ", best.gamma, "\n")

Cost: 79.43282

Gamma: 0.03162278

tune_results <- as.data.frame(tuneResult$performances)

tune_results$cost <- log10(tune_results$cost)

tune_results$gamma <- log10(tune_results$gamma)

ggplot(tune_results, aes(x = cost, y = gamma, fill = error)) +

geom_tile() +

geom_point(aes(x = log10(best.cost), y = log10(best.gamma)), color = "blue", size = 1, shape = 21, fill = "blue") +

scale_fill_gradient(low = "white", high = "red") +

labs(title = "SVM Parameter Tuning Results",

x = "Log10(Cost)",

y = "Log10(Gamma)",

fill = "Error") +

theme_minimal()


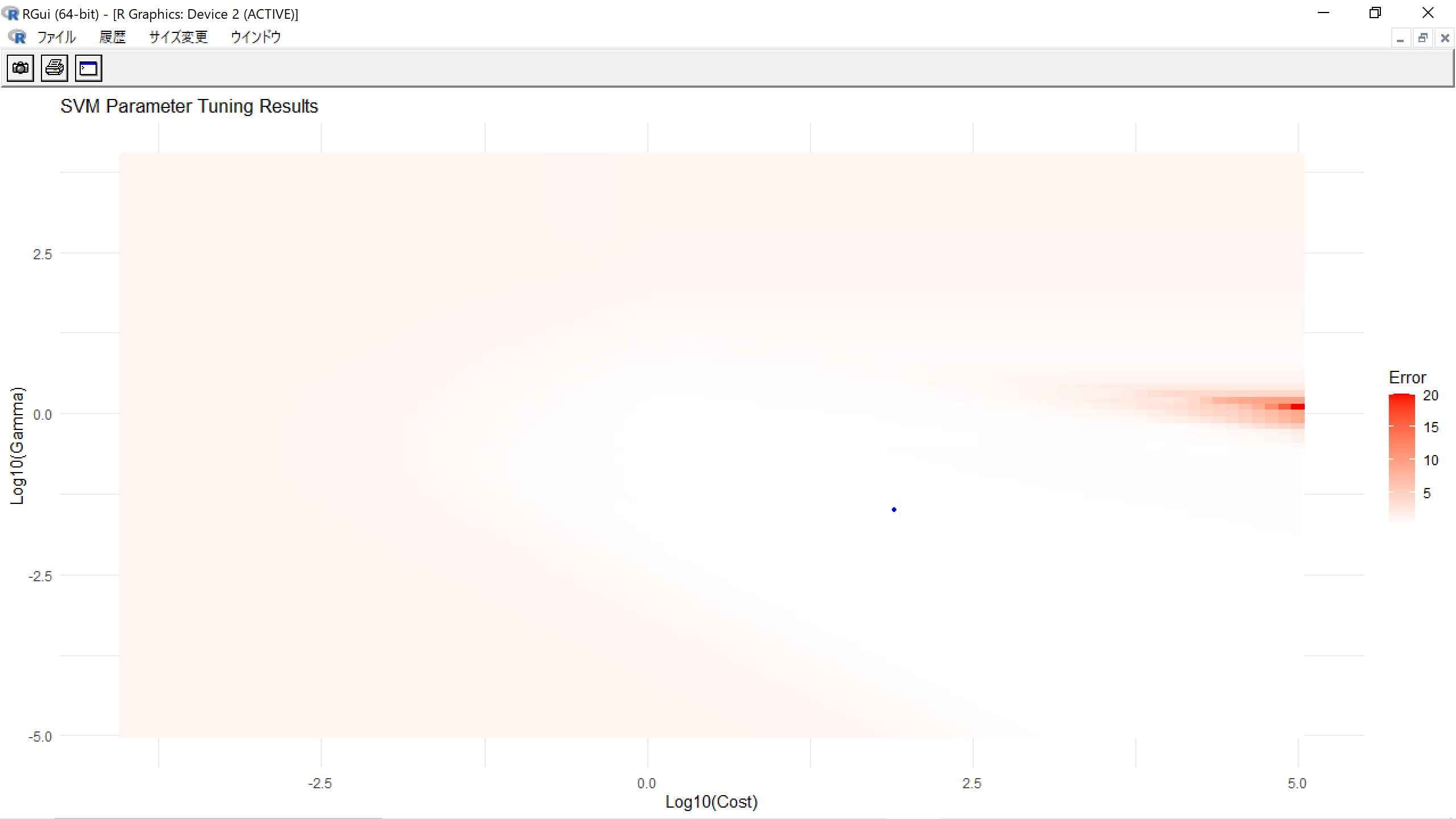


SVRM<-

svm(age~SLC12A5_3_methylation_rate_ave+SLC12A5_4_methylation_rate_ave,　data=ABBBS,

cost=best.cost, gamma=best.gamma, epsilon=0.1, scale = FALSE)

#LOOCV

nSamples<-nrow(ABBBS)

predict_SVRM_loocv<-numeric(nSamples)

for (z in 1:nSamples){

indices<-removeOne(nSamples,z)

dr<-data.frame(ABBBS$age[indices],ABBBS$SLC12A5_3_methylation_rate_ave[indices],ABBBS$SLC12A5_4_methylation_rate_ave[indices])

colnames(dr)<-c("age","methylslc_3","methylslc_4")

bestmodel_SVRM<-svm(age~methylslc_3+methylslc_4, data=dr,

cost=best.cost, gamma= best.gamma, epsilon=0.1, scale = FALSE)

newdata<-data.frame(methylslc_3=ABBBS$SLC12A5_3_methylation_rate_ave[z],methylslc_4=ABBBS$SLC12A5_4_methylation_rate_ave[z])

p<-predict(bestmodel_SVRM,newdata)*sd(AGE)+mean(AGE)

if (p<0){p=0}

predict_SVRM_loocv[z]<-p}

ABBB_SVRM_loocv<-cbind(ABBB,predict_SVRM_loocv)

MAE_SVRM_loocv<-mean(abs(ABBB_SVRM_loocv$predict_SVRM_loocv-ABBB$age))

MedianAE_SVRM_loocv<-median(abs(ABBB_SVRM_loocv$predict_SVRM_loocv-ABBB$age))

RMSE_SVRM_loocv<- sqrt(mean((ABBB_SVRM_loocv$predict_SVRM_loocv-ABBB$age)^2))

cat("MAE:", MAE_SVRM_loocv, "\nMed AE:", MedianAE_SVRM_loocv, "\nRMSE:", RMSE_SVRM_loocv, "\n")

MAE: 1.227047

Med AE: 0.8621172

RMSE: 1.50711

g_SVRM_loocv<-ggplot(ABBB_SVRM_loocv,aes(age,predict_SVRM_loocv))+theme_bw()+

annotate("segment",x=min(ABBB$age),xend=max(ABBB$age),y=min(ABBB$age)+1.227047,yend=max(ABBB$age)+1.227047,colour="orchid4",linetype=2,linewidth =0.7)+

annotate("segment",x= min(ABBB$age),xend=max(ABBB$age), y=min(ABBB$age)-1.227047,yend=max(ABBB$age)-1.227047,colour="orchid4",linetype=2,linewidth =0.7)+

geom_point(aes(shape=environment,color=sex),size=2,stroke=2)+

labs(x="Chronological age (year)",y="Predicted age (year)")+

scale_shape_manual(name="environment",labels=c("Captive"="captive","Wild"="wild"),values=c("Captive"=1, "Wild"=3))+

scale_color_manual(name="sex",labels=c("F"="female","M"="male"),values=c("F"="firebrick2","M"="dodgerblue4"))+

theme(axis.text.x=element_text(size=20),axis.text.y=element_text(size=20))+

theme(axis.title.x=element_text(size=17),axis.title.y=element_text(size=17))+

geom_line(aes(y =age), linewidth=1)+

labs(title="SVR model")+

theme(title=element_text(size=17),plot.title=element_text(hjust=0.5))+

scale_y_continuous(limits=c(-5,40))+

scale_x_continuous(limits=c(-5,40))+

labs(subtitle="SLC12A5-3, -4")+

theme(plot.subtitle=element_text(size=15,hjust=0.5))


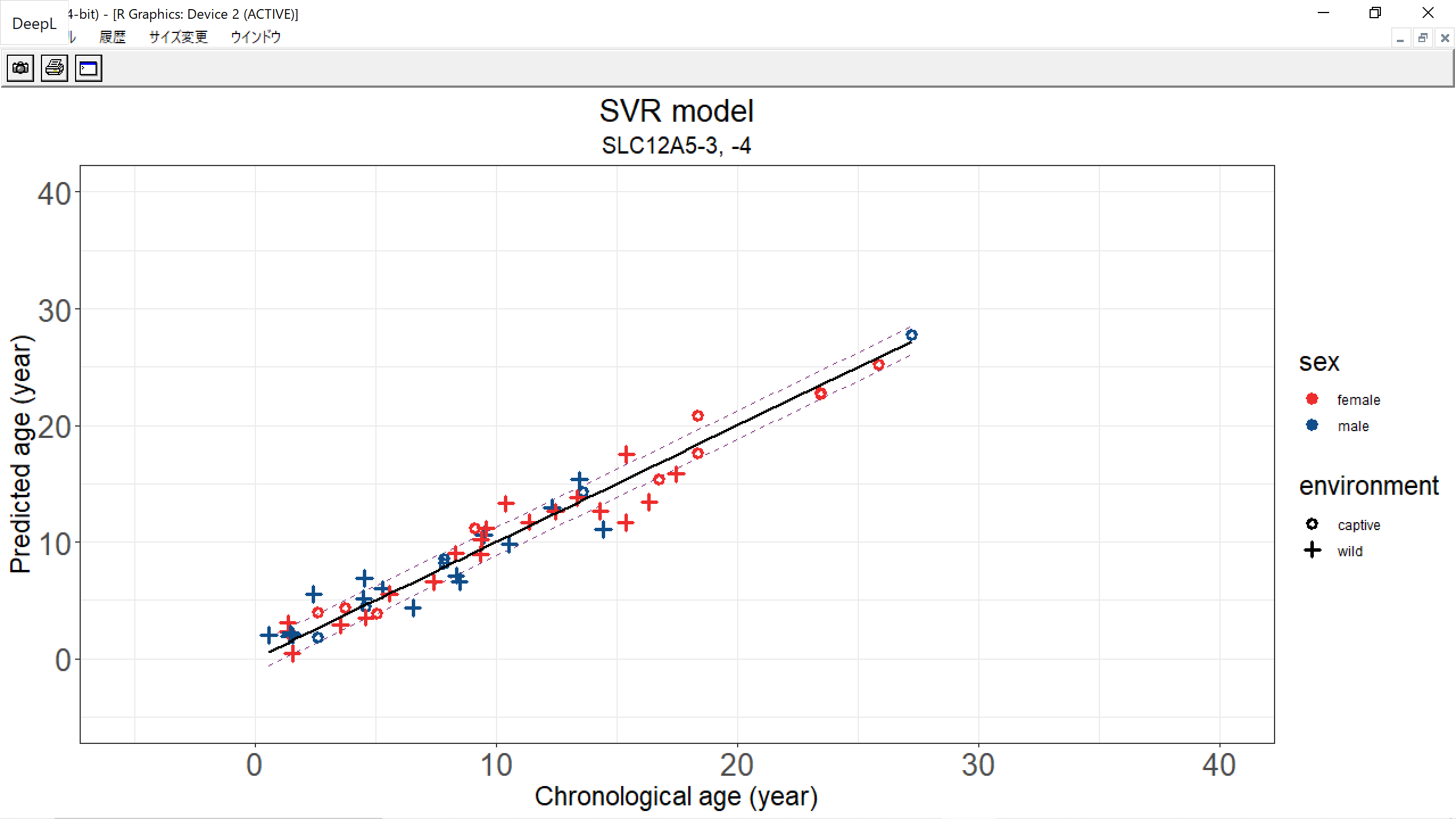


Influences of interaction among age, sex, and growth environment

Single regression (SLC12A5-4)

SRM_SLC12A5_4<-lm(formula=YS~slc4S,data=ABBBS)

predicted_age_SRM_SLC12A5_4_s <- predict(SRM_SLC12A5_4)

#Δage

deltaage_SRM_SLC12A5_4<- predicted_age_SRM_SLC12A5_4_s-ABBBS$age

modeldelta_SRM_SLC12A5_4<-lm(formula=deltaage_SRM_SLC12A5_4~ABBBS$age*ABBBS$sex*ABBBS$environment)

options(na.action="na.fail")

modellist_SRM_SLC12A5_4<-dredge(modeldelta_SRM_SLC12A5_4,rank="AIC")

bestmodel_SRM_SLC12A5_4<-get.models(dredge(modeldelta_SRM_SLC12A5_4,rank="AIC"),subset=1)

bestmodel_SRM_SLC12A5_4

$`2`

Call:

lm(formula = deltaage_SRM_SLC12A5_4 ~ ABBBS$age + 1)

Coefficients:

(Intercept) ABBBS$age

1.248e-12 -6.487e-02

attr(,"rank")

function (x)

do.call("rank", list(x))

<environment: 0x000001ac22df9098>

attr(,"call")

AIC(x)

attr(,"class")

[1] "function" "rankFunction"

attr(,"beta")

[1] "none"

summary(lm(formula = deltaage_SRM_SLC12A5_4 ~ ABBBS$age + 1))

Call:

lm(formula = deltaage_SRM_SLC12A5_4 ~ ABBBS$age + 1)

Residuals:

Min 1Q Median 3Q Max

-0.53976 -0.17030 -0.00859 0.18561 0.59677

Coefficients:

Estimate Std. Error t value Pr(>|t|)

(Intercept) 1.248e-12 3.450e-02 0.000 1.0000

ABBBS$age -6.487e-02 3.483e-02 -1.862 0.0684 .

---

Signif. codes: 0 ‘***’ 0.001 ‘**’ 0.01 ‘*’ 0.05 ‘.’ 0.1 ‘ ’ 1

Residual standard error: 0.2488 on 50 degrees of freedom

Multiple R-squared: 0.06487, Adjusted R-squared: 0.04617

F-statistic: 3.469 on 1 and 50 DF, p-value: 0.06842

#|Δage|

absdeltaage_SRM_SLC12A5_4<- abs(predicted_age_SRM_SLC12A5_4_s-ABBBS$age)

modelabsdelta_SRM_SLC12A5_4<-lm(formula=absdeltaage_SRM_SLC12A5_4~ABBBS$age*ABBBS$sex*ABBBS$environment)

options(na.action = "na.fail")

modellist_absSRM_SLC12A5_4<-dredge(modelabsdelta_SRM_SLC12A5_4,rank="AIC")

bestmodel_absSRM_SLC12A5_4<- get.models(dredge(modelabsdelta_SRM_SLC12A5_4,rank="AIC"),subset=1)

bestmodel_absSRM_SLC12A5_4

$`1`

Call:

lm(formula = absdeltaage_SRM_SLC12A5_4 ~ 1)

Coefficients:

(Intercept)

0.2039

attr(,"rank")

function (x)

do.call("rank", list(x))

<environment: 0x000001ac251eb7d8>

attr(,"call")

AIC(x)

attr(,"class")

[1] "function" "rankFunction"

attr(,"beta")

[1] "none"

Principal component regression (PC1)

ABBBS$SLC12A5_PC1 <- pca_slc$x[,1]

PCRM_SLC <- lm(formula = age ~ SLC12A5_PC1, data = ABBBS)

predicted_age_PCRM_SLC_s <- predict(PCRM_SLC)

#Δage

deltaage_PCRM_SLC<- predicted_age_PCRM_SLC_s-ABBBS$age

modeldelta_PCRM_SLC<-lm(formula=deltaage_PCRM_SLC~ABBBS$age*ABBBS$sex*ABBBS$environment)

options(na.action="na.fail")

modellist_PCRM_SLC<-dredge(modeldelta_PCRM_SLC,rank="AIC")

bestmodel_PCRM_SLC<-get.models(dredge(modeldelta_PCRM_SLC,rank="AIC"),subset=1)

bestmodel_PCRM_SLC

$`2`

Call:

lm(formula = deltaage_PCRM_SLC ~ ABBBS$age + 1)

Coefficients:

(Intercept) ABBBS$age

1.140e-12 -5.926e-02

attr(,"rank")

function (x)

do.call("rank", list(x))

<environment: 0x00000142440e5a50>

attr(,"call")

AIC(x)

attr(,"class")

[1] "function" "rankFunction"

attr(,"beta")

[1] "none" $`2`

Call:

lm(formula = deltaage_PCRM_SLC ~ ABBBS$age + 1)

Coefficients:

(Intercept) ABBBS$age

1.140e-12 -5.926e-02

attr(,"rank")

function (x)

do.call("rank", list(x))

<environment: 0x00000142440e5a50>

attr(,"call")

AIC(x)

attr(,"class")

[1] "function" "rankFunction"

attr(,"beta")

[1] "none"

summary(lm(formula = deltaage_PCRM_SLC ~ ABBBS$age + 1))

Call:

lm(formula = deltaage_PCRM_SLC ~ ABBBS$age + 1)

Residuals:

Min 1Q Median 3Q Max

-0.49790 -0.19442 -0.03396 0.13402 0.49181

Coefficients:

Estimate Std. Error t value Pr(>|t|)

(Intercept) 1.140e-12 3.307e-02 0.000 1.000

ABBBS$age -5.926e-02 3.339e-02 -1.775 0.082 .

---

Signif. codes: 0 ‘***’ 0.001 ‘**’ 0.01 ‘*’ 0.05 ‘.’ 0.1 ‘ ’ 1

Residual standard error: 0.2385 on 50 degrees of freedom

Multiple R-squared: 0.05926, Adjusted R-squared: 0.04045

F-statistic: 3.15 on 1 and 50 DF, p-value: 0.08202

#|Δage|

absdeltaage_PCRM_SLC<- abs(predicted_age_PCRM_SLC_s-ABBBS$age)

modelabsdelta_PCRM_SLC<-lm(formula=absdeltaage_PCRM_SLC~ABBBS$age*ABBBS$sex*ABBBS$environment)

options(na.action = "na.fail")

modellist_absPCRM_SLC<-dredge(modelabsdelta_PCRM_SLC,rank="AIC")

bestmodel_absPCRM_SLC<- get.models(dredge(modelabsdelta_PCRM_SLC,rank="AIC"),subset=1)

bestmodel_absPCRM_SLC

$`2`

Call:

lm(formula = absdeltaage_PCRM_SLC ~ ABBBS$age + 1)

Coefficients:

(Intercept) ABBBS$age

0.19074 0.04376

attr(,"rank")

function (x)

do.call("rank", list(x))

<environment: 0x00000142499dc958>

attr(,"call")

AIC(x)

attr(,"class")

[1] "function" "rankFunction"

attr(,"beta")

[1] "none"

summary(lm(formula = absdeltaage_PCRM_SLC ~ ABBBS$age + 1))

Call:

lm(formula = absdeltaage_PCRM_SLC ~ ABBBS$age + 1)

Residuals:

Min 1Q Median 3Q Max

-0.20510 -0.12250 -0.02401 0.07924 0.32016

Coefficients:

Estimate Std. Error t value Pr(>|t|)

(Intercept) 0.19074 0.01993 9.569 6.94e-13 ***

ABBBS$age 0.04376 0.02013 2.174 0.0345 *

---

Signif. codes: 0 ‘***’ 0.001 ‘**’ 0.01 ‘*’ 0.05 ‘.’ 0.1 ‘ ’ 1

Residual standard error: 0.1437 on 50 degrees of freedom

Multiple R-squared: 0.08635, Adjusted R-squared: 0.06808

F-statistic: 4.726 on 1 and 50 DF, p-value: 0.03448

Elastic net regression (SLC12A5-1, -2, -3, -4)

X <- cbind(slc1S,slc2S,slc3S,slc4S)

ENM4 <- glmnet(x = cbind(ABBBS$SLC12A5_1_methylation_rate_ave,ABBBS$SLC12A5_2_methylation_rate_ave,ABBBS$SLC12A5_3_methylation_rate_ave,ABBBS$SLC12A5_4_methylation_rate_ave),

y = ABBBS$age, family = "gaussian", lambda = 0.009484228, alpha = 0.01, standardize = FALSE)

predicted_age_s <- c(predict(ENM4, newx = X, s=0.009484228))

#Δage

deltaage_ENM4<- predicted_age_s-ABBBS$age

modeldelta_ENM4<-lm(formula =deltaage_ENM4~ABBBS$age*ABBBS$sex*ABBBS$environment)

options(na.action = "na.fail")

modellist_ENM4<-dredge(modeldelta_ENM4,rank="AIC")

bestmodel_ENM4<- get.models(dredge(modeldelta_ENM4,rank="AIC"),subset=1)

bestmodel_ENM4

$`2`

Call:

lm(formula = deltaage_ENM4 ~ ABBBS$age + 1)

Coefficients:

(Intercept) ABBBS$age

1.058e-12 -5.503e-02

attr(,"rank")

function (x)

do.call("rank", list(x))

<environment: 0x000001530f2924d0>

attr(,"call")

AIC(x)

attr(,"class")

[1] "function" "rankFunction"

attr(,"beta")

[1] "none"

summary(lm(formula = deltaage_ENM4 ~ ABBBS$age + 1))

Call:

lm(formula = deltaage_ENM4 ~ ABBBS$age + 1)

Residuals:

Min 1Q Median 3Q Max

-0.42193 -0.17430 0.03515 0.11775 0.46505

Coefficients:

Estimate Std. Error t value Pr(>|t|)

(Intercept) 1.058e-12 3.082e-02 0.000 1.0000

ABBBS$age -5.503e-02 3.112e-02 -1.768 0.0831 .

---

Signif. codes: 0 ‘***’ 0.001 ‘**’ 0.01 ‘*’ 0.05 ‘.’ 0.1 ‘ ’ 1

Residual standard error: 0.2222 on 50 degrees of freedom

Multiple R-squared: 0.05885, Adjusted R-squared: 0.04003

F-statistic: 3.127 on 1 and 50 DF, p-value: 0.08312

#|Δage|

absdeltaage_ENM4<- abs(predicted_age_s-ABBBS$age)

modeldelta_absENM4<-lm(formula =absdeltaage_ENM4~ABBBS$age*ABBBS$sex*ABBBS$environment)

options(na.action = "na.fail")

modellist_absENM4<-dredge(modeldelta_absENM4,rank="AIC")

bestmodel_absENM4<- get.models(dredge(modeldelta_absENM4,rank="AIC"),subset=1)

bestmodel_absENM4

$`1`

Call:

lm(formula = absdeltaage_ENM4 ~ 1)

Coefficients:

(Intercept)

0.1789

attr(,"rank")

function (x)

do.call("rank", list(x))

<environment: 0x0000015310aae310>

attr(,"call")

AIC(x)

attr(,"class")

[1] "function" "rankFunction"

attr(,"beta")

[1] "none"

Support vector regression ((SLC12A5-1, -2, -4)

SVRM3<-

svm(age~SLC12A5_1_methylation_rate_ave+SLC12A5_2_methylation_rate_ave+SLC12A5_4_methylation_rate_ave,　data=ABBBS,

cost=10^4.9,gamma=10^-3.1,epsilon=0.1, scale = FALSE)

predict_SVRM3_s <- predict(SVRM3)

#Δage

deltaage_SVRM3<- predict_SVRM3_s-ABBBS$age

modeldelta_SVRM3<-

lm(formula =deltaage_SVRM3~ABBBS$age*ABBBS$sex*ABBBS$environment)

options(na.action = "na.fail")

modellist_SVRM3<-dredge(modeldelta_SVRM3,rank="AIC")

bestmodel_SVRM3<- get.models(dredge(modeldelta_SVRM3,rank="AIC"),subset=1)

bestmodel_SVRM3

$`1`

Call:

lm(formula = deltaage_SVRM3 ~ 1)

Coefficients:

(Intercept)

0.0001824

attr(,"rank")

function (x)

do.call("rank", list(x))

<environment: 0x000001530f08fd98>

attr(,"call")

AIC(x)

attr(,"class")

[1] "function" "rankFunction"

attr(,"beta")

[1] "none"

#|Δage|

absdeltaage_SVRM3<- abs(predict_SVRM3_s-ABBBS$age)

modeldelta_absSVRM3<-

lm(formula =absdeltaage_SVRM3~ABBBS$age*ABBBS$sex*ABBBS$environment)

options(na.action = "na.fail")

modellist_absSVRM3<-dredge(modeldelta_absSVRM3,rank="AIC")

bestmodel_absSVRM3<- get.models(dredge(modeldelta_absSVRM3,rank="AIC"),subset=1)

bestmodel_absSVRM3

$`1`

Call:

lm(formula = absdeltaage_SVRM3 ~ 1)

Coefficients:

(Intercept)

0.153

attr(,"rank")

function (x)

do.call("rank", list(x))

<environment: 0x00000153157cf650>

attr(,"call")

AIC(x)

attr(,"class")

[1] "function" "rankFunction"

attr(,"beta")

[1] "none"

Support vector regression ((SLC12A5-3, -4)

SVRM2<-

svm(age~SLC12A5_3_methylation_rate_ave+SLC12A5_4_methylation_rate_ave,　data=ABBBS,

cost=10^1.9,gamma=10^-1.5,epsilon=0.1, scale = FALSE)

predict_SVRM2_s <- predict(SVRM2)

#Δage

deltaage_SVRM2<- predict_SVRM2_s-ABBBS$age

modeldelta_SVRM2<-

lm(formula =deltaage_SVRM2~ABBBS$age*ABBBS$sex*ABBBS$environment)

options(na.action = "na.fail")

modellist_SVRM2<-dredge(modeldelta_SVRM2,rank="AIC")

bestmodel_SVRM2<- get.models(dredge(modeldelta_SVRM2,rank="AIC"),subset=1)

bestmodel_SVRM2

$`2`

Call:

lm(formula = deltaage_SVRM2 ~ ABBBS$age + 1)

Coefficients:

(Intercept) ABBBS$age

0.01807 -0.05224

attr(,"rank")

function (x)

do.call("rank", list(x))

<environment: 0x0000015317c903c0>

attr(,"call")

AIC(x)

attr(,"class")

[1] "function" "rankFunction"

attr(,"beta")

[1] "none"

summary(lm(formula = deltaage_SVRM2 ~ ABBBS$age + 1))

Call:

lm(formula = deltaage_SVRM2 ~ ABBBS$age + 1)

Residuals:

Min 1Q Median 3Q Max

-0.50179 -0.15872 0.01717 0.10657 0.41394

Coefficients:

Estimate Std. Error t value Pr(>|t|)

(Intercept) 0.01807 0.02909 0.621 0.5372

ABBBS$age -0.05224 0.02937 -1.778 0.0814 .

---

Signif. codes: 0 ‘***’ 0.001 ‘**’ 0.01 ‘*’ 0.05 ‘.’ 0.1 ‘ ’ 1

Residual standard error: 0.2098 on 50 degrees of freedom

Multiple R-squared: 0.05949, Adjusted R-squared: 0.04068

F-statistic: 3.163 on 1 and 50 DF, p-value: 0.08141

#|Δage|

absdeltaage_SVRM2<- abs(predict_SVRM2_s-ABBBS$age)

modeldelta_absSVRM2<-

lm(formula =absdeltaage_SVRM2~ABBBS$age*ABBBS$sex*ABBBS$environment)

options(na.action = "na.fail")

modellist_absSVRM2<-dredge(modeldelta_absSVRM2,rank="AIC")

bestmodel_absSVRM2<- get.models(dredge(modeldelta_absSVRM2,rank="AIC"),subset=1)

bestmodel_absSVRM2

$`12`

Call:

lm(formula = absdeltaage_SVRM2 ~ ABBBS$age + ABBBS$environment +

ABBBS$age:ABBBS$environment + 1)

Coefficients:

(Intercept) ABBBS$age

0.1274313 -0.0005674

ABBBS$environmentWild ABBBS$age:ABBBS$environmentWild

0.0773522 0.0644139

attr(,"rank")

function (x)

do.call("rank", list(x))

<environment: 0x00000153122a2ad0>

attr(,"call")

AIC(x)

attr(,"class")

[1] "function" "rankFunction"

attr(,"beta")

[1] "none"

summary(lm(formula = absdeltaage_SVRM2 ~ ABBBS$age + ABBBS$environment +

ABBBS$age:ABBBS$environment + 1))

Call:

lm(formula = absdeltaage_SVRM2 ~ ABBBS$age + ABBBS$environment +

ABBBS$age:ABBBS$environment + 1)

Residuals:

Min 1Q Median 3Q Max

-0.20712 -0.06435 -0.02737 0.05069 0.30755

Coefficients:

Estimate Std. Error t value Pr(>|t|)

(Intercept) 0.1274313 0.0329002 3.873 0.000325 ***

ABBBS$age -0.0005674 0.0241845 -0.023 0.981379

ABBBS$environmentWild 0.0773522 0.0391782 1.974 0.054109 .

ABBBS$age:ABBBS$environmentWild 0.0644139 0.0366946 1.755 0.085570 .

---

Signif. codes: 0 ‘***’ 0.001 ‘**’ 0.01 ‘*’ 0.05 ‘.’ 0.1 ‘ ’ 1

Residual standard error: 0.122 on 48 degrees of freedom

Multiple R-squared: 0.1478, Adjusted R-squared: 0.09449

F-statistic: 2.774 on 3 and 48 DF, p-value: 0.05141

How to apply to the models

MD <- read.csv("measurement_data.csv")

MD[,2] <- (MD[,2] – 36.585) / 12.342

MD[,3] <- (MD[,3] – 32.920) / 12.203

MD[,4] <- (MD[,4] – 24.984) / 9.9268

MD[,5] <- (MD[,5] – 31.146) / 11.636

write.csv(MD, "measurement_data_standardized.csv", row.names = FALSE)

MDS <- read.csv("measurement_data_standardized.csv")

ABBBS <- read.csv("Asian_black_bear_blood_standardized.csv")

MEAN <- 9.6959

SD <- 6.7553

The file “measurement_data.csv” is a template file, available in Dryad (DOI: 10.5061/dryad.b5mkkwhqt). Write the sample IDs and measured methylation levels on it.

The “measurement_data_standardized.csv” file contains values of standardized methylation levels of “measurement_data.csv”.

The standardized values are calculated by the following equation.

“standardized value” = (“original value” − “mean of training data”) ÷ “standard deviation of training data”)

Single regression (SLC12A5-4)

SRM <-lm(formula=age~SLC12A5_4_methylation_rate_ave,data=ABBBS)

predicted_age_SRM <- predict(SRM,MDS)*SD+MEAN

predicted_age_SRM[predicted_age_SRM < 0] <- 0

data.frame(Sample_ID = MDS$Sample_ID, Predicted_Age = predicted_age_SRM)

Principal component regression (PC1)

pca_slc <- prcomp(ABBBS[, c("SLC12A5_1_methylation_rate_ave",

"SLC12A5_2_methylation_rate_ave",

"SLC12A5_3_methylation_rate_ave",

"SLC12A5_4_methylation_rate_ave")],

scale = FALSE)

ABBBS$SLC12A5_PC1 <- pca_slc$x[,1]

PCRM <- lm(formula = age ~ SLC12A5_PC1, data = ABBBS)

MDS$SLC12A5_PC1 <- as.numeric(as.matrix(MDS[,c("SLC12A5_1_methylation_rate_ave", "SLC12A5_2_methylation_rate_ave", "SLC12A5_3_methylation_rate_ave", "SLC12A5_4_methylation_rate_ave")]) %*% pca_slc$rotation[,1])

predicted_age_PCRM <- predict(PCRM,MDS)*SD+MEAN

predicted_age_PCRM[predicted_age_PCRM < 0] <- 0

data.frame(Sample_ID = MDS$Sample_ID, Predicted_Age = predicted_age_PCRM)

Elastic net regression (SLC12A5-1, -2, -3 -4)

library(glmnet)

ENM <- glmnet(x = cbind(ABBBS$SLC12A5_1_methylation_rate_ave,ABBBS$SLC12A5_2_methylation_rate_ave,ABBBS$SLC12A5_3_methylation_rate_ave,ABBBS$SLC12A5_4_methylation_rate_ave),

y = ABBBS$age, family = "gaussian", lambda = 0.009484228, alpha = 0.01, standardize = FALSE)

MDS_ENM <- cbind(MDS$SLC12A5_1_methylation_rate_ave, MDS$SLC12A5_2_methylation_rate_ave, MDS$SLC12A5_3_methylation_rate_ave, MDS$SLC12A5_4_methylation_rate_ave)

predicted_age_ENM <- c(predict(ENM,MDS_ENM,s= 0.009484228)*SD+MEAN)

predicted_age_ENM[predicted_age_ENM < 0] <- 0

data.frame(Sample_ID = MDS$Sample_ID, Predicted_Age = predicted_age_ENM)

Support vector regression (SLC12A5-1, -2, -4)

library(e1071)

SVRM<-

svm(age~SLC12A5_1_methylation_rate_ave+SLC12A5_2_methylation_rate_ave+SLC12A5_4_methylation_rate_ave,data=ABBBS, cost=10^4.9, gamma=10^-3.1, epsilon=0.1, scale = FALSE)

predicted_age_SVRM<-predict(SVRM,MDS)*SD+MEAN

predicted_age_SVRM[predicted_age_SVRM < 0] <- 0

data.frame(Sample_ID = MDS$Sample_ID, Predicted_Age = predicted_age_SVRM)

Output to a csv file

sample_ID <- MDS[, 1, drop = FALSE]

predicted_age<-cbind(sample_ID,predicted_age_SRM,predicted_age_PCRM,predicted_age_ENM,predicted_age_SVRM)

write.csv(predicted_age, "predicted_age_result.csv", row.names = FALSE)

At this point, two files, “measurement_data_standardized.csv” and “predicted_age_result.csv”, should have been generated. Leaving them as they are may cause errors in subsequent runs, so please either rename the files or move them to a different folder.
