# Supplementary material for: Epigenetic Clock in Bears: A Simple Cost‐Effective Blood DNA Methylation‐Based Age Estimation Method Applicable to Multiple Bear Species
Source: Ecol Evol. 2025 May 6;15(5):e71424. doi: 10.1002/ece3.71424 (PMC12055220; doi:10.1002/ece3.71424)
Supplement: Supplementary file 2 — Appendix S2. [file ECE3-15-e71424-s003.docx]

Supplementary file_R script_Pan-bear

**A Simple Cost-Effective Blood DNA Methylation-Based Age Estimation Method Applicable to Multiple Bear Species**

Michito Shimozuru, Shiori Nakamura, Jumpei Yamazaki, Yojiro Yanagawa, Hiroo Tamatani, Misako Kuroe, Koji Yamazaki, Shinsuke Koike, Yusuke Goto, Tomoko Naganuma, Kahoko Tochigi, Akino Inagaki, Naoki Takekoshi, Seungyun Baek, Nobutaka Sato, Yusuke Honda, Toshio Tsubota, Hideyuki Ito

- [Load in packages](#Load_in_packages)
- [Data input](#Data_input)
- [The correlation between methylation level and age](#The_correlation_between_methyltion_level)
- [Age estimation model 【Single regression】](#Age_estimation_model_【Single_regression】)
  - [Single regression (SLC12A5-1)](#S_1)
  - [Single regression (SLC12A5-2)](#S_2)
  - [Single regression (SLC12A5-3)](#S_3)
  - [Single regression (SLC12A5-4)](#S_4)
- [Age estimation model 【Principal component regression (PC1)】](#Age_estimation_model_【PCR】)
  - [Principal component regression (PC1)](#PC1)
- [Age estimation model 【Elastic net regression】](#Age_estimation_model_【Elastic_net_regres)

- - [Elastic net regression (SLC12A5-1, -2, -3, -4)](#E_1234)
- [Age estimation model 【Support vector regression】](#Age_estimation_model_【Suport_vector_regr)
  - [Support vector regression (SLC12A5-1, -2, -3, -4)](#SVR_1234)
  - [Support vector regression (SLC12A5-1, -2, -3)](#SVR_123)
  - [Support vector regression (SLC12A5-1, -2, -4)](#SVR_124)
  - [Support vector regression (SLC12A5-1, -3, -4)](#SVR_134)
  - [Support vector regression (SLC12A5-2, -3, -4)](#SVR_234)
  - [Support vector regression (SLC12A5-1, -2)](#SVR_12)
  - [Support vector regression (SLC12A5-1, -3)](#SVR_13)
  - [Support vector regression (SLC12A5-1, -4)](#SVR_14)
  - [Support vector regression (SLC12A5-2, -3)](#SVR_23)
  - [Support vector regression (SLC12A5-2, -4)](#SVR_24)
  - [Support vector regression (SLC12A5-3, -4)](#SVR_34)
- [Influences of interaction among age, sex, and growth environment](#Influences_of_interaction_among_age_sex)
  - [Single regression (SLC12A5-4)](#I_S)
  - [Principal component regression (PC1)](#I_PC1)
  - [Elastic net regression (SLC12A5-1, -2, -3, -4)](#I_E_124)
  - [Support vector regression (SLC12A5-1, -2, -4)](#I_SVR_134)
  - [Support vector regression (SLC12A5-1, -3, -4)](#I_SVR_24)
- [How to apply to the models](#How_to_apply_to_the_models)
  - [Single regression (SLC12A5-4)](#H_S)
  - [Principal component regression (PC1)](#H_PC1)
  - [Elastic net regression (SLC12A5-1, -2, -3, -4)](#H_E)
  - [Support vector regression (SLC12A5-1, -3, -4)](#H_SVR)
  - [Output to a csv file](#Output_to_a_csv_file)

Load in packages

library(dplyr)

library(MuMIn)

library(glmnet)

library(e1071)

library(ggplot2)

library(car)

Data input

IBB<-read.csv("integrate_bear_blood.csv")

IBBS<-read.csv("integrate_bear_blood_standardized.csv")

The correlation between methylation level and age

#SLC12A5-1

cor.test(IBB$age,IBB$SLC12A5_1_methylation_rate_ave)

Pearson's product-moment correlation

data: IBB$age and IBB$SLC12A5_1_methylation_rate_ave

t = 33.467, df = 128, p-value < 2.2e-16

alternative hypothesis: true correlation is not equal to 0

95 percent confidence interval:

0.9262311 0.9625156

sample estimates:

cor

0.9473327

SLC1_ss<-ggplot(IBB,aes(x=age,y=SLC12A5_1_methylation_rate_ave))+theme_bw()+

geom_point(aes(shape=sex,color=species),size=2,stroke=2)+

labs(x="Age (year)",y="DNA methylation (%)")+

scale_shape_manual(name="sex",labels=c("F" = "female", "M" = "male"),values=c("F" = 1, "M" = 3))+

scale_color_manual(name="species",labels=c("ABB" = "Asian black bear", "PB" = "Polar bear", "BB" = "Brown bear", "SB" = "Sun bear"), values = c("ABB" = "#66C2A5", "PB" = "#8DA0CB", "BB" = "#FC8D62", "SB" = "#E78AC3"))+

theme(axis.text.x=element_text(size=20),axis.text.y=element_text(size=20))+

theme(axis.title.x=element_text(size=17),axis.title.y=element_text(size=17))+

annotate("text",size=6,x=-Inf,y=Inf,hjust=-.1,vjust=2,label="R=0.95, p<0.001")+

labs (title="SLC12A5-1")+

theme(plot.title=element_text(size=20,hjust = 0.5))+

guides(color = guide_legend(order = 1), shape = guide_legend(order = 2))


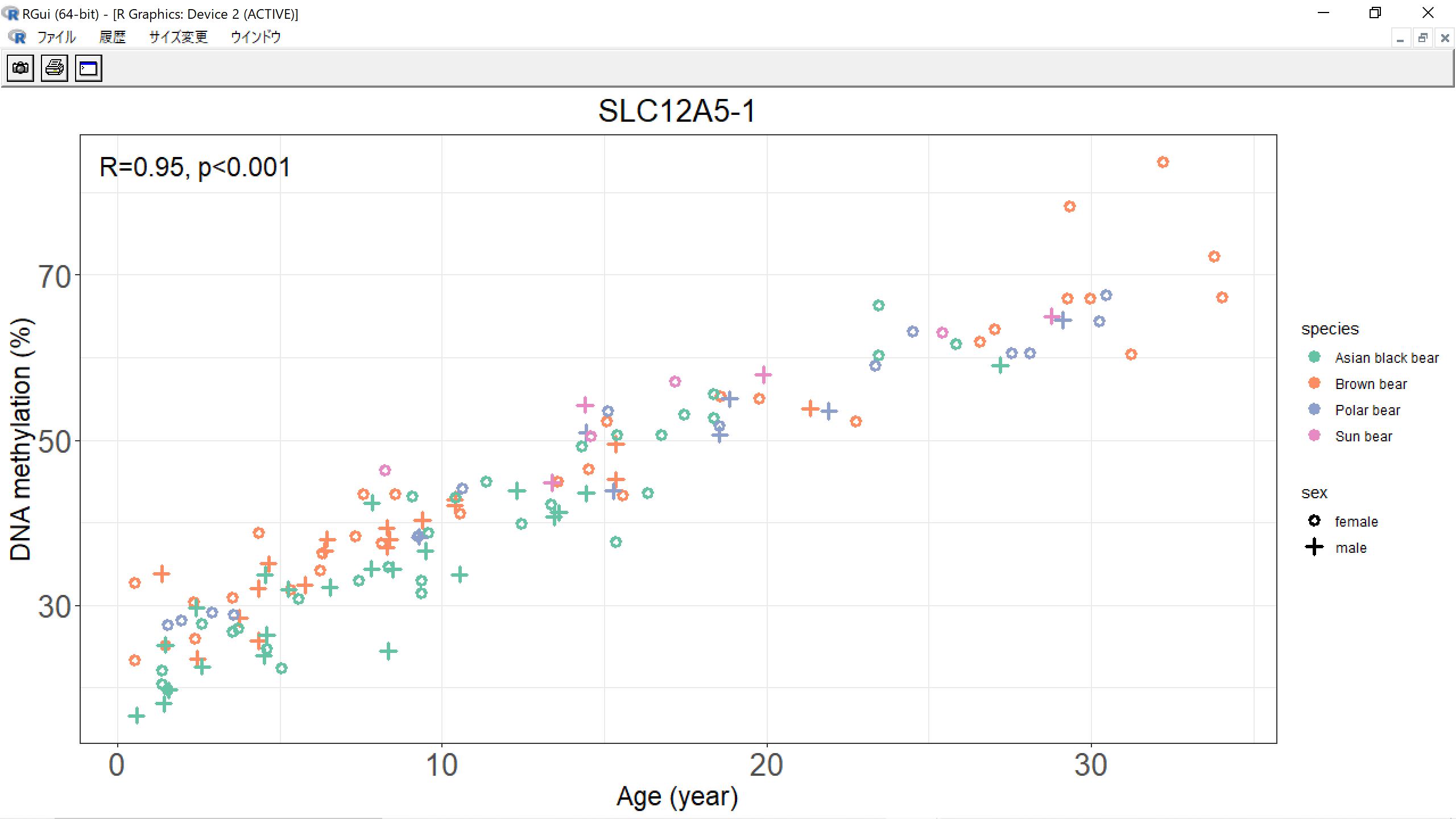


SLC1_se<-ggplot(IBB,aes(x=age,y=SLC12A5_1_methylation_rate_ave))+theme_bw()+

geom_point(aes(shape=environment,color=species),size=2,stroke=2)+

labs(x="Age (year)",y="DNA methylation (%)")+

scale_shape_manual(name="environment",labels=c("captive", "wild"),values=c(1,3))+

scale_color_manual(name="species",labels=c("ABB" = "Asian black bear", "PB" = "Polar bear", "BB" = "Brown bear", "SB" = "Sun bear"), values = c("ABB" = "#66C2A5", "PB" = "#8DA0CB", "BB" = "#FC8D62", "SB" = "#E78AC3"))+

theme(axis.text.x=element_text(size=20),axis.text.y=element_text(size=20))+

theme(axis.title.x=element_text(size=17),axis.title.y=element_text(size=17))+

annotate("text",size=6,x=-Inf,y=Inf,hjust=-.1,vjust=2,label="R=0.95, p<0.001")+

labs (title="SLC12A5-1")+

theme(plot.title=element_text(size=20,hjust = 0.5))+

guides(color = guide_legend(order = 1), shape = guide_legend(order = 2))


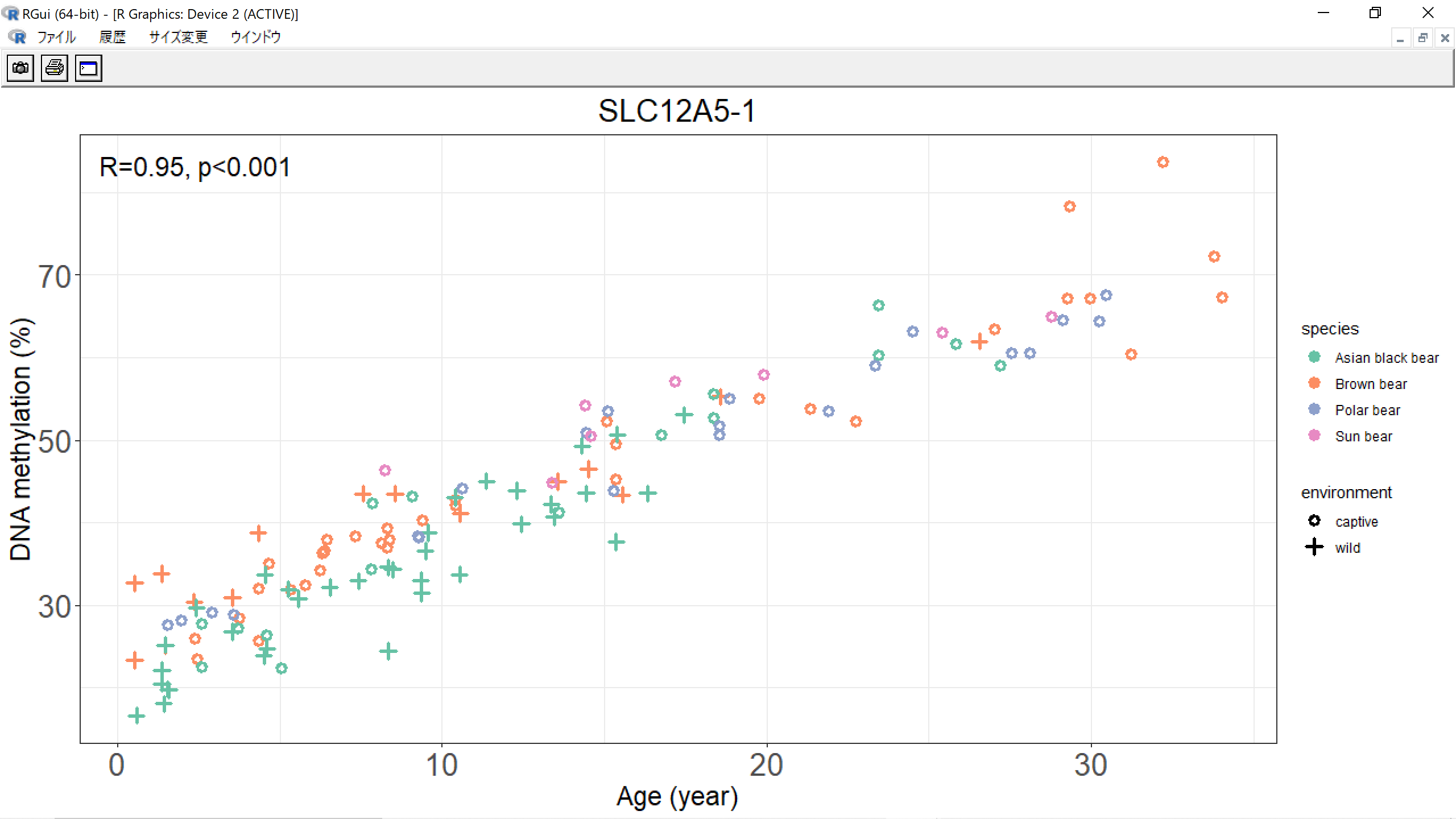


#SLC12A5-2

cor.test(IBB$age,IBB$SLC12A5_2_methylation_rate_ave)

Pearson's product-moment correlation

data: IBB$age and IBB$SLC12A5_2_methylation_rate_ave

t = 39.873, df = 128, p-value < 2.2e-16

alternative hypothesis: true correlation is not equal to 0

95 percent confidence interval:

0.9466449 0.9730291

sample estimates:

cor

0.9620223

SLC2_ss<-ggplot(IBB,aes(x=age,y=SLC12A5_2_methylation_rate_ave))+theme_bw()+

geom_point(aes(shape=sex,color=species),size=2,stroke=2)+

labs(x="Age (year)",y="DNA methylation (%)")+

scale_shape_manual(name="sex",labels=c("F" = "female", "M" = "male"),values=c("F" = 1, "M" = 3))+

scale_color_manual(name="species",labels=c("ABB" = "Asian black bear", "PB" = "Polar bear", "BB" = "Brown bear", "SB" = "Sun bear"), values = c("ABB" = "#66C2A5", "PB" = "#8DA0CB", "BB" = "#FC8D62", "SB" = "#E78AC3"))+

theme(axis.text.x=element_text(size=20),axis.text.y=element_text(size=20))+

theme(axis.title.x=element_text(size=17),axis.title.y=element_text(size=17))+

annotate("text",size=6,x=-Inf,y=Inf,hjust=-.1,vjust=2,label="R=0.96, p<0.001")+

labs (title="SLC12A5-2")+

theme(plot.title=element_text(size=20,hjust = 0.5))+

guides(color = guide_legend(order = 1), shape = guide_legend(order = 2))


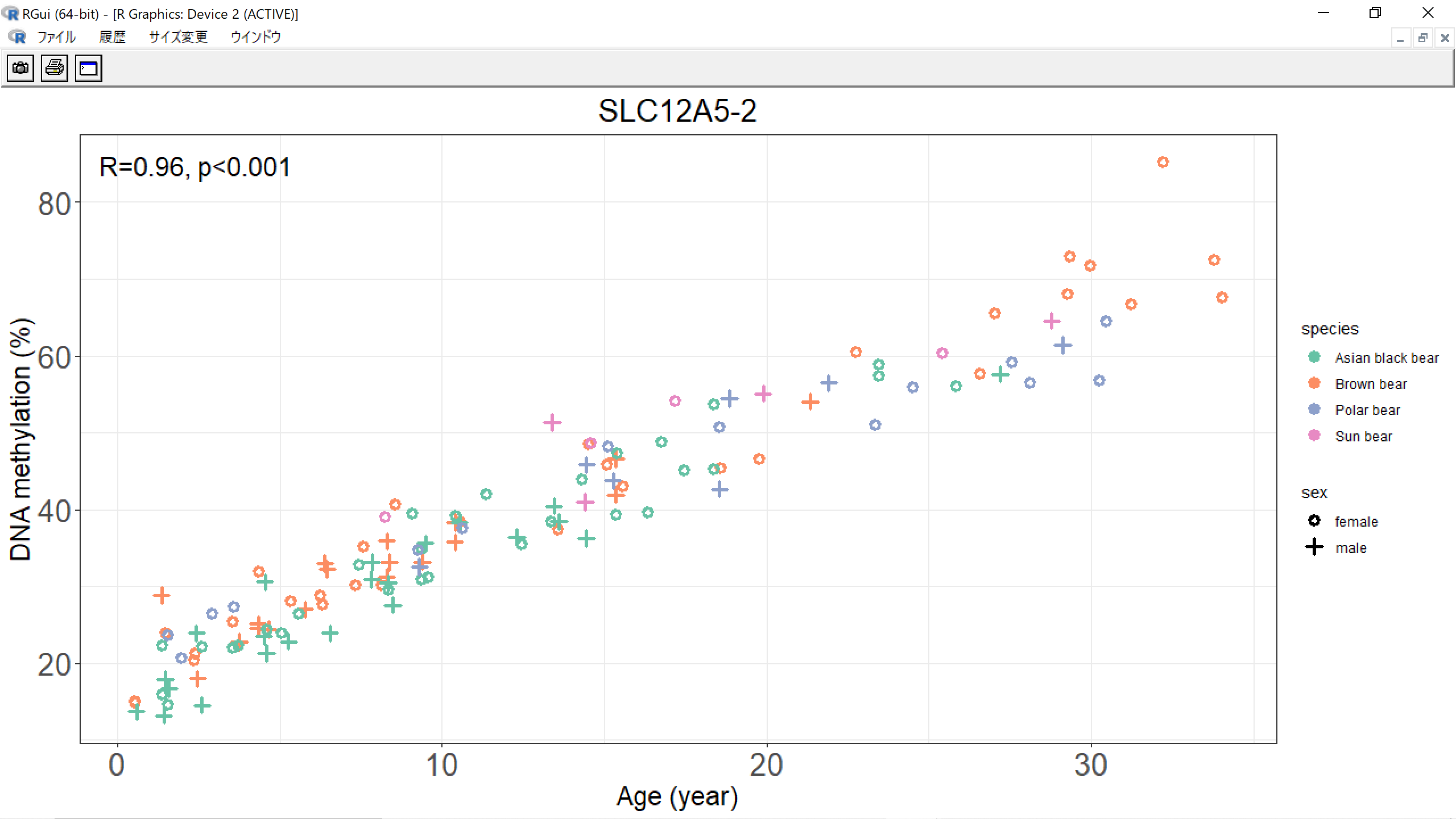


SLC2_se<-ggplot(IBB,aes(x=age,y=SLC12A5_2_methylation_rate_ave))+theme_bw()+

geom_point(aes(shape=environment,color=species),size=2,stroke=2)+

labs(x="Age (year)",y="DNA methylation (%)")+

scale_shape_manual(name="environment",labels=c("captive", "wild"),values=c(1,3))+

scale_color_manual(name="species",labels=c("ABB" = "Asian black bear", "PB" = "Polar bear", "BB" = "Brown bear", "SB" = "Sun bear"), values = c("ABB" = "#66C2A5", "PB" = "#8DA0CB", "BB" = "#FC8D62", "SB" = "#E78AC3"))+

theme(axis.text.x=element_text(size=20),axis.text.y=element_text(size=20))+

theme(axis.title.x=element_text(size=17),axis.title.y=element_text(size=17))+

annotate("text",size=6,x=-Inf,y=Inf,hjust=-.1,vjust=2,label="R=0.96, p<0.001")+

labs (title="SLC12A5-2")+

theme(plot.title=element_text(size=20,hjust = 0.5))+

guides(color = guide_legend(order = 1), shape = guide_legend(order = 2))


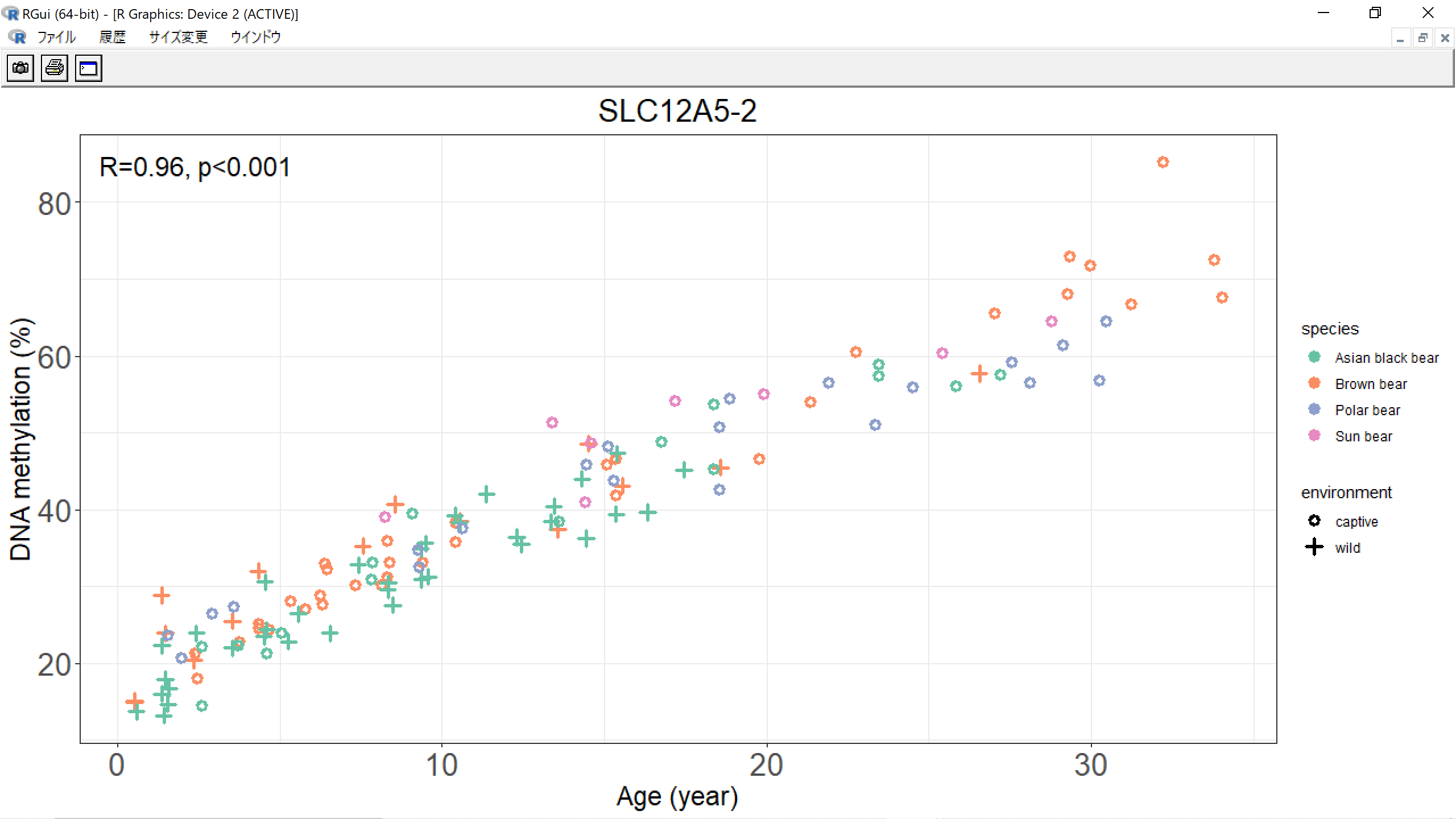


#SLC12A5-3

cor.test(IBB$age,IBB$SLC12A5_3_methylation_rate_ave)

Pearson's product-moment correlation

data: IBB$age and IBB$SLC12A5_3_methylation_rate_ave

t = 36.414, df = 128, p-value < 2.2e-16

alternative hypothesis: true correlation is not equal to 0

95 percent confidence interval:

0.9368284 0.9679871

sample estimates:

cor

0.9549695

SLC3_ss<-ggplot(IBB,aes(x=age,y=SLC12A5_3_methylation_rate_ave))+theme_bw()+

geom_point(aes(shape=sex,color=species),size=2,stroke=2)+

labs(x="Age (year)",y="DNA methylation (%)")+

scale_shape_manual(name="sex",labels=c("F" = "female", "M" = "male"),values=c("F" = 1, "M" = 3))+

scale_color_manual(name="species",labels=c("ABB" = "Asian black bear", "PB" = "Polar bear", "BB" = "Brown bear", "SB" = "Sun bear"), values = c("ABB" = "#66C2A5", "PB" = "#8DA0CB", "BB" = "#FC8D62", "SB" = "#E78AC3"))+

theme(axis.text.x=element_text(size=20),axis.text.y=element_text(size=20))+

theme(axis.title.x=element_text(size=17),axis.title.y=element_text(size=17))+

annotate("text",size=6,x=-Inf,y=Inf,hjust=-.1,vjust=2,label="R=0.95, p<0.001")+

labs (title="SLC12A5-3")+

theme(plot.title=element_text(size=20,hjust = 0.5))+

guides(color = guide_legend(order = 1), shape = guide_legend(order = 2))


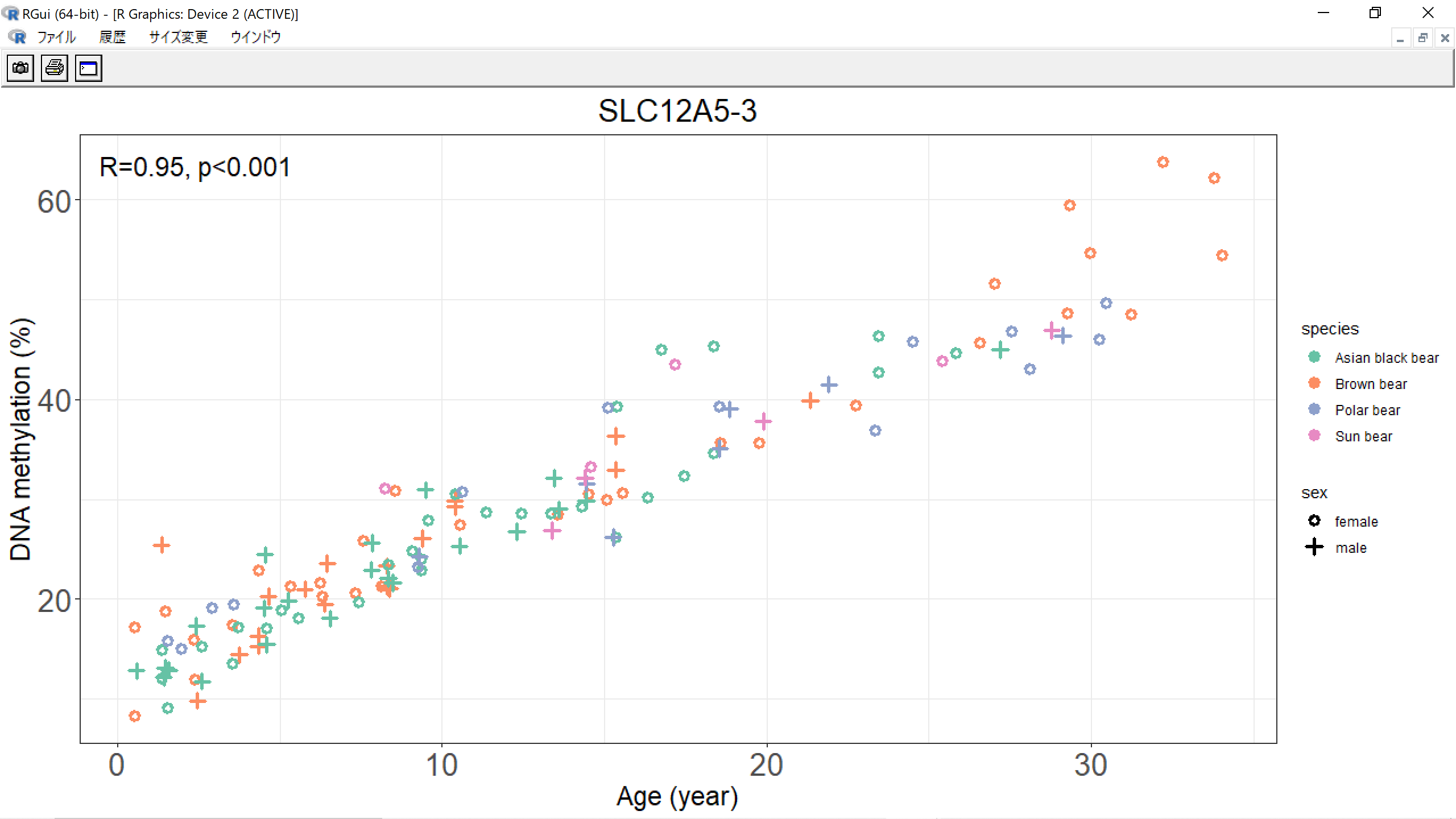


SLC3_se<-ggplot(IBB,aes(x=age,y=SLC12A5_3_methylation_rate_ave))+theme_bw()+

geom_point(aes(shape=environment,color=species),size=2,stroke=2)+

labs(x="Age (year)",y="DNA methylation (%)")+

scale_shape_manual(name="environment",labels=c("captive", "wild"),values=c(1,3))+

scale_color_manual(name="species",labels=c("ABB" = "Asian black bear", "PB" = "Polar bear", "BB" = "Brown bear", "SB" = "Sun bear"), values = c("ABB" = "#66C2A5", "PB" = "#8DA0CB", "BB" = "#FC8D62", "SB" = "#E78AC3"))+

theme(axis.text.x=element_text(size=20),axis.text.y=element_text(size=20))+

theme(axis.title.x=element_text(size=17),axis.title.y=element_text(size=17))+

annotate("text",size=6,x=-Inf,y=Inf,hjust=-.1,vjust=2,label="R=0.95, p<0.001")+

labs (title="SLC12A5-3")+

theme(plot.title=element_text(size=20,hjust = 0.5))+

guides(color = guide_legend(order = 1), shape = guide_legend(order = 2))


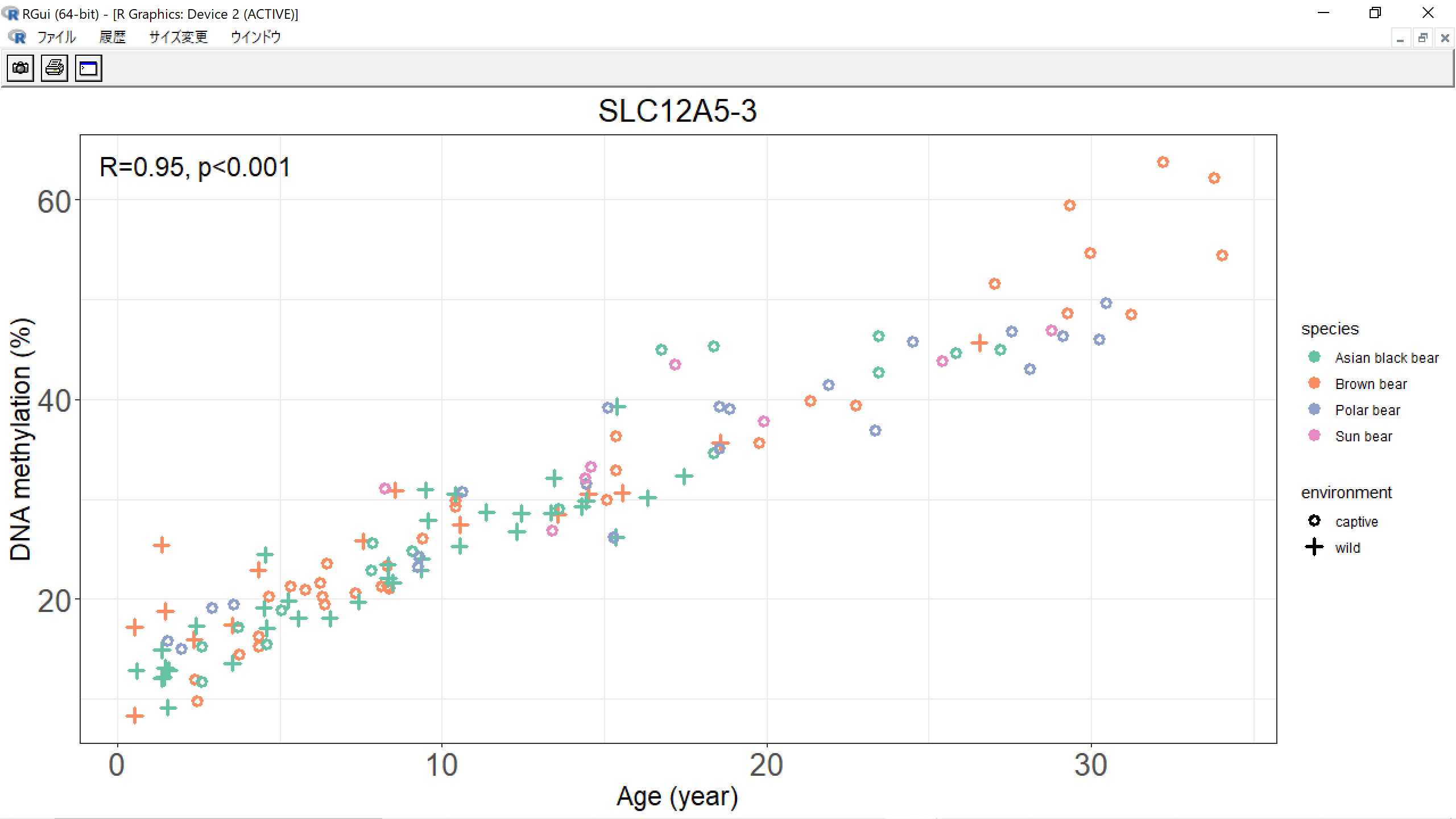


#SLC12A5-4

cor.test(IBB$age,IBB$SLC12A5_4_methylation_rate_ave)

Pearson's product-moment correlation

data: IBB$age and IBB$SLC12A5_4_methylation_rate_ave

t = 46.441, df = 128, p-value < 2.2e-16

alternative hypothesis: true correlation is not equal to 0

95 percent confidence interval:

0.9600009 0.9798489

sample estimates:

cor

0.9715851

SLC4_ss<-ggplot(IBB,aes(x=age,y=SLC12A5_4_methylation_rate_ave))+theme_bw()+

geom_point(aes(shape=sex,color=species),size=2,stroke=2)+

labs(x="Age (year)",y="DNA methylation (%)")+

scale_shape_manual(name="sex",labels=c("F" = "female", "M" = "male"),values=c("F" = 1, "M" = 3))+

scale_color_manual(name="species",labels=c("ABB" = "Asian black bear", "PB" = "Polar bear", "BB" = "Brown bear", "SB" = "Sun bear"), values = c("ABB" = "#66C2A5", "PB" = "#8DA0CB", "BB" = "#FC8D62", "SB" = "#E78AC3"))+

theme(axis.text.x=element_text(size=20),axis.text.y=element_text(size=20))+

theme(axis.title.x=element_text(size=17),axis.title.y=element_text(size=17))+

annotate("text",size=6,x=-Inf,y=Inf,hjust=-.1,vjust=2,label="R=0.97, p<0.001")+

labs (title="SLC12A5-4")+

theme(plot.title=element_text(size=20,hjust = 0.5))+

guides(color = guide_legend(order = 1), shape = guide_legend(order = 2))


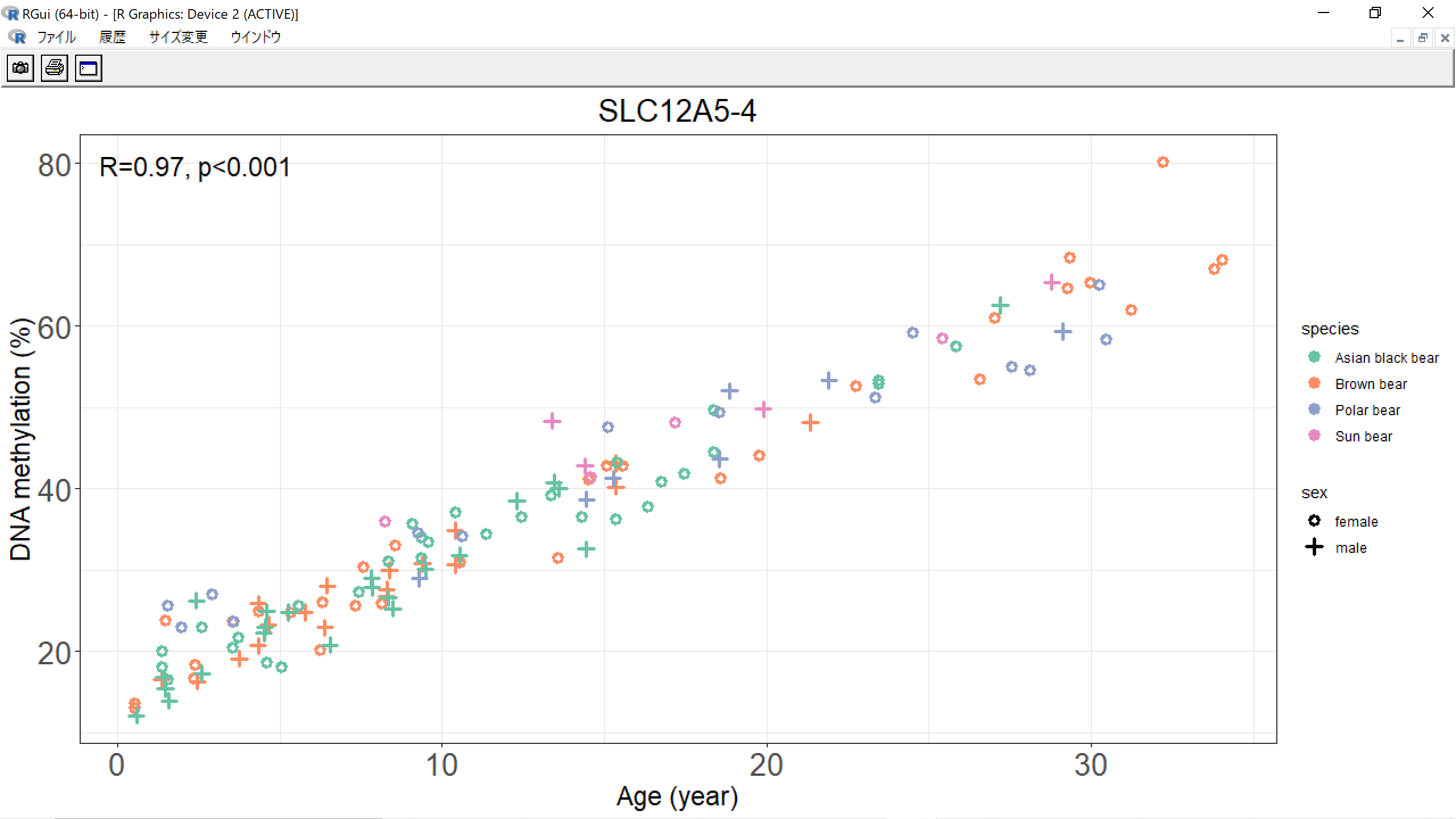


SLC4_se<-ggplot(IBB,aes(x=age,y=SLC12A5_4_methylation_rate_ave))+theme_bw()+

geom_point(aes(shape=environment,color=species),size=2,stroke=2)+

labs(x="Age (year)",y="DNA methylation (%)")+

scale_shape_manual(name="environment",labels=c("captive", "wild"),values=c(1,3))+

scale_color_manual(name="species",labels=c("ABB" = "Asian black bear", "PB" = "Polar bear", "BB" = "Brown bear", "SB" = "Sun bear"), values = c("ABB" = "#66C2A5", "PB" = "#8DA0CB", "BB" = "#FC8D62", "SB" = "#E78AC3"))+

theme(axis.text.x=element_text(size=20),axis.text.y=element_text(size=20))+

theme(axis.title.x=element_text(size=17),axis.title.y=element_text(size=17))+

annotate("text",size=6,x=-Inf,y=Inf,hjust=-.1,vjust=2,label="R=0.97, p<0.001")+

labs (title="SLC12A5-4")+

theme(plot.title=element_text(size=20,hjust = 0.5))+

guides(color = guide_legend(order = 1), shape = guide_legend(order = 2))


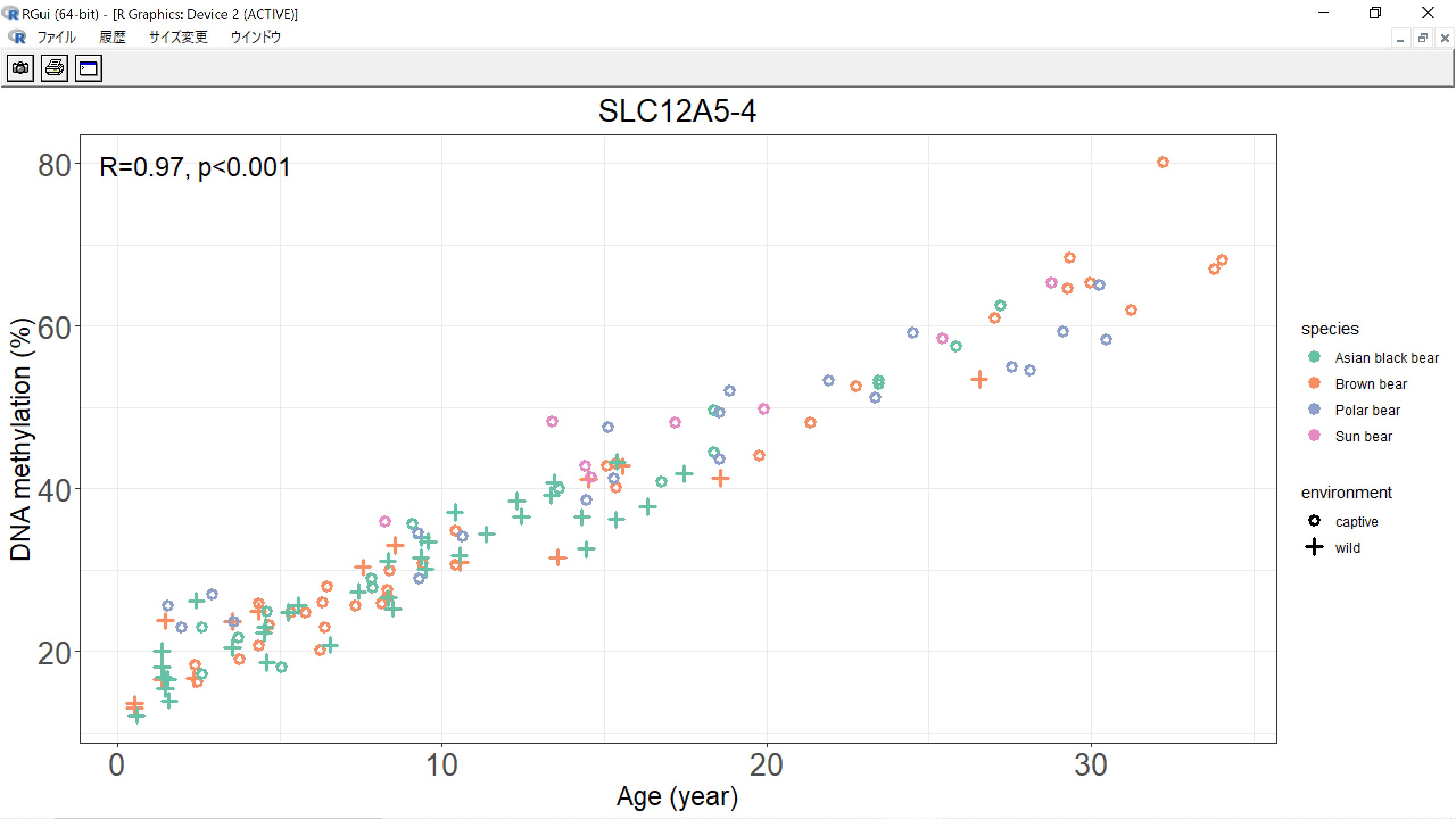


Age estimation model 【Single regression】

YS <- IBBS$age

slc1S <- IBBS$SLC12A5_1_methylation_rate_ave

slc2S <- IBBS$SLC12A5_2_methylation_rate_ave

slc3S <- IBBS$SLC12A5_3_methylation_rate_ave

slc4S <- IBBS$SLC12A5_4_methylation_rate_ave

AGE <- IBB$age

#RemoveOne function

removeOne <- function(dat,x) {

if(x<dat){

list=seq(1,dat)

x1=x-1;x2=x+1

v1=c(list[0:x1]);v2=c(list[x2:dat])

data=c(v1,v2)}

else {data=seq(1,dat-1)}

return (data)}

Single regression (SLC12A5-1)

SRM_SLC_1<-lm(formula=YS~slc1S,data=IBBS)

coef(SRM_SLC_1)

(Intercept) slc1S

-8.103596e-13 9.473327e-01

#LOOCV

nSamples<-nrow(IBBS)

predict_SRM_SLC_1_loocv<-numeric(nSamples)

for (z in 1:nSamples){

indices<-removeOne(nSamples,z)

dr<-data.frame(IBBS$age[indices],IBBS$SLC12A5_1_methylation_rate_ave[indices])

colnames(dr)<-c("age","methylslc_1")

bestmodel_SRM_SLC_1_loocv<-lm(age~methylslc_1,data=dr)

newdata<-data.frame(methylslc_1=IBBS$SLC12A5_1_methylation_rate_ave[z])

p<-predict(bestmodel_SRM_SLC_1_loocv,newdata)*sd(AGE)+mean(AGE)

if (p<0){p=0}

predict_SRM_SLC_1_loocv[z]<-p}

IBB_predict_SRM_SLC_1_loocv<-cbind(IBB,predict_SRM_SLC_1_loocv)

MAE_SRM_SLC_1_loocv<-mean(abs(IBB_predict_SRM_SLC_1_loocv$predict_SRM_SLC_1_loocv-IBB_predict_SRM_SLC_1_loocv$age))

MedianAE_SRM_SLC_1_loocv<-median(abs(IBB_predict_SRM_SLC_1_loocv$predict_SRM_SLC_1_loocv-IBB_predict_SRM_SLC_1_loocv$age))

RMSE_SRM_SLC_1_loocv<-sqrt(mean((IBB_predict_SRM_SLC_1_loocv$predict_SRM_SLC_1_loocv-IBB_predict_SRM_SLC_1_loocv$age)^2))

cat("MAE:", MAE_SRM_SLC_1_loocv, "\nMed AE:", MedianAE_SRM_SLC_1_loocv, "\nRMSE:", RMSE_SRM_SLC_1_loocv, "\n")

MAE: 2.294431

Med AE: 1.959464

RMSE: 2.872834

g_SRM_SLC_1_loocv_ss<-ggplot(IBB_predict_SRM_SLC_1_loocv,aes(age,predict_SRM_SLC_1_loocv))+theme_bw()+

annotate("segment",x=min(IBB$age),xend=max(IBB$age),y=min(IBB$age)+2.294431,yend=max(IBB$age)+2.294431,colour="orchid4",linetype=2, linewidth =0.7)+

annotate("segment",x=min(IBB$age),xend=max(IBB$age),y=min(IBB$age)-2.294431,yend=max(IBB$age)-2.294431,colour="orchid4",linetype=2, linewidth =0.7)+

geom_point(aes(shape=sex,color=species),size=2,stroke=2)+

labs(x="Chronological age (year)",y="Predicted age (year)")+

scale_shape_manual(name="sex",labels=c("F" = "female", "M" = "male"),values=c("F" = 1, "M" = 3))+

scale_color_manual(name="species",labels=c("ABB" = "Asian black bear", "PB" = "Polar bear", "BB" = "Brown bear", "SB" = "Sun bear"), values = c("ABB" = "#66C2A5", "PB" = "#8DA0CB", "BB" = "#FC8D62", "SB" = "#E78AC3"))+

theme(axis.text.x=element_text(size=20),axis.text.y=element_text(size=20))+

theme(axis.title.x=element_text(size=17),axis.title.y=element_text(size=17))+

geom_line(aes(y =age), linewidth=1)+

labs(title="single regression model")+

theme(title=element_text(size=17),plot.title=element_text(hjust=0.5))+

scale_y_continuous(limits=c(-5,40))+

scale_x_continuous(limits=c(-5,40))+

labs(subtitle="SLC12A5-1")+

theme(plot.subtitle=element_text(size=15,hjust=0.5))+

guides(color = guide_legend(order = 1), shape = guide_legend(order = 2))


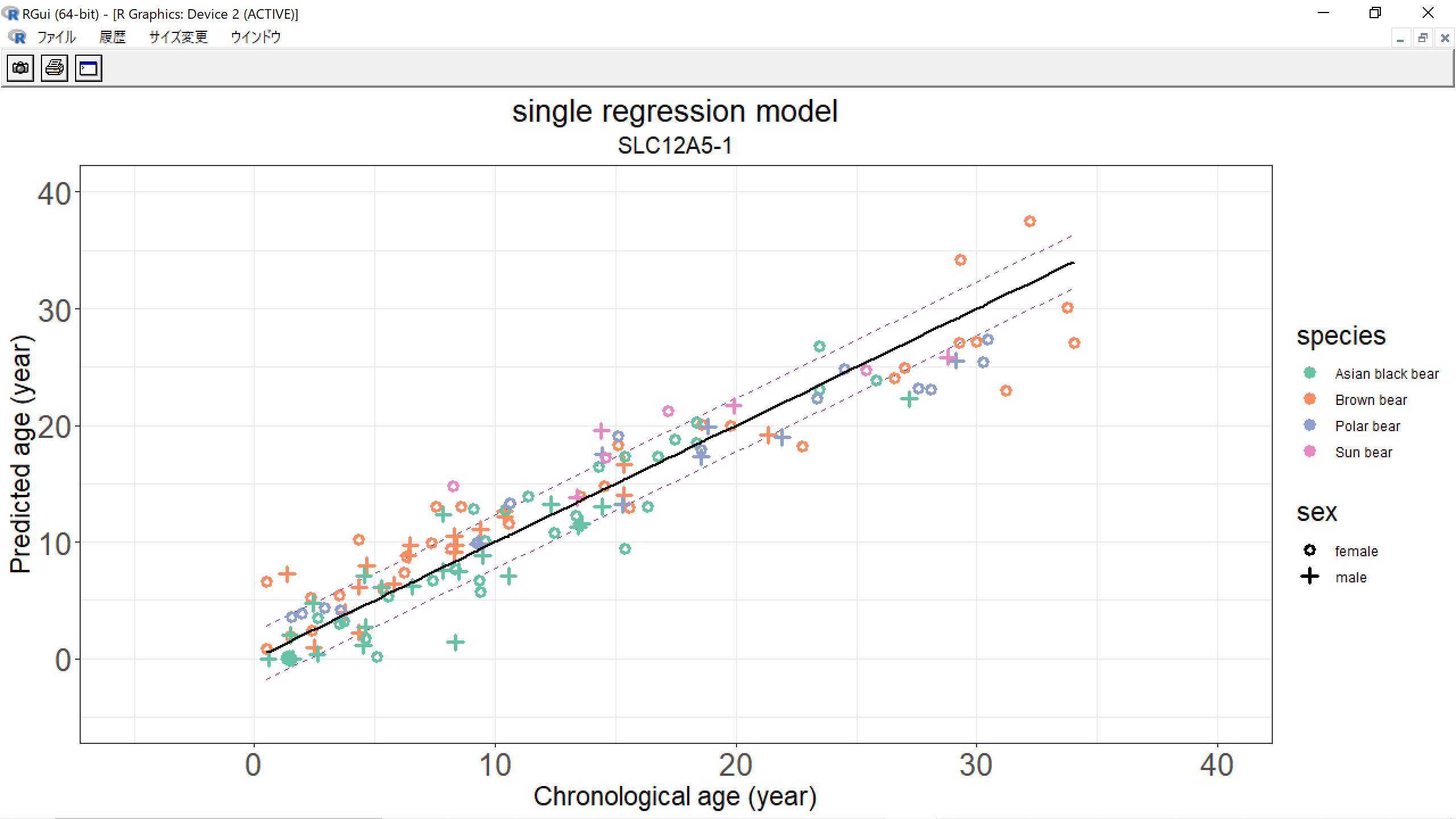


g_SRM_SLC_1_loocv_se<-ggplot(IBB_predict_SRM_SLC_1_loocv,aes(age,predict_SRM_SLC_1_loocv))+theme_bw()+

annotate("segment",x=min(IBB$age),xend=max(IBB$age),y=min(IBB$age)+2.294431,yend=max(IBB$age)+2.294431,colour="orchid4",linetype=2, linewidth =0.7)+

annotate("segment",x=min(IBB$age),xend=max(IBB$age),y=min(IBB$age)-2.294431,yend=max(IBB$age)-2.294431,colour="orchid4",linetype=2, linewidth =0.7)+

geom_point(aes(shape=environment,color=species),size=2,stroke=2)+

labs(x="Chronological age (year)",y="Predicted age (year)")+

scale_shape_manual(name="environment",labels=c("captive", "wild"),values=c(1,3))+

scale_color_manual(name="species",labels=c("ABB" = "Asian black bear", "PB" = "Polar bear", "BB" = "Brown bear", "SB" = "Sun bear"), values = c("ABB" = "#66C2A5", "PB" = "#8DA0CB", "BB" = "#FC8D62", "SB" = "#E78AC3"))+

theme(axis.text.x=element_text(size=20),axis.text.y=element_text(size=20))+

theme(axis.title.x=element_text(size=17),axis.title.y=element_text(size=17))+

geom_line(aes(y =age), linewidth=1)+

labs(title="single regression model")+

theme(title=element_text(size=17),plot.title=element_text(hjust=0.5))+

scale_y_continuous(limits=c(-5,40))+

scale_x_continuous(limits=c(-5,40))+

labs(subtitle="SLC12A5-1")+

theme(plot.subtitle=element_text(size=15,hjust=0.5))+

guides(color = guide_legend(order = 1), shape = guide_legend(order = 2))


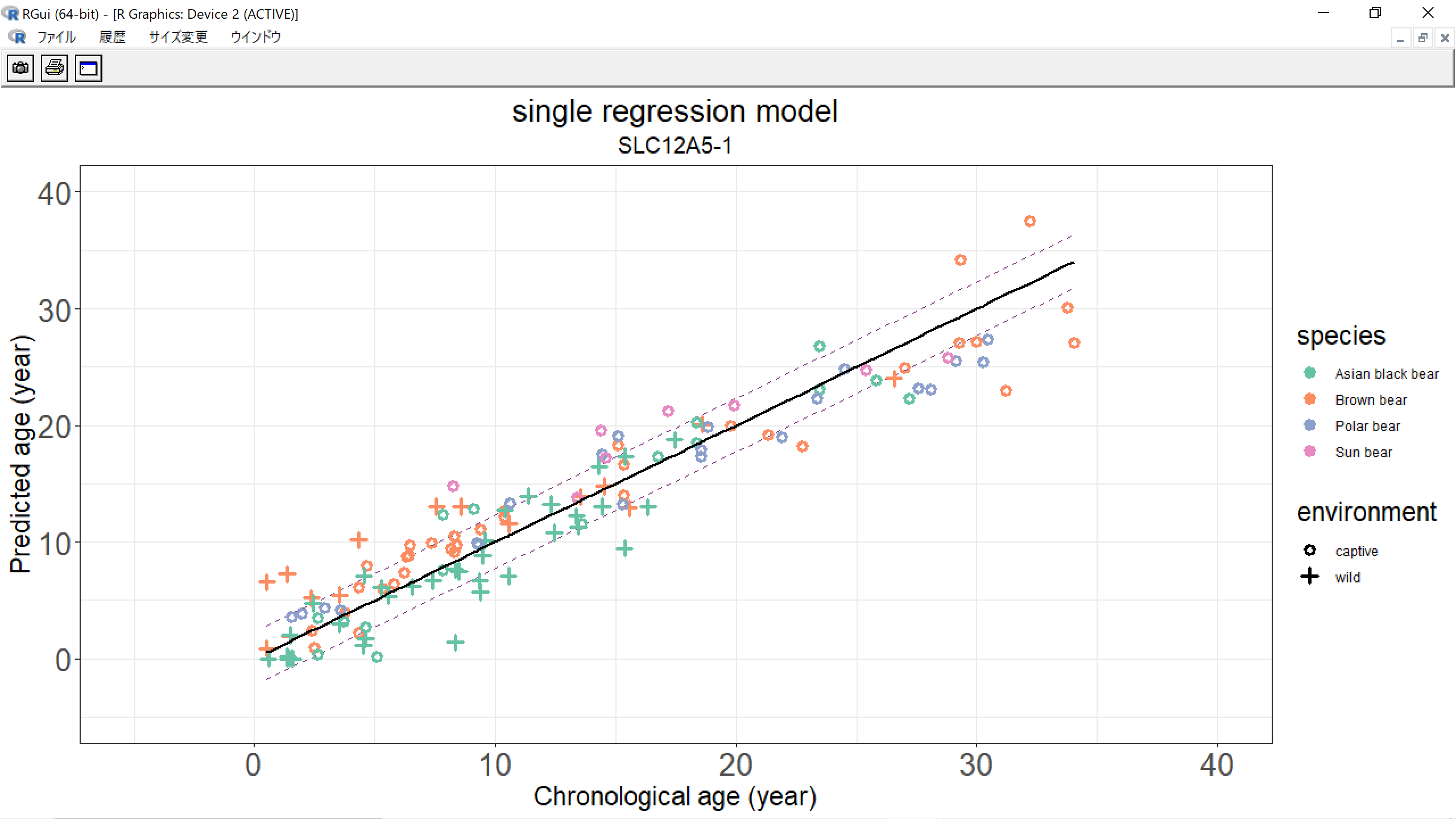


Single regression (SLC12A5-2)

SRM_SLC_2<-lm(formula=YS~slc2S,data=IBBS)

coef(SRM_SLC_2)

(Intercept) slc2S

-7.984542e-12 9.620223e-01

#LOOCV

nSamples<-nrow(IBBS)

predict_SRM_SLC_2_loocv<-numeric(nSamples)

for (z in 1:nSamples){

indices<-removeOne(nSamples,z)

dr<-data.frame(IBBS$age[indices],IBBS$SLC12A5_2_methylation_rate_ave[indices])

colnames(dr)<-c("age","methylslc_2")

bestmodel_SRM_SLC_2_loocv<-lm(age~methylslc_2,data=dr)

newdata<-data.frame(methylslc_2=IBBS$SLC12A5_2_methylation_rate_ave[z])

p<-predict(bestmodel_SRM_SLC_2_loocv,newdata)*sd(AGE)+mean(AGE)

if (p<0){p=0}

predict_SRM_SLC_2_loocv[z]<-p}

IBB_predict_SRM_SLC_2_loocv<-cbind(IBB,predict_SRM_SLC_2_loocv)

MAE_SRM_SLC_2_loocv<-mean(abs(IBB_predict_SRM_SLC_2_loocv$predict_SRM_SLC_2_loocv-IBB_predict_SRM_SLC_2_loocv$age))

MedianAE_SRM_SLC_2_loocv<-median(abs(IBB_predict_SRM_SLC_2_loocv$predict_SRM_SLC_2_loocv-IBB_predict_SRM_SLC_2_loocv$age))

RMSE_SRM_SLC_2_loocv<-sqrt(mean((IBB_predict_SRM_SLC_2_loocv$predict_SRM_SLC_2_loocv-IBB_predict_SRM_SLC_2_loocv$age)^2))

cat("MAE:", MAE_SRM_SLC_2_loocv, "\nMed AE:", MedianAE_SRM_SLC_2_loocv, "\nRMSE:", RMSE_SRM_SLC_2_loocv, "\n")

MAE: 1.898385

Med AE: 1.588622

RMSE: 2.444901

g_SRM_SLC_2_loocv_ss<-ggplot(IBB_predict_SRM_SLC_2_loocv,aes(age,predict_SRM_SLC_2_loocv))+theme_bw()+

annotate("segment",x=min(IBB$age),xend=max(IBB$age),y=min(IBB$age)+1.898385,yend=max(IBB$age)+1.898385,colour="orchid4",linetype=2, linewidth =0.7)+

annotate("segment",x=min(IBB$age),xend=max(IBB$age),y=min(IBB$age)-1.898385,yend=max(IBB$age)-1.898385,colour="orchid4",linetype=2, linewidth =0.7)+

geom_point(aes(shape=sex,color=species),size=2,stroke=2)+

labs(x="Chronological age (year)",y="Predicted age (year)")+

scale_shape_manual(name="sex",labels=c("F" = "female", "M" = "male"),values=c("F" = 1, "M" = 3))+

scale_color_manual(name="species",labels=c("ABB" = "Asian black bear", "PB" = "Polar bear", "BB" = "Brown bear", "SB" = "Sun bear"), values = c("ABB" = "#66C2A5", "PB" = "#8DA0CB", "BB" = "#FC8D62", "SB" = "#E78AC3"))+

theme(axis.text.x=element_text(size=20),axis.text.y=element_text(size=20))+

theme(axis.title.x=element_text(size=17),axis.title.y=element_text(size=17))+

geom_line(aes(y =age), linewidth=1)+

labs(title="single regression model")+

theme(title=element_text(size=17),plot.title=element_text(hjust=0.5))+

scale_y_continuous(limits=c(-5,40))+

scale_x_continuous(limits=c(-5,40))+

labs(subtitle="SLC12A5-2")+

theme(plot.subtitle=element_text(size=15,hjust=0.5))+

guides(color = guide_legend(order = 1), shape = guide_legend(order = 2))


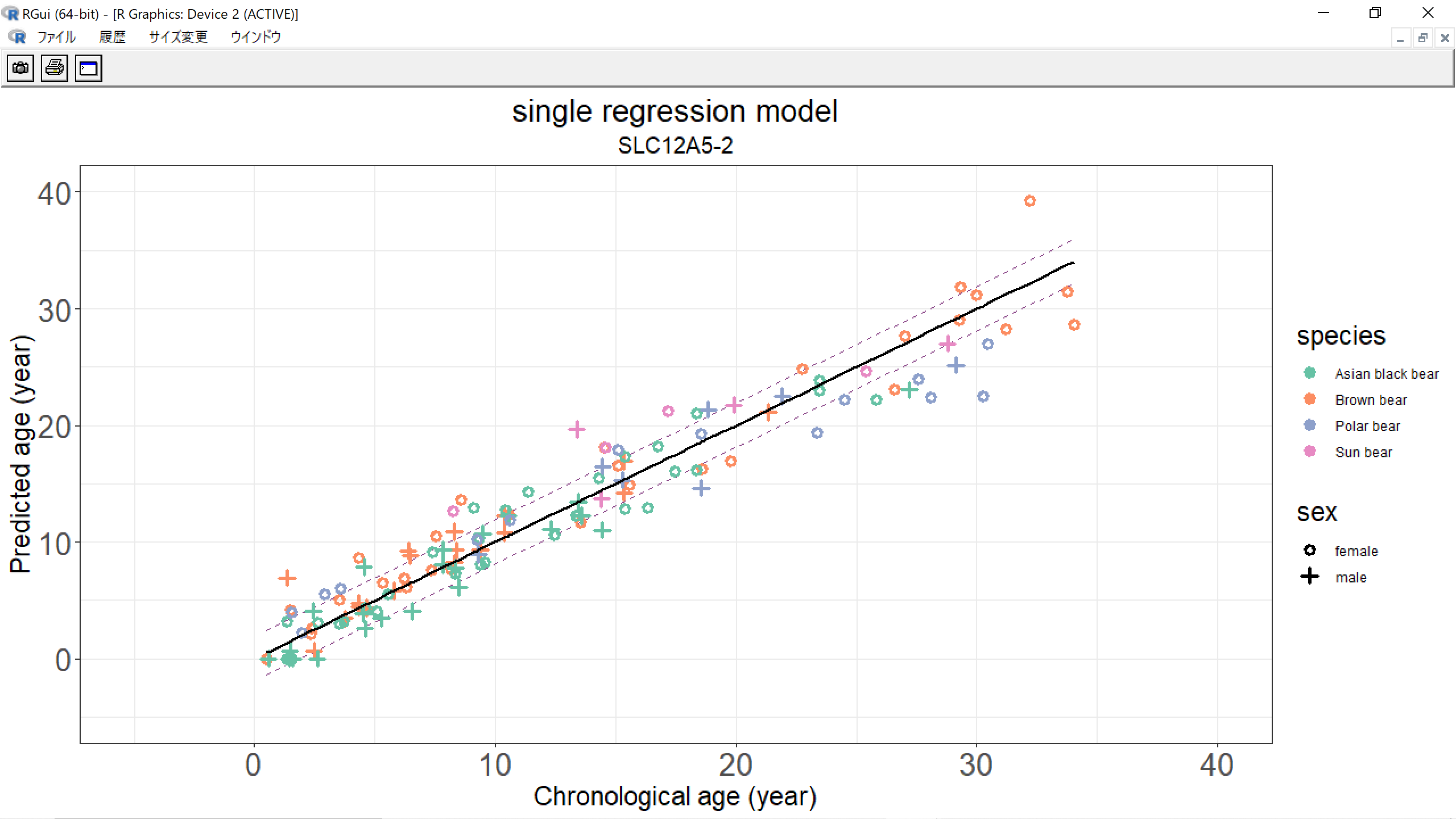


g_SRM_SLC_2_loocv_se<-ggplot(IBB_predict_SRM_SLC_2_loocv,aes(age,predict_SRM_SLC_2_loocv))+theme_bw()+

annotate("segment",x=min(IBB$age),xend=max(IBB$age),y=min(IBB$age)+1.898385,yend=max(IBB$age)+1.898385,colour="orchid4",linetype=2, linewidth =0.7)+

annotate("segment",x=min(IBB$age),xend=max(IBB$age),y=min(IBB$age)-1.898385,yend=max(IBB$age)-1.898385,colour="orchid4",linetype=2, linewidth =0.7)+

geom_point(aes(shape=environment,color=species),size=2,stroke=2)+

labs(x="Chronological age (year)",y="Predicted age (year)")+

scale_shape_manual(name="environment",labels=c("captive", "wild"),values=c(1,3))+

scale_color_manual(name="species",labels=c("ABB" = "Asian black bear", "PB" = "Polar bear", "BB" = "Brown bear", "SB" = "Sun bear"), values = c("ABB" = "#66C2A5", "PB" = "#8DA0CB", "BB" = "#FC8D62", "SB" = "#E78AC3"))+

theme(axis.text.x=element_text(size=20),axis.text.y=element_text(size=20))+

theme(axis.title.x=element_text(size=17),axis.title.y=element_text(size=17))+

geom_line(aes(y =age), linewidth=1)+

labs(title="single regression model")+

theme(title=element_text(size=17),plot.title=element_text(hjust=0.5))+

scale_y_continuous(limits=c(-5,40))+

scale_x_continuous(limits=c(-5,40))+

labs(subtitle="SLC12A5-2")+

theme(plot.subtitle=element_text(size=15,hjust=0.5))+

guides(color = guide_legend(order = 1), shape = guide_legend(order = 2))


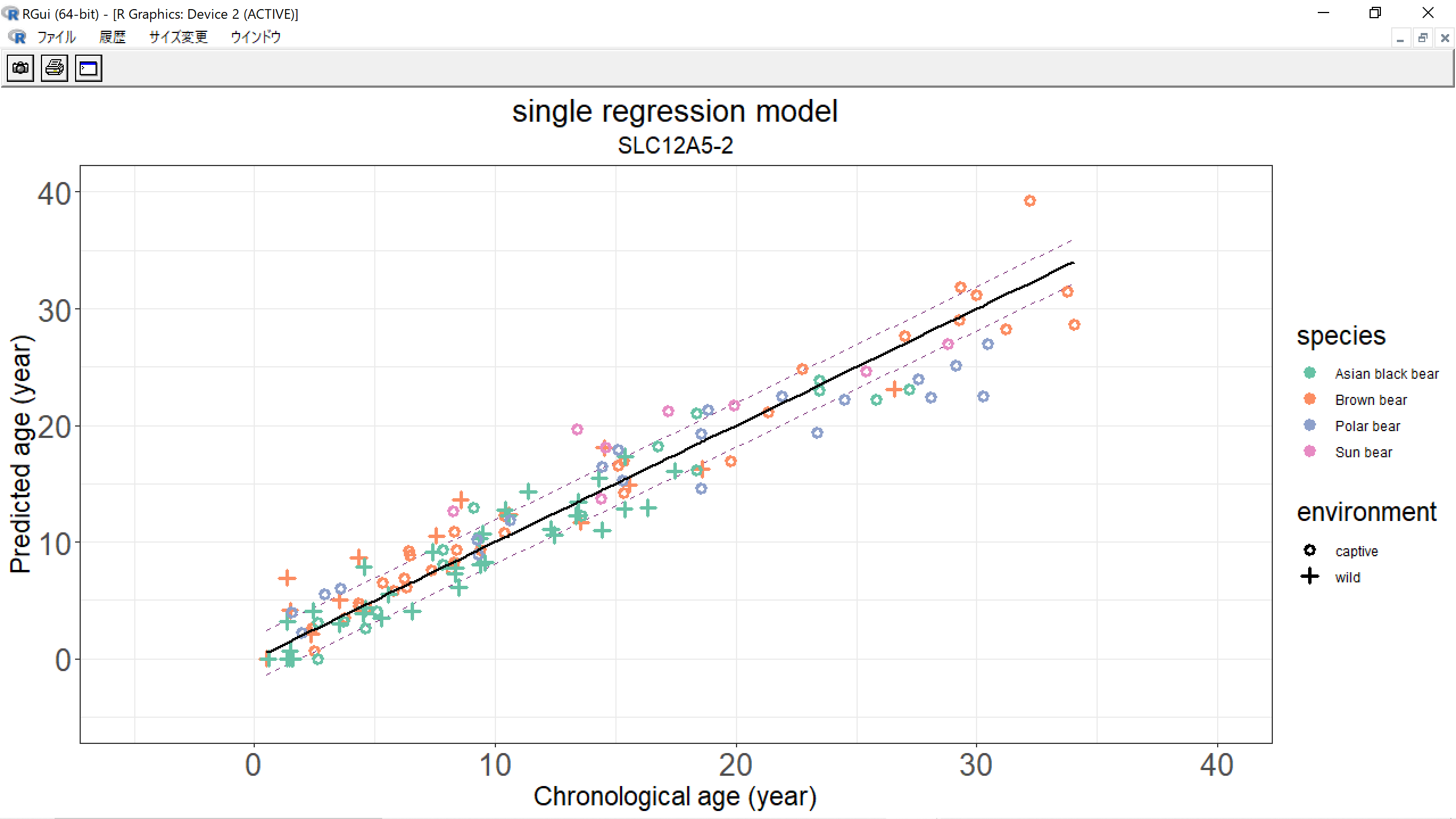


Single regression (SLC12A5-3)

SRM_SLC_3<-lm(formula=YS~slc3S,data=IBBS)

coef(SRM_SLC_3)

(Intercept) slc3S

-2.273060e-11 9.549695e-01

#LOOCV

nSamples<-nrow(IBBS)

predict_SRM_SLC_3_loocv<-numeric(nSamples)

for (z in 1:nSamples){

indices<-removeOne(nSamples,z)

dr<-data.frame(IBBS$age[indices],IBBS$SLC12A5_3_methylation_rate_ave[indices])

colnames(dr)<-c("age","methylslc_3")

bestmodel_SRM_SLC_3_loocv<-lm(age~methylslc_3,data=dr)

newdata<-data.frame(methylslc_3=IBBS$SLC12A5_3_methylation_rate_ave[z])

p<-predict(bestmodel_SRM_SLC_3_loocv,newdata)*sd(AGE)+mean(AGE)

if (p<0){p=0}

predict_SRM_SLC_3_loocv[z]<-p}

IBB_predict_SRM_SLC_3_loocv<-cbind(IBB,predict_SRM_SLC_3_loocv)

MAE_SRM_SLC_3_loocv<-mean(abs(IBB_predict_SRM_SLC_3_loocv$predict_SRM_SLC_3_loocv-IBB_predict_SRM_SLC_3_loocv$age))

MedianAE_SRM_SLC_3_loocv<-median(abs(IBB_predict_SRM_SLC_3_loocv$predict_SRM_SLC_3_loocv-IBB_predict_SRM_SLC_3_loocv$age))

RMSE_SRM_SLC_3_loocv<-sqrt(mean((IBB_predict_SRM_SLC_3_loocv$predict_SRM_SLC_3_loocv-IBB_predict_SRM_SLC_3_loocv$age)^2))

cat("MAE:", MAE_SRM_SLC_3_loocv, "\nMed AE:", MedianAE_SRM_SLC_3_loocv, "\nRMSE:", RMSE_SRM_SLC_3_loocv, "\n")

MAE: 2.075267

Med AE: 1.514708

RMSE: 2.682901

g_SRM_SLC_3_loocv_ss<-ggplot(IBB_predict_SRM_SLC_3_loocv,aes(age,predict_SRM_SLC_3_loocv))+theme_bw()+

annotate("segment",x=min(IBB$age),xend=max(IBB$age),y=min(IBB$age)+2.075267,yend=max(IBB$age)+2.075267,colour="orchid4",linetype=2, linewidth =0.7)+

annotate("segment",x=min(IBB$age),xend=max(IBB$age),y=min(IBB$age)-2.075267,yend=max(IBB$age)-2.075267,colour="orchid4",linetype=2, linewidth =0.7)+

geom_point(aes(shape=sex,color=species),size=2,stroke=2)+

labs(x="Chronological age (year)",y="Predicted age (year)")+

scale_shape_manual(name="sex",labels=c("F" = "female", "M" = "male"),values=c("F" = 1, "M" = 3))+

scale_color_manual(name="species",labels=c("ABB" = "Asian black bear", "PB" = "Polar bear", "BB" = "Brown bear", "SB" = "Sun bear"), values = c("ABB" = "#66C2A5", "PB" = "#8DA0CB", "BB" = "#FC8D62", "SB" = "#E78AC3"))+

theme(axis.text.x=element_text(size=20),axis.text.y=element_text(size=20))+

theme(axis.title.x=element_text(size=17),axis.title.y=element_text(size=17))+

geom_line(aes(y =age), linewidth=1)+

labs(title="single regression model")+

theme(title=element_text(size=17),plot.title=element_text(hjust=0.5))+

scale_y_continuous(limits=c(-5,40))+

scale_x_continuous(limits=c(-5,40))+

labs(subtitle="SLC12A5-3")+

theme(plot.subtitle=element_text(size=15,hjust=0.5))+

guides(color = guide_legend(order = 1), shape = guide_legend(order = 2))


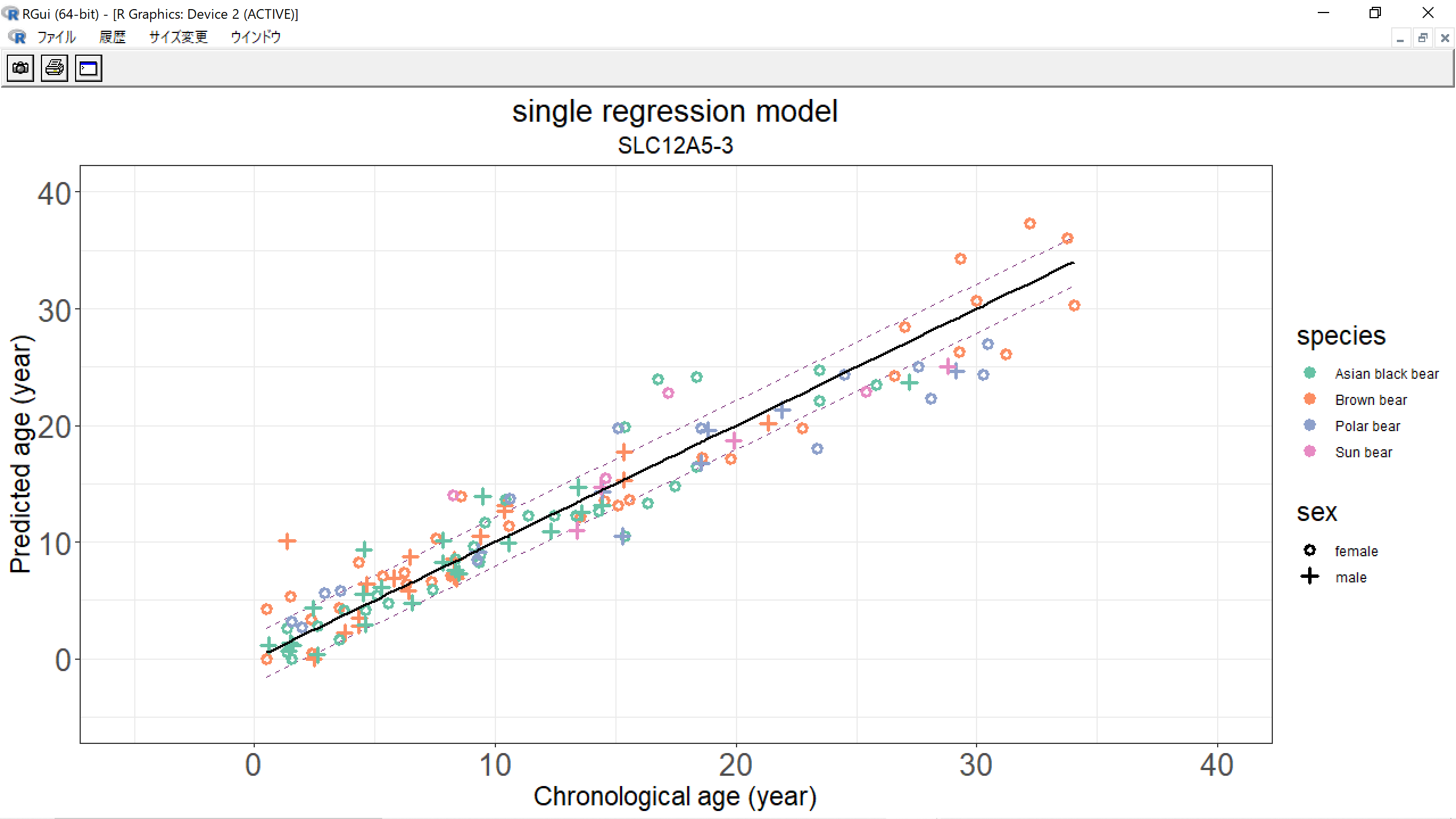


g_SRM_SLC_3_loocv_se<-ggplot(IBB_predict_SRM_SLC_3_loocv,aes(age,predict_SRM_SLC_3_loocv))+theme_bw()+

annotate("segment",x=min(IBB$age),xend=max(IBB$age),y=min(IBB$age)+2.075267,yend=max(IBB$age)+2.075267,colour="orchid4",linetype=2, linewidth =0.7)+

annotate("segment",x=min(IBB$age),xend=max(IBB$age),y=min(IBB$age)-2.075267,yend=max(IBB$age)-2.075267,colour="orchid4",linetype=2, linewidth =0.7)+

geom_point(aes(shape=environment,color=species),size=2,stroke=2)+

labs(x="Chronological age (year)",y="Predicted age (year)")+

scale_shape_manual(name="environment",labels=c("captive", "wild"),values=c(1,3))+

scale_color_manual(name="species",labels=c("ABB" = "Asian black bear", "PB" = "Polar bear", "BB" = "Brown bear", "SB" = "Sun bear"), values = c("ABB" = "#66C2A5", "PB" = "#8DA0CB", "BB" = "#FC8D62", "SB" = "#E78AC3"))+

theme(axis.text.x=element_text(size=20),axis.text.y=element_text(size=20))+

theme(axis.title.x=element_text(size=17),axis.title.y=element_text(size=17))+

geom_line(aes(y =age), linewidth=1)+

labs(title="single regression model")+

theme(title=element_text(size=17),plot.title=element_text(hjust=0.5))+

scale_y_continuous(limits=c(-5,40))+

scale_x_continuous(limits=c(-5,40))+

labs(subtitle="SLC12A5-3")+

theme(plot.subtitle=element_text(size=15,hjust=0.5))+

guides(color = guide_legend(order = 1), shape = guide_legend(order = 2))


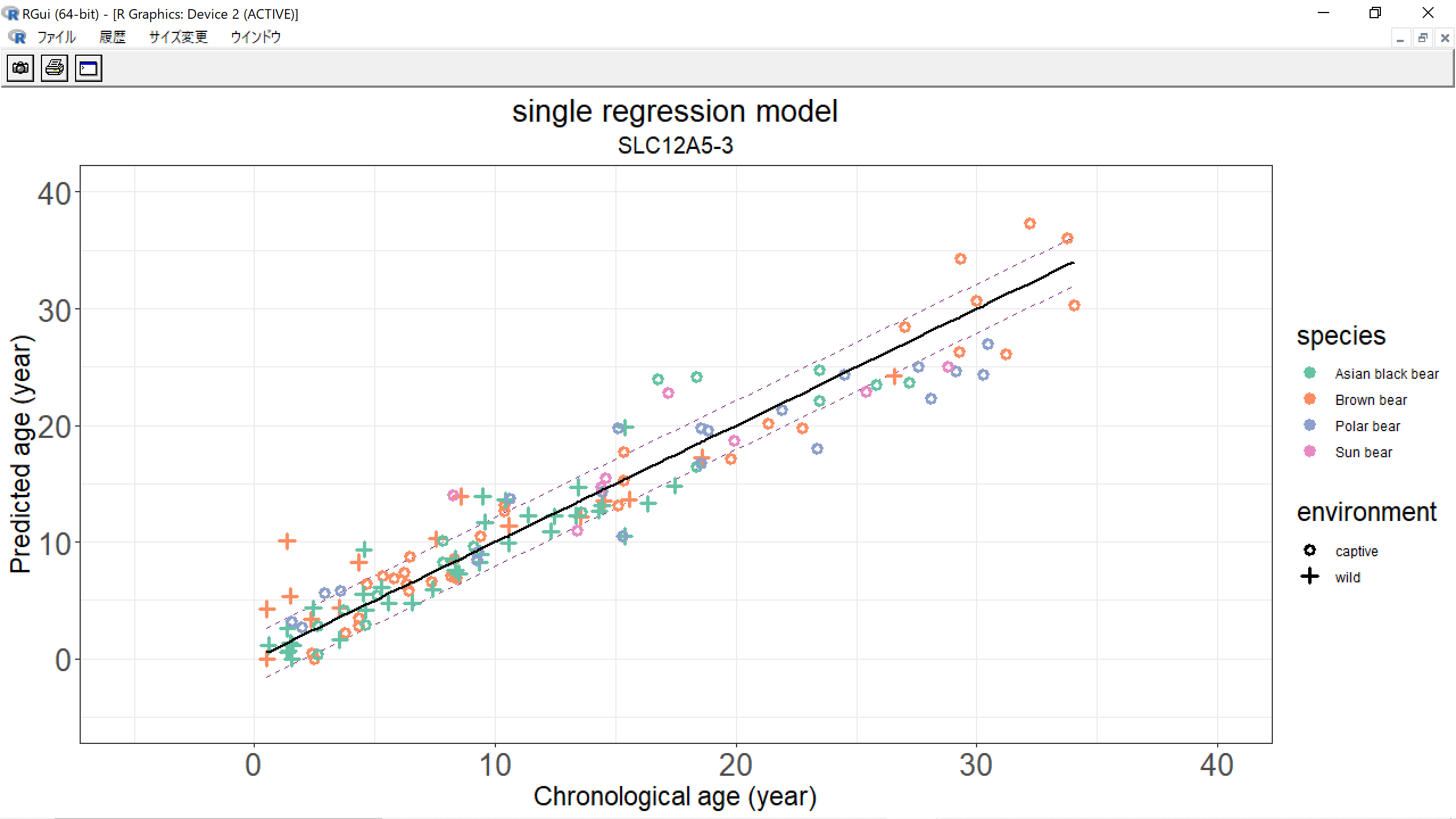


Single regression (SLC12A5-4)

SRM_SLC_4<-lm(formula=YS~slc4S,data=IBBS)

coef(SRM_SLC_4)

(Intercept) slc4S

-6.770082e-11 9.715851e-01

#LOOCV

nSamples<-nrow(IBBS)

predict_SRM_SLC_4_loocv<-numeric(nSamples)

for (z in 1:nSamples){

indices<-removeOne(nSamples,z)

dr<-data.frame(IBBS$age[indices],IBBS$SLC12A5_4_methylation_rate_ave[indices])

colnames(dr)<-c("age","methylslc_4")

bestmodel_SRM_SLC_4_loocv<-lm(age~methylslc_4,data=dr)

newdata<-data.frame(methylslc_4=IBBS$SLC12A5_4_methylation_rate_ave[z])

p<-predict(bestmodel_SRM_SLC_4_loocv,newdata)*sd(AGE)+mean(AGE)

if (p<0){p=0}

predict_SRM_SLC_4_loocv[z]<-p}

IBB_predict_SRM_SLC_4_loocv<-cbind(IBB,predict_SRM_SLC_4_loocv)

MAE_SRM_SLC_4_loocv<-mean(abs(IBB_predict_SRM_SLC_4_loocv$predict_SRM_SLC_4_loocv-IBB_predict_SRM_SLC_4_loocv$age))

MedianAE_SRM_SLC_4_loocv<-median(abs(IBB_predict_SRM_SLC_4_loocv$predict_SRM_SLC_4_loocv-IBB_predict_SRM_SLC_4_loocv$age))

RMSE_SRM_SLC_4_loocv<-sqrt(mean((IBB_predict_SRM_SLC_4_loocv$predict_SRM_SLC_4_loocv-IBB_predict_SRM_SLC_4_loocv$age)^2))

cat("MAE:", MAE_SRM_SLC_4_loocv, "\nMed AE:", MedianAE_SRM_SLC_4_loocv, "\nRMSE:", RMSE_SRM_SLC_4_loocv, "\n")

MAE: 1.662605

Med AE: 1.309572

RMSE: 2.149305

g_SRM_SLC_4_loocv_ss<-ggplot(IBB_predict_SRM_SLC_4_loocv,aes(age,predict_SRM_SLC_4_loocv))+theme_bw()+

annotate("segment",x=min(IBB$age),xend=max(IBB$age),y=min(IBB$age)+1.662605,yend=max(IBB$age)+1.662605,colour="orchid4",linetype=2, linewidth =0.7)+

annotate("segment",x=min(IBB$age),xend=max(IBB$age),y=min(IBB$age)-1.662605,yend=max(IBB$age)-1.662605,colour="orchid4",linetype=2, linewidth =0.7)+

geom_point(aes(shape=sex,color=species),size=2,stroke=2)+

labs(x="Chronological age (year)",y="Predicted age (year)")+

scale_shape_manual(name="sex",labels=c("F" = "female", "M" = "male"),values=c("F" = 1, "M" = 3))+

scale_color_manual(name="species",labels=c("ABB" = "Asian black bear", "PB" = "Polar bear", "BB" = "Brown bear", "SB" = "Sun bear"), values = c("ABB" = "#66C2A5", "PB" = "#8DA0CB", "BB" = "#FC8D62", "SB" = "#E78AC3"))+

theme(axis.text.x=element_text(size=20),axis.text.y=element_text(size=20))+

theme(axis.title.x=element_text(size=17),axis.title.y=element_text(size=17))+

geom_line(aes(y =age), linewidth=1)+

labs(title="single regression model")+

theme(title=element_text(size=17),plot.title=element_text(hjust=0.5))+

scale_y_continuous(limits=c(-5,40))+

scale_x_continuous(limits=c(-5,40))+

labs(subtitle="SLC12A5-4")+

theme(plot.subtitle=element_text(size=15,hjust=0.5))+

guides(color = guide_legend(order = 1), shape = guide_legend(order = 2))


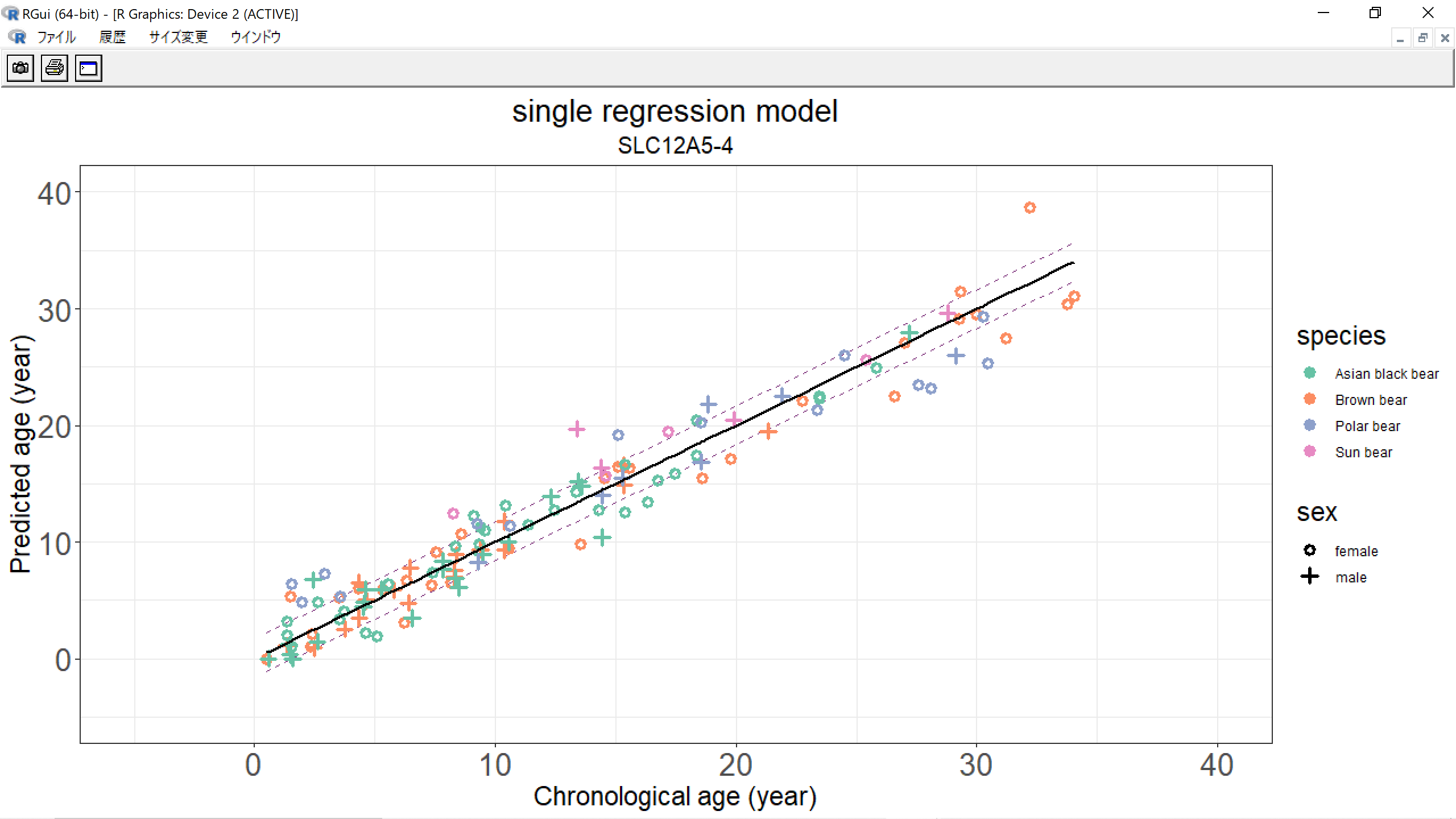


g_SRM_SLC_4_loocv_se<-ggplot(IBB_predict_SRM_SLC_4_loocv,aes(age,predict_SRM_SLC_4_loocv))+theme_bw()+

annotate("segment",x=min(IBB$age),xend=max(IBB$age),y=min(IBB$age)+1.662605,yend=max(IBB$age)+1.662605,colour="orchid4",linetype=2, linewidth =0.7)+

annotate("segment",x=min(IBB$age),xend=max(IBB$age),y=min(IBB$age)-1.662605,yend=max(IBB$age)-1.662605,colour="orchid4",linetype=2, linewidth =0.7)+

geom_point(aes(shape=environment,color=species),size=2,stroke=2)+

labs(x="Chronological age (year)",y="Predicted age (year)")+

scale_shape_manual(name="environment",labels=c("captive", "wild"),values=c(1,3))+

scale_color_manual(name="species",labels=c("ABB" = "Asian black bear", "PB" = "Polar bear", "BB" = "Brown bear", "SB" = "Sun bear"), values = c("ABB" = "#66C2A5", "PB" = "#8DA0CB", "BB" = "#FC8D62", "SB" = "#E78AC3"))+

theme(axis.text.x=element_text(size=20),axis.text.y=element_text(size=20))+

theme(axis.title.x=element_text(size=17),axis.title.y=element_text(size=17))+

geom_line(aes(y =age), linewidth=1)+

labs(title="single regression model")+

theme(title=element_text(size=17),plot.title=element_text(hjust=0.5))+

scale_y_continuous(limits=c(-5,40))+

scale_x_continuous(limits=c(-5,40))+

labs(subtitle="SLC12A5-4")+

theme(plot.subtitle=element_text(size=15,hjust=0.5))+

guides(color = guide_legend(order = 1), shape = guide_legend(order = 2))


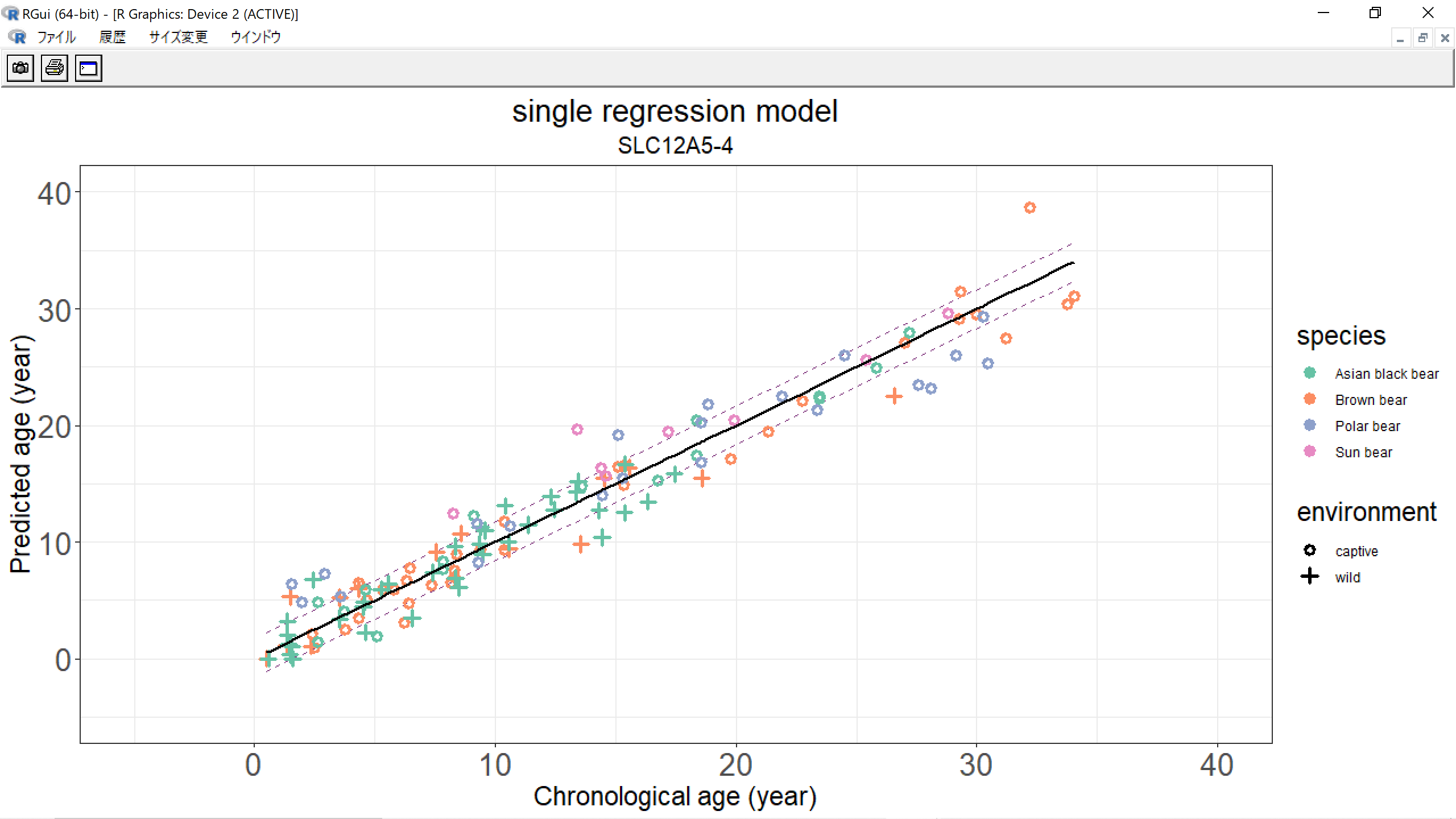


Age estimation model 【Principal component regression】

AGE <- IBB$age

#RemoveOne function

removeOne <- function(dat,x) {

if(x<dat){

list=seq(1,dat)

x1=x-1;x2=x+1

v1=c(list[0:x1]);v2=c(list[x2:dat])

data=c(v1,v2)}

else {data=seq(1,dat-1)}

return (data)}

pca_slc <- prcomp(IBBS[, c("SLC12A5_1_methylation_rate_ave",

"SLC12A5_2_methylation_rate_ave",

"SLC12A5_3_methylation_rate_ave",

"SLC12A5_4_methylation_rate_ave")],

scale = FALSE)

pca_slc

Standard deviations (1, .., p=4):

[1] 1.9736726 0.2188335 0.1889276 0.1450342

Rotation (n x k) = (4 x 4):

PC1 PC2 PC3 PC4

SLC12A5_1_methylation_rate_ave 0.4986143 0.6327512 0.5833223 0.1036572

SLC12A5_2_methylation_rate_ave 0.5023919 -0.1948124 -0.0689211 -0.8395835

SLC12A5_3_methylation_rate_ave 0.4999629 0.2645860 -0.7678198 0.3008059

SLC12A5_4_methylation_rate_ave 0.4990223 -0.7011907 0.2558080 0.4403074

screeplot(pca_slc, type = "lines", main = "Scree plot")


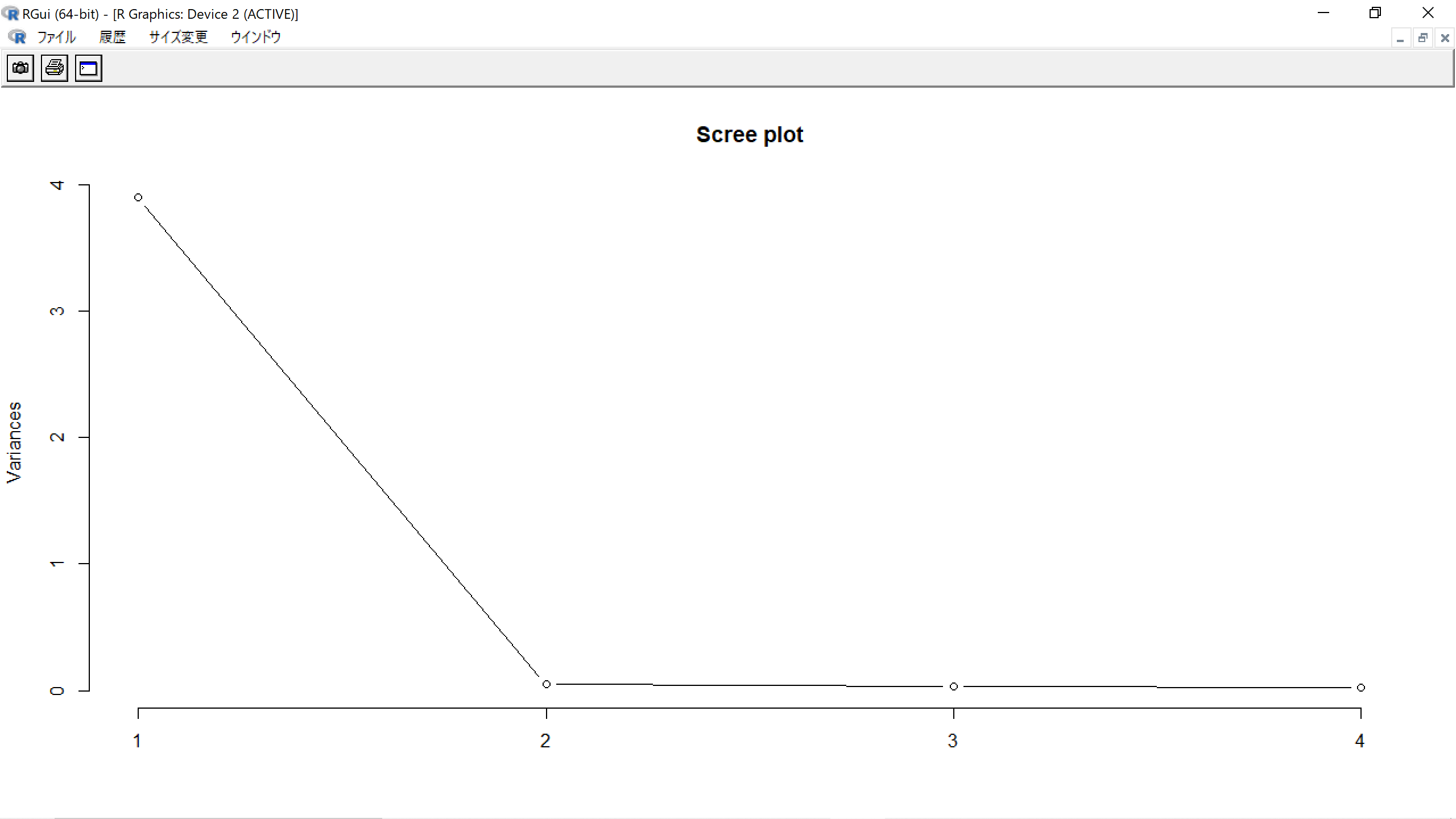


summary(pca_slc)

Importance of components:

PC1 PC2 PC3 PC4

Standard deviation 1.9737 0.21883 0.18893 0.14503

Proportion of Variance 0.9738 0.01197 0.00892 0.00526

Cumulative Proportion 0.9738 0.98582 0.99474 1.00000

Principal component regression (PC1)

IBBS$SLC12A5_PC1 <- pca_slc$x[,1]

PCRM_SLC <- lm(formula = age ~ SLC12A5_PC1, data = IBBS)

coef(PCRM_SLC)

(Intercept) SLC12A5_PC1

-1.538460e-11 4.923669e-01

#LOOCV

nSamples <- nrow(IBBS)

predict_PCRM_SLC_loocv <- numeric(nSamples)

for (z in 1:nSamples) {

indices <- removeOne(nSamples, z)

dr <- data.frame(IBBS$age[indices], IBBS$SLC12A5_PC1[indices])

colnames(dr) <- c("age", "methylslc_pc1")

bestmodel_PCRM_SLC_loocv <- lm(age ~ methylslc_pc1, data = dr)

newdata <- data.frame(methylslc_pc1 = IBBS$SLC12A5_PC1[z])

p <- predict(bestmodel_PCRM_SLC_loocv, newdata) * sd(AGE) + mean(AGE)

if (p < 0) { p = 0 }

predict_PCRM_SLC_loocv[z] <- p

}

IBB_predict_PCRM_SLC_loocv <- cbind(IBB, predict_PCRM_SLC_loocv)

MAE_PCRM_SLC_loocv <- mean(abs(IBB_predict_PCRM_SLC_loocv$predict_PCRM_SLC_loocv - IBB_predict_PCRM_SLC_loocv$age))

MedianAE_PCRM_SLC_loocv <- median(abs(IBB_predict_PCRM_SLC_loocv$predict_PCRM_SLC_loocv - IBB_predict_PCRM_SLC_loocv$age))

RMSE_PCRM_SLC_loocv <- sqrt(mean((IBB_predict_PCRM_SLC_loocv$predict_PCRM_SLC_loocv - IBB_predict_PCRM_SLC_loocv$age)^2))

cat("MAE:", MAE_PCRM_SLC_loocv, "\nMed AE:", MedianAE_PCRM_SLC_loocv, "\nRMSE:", RMSE_PCRM_SLC_loocv, "\n")

MAE: 1.668582

Med AE: 1.267238

RMSE: 2.13043

g_PCRM_SLC_loocv_ss<-ggplot(IBB_predict_PCRM_SLC_loocv,aes(age,predict_PCRM_SLC_loocv))+theme_bw()+

annotate("segment",x=min(IBB$age),xend=max(IBB$age),y=min(IBB$age)+1.668582,yend=max(IBB$age)+1.668582,colour="orchid4",linetype=2,linewidth =0.7)+

annotate("segment",x=min(IBB$age),xend=max(IBB$age),y=min(IBB$age)-1.668582,yend=max(IBB$age)-1.668582,colour="orchid4",linetype=2,linewidth =0.7)+

geom_point(aes(shape=sex,color=species),size=2,stroke=2)+

labs(x="Chronological age (year)",y="Predicted age (year)")+

scale_shape_manual(name="sex",labels=c("F" = "female", "M" = "male"),values=c("F" = 1, "M" = 3))+

scale_color_manual(name="species",labels=c("ABB" = "Asian black bear", "PB" = "Polar bear", "BB" = "Brown bear", "SB" = "Sun bear"), values = c("ABB" = "#66C2A5", "PB" = "#8DA0CB", "BB" = "#FC8D62", "SB" = "#E78AC3"))+

theme(axis.text.x=element_text(size=20),axis.text.y=element_text(size=20))+

theme(axis.title.x=element_text(size=17),axis.title.y=element_text(size=17))+

geom_line(aes(y =age), linewidth=1)+

labs(title="principal component regression model")+

theme(title=element_text(size=17),plot.title=element_text(hjust=0.5))+

scale_y_continuous(limits=c(-5,40))+

scale_x_continuous(limits=c(-5,40))+

labs(subtitle="SLC12A5-PC1")+

theme(plot.subtitle=element_text(size=15,hjust=0.5))+

guides(color = guide_legend(order = 1), shape = guide_legend(order = 2))


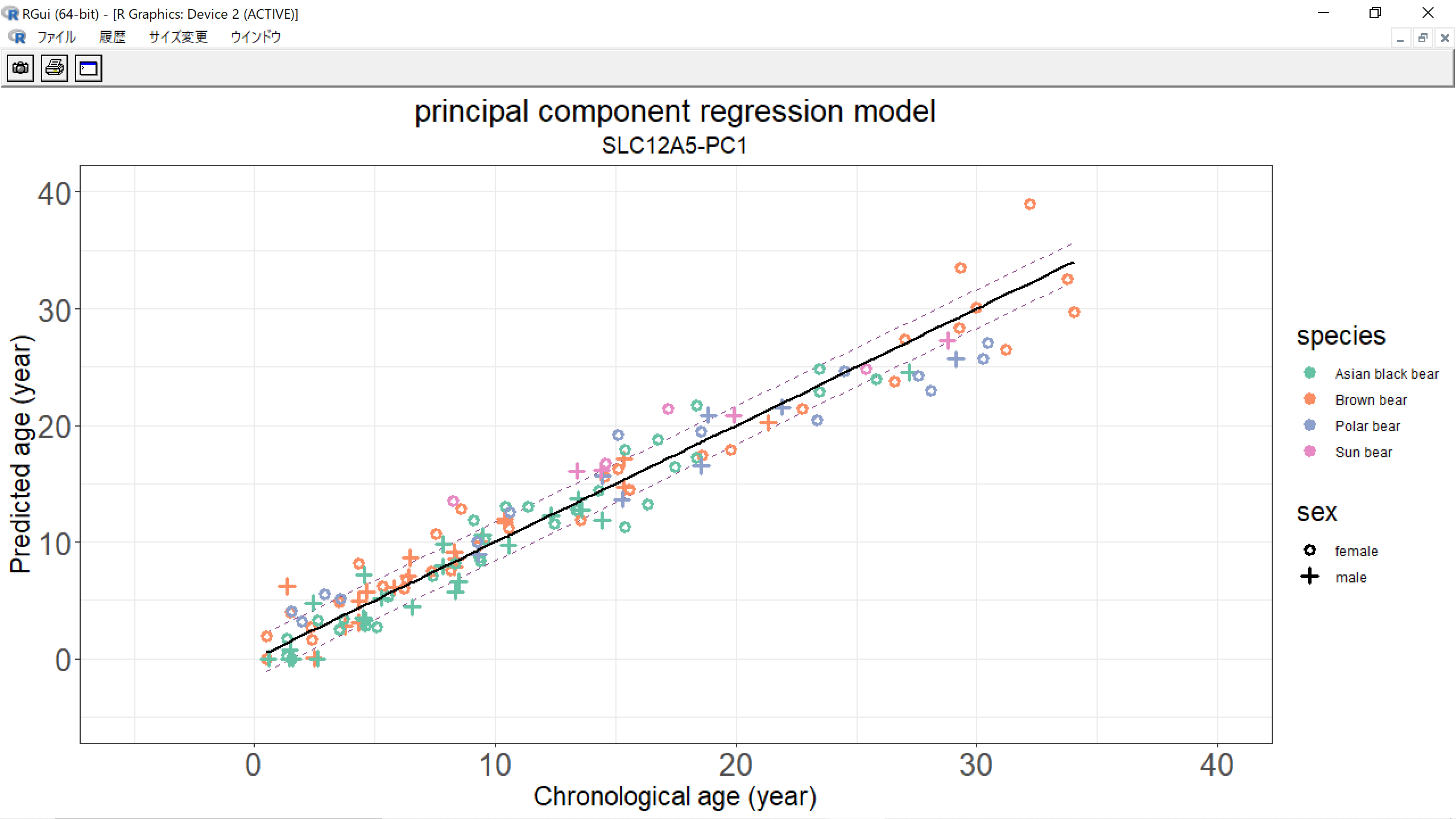


g_PCRM_SLC_loocv_se<-ggplot(IBB_predict_PCRM_SLC_loocv,aes(age,predict_PCRM_SLC_loocv))+theme_bw()+

annotate("segment",x=min(IBB$age),xend=max(IBB$age),y=min(IBB$age)+1.668582,yend=max(IBB$age)+1.668582,colour="orchid4",linetype=2,linewidth =0.7)+

annotate("segment",x=min(IBB$age),xend=max(IBB$age),y=min(IBB$age)-1.668582,yend=max(IBB$age)-1.668582,colour="orchid4",linetype=2,linewidth =0.7)+

geom_point(aes(shape=environment,color=species),size=2,stroke=2)+

labs(x="Chronological age (year)",y="Predicted age (year)")+

scale_shape_manual(name="environment",labels=c("captive", "wild"),values=c(1,3))+

scale_color_manual(name="species",labels=c("ABB" = "Asian black bear", "PB" = "Polar bear", "BB" = "Brown bear", "SB" = "Sun bear"), values = c("ABB" = "#66C2A5", "PB" = "#8DA0CB", "BB" = "#FC8D62", "SB" = "#E78AC3"))+

theme(axis.text.x=element_text(size=20),axis.text.y=element_text(size=20))+

theme(axis.title.x=element_text(size=17),axis.title.y=element_text(size=17))+

geom_line(aes(y =age), linewidth=1)+

labs(title="principal component regression model")+

theme(title=element_text(size=17),plot.title=element_text(hjust=0.5))+

scale_y_continuous(limits=c(-5,40))+

scale_x_continuous(limits=c(-5,40))+

labs(subtitle="SLC12A5-PC1")+

theme(plot.subtitle=element_text(size=15,hjust=0.5))+

guides(color = guide_legend(order = 1), shape = guide_legend(order = 2))


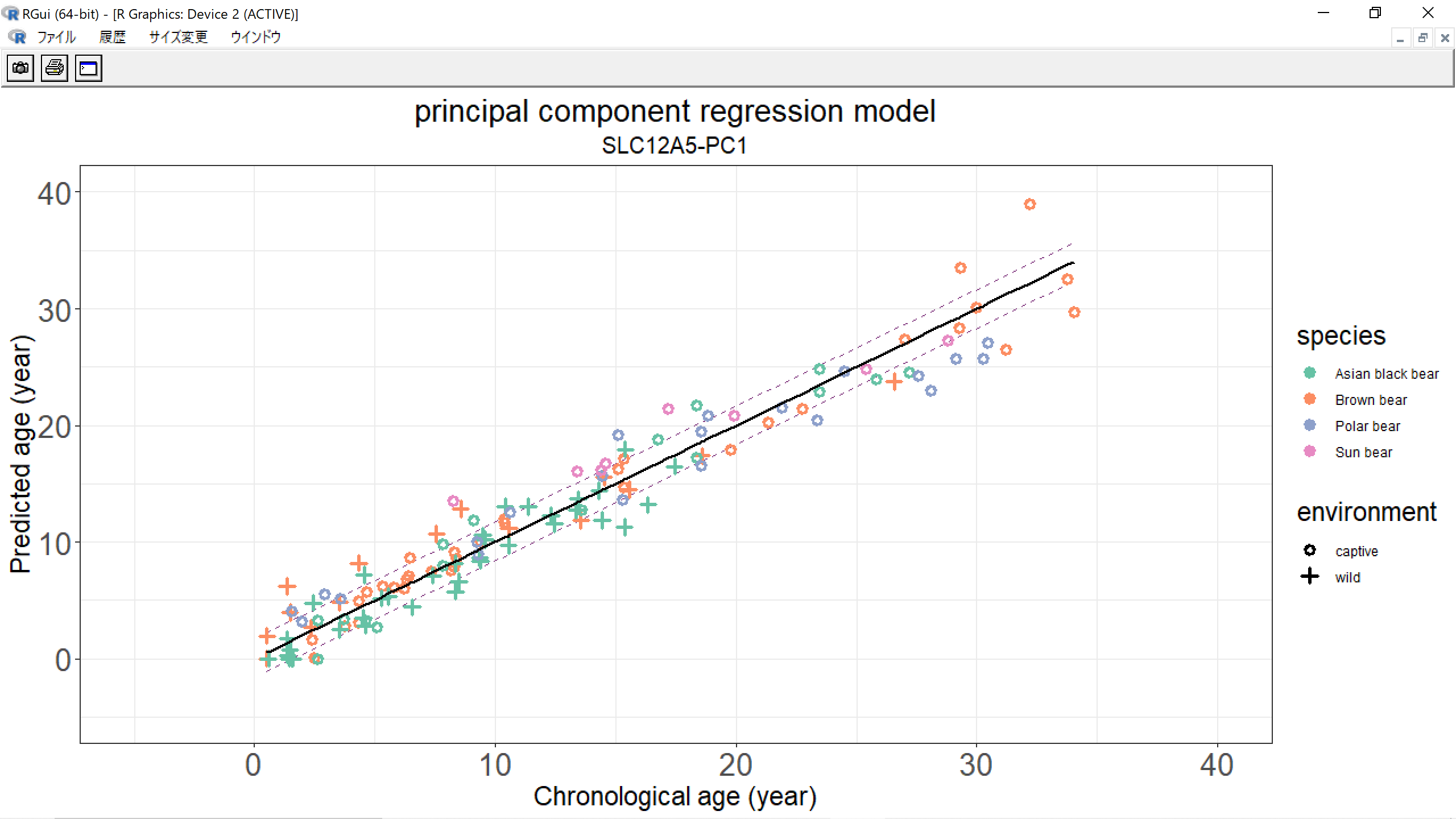


Age estimation model 【Elastic net regression】

YS <- IBBS$age

slc1S <- IBBS$SLC12A5_1_methylation_rate_ave

slc2S <- IBBS$SLC12A5_2_methylation_rate_ave

slc3S <- IBBS$SLC12A5_3_methylation_rate_ave

slc4S <- IBBS$SLC12A5_4_methylation_rate_ave

AGE <- IBB$age

#RemoveOne function

removeOne <- function(dat,x) {

if(x<dat){

list=seq(1,dat)

x1=x-1;x2=x+1

v1=c(list[0:x1]);v2=c(list[x2:dat])

data=c(v1,v2)}

else {data=seq(1,dat-1)}

return (data)}

Elastic net regression (SLC12A5-1, -2, -3, -4)

XS <- cbind(slc1S,slc2S,slc3S,slc4S)

set.seed(1)

alpha <- seq(0.01, 0.99, 0.01)

repeats <- 20

alpha.cvm.df <- NULL

for (i in 1:length(alpha)) {

alpha.repeats <- numeric(repeats)

for (j in 1:repeats) {

set.seed(j)

m <- cv.glmnet(x = XS, y = YS, family = "gaussian", alpha = alpha[i], standardize = FALSE)

alpha.repeats[j] <- min(m$cvm)

}

alpha.cvm.df <- rbind(alpha.cvm.df, data.frame(alpha = alpha[i], mincvm = mean(alpha.repeats)))

}

best.alpha <- alpha.cvm.df$alpha[which.min(alpha.cvm.df$mincvm)]

lambda.cvm.df <- NULL

lambda.list <- numeric(repeats)

cv.errors <- numeric(repeats)

for (i in 1:repeats) {

set.seed(i)

m <- cv.glmnet(x = XS, y = YS, family = "gaussian", alpha = best.alpha, standardize = FALSE)

lambda.cvm.df <- rbind(lambda.cvm.df, data.frame(lambda = m$lambda.min, mincvm = min(m$cvm)))

}

lambda_counts <- lambda.cvm.df %>%

group_by(lambda) %>%

summarise(count = n(), .groups = 'drop')

max_count <- max(lambda_counts$count)

candidates <- lambda_counts$lambda[lambda_counts$count == max_count]

best.lambda <- lambda.cvm.df %>%

filter(lambda %in% candidates) %>%

slice(which.min(mincvm)) %>%

pull(lambda)

cat("Best alpha:", best.alpha, "\nBest lambda:", best.lambda, "\n")

Best alpha: 0.02

Best lambda: 0.00842406

ENM1 <- glmnet(x = XS, y = YS, family = "gaussian", lambda = best.lambda, alpha = best.alpha, standardize = FALSE)

coef(ENM1,s=best.lambda)

5 x 1 sparse Matrix of class "dgCMatrix"

s1

(Intercept) -4.398378e-11

slc1S 9.913248e-02

slc2S 1.346553e-01

slc3S 1.988949e-01

slc4S 5.502739e-01

#LOOCV

nSamples<-nrow(IBBS)

predict_ENM_loocv<-numeric(nSamples)

for (z in 1:nSamples){

indices<-removeOne(nSamples,z)

X1 <-cbind(IBBS$SLC12A5_1_methylation_rate_ave[indices],IBBS$SLC12A5_2_methylation_rate_ave[indices],IBBS$SLC12A5_3_methylation_rate_ave[indices],IBBS$SLC12A5_4_methylation_rate_ave[indices])

Y1 <- IBBS$age[indices]

ENM2 <- glmnet(x = X1, y = Y1, family = "gaussian", lambda = best.lambda, alpha = best.alpha, standardize = FALSE)

Xnew <- cbind(IBBS$SLC12A5_1_methylation_rate_ave[z],IBBS$SLC12A5_2_methylation_rate_ave[z],IBBS$SLC12A5_3_methylation_rate_ave[z],IBBS$SLC12A5_4_methylation_rate_ave[z])

p<-predict(ENM2,Xnew,s=best.lambda) *sd(AGE)+mean(AGE)

if (p<0){p=0}

predict_ENM_loocv[z]<-p}

IBB_predict_ENM_loocv<-cbind(IBB,predict_ENM_loocv)

MAE_ENM_loocv<-mean(abs(IBB_predict_ENM_loocv$predict_ENM_loocv-IBB_predict_ENM_loocv$age))

MedianAE_ENM_loocv<-median(abs(IBB_predict_ENM_loocv$predict_ENM_loocv-IBB_predict_ENM_loocv$age))

RMSE_ENM_loocv<- sqrt(mean((IBB_predict_ENM_loocv$predict_ENM_loocv-IBB_predict_ENM_loocv$age)^2))

cat("MAE:", MAE_ENM_loocv, "\nMed AE:", MedianAE_ENM_loocv, "\nRMSE:", RMSE_ENM_loocv, "\n")

MAE: 1.572503

Med AE: 1.171544

RMSE: 2.044372

g_ENM_loocv_ss<-ggplot(IBB_predict_ENM_loocv,aes(age,predict_ENM_loocv))+theme_bw()+

annotate("segment",x=min(IBB$age),xend=max(IBB$age),y=min(IBB$age)+1.572503,yend=max(IBB$age)+1.572503,colour="orchid4",linetype=2,linewidth =0.7)+

annotate("segment",x=min(IBB$age),xend=max(IBB$age),y=min(IBB$age)-1.572503,yend=max(IBB$age)-1.572503,colour="orchid4",linetype=2,linewidth =0.7)+

geom_point(aes(shape=sex,color=species),size=2,stroke=2)+

labs(x="Chronological age (year)",y="Predicted age (year)")+

scale_shape_manual(name="sex",labels=c("F" = "female", "M" = "male"),values=c("F" = 1, "M" = 3))+

scale_color_manual(name="species",labels=c("ABB" = "Asian black bear", "PB" = "Polar bear", "BB" = "Brown bear", "SB" = "Sun bear"), values = c("ABB" = "#66C2A5", "PB" = "#8DA0CB", "BB" = "#FC8D62", "SB" = "#E78AC3"))+

theme(axis.text.x=element_text(size=20),axis.text.y=element_text(size=20))+

theme(axis.title.x=element_text(size=17),axis.title.y=element_text(size=17))+

geom_line(aes(y =age), linewidth=1)+

labs(title="elastic net regression model")+

theme(title=element_text(size=17),plot.title=element_text(hjust=0.5))+

scale_y_continuous(limits=c(-5,40))+

scale_x_continuous(limits=c(-5,40))+

labs(subtitle="SLC12A5-1, -2, -3, -4")+

theme(plot.subtitle=element_text(size=15,hjust=0.5))+

guides(color = guide_legend(order = 1), shape = guide_legend(order = 2))


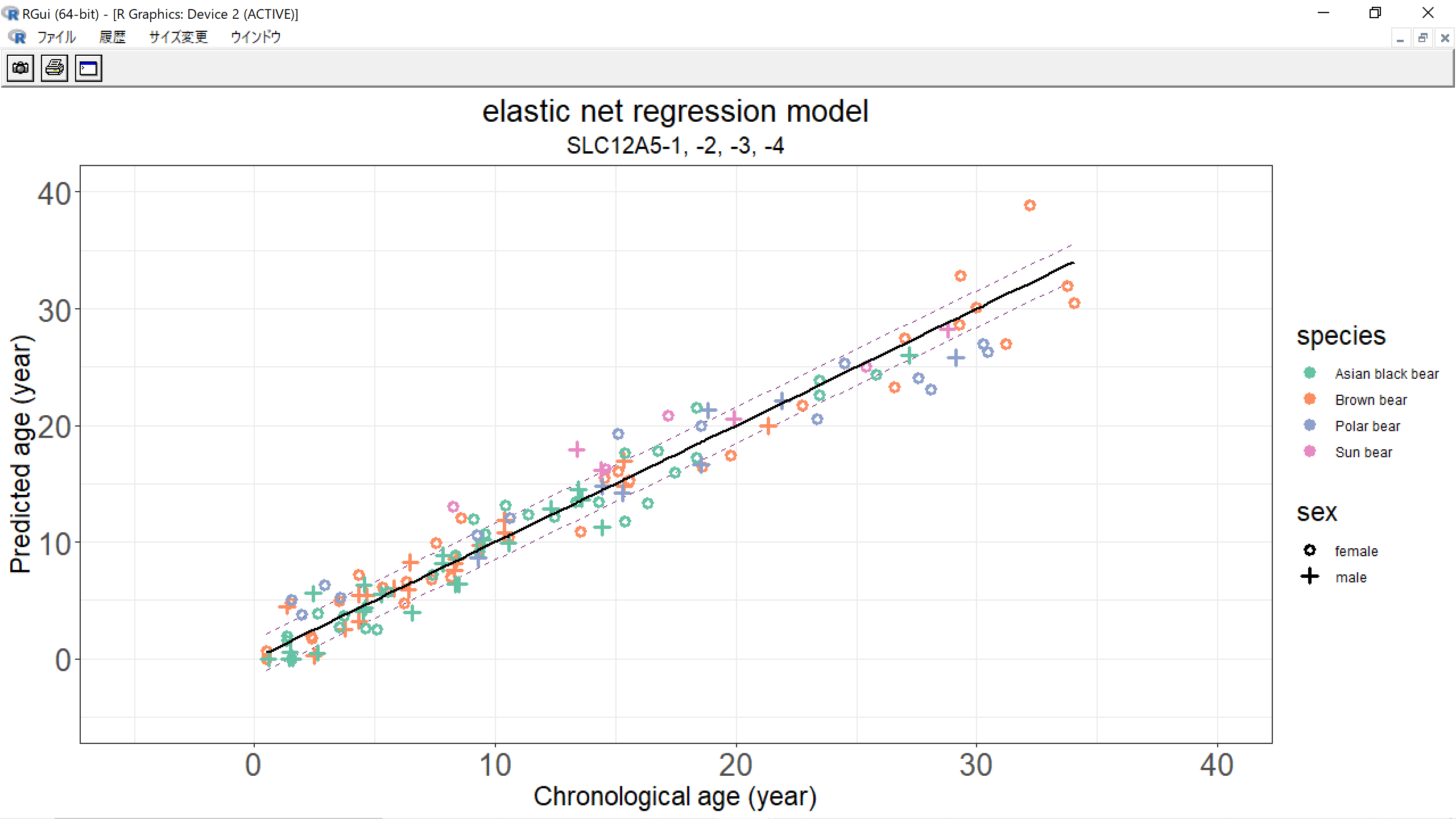


g_ENM_loocv_se<-ggplot(IBB_predict_ENM_loocv,aes(age,predict_ENM_loocv))+theme_bw()+

annotate("segment",x=min(IBB$age),xend=max(IBB$age),y=min(IBB$age)+1.572503,yend=max(IBB$age)+1.572503,colour="orchid4",linetype=2,linewidth =0.7)+

annotate("segment",x=min(IBB$age),xend=max(IBB$age),y=min(IBB$age)-1.572503,yend=max(IBB$age)-1.572503,colour="orchid4",linetype=2,linewidth =0.7)+

geom_point(aes(shape=environment,color=species),size=2,stroke=2)+

labs(x="Chronological age (year)",y="Predicted age (year)")+

scale_shape_manual(name="environment",labels=c("captive", "wild"),values=c(1,3))+

scale_color_manual(name="species",labels=c("ABB" = "Asian black bear", "PB" = "Polar bear", "BB" = "Brown bear", "SB" = "Sun bear"), values = c("ABB" = "#66C2A5", "PB" = "#8DA0CB", "BB" = "#FC8D62", "SB" = "#E78AC3"))+

theme(axis.text.x=element_text(size=20),axis.text.y=element_text(size=20))+

theme(axis.title.x=element_text(size=17),axis.title.y=element_text(size=17))+

geom_line(aes(y =age), linewidth=1)+

labs(title="elastic net regression model")+

theme(title=element_text(size=17),plot.title=element_text(hjust=0.5))+

scale_y_continuous(limits=c(-5,40))+

scale_x_continuous(limits=c(-5,40))+

labs(subtitle="SLC12A5-1, -2, -3, -4")+

theme(plot.subtitle=element_text(size=15,hjust=0.5))+

guides(color = guide_legend(order = 1), shape = guide_legend(order = 2))


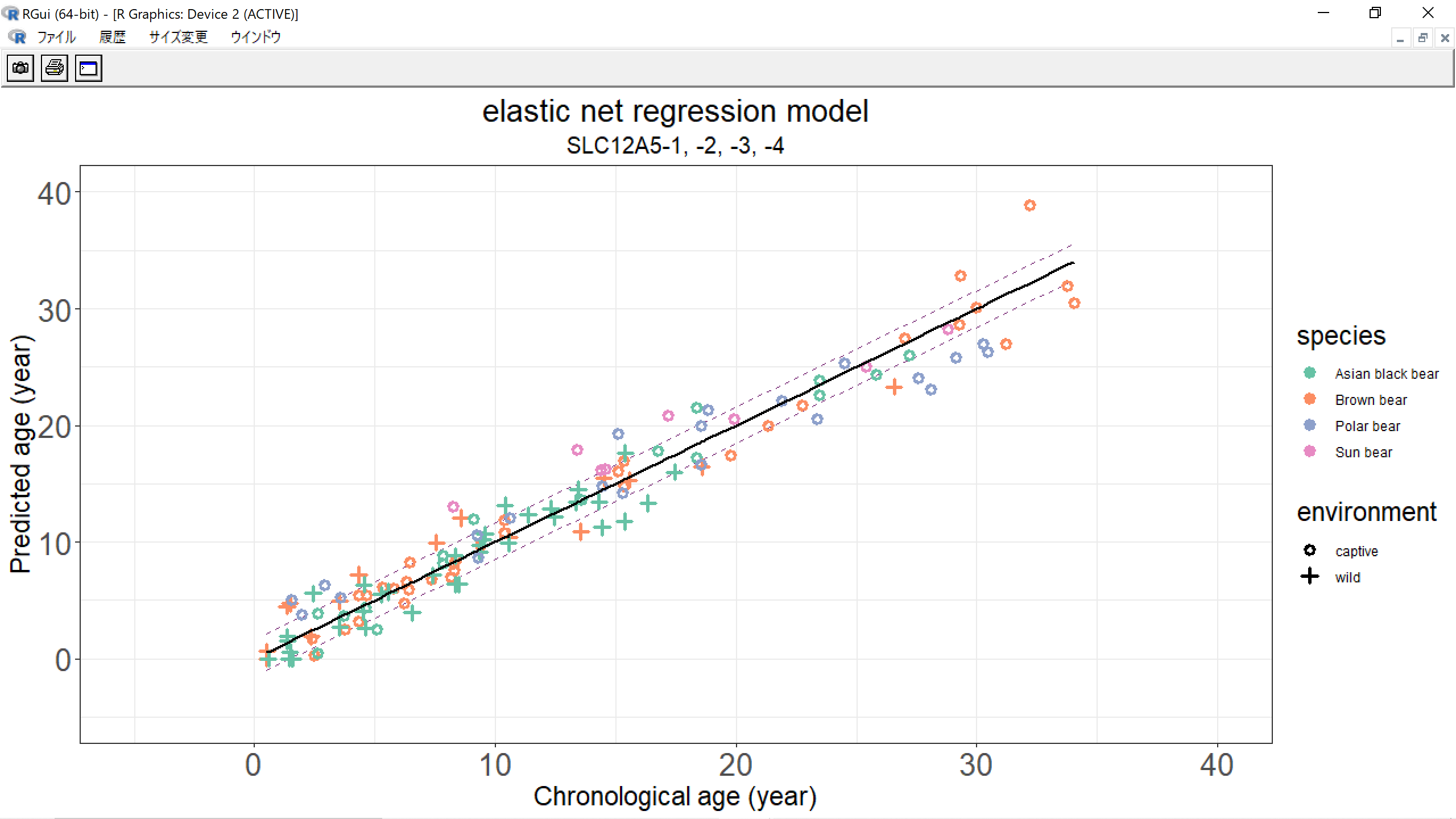


Age estimation model 【Support vector regression】

AGE <- IBB$age

#RemoveOne function

removeOne <- function(dat,x) {

if(x<dat){

list=seq(1,dat)

x1=x-1;x2=x+1

v1=c(list[0:x1]);v2=c(list[x2:dat])

data=c(v1,v2)}

else {data=seq(1,dat-1)}

return (data)}

Support vector regression (SLC12A5-1, -2, -3, -4)

set.seed(1)

tuneResult<-

tune(svm,age~SLC12A5_1_methylation_rate_ave+SLC12A5_2_methylation_rate_ave+SLC12A5_3_methylation_rate_ave+SLC12A5_4_methylation_rate_ave,data=IBBS,

ranges=list(cost=10^(seq(-2,4,0.1)),gamma=10^(seq(-5,1,0.1))),

tunecontrol = tune.control(sampling = "cross", cross = 10), scale = FALSE)

tunedModel <- tuneResult$best.model

tunedModel

Call:

best.tune(METHOD = svm, train.x = age ~ SLC12A5_1_methylation_rate_ave +

SLC12A5_2_methylation_rate_ave + SLC12A5_3_methylation_rate_ave +

SLC12A5_4_methylation_rate_ave, data = IBBS, ranges = list(cost = 10^(seq(-2,

4, 0.1)), gamma = 10^(seq(-5, 1, 0.1))), tunecontrol = tune.control(sampling = "cross",

cross = 10), scale = FALSE)

Parameters:

SVM-Type: eps-regression

SVM-Kernel: radial

cost: 1.584893

gamma: 0.06309573

epsilon: 0.1

Number of Support Vectors: 76

best.cost <- tunedModel$cost

best.gamma <- tunedModel$gamma

cat("Cost: ", best.cost, "\nGamma: ", best.gamma, "\n")

Cost: 1.584893

Gamma: 0.06309573

tune_results <- as.data.frame(tuneResult$performances)

tune_results$cost <- log10(tune_results$cost)

tune_results$gamma <- log10(tune_results$gamma)

ggplot(tune_results, aes(x = cost, y = gamma, fill = error)) +

geom_tile() +

geom_point(aes(x = log10(best.cost), y = log10(best.gamma)), color = "blue", size = 1, shape = 21, fill = "blue") +

scale_fill_gradient(low = "white", high = "red") +

labs(title = "SVM Parameter Tuning Results",

x = "Log10(Cost)",

y = "Log10(Gamma)",

fill = "Error") +

theme_minimal()


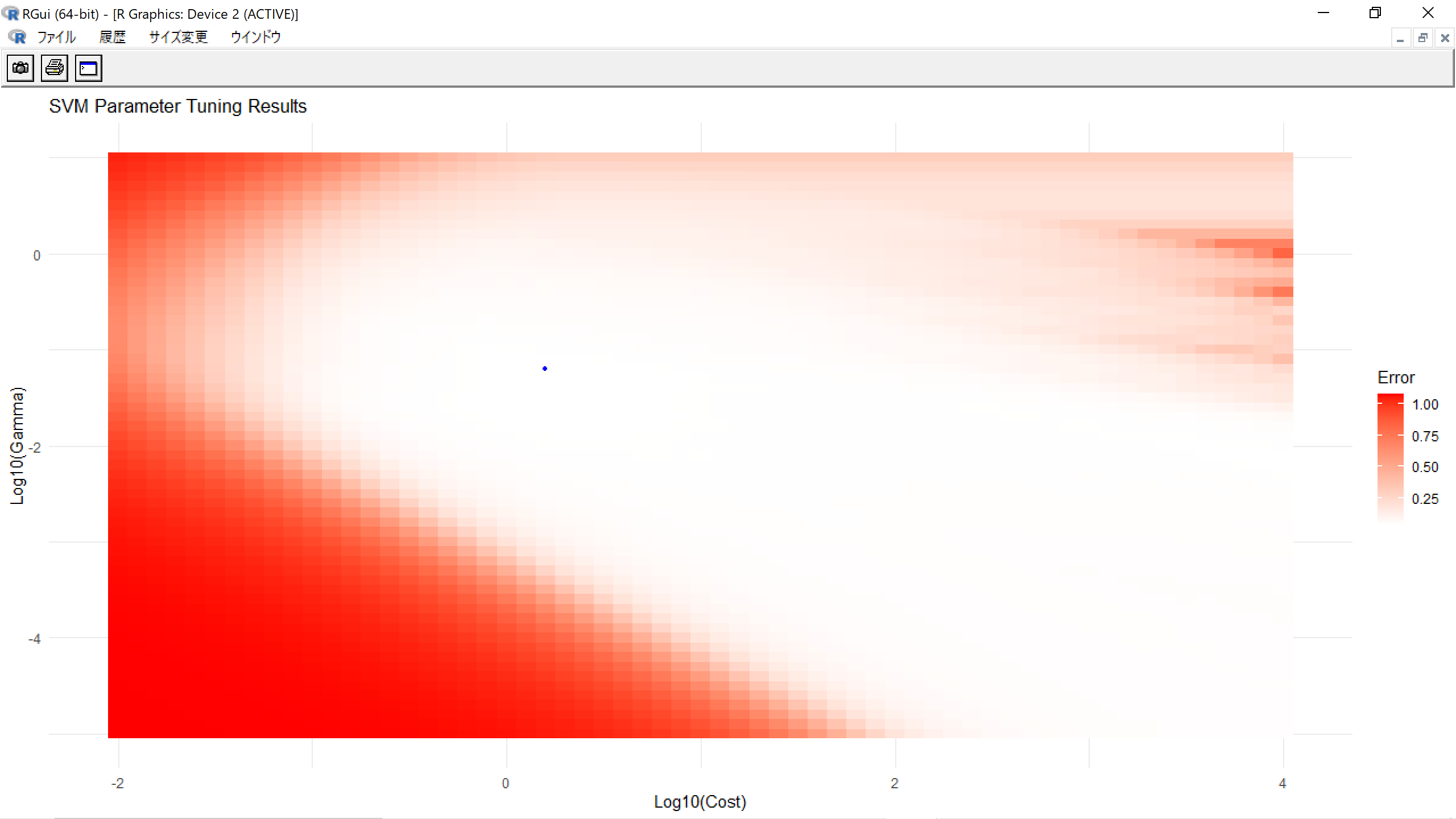


SVRM<-

svm(age~SLC12A5_1_methylation_rate_ave+SLC12A5_2_methylation_rate_ave+SLC12A5_3_methylation_rate_ave+SLC12A5_4_methylation_rate_ave,　data=IBBS,

cost=best.cost, gamma=best.gamma, epsilon=0.1, scale = FALSE)

#LOOCV

nSamples<-nrow(IBBS)

predict_SVRM_loocv<-numeric(nSamples)

for (z in 1:nSamples){

indices<-removeOne(nSamples,z)

dr<-data.frame(IBBS$age[indices],IBBS$SLC12A5_1_methylation_rate_ave[indices],IBBS$SLC12A5_2_methylation_rate_ave[indices],IBBS$SLC12A5_3_methylation_rate_ave[indices],IBBS$SLC12A5_4_methylation_rate_ave[indices])

colnames(dr)<-c("age","methylslc_1","methylslc_2","methylslc_3","methylslc_4")

bestmodel_SVRM<-svm(age~methylslc_1+methylslc_2+methylslc_3+methylslc_4, data=dr,

cost=best.cost, gamma= best.gamma, epsilon=0.1, scale = FALSE)

newdata<-data.frame(methylslc_1=IBBS$SLC12A5_1_methylation_rate_ave[z],methylslc_2=IBBS$SLC12A5_2_methylation_rate_ave[z],methylslc_3=IBBS$SLC12A5_3_methylation_rate_ave[z],methylslc_4=IBBS$SLC12A5_4_methylation_rate_ave[z])

p<-predict(bestmodel_SVRM,newdata)*sd(AGE)+mean(AGE)

if (p<0){p=0}

predict_SVRM_loocv[z]<-p}

IBB_SVRM_loocv<-cbind(IBB,predict_SVRM_loocv)

MAE_SVRM_loocv<-mean(abs(IBB_SVRM_loocv$predict_SVRM_loocv-IBB$age))

MedianAE_SVRM_loocv<-median(abs(IBB_SVRM_loocv$predict_SVRM_loocv-IBB$age))

RMSE_SVRM_loocv<- sqrt(mean((IBB_SVRM_loocv$predict_SVRM_loocv-IBB$age)^2))

cat("MAE:", MAE_SVRM_loocv, "\nMed AE:", MedianAE_SVRM_loocv, "\nRMSE:", RMSE_SVRM_loocv, "\n")

MAE: 1.475438

Med AE: 1.19218

RMSE: 1.863625

Support vector regression (SLC12A5-1, -2, -3)

set.seed(1)

tuneResult<-

tune(svm,age~SLC12A5_1_methylation_rate_ave+SLC12A5_2_methylation_rate_ave+SLC12A5_3_methylation_rate_ave,data=IBBS,

ranges=list(cost=10^(seq(-2,4,0.1)),gamma=10^(seq(-5,1,0.1))),

tunecontrol = tune.control(sampling = "cross", cross = 10), scale = FALSE)

tunedModel <- tuneResult$best.model

tunedModel

Call:

best.tune(METHOD = svm, train.x = age ~ SLC12A5_1_methylation_rate_ave +

SLC12A5_2_methylation_rate_ave + SLC12A5_3_methylation_rate_ave,

data = IBBS, ranges = list(cost = 10^(seq(-2, 4, 0.1)), gamma = 10^(seq(-5,

1, 0.1))), tunecontrol = tune.control(sampling = "cross",

cross = 10), scale = FALSE)

Parameters:

SVM-Type: eps-regression

SVM-Kernel: radial

cost: 1.258925

gamma: 0.1

epsilon: 0.1

Number of Support Vectors: 86

best.cost <- tunedModel$cost

best.gamma <- tunedModel$gamma

cat("Cost: ", best.cost, "\nGamma: ", best.gamma, "\n")

Cost: 1.258925

Gamma: 0.1

tune_results <- as.data.frame(tuneResult$performances)

tune_results$cost <- log10(tune_results$cost)

tune_results$gamma <- log10(tune_results$gamma)

ggplot(tune_results, aes(x = cost, y = gamma, fill = error)) +

geom_tile() +

geom_point(aes(x = log10(best.cost), y = log10(best.gamma)), color = "blue", size = 1, shape = 21, fill = "blue") +

scale_fill_gradient(low = "white", high = "red") +

labs(title = "SVM Parameter Tuning Results",

x = "Log10(Cost)",

y = "Log10(Gamma)",

fill = "Error") +

theme_minimal()


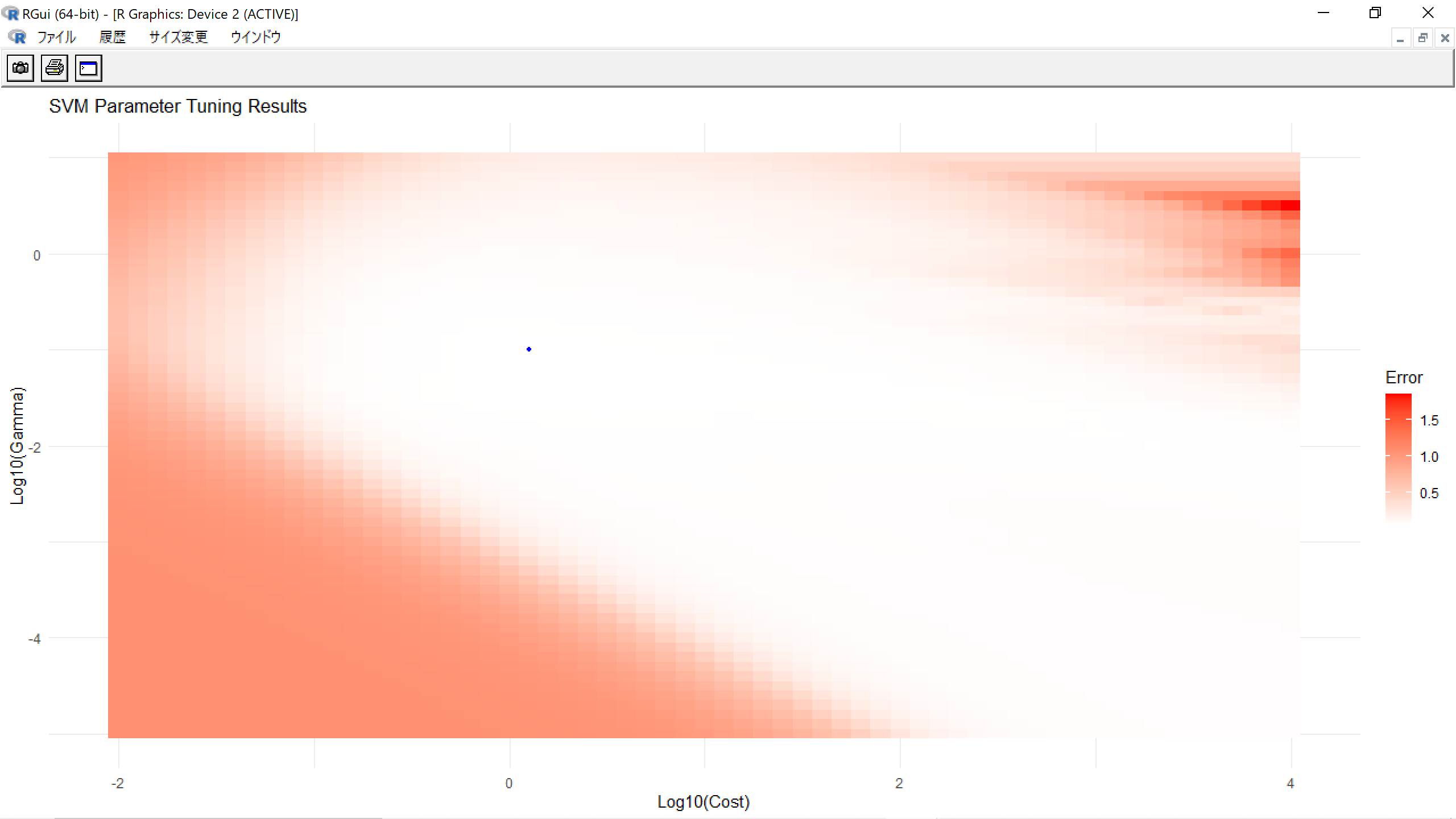


SVRM<-

svm(age~SLC12A5_1_methylation_rate_ave+SLC12A5_2_methylation_rate_ave+SLC12A5_3_methylation_rate_ave,　data=IBBS,

cost=best.cost, gamma=best.gamma, epsilon=0.1, scale = FALSE)

#LOOCV

nSamples<-nrow(IBBS)

predict_SVRM_loocv<-numeric(nSamples)

for (z in 1:nSamples){

indices<-removeOne(nSamples,z)

dr<-data.frame(IBBS$age[indices],IBBS$SLC12A5_1_methylation_rate_ave[indices],IBBS$SLC12A5_2_methylation_rate_ave[indices],IBBS$SLC12A5_3_methylation_rate_ave[indices])

colnames(dr)<-c("age","methylslc_1","methylslc_2","methylslc_3")

bestmodel_SVRM<-svm(age~methylslc_1+methylslc_2+methylslc_3, data=dr,

cost=best.cost, gamma= best.gamma, epsilon=0.1, scale = FALSE)

newdata<-data.frame(methylslc_1=IBBS$SLC12A5_1_methylation_rate_ave[z],methylslc_2=IBBS$SLC12A5_2_methylation_rate_ave[z],methylslc_3=IBBS$SLC12A5_3_methylation_rate_ave[z])

p<-predict(bestmodel_SVRM,newdata)*sd(AGE)+mean(AGE)

if (p<0){p=0}

predict_SVRM_loocv[z]<-p}

IBB_SVRM_loocv<-cbind(IBB,predict_SVRM_loocv)

MAE_SVRM_loocv<-mean(abs(IBB_SVRM_loocv$predict_SVRM_loocv-IBB$age))

MedianAE_SVRM_loocv<-median(abs(IBB_SVRM_loocv$predict_SVRM_loocv-IBB$age))

RMSE_SVRM_loocv<- sqrt(mean((IBB_SVRM_loocv$predict_SVRM_loocv-IBB$age)^2))

cat("MAE:", MAE_SVRM_loocv, "\nMed AE:", MedianAE_SVRM_loocv, "\nRMSE:", RMSE_SVRM_loocv, "\n")

MAE: 1.654124

Med AE: 1.383427

RMSE: 2.076068

Support vector regression (SLC12A5-1, -2, -4)

set.seed(1)

tuneResult<-

tune(svm,age~SLC12A5_1_methylation_rate_ave+SLC12A5_2_methylation_rate_ave+SLC12A5_4_methylation_rate_ave,data=IBBS,

ranges=list(cost=10^(seq(-2,4,0.1)),gamma=10^(seq(-5,1,0.1))),

tunecontrol = tune.control(sampling = "cross", cross = 10), scale = FALSE)

tunedModel <- tuneResult$best.model

tunedModel

Call:

best.tune(METHOD = svm, train.x = age ~ SLC12A5_1_methylation_rate_ave +

SLC12A5_2_methylation_rate_ave + SLC12A5_4_methylation_rate_ave,

data = IBBS, ranges = list(cost = 10^(seq(-2, 4, 0.1)), gamma = 10^(seq(-5,

1, 0.1))), tunecontrol = tune.control(sampling = "cross",

cross = 10), scale = FALSE)

Parameters:

SVM-Type: eps-regression

SVM-Kernel: radial

cost: 1.584893

gamma: 0.03162278

epsilon: 0.1

Number of Support Vectors: 76

best.cost <- tunedModel$cost

best.gamma <- tunedModel$gamma

cat("Cost: ", best.cost, "\nGamma: ", best.gamma, "\n")

Cost: 1.584893

Gamma: 0.03162278

tune_results <- as.data.frame(tuneResult$performances)

tune_results$cost <- log10(tune_results$cost)

tune_results$gamma <- log10(tune_results$gamma)

ggplot(tune_results, aes(x = cost, y = gamma, fill = error)) +

geom_tile() +

geom_point(aes(x = log10(best.cost), y = log10(best.gamma)), color = "blue", size = 1, shape = 21, fill = "blue") +

scale_fill_gradient(low = "white", high = "red") +

labs(title = "SVM Parameter Tuning Results",

x = "Log10(Cost)",

y = "Log10(Gamma)",

fill = "Error") +

theme_minimal()


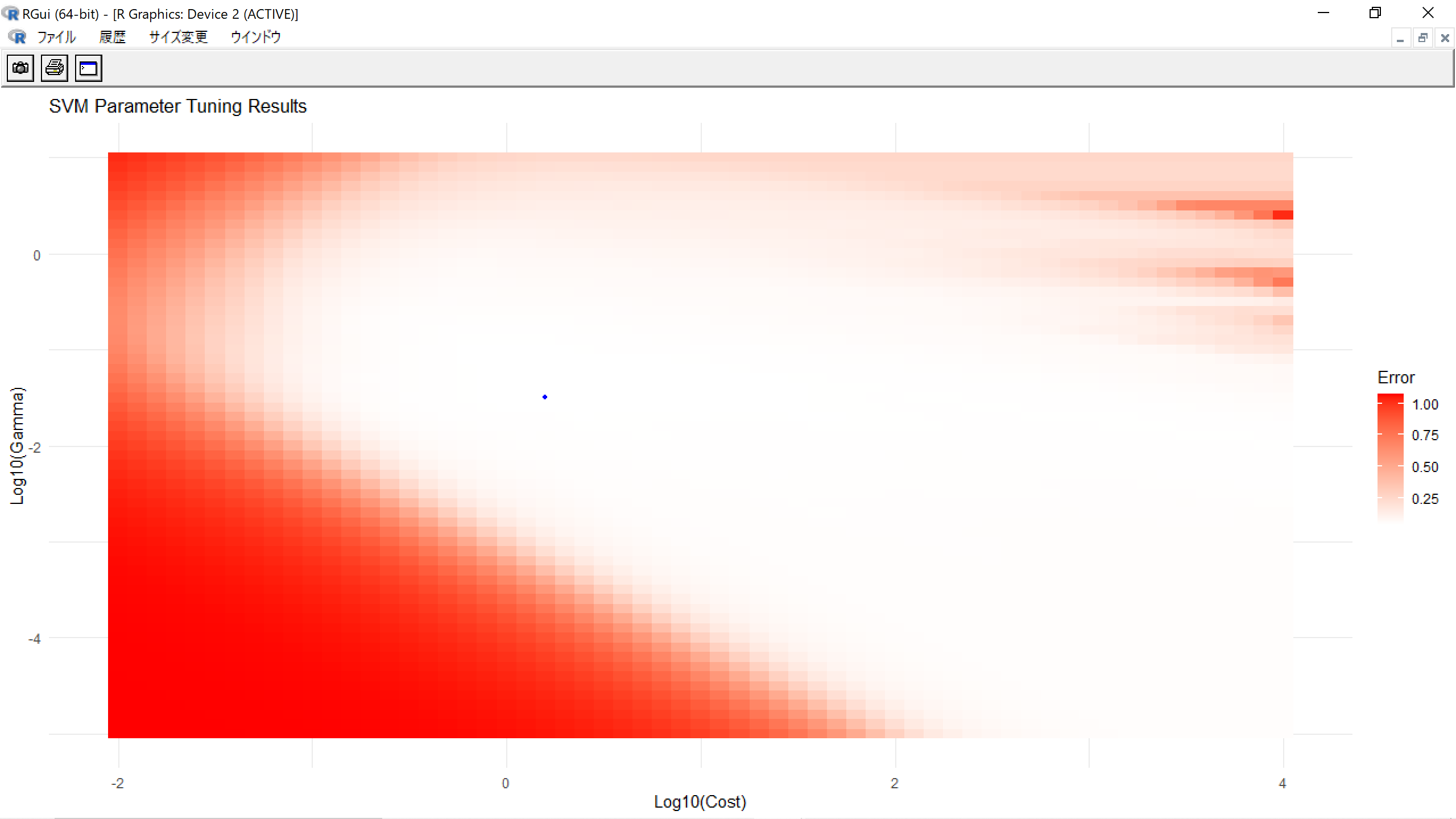


SVRM<-

svm(age~SLC12A5_1_methylation_rate_ave+SLC12A5_2_methylation_rate_ave+SLC12A5_4_methylation_rate_ave,　data=IBBS,

cost=best.cost, gamma=best.gamma, epsilon=0.1, scale = FALSE)

#LOOCV

nSamples<-nrow(IBBS)

predict_SVRM_loocv<-numeric(nSamples)

for (z in 1:nSamples){

indices<-removeOne(nSamples,z)

dr<-data.frame(IBBS$age[indices],IBBS$SLC12A5_1_methylation_rate_ave[indices],IBBS$SLC12A5_2_methylation_rate_ave[indices],IBBS$SLC12A5_4_methylation_rate_ave[indices])

colnames(dr)<-c("age","methylslc_1","methylslc_2","methylslc_4")

bestmodel_SVRM<-svm(age~methylslc_1+methylslc_2+methylslc_4, data=dr,

cost=best.cost, gamma= best.gamma, epsilon=0.1, scale = FALSE)

newdata<-data.frame(methylslc_1=IBBS$SLC12A5_1_methylation_rate_ave[z],methylslc_2=IBBS$SLC12A5_2_methylation_rate_ave[z],methylslc_4=IBBS$SLC12A5_4_methylation_rate_ave[z])

p<-predict(bestmodel_SVRM,newdata)*sd(AGE)+mean(AGE)

if (p<0){p=0}

predict_SVRM_loocv[z]<-p}

IBB_SVRM_loocv<-cbind(IBB,predict_SVRM_loocv)

MAE_SVRM_loocv<-mean(abs(IBB_SVRM_loocv$predict_SVRM_loocv-IBB$age))

MedianAE_SVRM_loocv<-median(abs(IBB_SVRM_loocv$predict_SVRM_loocv-IBB$age))

RMSE_SVRM_loocv<- sqrt(mean((IBB_SVRM_loocv$predict_SVRM_loocv-IBB$age)^2))

cat("MAE:", MAE_SVRM_loocv, "\nMed AE:", MedianAE_SVRM_loocv, "\nRMSE:", RMSE_SVRM_loocv, "\n")

MAE: 1.461942

Med AE: 1.043847

RMSE: 1.873955

g_SVRM_loocv_ss<-ggplot(IBB_SVRM_loocv,aes(age,predict_SVRM_loocv))+theme_bw()+

annotate("segment",x=min(IBB$age),xend=max(IBB$age),y=min(IBB$age)+1.461942,yend=max(IBB$age)+1.461942,colour="orchid4",linetype=2,linewidth =0.7)+

annotate("segment",x= min(IBB$age),xend=max(IBB$age), y=min(IBB$age)-1.461942,yend=max(IBB$age)-1.461942,colour="orchid4",linetype=2,linewidth =0.7)+

geom_point(aes(shape=sex,color=species),size=2,stroke=2)+

labs(x="Chronological age (year)",y="Predicted age (year)")+

scale_shape_manual(name="sex",labels=c("F" = "female", "M" = "male"),values=c("F" = 1, "M" = 3))+

scale_color_manual(name="species",labels=c("ABB" = "Asian black bear", "PB" = "Polar bear", "BB" = "Brown bear", "SB" = "Sun bear"), values = c("ABB" = "#66C2A5", "PB" = "#8DA0CB", "BB" = "#FC8D62", "SB" = "#E78AC3"))+

theme(axis.text.x=element_text(size=20),axis.text.y=element_text(size=20))+

theme(axis.title.x=element_text(size=17),axis.title.y=element_text(size=17))+

geom_line(aes(y =age), linewidth=1)+

labs(title="SVR model")+

theme(title=element_text(size=17),plot.title=element_text(hjust=0.5))+

scale_y_continuous(limits=c(-5,40))+

scale_x_continuous(limits=c(-5,40))+

labs(subtitle="SLC12A5-1, -2, -4")+

theme(plot.subtitle=element_text(size=15,hjust=0.5))+

guides(color = guide_legend(order = 1), shape = guide_legend(order = 2))


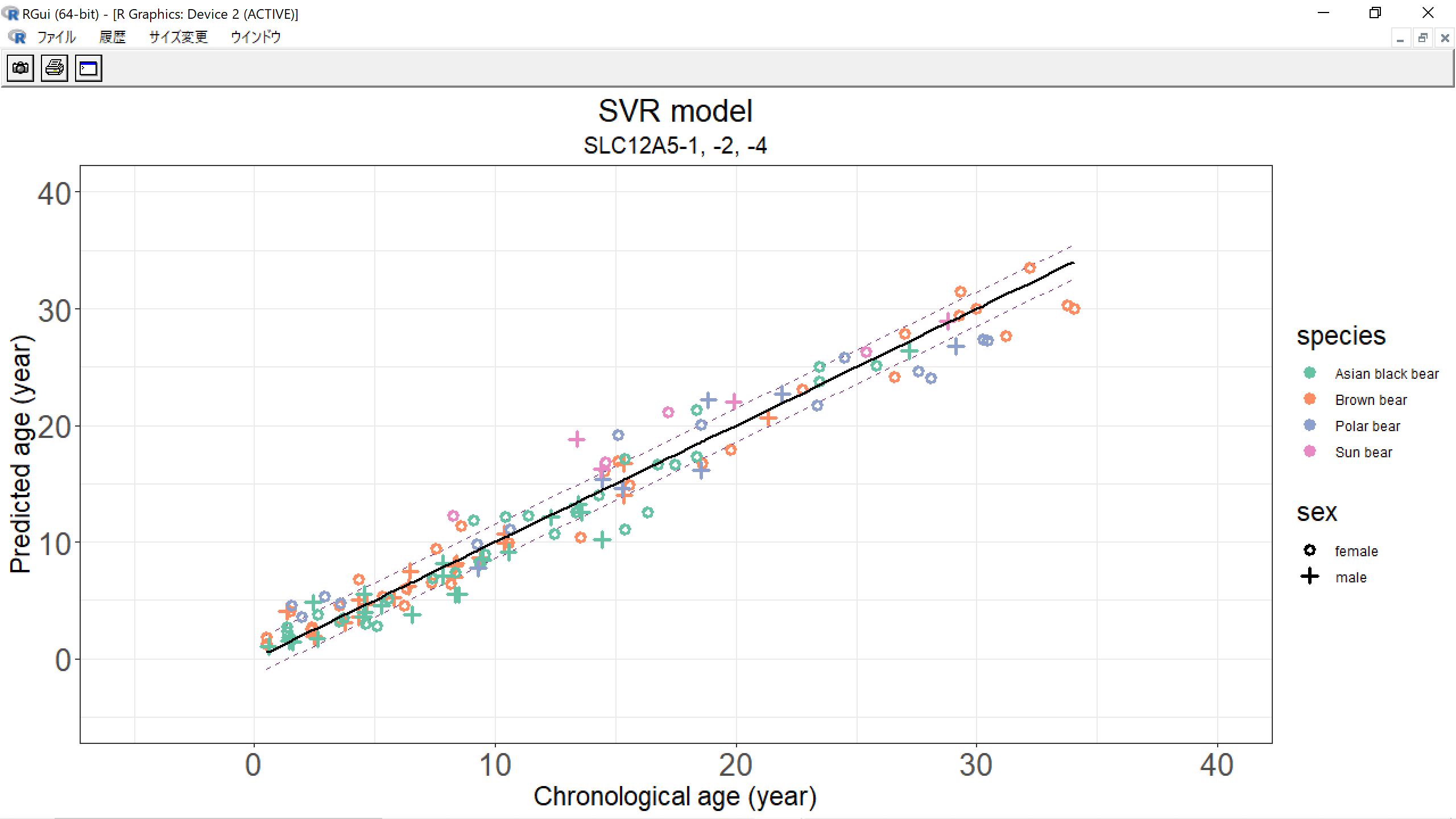


g_SVRM_loocv_se<-ggplot(IBB_SVRM_loocv,aes(age,predict_SVRM_loocv))+theme_bw()+

annotate("segment",x=min(IBB$age),xend=max(IBB$age),y=min(IBB$age)+1.461942,yend=max(IBB$age)+1.461942,colour="orchid4",linetype=2,linewidth =0.7)+

annotate("segment",x= min(IBB$age),xend=max(IBB$age), y=min(IBB$age)-1.461942,yend=max(IBB$age)-1.461942,colour="orchid4",linetype=2,linewidth =0.7)+

geom_point(aes(shape=environment,color=species),size=2,stroke=2)+

labs(x="Chronological age (year)",y="Predicted age (year)")+

scale_shape_manual(name="environment",labels=c("captive", "wild"),values=c(1,3))+

scale_color_manual(name="species",labels=c("ABB" = "Asian black bear", "PB" = "Polar bear", "BB" = "Brown bear", "SB" = "Sun bear"), values = c("ABB" = "#66C2A5", "PB" = "#8DA0CB", "BB" = "#FC8D62", "SB" = "#E78AC3"))+

theme(axis.text.x=element_text(size=20),axis.text.y=element_text(size=20))+

theme(axis.title.x=element_text(size=17),axis.title.y=element_text(size=17))+

geom_line(aes(y =age), linewidth=1)+

labs(title="SVR model")+

theme(title=element_text(size=17),plot.title=element_text(hjust=0.5))+

scale_y_continuous(limits=c(-5,40))+

scale_x_continuous(limits=c(-5,40))+

labs(subtitle="SLC12A5-1, -2, -4")+

theme(plot.subtitle=element_text(size=15,hjust=0.5))+

guides(color = guide_legend(order = 1), shape = guide_legend(order = 2))


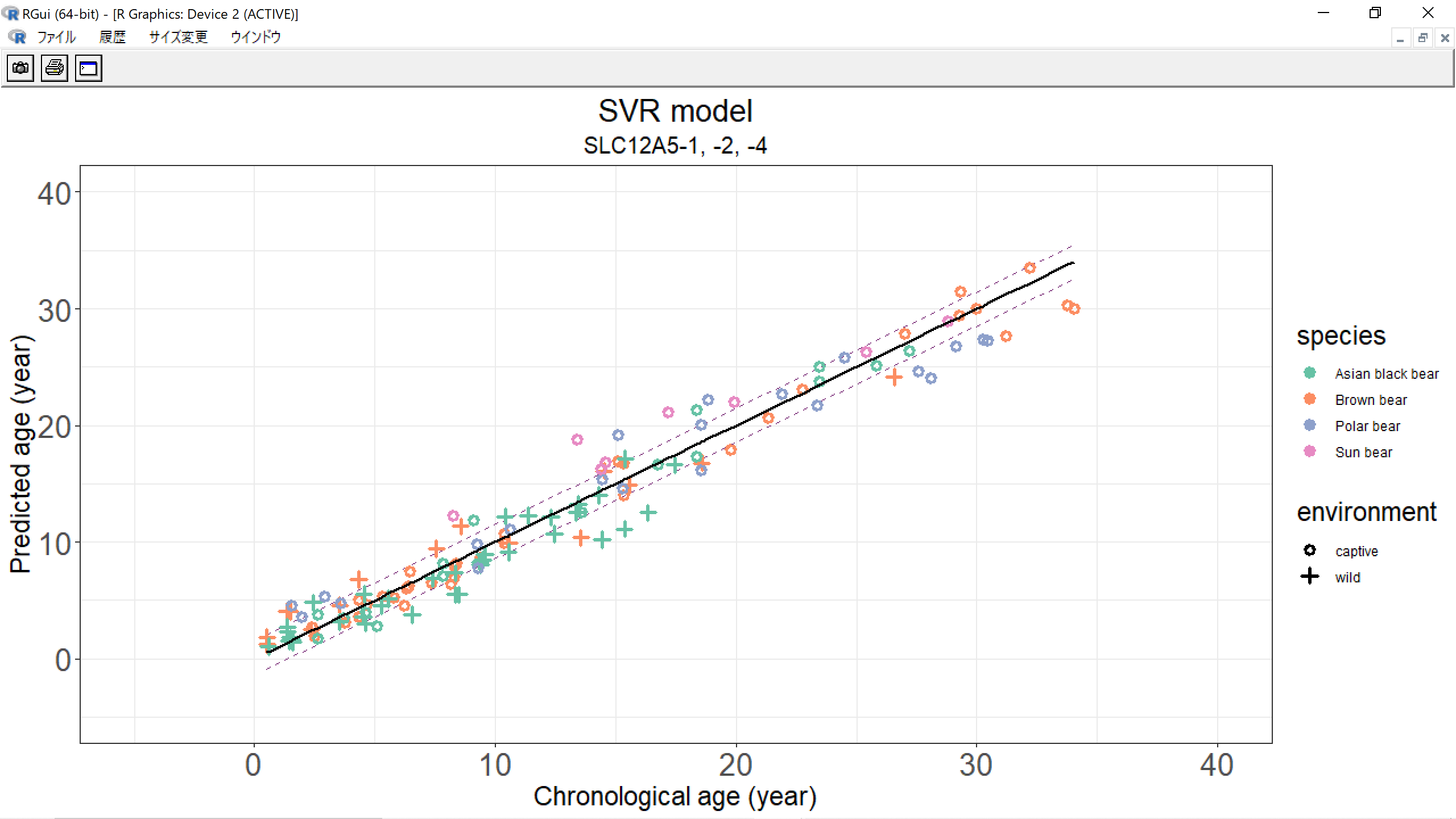


Support vector regression (SLC12A5-1, -3, -4)

set.seed(1)

tuneResult<-

tune(svm,age~SLC12A5_1_methylation_rate_ave+SLC12A5_3_methylation_rate_ave+SLC12A5_4_methylation_rate_ave,data=IBBS,

ranges=list(cost=10^(seq(-2,4,0.1)),gamma=10^(seq(-5,1,0.1))),

tunecontrol = tune.control(sampling = "cross", cross = 10), scale = FALSE)

tunedModel <- tuneResult$best.model

tunedModel

Call:

best.tune(METHOD = svm, train.x = age ~ SLC12A5_1_methylation_rate_ave +

SLC12A5_3_methylation_rate_ave + SLC12A5_4_methylation_rate_ave,

data = IBBS, ranges = list(cost = 10^(seq(-2, 4, 0.1)), gamma = 10^(seq(-5,

1, 0.1))), tunecontrol = tune.control(sampling = "cross",

cross = 10), scale = FALSE)

Parameters:

SVM-Type: eps-regression

SVM-Kernel: radial

cost: 5011.872

gamma: 0.01258925

epsilon: 0.1

Number of Support Vectors: 86

best.cost <- tunedModel$cost

best.gamma <- tunedModel$gamma

cat("Cost: ", best.cost, "\nGamma: ", best.gamma, "\n")

Cost: 5011.872

Gamma: 0.01258925

tune_results <- as.data.frame(tuneResult$performances)

tune_results$cost <- log10(tune_results$cost)

tune_results$gamma <- log10(tune_results$gamma)

ggplot(tune_results, aes(x = cost, y = gamma, fill = error)) +

geom_tile() +

geom_point(aes(x = log10(best.cost), y = log10(best.gamma)), color = "blue", size = 1, shape = 21, fill = "blue") +

scale_fill_gradient(low = "white", high = "red") +

labs(title = "SVM Parameter Tuning Results",

x = "Log10(Cost)",

y = "Log10(Gamma)",

fill = "Error") +

theme_minimal()


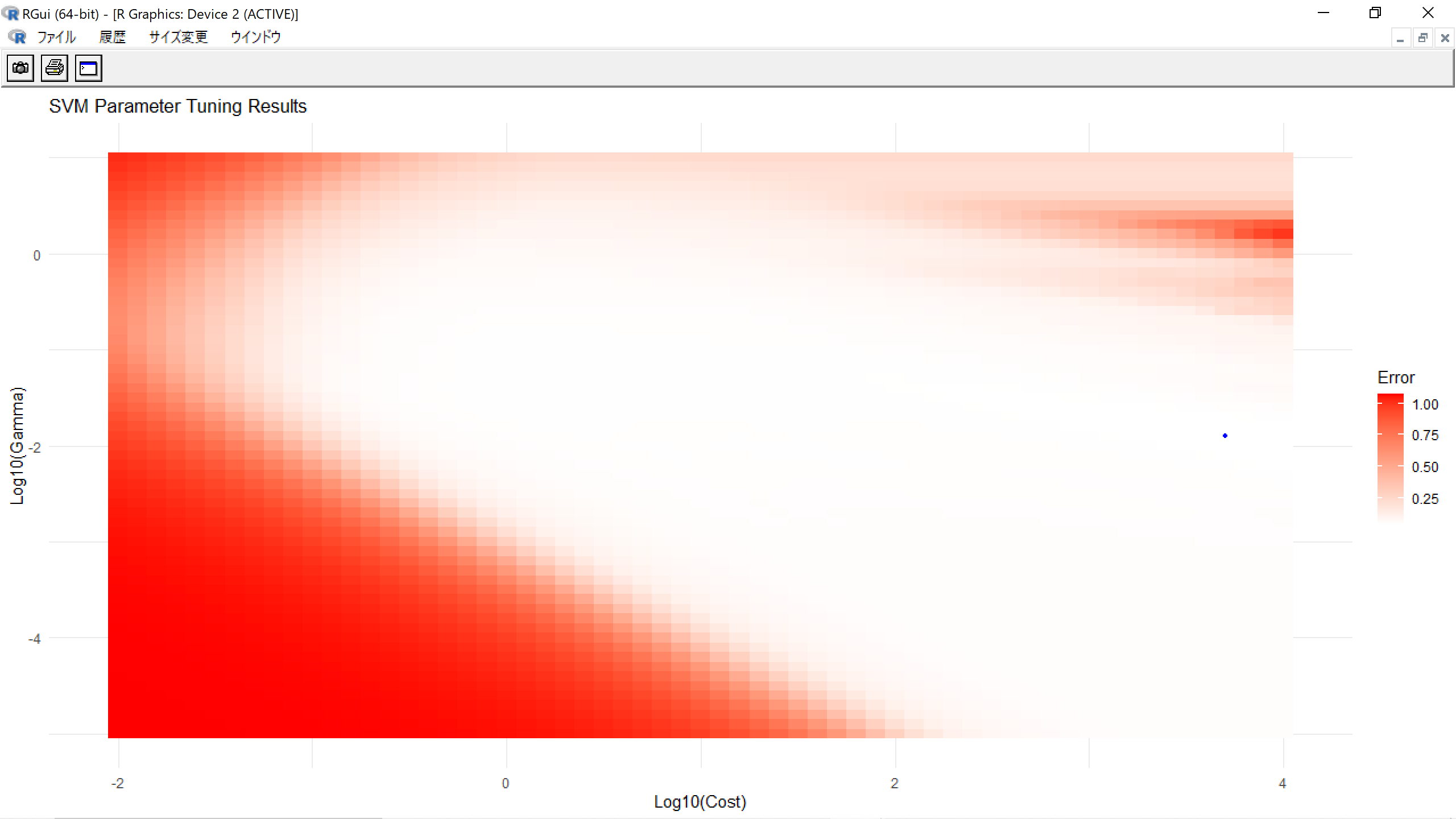


SVRM<-

svm(age~SLC12A5_1_methylation_rate_ave+SLC12A5_3_methylation_rate_ave+SLC12A5_4_methylation_rate_ave,　data=IBBS,

cost=best.cost, gamma=best.gamma, epsilon=0.1, scale = FALSE)

#LOOCV

nSamples<-nrow(IBBS)

predict_SVRM_loocv<-numeric(nSamples)

for (z in 1:nSamples){

indices<-removeOne(nSamples,z)

dr<-data.frame(IBBS$age[indices],IBBS$SLC12A5_1_methylation_rate_ave[indices],IBBS$SLC12A5_3_methylation_rate_ave[indices],IBBS$SLC12A5_4_methylation_rate_ave[indices])

colnames(dr)<-c("age","methylslc_1","methylslc_3","methylslc_4")

bestmodel_SVRM<-svm(age~methylslc_1+methylslc_3+methylslc_4, data=dr,

cost=best.cost, gamma= best.gamma, epsilon=0.1, scale = FALSE)

newdata<-data.frame(methylslc_1=IBBS$SLC12A5_1_methylation_rate_ave[z],methylslc_3=IBBS$SLC12A5_3_methylation_rate_ave[z],methylslc_4=IBBS$SLC12A5_4_methylation_rate_ave[z])

p<-predict(bestmodel_SVRM,newdata)*sd(AGE)+mean(AGE)

if (p<0){p=0}

predict_SVRM_loocv[z]<-p}

IBB_SVRM_loocv<-cbind(IBB,predict_SVRM_loocv)

MAE_SVRM_loocv<-mean(abs(IBB_SVRM_loocv$predict_SVRM_loocv-IBB$age))

MedianAE_SVRM_loocv<-median(abs(IBB_SVRM_loocv$predict_SVRM_loocv-IBB$age))

RMSE_SVRM_loocv<- sqrt(mean((IBB_SVRM_loocv$predict_SVRM_loocv-IBB$age)^2))

cat("MAE:", MAE_SVRM_loocv, "\nMed AE:", MedianAE_SVRM_loocv, "\nRMSE:", RMSE_SVRM_loocv, "\n")

MAE: 1.455357

Med AE: 1.183261

RMSE: 1.807348

g_SVRM_loocv_ss<-ggplot(IBB_SVRM_loocv,aes(age,predict_SVRM_loocv))+theme_bw()+

annotate("segment",x=min(IBB$age),xend=max(IBB$age),y=min(IBB$age)+1.455357,yend=max(IBB$age)+1.455357,colour="orchid4",linetype=2,linewidth =0.7)+

annotate("segment",x= min(IBB$age),xend=max(IBB$age), y=min(IBB$age)-1.455357,yend=max(IBB$age)-1.455357,colour="orchid4",linetype=2,linewidth =0.7)+

geom_point(aes(shape=sex,color=species),size=2,stroke=2)+

labs(x="Chronological age (year)",y="Predicted age (year)")+

scale_shape_manual(name="sex",labels=c("F" = "female", "M" = "male"),values=c("F" = 1, "M" = 3))+

scale_color_manual(name="species",labels=c("ABB" = "Asian black bear", "PB" = "Polar bear", "BB" = "Brown bear", "SB" = "Sun bear"), values = c("ABB" = "#66C2A5", "PB" = "#8DA0CB", "BB" = "#FC8D62", "SB" = "#E78AC3"))+

theme(axis.text.x=element_text(size=20),axis.text.y=element_text(size=20))+

theme(axis.title.x=element_text(size=17),axis.title.y=element_text(size=17))+

geom_line(aes(y =age), linewidth=1)+

labs(title="SVR model")+

theme(title=element_text(size=17),plot.title=element_text(hjust=0.5))+

scale_y_continuous(limits=c(-5,40))+

scale_x_continuous(limits=c(-5,40))+

labs(subtitle="SLC12A5-1, -3, -4")+

theme(plot.subtitle=element_text(size=15,hjust=0.5))+

guides(color = guide_legend(order = 1), shape = guide_legend(order = 2))


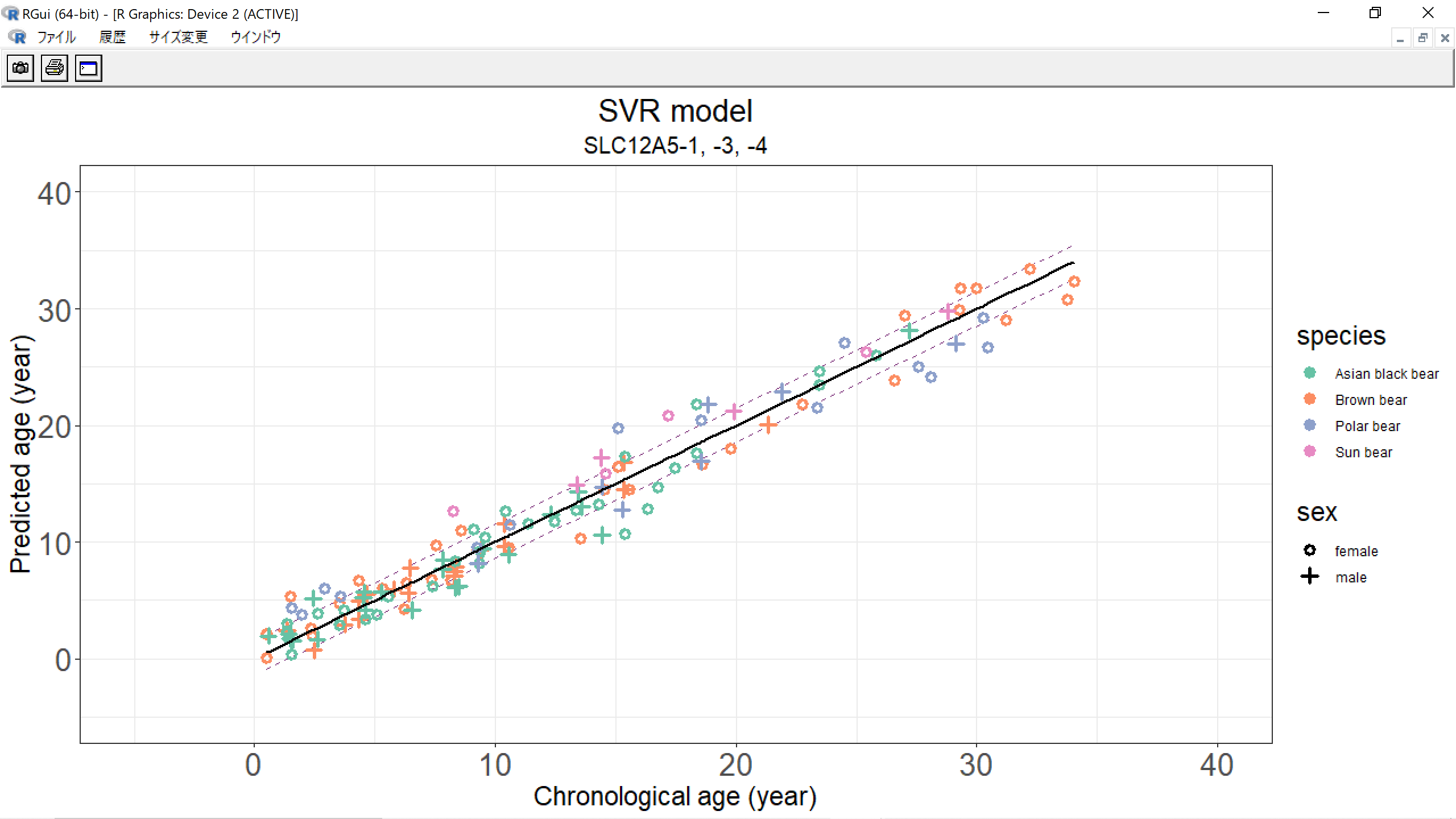


g_SVRM_loocv_se<-ggplot(IBB_SVRM_loocv,aes(age,predict_SVRM_loocv))+theme_bw()+

annotate("segment",x=min(IBB$age),xend=max(IBB$age),y=min(IBB$age)+1.455357,yend=max(IBB$age)+1.455357,colour="orchid4",linetype=2,linewidth =0.7)+

annotate("segment",x= min(IBB$age),xend=max(IBB$age), y=min(IBB$age)-1.455357,yend=max(IBB$age)-1.455357,colour="orchid4",linetype=2,linewidth =0.7)+

geom_point(aes(shape=environment,color=species),size=2,stroke=2)+

labs(x="Chronological age (year)",y="Predicted age (year)")+

scale_shape_manual(name="environment",labels=c("captive", "wild"),values=c(1,3))+

scale_color_manual(name="species",labels=c("ABB" = "Asian black bear", "PB" = "Polar bear", "BB" = "Brown bear", "SB" = "Sun bear"), values = c("ABB" = "#66C2A5", "PB" = "#8DA0CB", "BB" = "#FC8D62", "SB" = "#E78AC3"))+

theme(axis.text.x=element_text(size=20),axis.text.y=element_text(size=20))+

theme(axis.title.x=element_text(size=17),axis.title.y=element_text(size=17))+

geom_line(aes(y =age), linewidth=1)+

labs(title="SVR model")+

theme(title=element_text(size=17),plot.title=element_text(hjust=0.5))+

scale_y_continuous(limits=c(-5,40))+

scale_x_continuous(limits=c(-5,40))+

labs(subtitle="SLC12A5-1, -3, -4")+

theme(plot.subtitle=element_text(size=15,hjust=0.5))+

guides(color = guide_legend(order = 1), shape = guide_legend(order = 2))


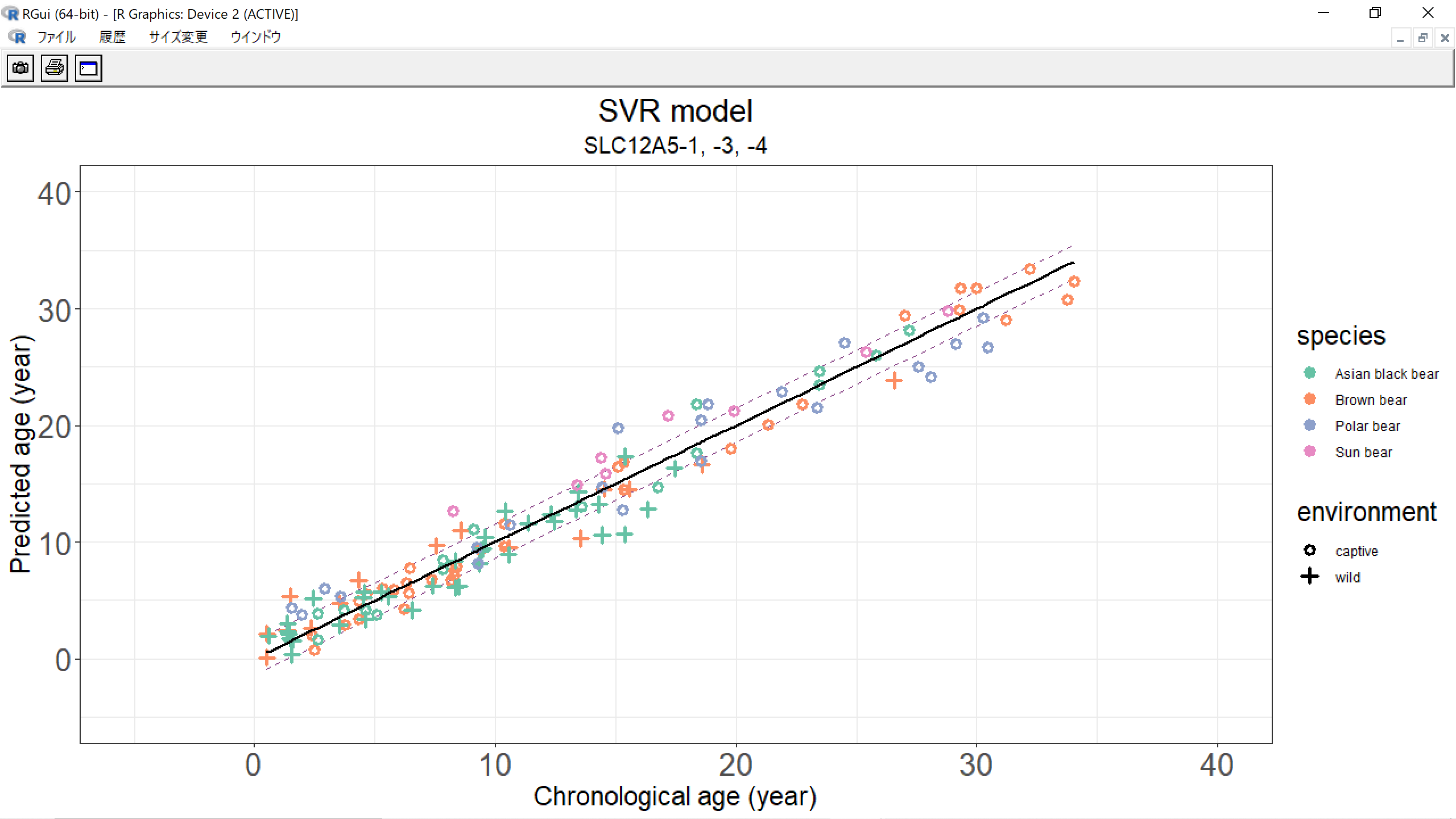


Support vector regression (SLC12A5-2, -3, -4)

set.seed(1)

tuneResult<-

tune(svm,age~SLC12A5_2_methylation_rate_ave+SLC12A5_3_methylation_rate_ave+SLC12A5_4_methylation_rate_ave,data=IBBS,

ranges=list(cost=10^(seq(-2,4,0.1)),gamma=10^(seq(-5,1,0.1))),

tunecontrol = tune.control(sampling = "cross", cross = 10), scale = FALSE)

tunedModel <- tuneResult$best.model

tunedModel

Call:

best.tune(METHOD = svm, train.x = age ~ SLC12A5_2_methylation_rate_ave +

SLC12A5_3_methylation_rate_ave + SLC12A5_4_methylation_rate_ave,

data = IBBS, ranges = list(cost = 10^(seq(-2, 4, 0.1)), gamma = 10^(seq(-5,

1, 0.1))), tunecontrol = tune.control(sampling = "cross",

cross = 10), scale = FALSE)

Parameters:

SVM-Type: eps-regression

SVM-Kernel: radial

cost: 2.511886

gamma: 0.1

epsilon: 0.1

Number of Support Vectors: 73

best.cost <- tunedModel$cost

best.gamma <- tunedModel$gamma

cat("Cost: ", best.cost, "\nGamma: ", best.gamma, "\n")

Cost: 2.511886

Gamma: 0.1

tune_results <- as.data.frame(tuneResult$performances)

tune_results$cost <- log10(tune_results$cost)

tune_results$gamma <- log10(tune_results$gamma)

ggplot(tune_results, aes(x = cost, y = gamma, fill = error)) +

geom_tile() +

geom_point(aes(x = log10(best.cost), y = log10(best.gamma)), color = "blue", size = 1, shape = 21, fill = "blue") +

scale_fill_gradient(low = "white", high = "red") +

labs(title = "SVM Parameter Tuning Results",

x = "Log10(Cost)",

y = "Log10(Gamma)",

fill = "Error") +

theme_minimal()


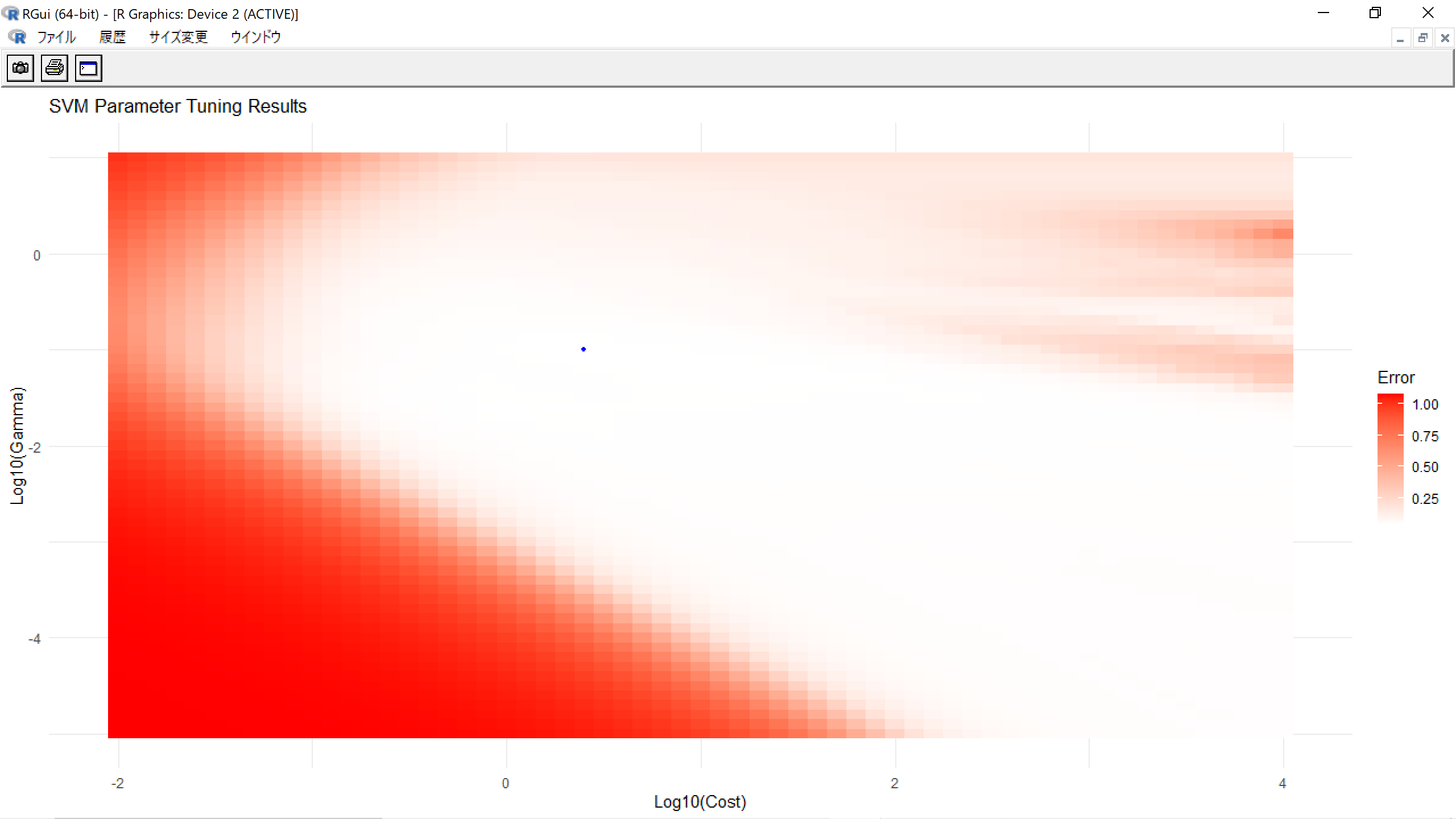


SVRM<-

svm(age~SLC12A5_2_methylation_rate_ave+SLC12A5_3_methylation_rate_ave+SLC12A5_4_methylation_rate_ave,　data=IBBS,

cost=best.cost, gamma=best.gamma, epsilon=0.1, scale = FALSE)

#LOOCV

nSamples<-nrow(IBBS)

predict_SVRM_loocv<-numeric(nSamples)

for (z in 1:nSamples){

indices<-removeOne(nSamples,z)

dr<-data.frame(IBBS$age[indices],IBBS$SLC12A5_2_methylation_rate_ave[indices],IBBS$SLC12A5_3_methylation_rate_ave[indices],IBBS$SLC12A5_4_methylation_rate_ave[indices])

colnames(dr)<-c("age","methylslc_2","methylslc_3","methylslc_4")

bestmodel_SVRM<-svm(age~ methylslc_2+methylslc_3+methylslc_4, data=dr,

cost=best.cost, gamma= best.gamma, epsilon=0.1, scale = FALSE)

newdata<-data.frame(methylslc_2=IBBS$SLC12A5_2_methylation_rate_ave[z],methylslc_3=IBBS$SLC12A5_3_methylation_rate_ave[z],methylslc_4=IBBS$SLC12A5_4_methylation_rate_ave[z])

p<-predict(bestmodel_SVRM,newdata)*sd(AGE)+mean(AGE)

if (p<0){p=0}

predict_SVRM_loocv[z]<-p}

IBB_SVRM_loocv<-cbind(IBB,predict_SVRM_loocv)

MAE_SVRM_loocv<-mean(abs(IBB_SVRM_loocv$predict_SVRM_loocv-IBB$age))

MedianAE_SVRM_loocv<-median(abs(IBB_SVRM_loocv$predict_SVRM_loocv-IBB$age))

RMSE_SVRM_loocv<- sqrt(mean((IBB_SVRM_loocv$predict_SVRM_loocv-IBB$age)^2))

cat("MAE:", MAE_SVRM_loocv, "\nMed AE:", MedianAE_SVRM_loocv, "\nRMSE:", RMSE_SVRM_loocv, "\n")

MAE: 1.468971

Med AE: 1.121346

RMSE: 1.892641

Support vector regression (SLC12A5-1, -2)

set.seed(1)

tuneResult<-

tune(svm,age~SLC12A5_1_methylation_rate_ave+SLC12A5_2_methylation_rate_ave,data=IBBS,

ranges=list(cost=10^(seq(-2,4,0.1)),gamma=10^(seq(-5,1,0.1))),

tunecontrol = tune.control(sampling = "cross", cross = 10), scale = FALSE)

tunedModel <- tuneResult$best.model

tunedModel

Call:

best.tune(METHOD = svm, train.x = age ~ SLC12A5_1_methylation_rate_ave +

SLC12A5_2_methylation_rate_ave, data = IBBS, ranges = list(cost = 10^(seq(-2,

4, 0.1)), gamma = 10^(seq(-5, 1, 0.1))), tunecontrol = tune.control(sampling = "cross",

cross = 10), scale = FALSE)

Parameters:

SVM-Type: eps-regression

SVM-Kernel: radial

cost: 12.58925

gamma: 0.07943282

epsilon: 0.1

Number of Support Vectors: 84

best.cost <- tunedModel$cost

best.gamma <- tunedModel$gamma

cat("Cost: ", best.cost, "\nGamma: ", best.gamma, "\n")

Cost: 12.58925

Gamma: 0.07943282

tune_results <- as.data.frame(tuneResult$performances)

tune_results$cost <- log10(tune_results$cost)

tune_results$gamma <- log10(tune_results$gamma)

ggplot(tune_results, aes(x = cost, y = gamma, fill = error)) +

geom_tile() +

geom_point(aes(x = log10(best.cost), y = log10(best.gamma)), color = "blue", size = 1, shape = 21, fill = "blue") +

scale_fill_gradient(low = "white", high = "red") +

labs(title = "SVM Parameter Tuning Results",

x = "Log10(Cost)",

y = "Log10(Gamma)",

fill = "Error") +

theme_minimal()


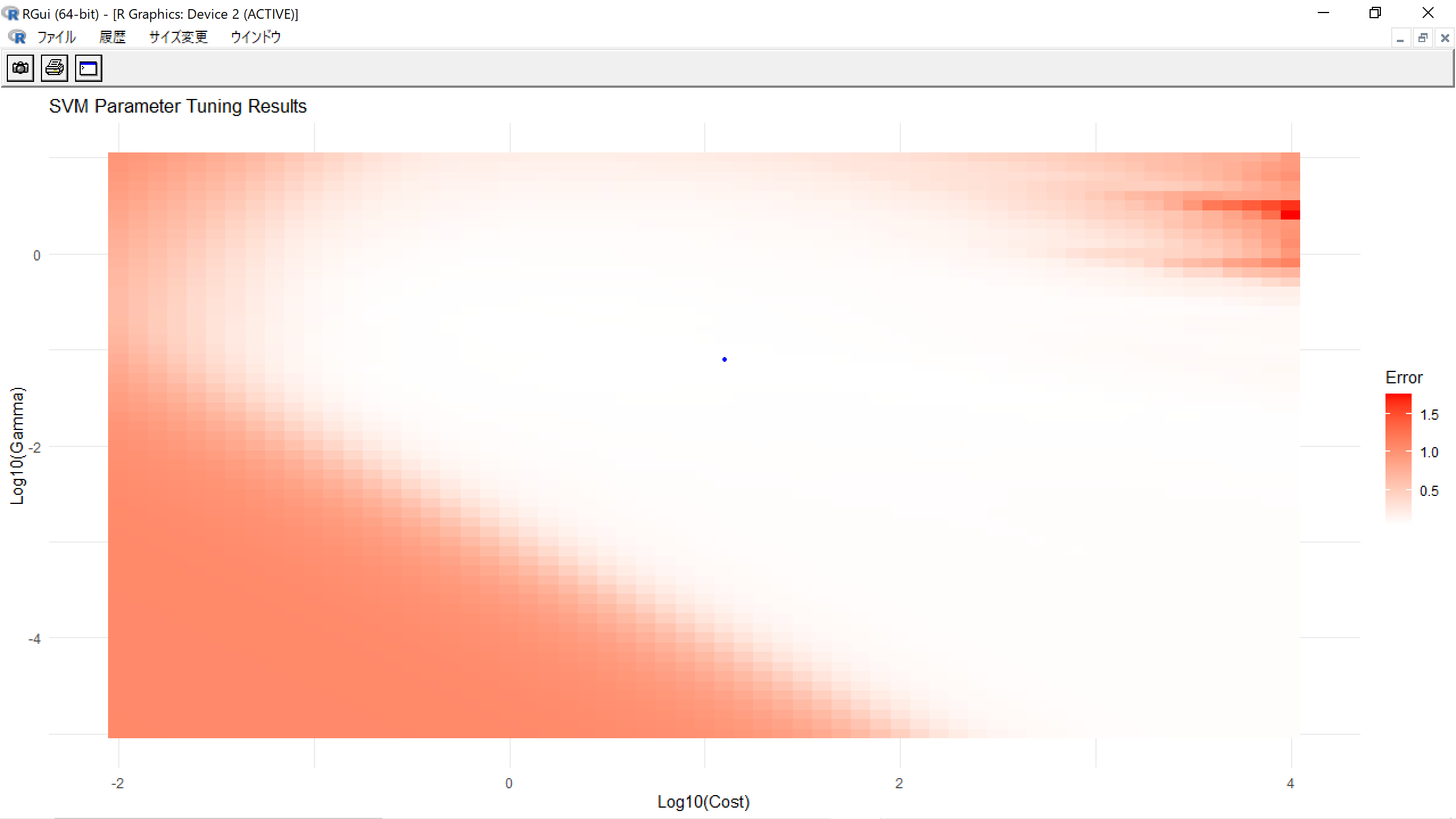


SVRM<-

svm(age~SLC12A5_1_methylation_rate_ave+SLC12A5_2_methylation_rate_ave,　data=IBBS,

cost=best.cost, gamma=best.gamma, epsilon=0.1, scale = FALSE)

#LOOCV

nSamples<-nrow(IBBS)

predict_SVRM_loocv<-numeric(nSamples)

for (z in 1:nSamples){

indices<-removeOne(nSamples,z)

dr<-data.frame(IBBS$age[indices],IBBS$SLC12A5_1_methylation_rate_ave[indices],IBBS$SLC12A5_2_methylation_rate_ave[indices])

colnames(dr)<-c("age","methylslc_1","methylslc_2")

bestmodel_SVRM<-svm(age~methylslc_1+methylslc_2, data=dr,

cost=best.cost, gamma= best.gamma, epsilon=0.1, scale = FALSE)

newdata<-data.frame(methylslc_1=IBBS$SLC12A5_1_methylation_rate_ave[z],methylslc_2=IBBS$SLC12A5_2_methylation_rate_ave[z])

p<-predict(bestmodel_SVRM,newdata)*sd(AGE)+mean(AGE)

if (p<0){p=0}

predict_SVRM_loocv[z]<-p}

IBB_SVRM_loocv<-cbind(IBB,predict_SVRM_loocv)

MAE_SVRM_loocv<-mean(abs(IBB_SVRM_loocv$predict_SVRM_loocv-IBB$age))

MedianAE_SVRM_loocv<-median(abs(IBB_SVRM_loocv$predict_SVRM_loocv-IBB$age))

RMSE_SVRM_loocv<- sqrt(mean((IBB_SVRM_loocv$predict_SVRM_loocv-IBB$age)^2))

cat("MAE:", MAE_SVRM_loocv, "\nMed AE:", MedianAE_SVRM_loocv, "\nRMSE:", RMSE_SVRM_loocv, "\n")

MAE: 1.667281

Med AE: 1.424805

RMSE: 2.102992

Support vector regression (SLC12A5-1, -3)

set.seed(1)

tuneResult<-

tune(svm,age~SLC12A5_1_methylation_rate_ave+SLC12A5_3_methylation_rate_ave,data=IBBS,

ranges=list(cost=10^(seq(-2,4,0.1)),gamma=10^(seq(-5,1,0.1))),

tunecontrol = tune.control(sampling = "cross", cross = 10), scale = FALSE)

tunedModel <- tuneResult$best.model

tunedModel

Call:

best.tune(METHOD = svm, train.x = age ~ SLC12A5_1_methylation_rate_ave +

SLC12A5_3_methylation_rate_ave, data = IBBS, ranges = list(cost = 10^(seq(-2,

4, 0.1)), gamma = 10^(seq(-5, 1, 0.1))), tunecontrol = tune.control(sampling = "cross",

cross = 10), scale = FALSE)

Parameters:

SVM-Type: eps-regression

SVM-Kernel: radial

cost: 1.995262

gamma: 0.1258925

epsilon: 0.1

Number of Support Vectors: 91

best.cost <- tunedModel$cost

best.gamma <- tunedModel$gamma

cat("Cost: ", best.cost, "\nGamma: ", best.gamma, "\n")

Cost: 1.995262

Gamma: 0.1258925

tune_results <- as.data.frame(tuneResult$performances)

tune_results$cost <- log10(tune_results$cost)

tune_results$gamma <- log10(tune_results$gamma)

ggplot(tune_results, aes(x = cost, y = gamma, fill = error)) +

geom_tile() +

geom_point(aes(x = log10(best.cost), y = log10(best.gamma)), color = "blue", size = 1, shape = 21, fill = "blue") +

scale_fill_gradient(low = "white", high = "red") +

labs(title = "SVM Parameter Tuning Results",

x = "Log10(Cost)",

y = "Log10(Gamma)",

fill = "Error") +

theme_minimal()


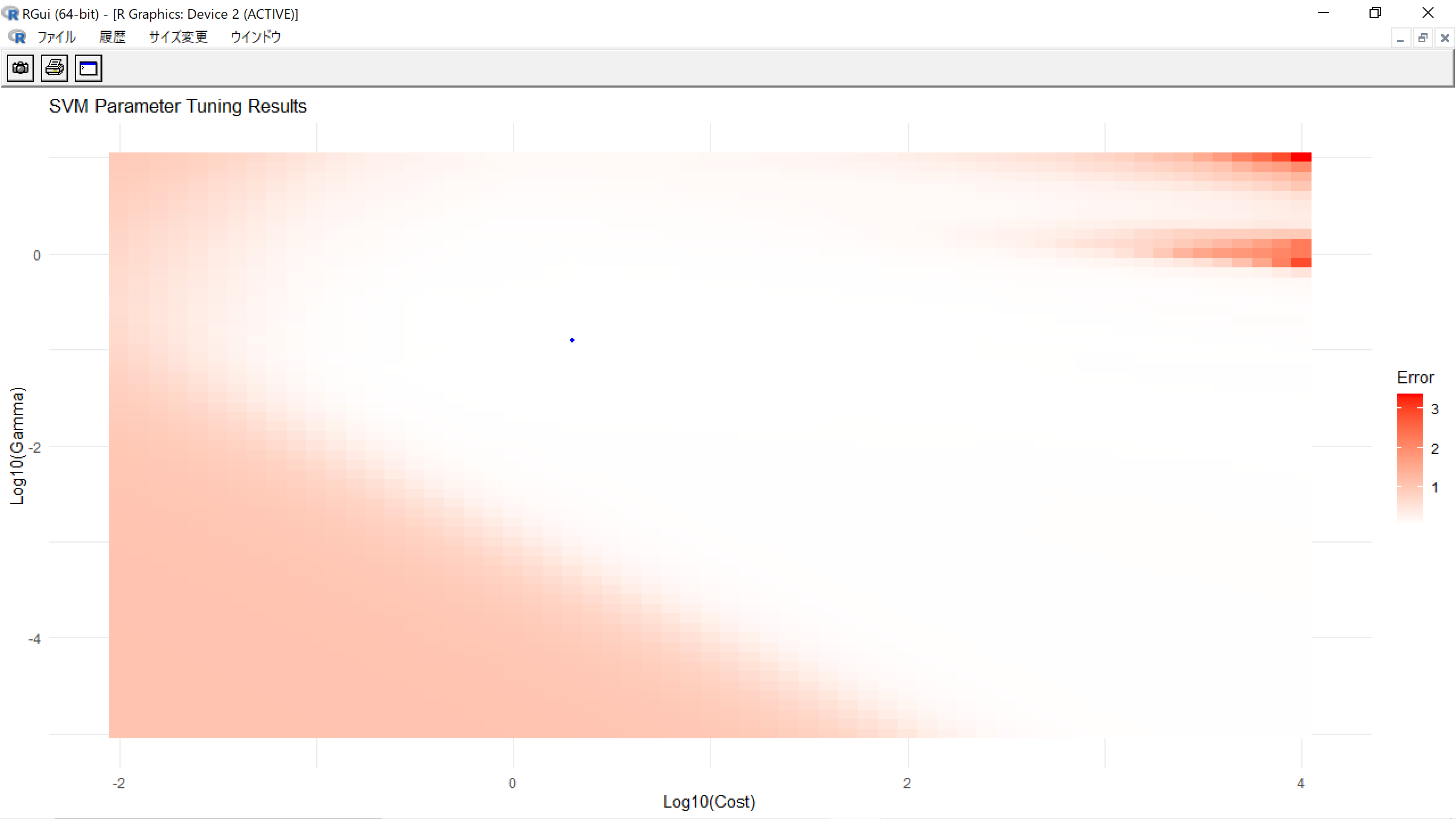


SVRM<-

svm(age~SLC12A5_1_methylation_rate_ave+SLC12A5_3_methylation_rate_ave,　data=IBBS,

cost=best.cost, gamma=best.gamma, epsilon=0.1, scale = FALSE)

#LOOCV

nSamples<-nrow(IBBS)

predict_SVRM_loocv<-numeric(nSamples)

for (z in 1:nSamples){

indices<-removeOne(nSamples,z)

dr<-data.frame(IBBS$age[indices],IBBS$SLC12A5_1_methylation_rate_ave[indices],IBBS$SLC12A5_3_methylation_rate_ave[indices])

colnames(dr)<-c("age","methylslc_1","methylslc_3")

bestmodel_SVRM<-svm(age~methylslc_1+methylslc_3, data=dr,

cost=best.cost, gamma= best.gamma, epsilon=0.1, scale = FALSE)

newdata<-data.frame(methylslc_1=IBBS$SLC12A5_1_methylation_rate_ave[z],methylslc_2=IBBS$SLC12A5_2_methylation_rate_ave[z],methylslc_3=IBBS$SLC12A5_3_methylation_rate_ave[z],methylslc_4=IBBS$SLC12A5_4_methylation_rate_ave[z])

p<-predict(bestmodel_SVRM,newdata)*sd(AGE)+mean(AGE)

if (p<0){p=0}

predict_SVRM_loocv[z]<-p}

IBB_SVRM_loocv<-cbind(IBB,predict_SVRM_loocv)

MAE_SVRM_loocv<-mean(abs(IBB_SVRM_loocv$predict_SVRM_loocv-IBB$age))

MedianAE_SVRM_loocv<-median(abs(IBB_SVRM_loocv$predict_SVRM_loocv-IBB$age))

RMSE_SVRM_loocv<- sqrt(mean((IBB_SVRM_loocv$predict_SVRM_loocv-IBB$age)^2))

cat("MAE:", MAE_SVRM_loocv, "\nMed AE:", MedianAE_SVRM_loocv, "\nRMSE:", RMSE_SVRM_loocv, "\n")

MAE: 1.754572

Med AE: 1.383027

RMSE: 2.267265

Support vector regression (SLC12A5-1, -4)

set.seed(1)

tuneResult<-

tune(svm,age~SLC12A5_1_methylation_rate_ave+SLC12A5_4_methylation_rate_ave,data=IBBS,

ranges=list(cost=10^(seq(-2,4,0.1)),gamma=10^(seq(-5,1,0.1))),

tunecontrol = tune.control(sampling = "cross", cross = 10), scale = FALSE)

tunedModel <- tuneResult$best.model

tunedModel

Call:

best.tune(METHOD = svm, train.x = age ~ SLC12A5_1_methylation_rate_ave +

SLC12A5_4_methylation_rate_ave, data = IBBS, ranges = list(cost = 10^(seq(-2,

4, 0.1)), gamma = 10^(seq(-5, 1, 0.1))), tunecontrol = tune.control(sampling = "cross",

cross = 10), scale = FALSE)

Parameters:

SVM-Type: eps-regression

SVM-Kernel: radial

cost: 7943.282

gamma: 0.01258925

epsilon: 0.1

Number of Support Vectors: 82

best.cost <- tunedModel$cost

best.gamma <- tunedModel$gamma

cat("Cost: ", best.cost, "\nGamma: ", best.gamma, "\n")

Cost: 7943.282

Gamma: 0.01258925

tune_results <- as.data.frame(tuneResult$performances)

tune_results$cost <- log10(tune_results$cost)

tune_results$gamma <- log10(tune_results$gamma)

ggplot(tune_results, aes(x = cost, y = gamma, fill = error)) +

geom_tile() +

geom_point(aes(x = log10(best.cost), y = log10(best.gamma)), color = "blue", size = 1, shape = 21, fill = "blue") +

scale_fill_gradient(low = "white", high = "red") +

labs(title = "SVM Parameter Tuning Results",

x = "Log10(Cost)",

y = "Log10(Gamma)",

fill = "Error") +

theme_minimal()


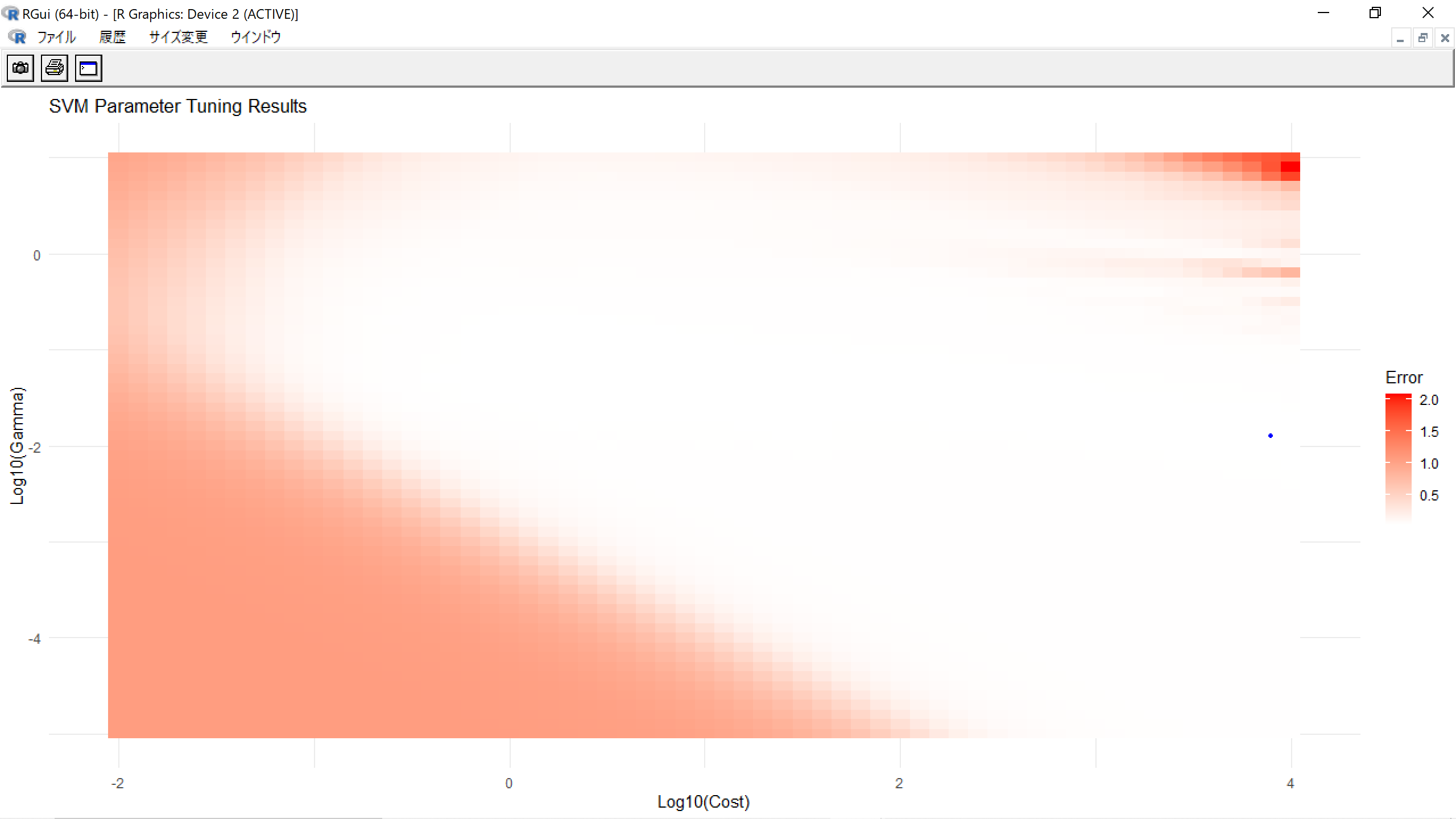


SVRM<-

svm(age~SLC12A5_1_methylation_rate_ave+SLC12A5_4_methylation_rate_ave,　data=IBBS,

cost=best.cost, gamma=best.gamma, epsilon=0.1, scale = FALSE)

#LOOCV

nSamples<-nrow(IBBS)

predict_SVRM_loocv<-numeric(nSamples)

for (z in 1:nSamples){

indices<-removeOne(nSamples,z)

dr<-data.frame(IBBS$age[indices],IBBS$SLC12A5_1_methylation_rate_ave[indices],IBBS$SLC12A5_4_methylation_rate_ave[indices])

colnames(dr)<-c("age","methylslc_1","methylslc_4")

bestmodel_SVRM<-svm(age~methylslc_1+methylslc_4, data=dr,

cost=best.cost, gamma= best.gamma, epsilon=0.1, scale = FALSE)

newdata<-data.frame(methylslc_1=IBBS$SLC12A5_1_methylation_rate_ave[z],methylslc_4=IBBS$SLC12A5_4_methylation_rate_ave[z])

p<-predict(bestmodel_SVRM,newdata)*sd(AGE)+mean(AGE)

if (p<0){p=0}

predict_SVRM_loocv[z]<-p}

IBB_SVRM_loocv<-cbind(IBB,predict_SVRM_loocv)

MAE_SVRM_loocv<-mean(abs(IBB_SVRM_loocv$predict_SVRM_loocv-IBB$age))

MedianAE_SVRM_loocv<-median(abs(IBB_SVRM_loocv$predict_SVRM_loocv-IBB$age))

RMSE_SVRM_loocv<- sqrt(mean((IBB_SVRM_loocv$predict_SVRM_loocv-IBB$age)^2))

cat("MAE:", MAE_SVRM_loocv, "\nMed AE:", MedianAE_SVRM_loocv, "\nRMSE:", RMSE_SVRM_loocv, "\n")

MAE: 1.492359

Med AE: 1.203801

RMSE: 1.898139

Support vector regression (SLC12A5-2, -3)

set.seed(1)

tuneResult<-

tune(svm,age~SLC12A5_2_methylation_rate_ave+SLC12A5_3_methylation_rate_ave,data=IBBS,

ranges=list(cost=10^(seq(-2,4,0.1)),gamma=10^(seq(-5,1,0.1))),

tunecontrol = tune.control(sampling = "cross", cross = 10), scale = FALSE)

tunedModel <- tuneResult$best.model

tunedModel

Call:

best.tune(METHOD = svm, train.x = age ~ SLC12A5_2_methylation_rate_ave +

SLC12A5_3_methylation_rate_ave, data = IBBS, ranges = list(cost = 10^(seq(-2,

4, 0.1)), gamma = 10^(seq(-5, 1, 0.1))), tunecontrol = tune.control(sampling = "cross",

cross = 10), scale = FALSE)

Parameters:

SVM-Type: eps-regression

SVM-Kernel: radial

cost: 1584.893

gamma: 0.02511886

epsilon: 0.1

Number of Support Vectors: 85

best.cost <- tunedModel$cost

best.gamma <- tunedModel$gamma

cat("Cost: ", best.cost, "\nGamma: ", best.gamma, "\n")

Cost: 1584.893

Gamma: 0.02511886

tune_results <- as.data.frame(tuneResult$performances)

tune_results$cost <- log10(tune_results$cost)

tune_results$gamma <- log10(tune_results$gamma)

ggplot(tune_results, aes(x = cost, y = gamma, fill = error)) +

geom_tile() +

geom_point(aes(x = log10(best.cost), y = log10(best.gamma)), color = "blue", size = 1, shape = 21, fill = "blue") +

scale_fill_gradient(low = "white", high = "red") +

labs(title = "SVM Parameter Tuning Results",

x = "Log10(Cost)",

y = "Log10(Gamma)",

fill = "Error") +

theme_minimal()


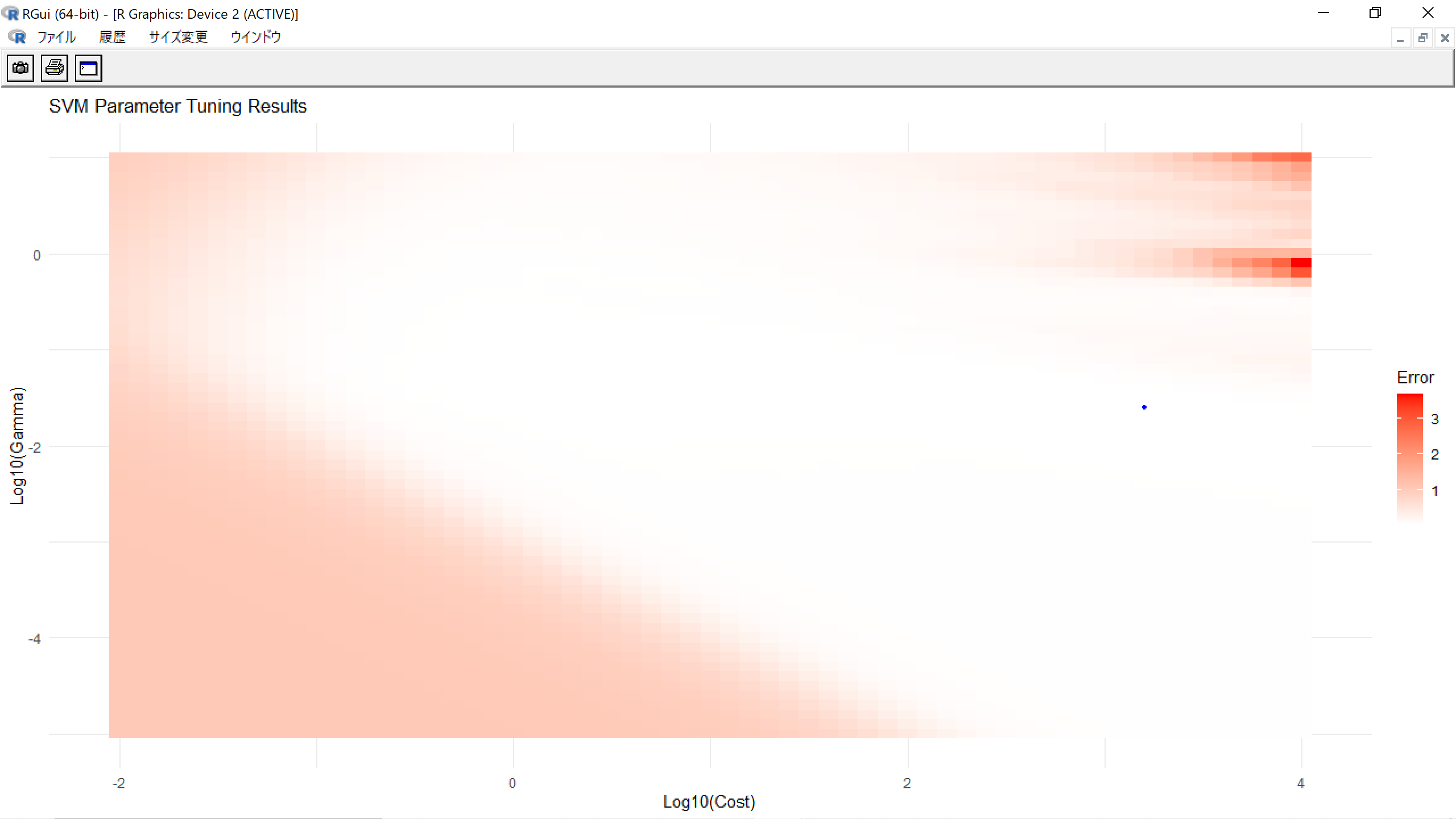


SVRM<-

svm(age~SLC12A5_2_methylation_rate_ave+SLC12A5_3_methylation_rate_ave,　data=IBBS,

cost=best.cost, gamma=best.gamma, epsilon=0.1, scale = FALSE)

#LOOCV

nSamples<-nrow(IBBS)

predict_SVRM_loocv<-numeric(nSamples)

for (z in 1:nSamples){

indices<-removeOne(nSamples,z)

dr<-data.frame(IBBS$age[indices],IBBS$SLC12A5_2_methylation_rate_ave[indices],IBBS$SLC12A5_3_methylation_rate_ave[indices])

colnames(dr)<-c("age","methylslc_2","methylslc_3")

bestmodel_SVRM<-svm(age~methylslc_2+methylslc_3, data=dr,

cost=best.cost, gamma= best.gamma, epsilon=0.1, scale = FALSE)

newdata<-data.frame(methylslc_2=IBBS$SLC12A5_2_methylation_rate_ave[z],methylslc_3=IBBS$SLC12A5_3_methylation_rate_ave[z])

p<-predict(bestmodel_SVRM,newdata)*sd(AGE)+mean(AGE)

if (p<0){p=0}

predict_SVRM_loocv[z]<-p}

IBB_SVRM_loocv<-cbind(IBB,predict_SVRM_loocv)

MAE_SVRM_loocv<-mean(abs(IBB_SVRM_loocv$predict_SVRM_loocv-IBB$age))

MedianAE_SVRM_loocv<-median(abs(IBB_SVRM_loocv$predict_SVRM_loocv-IBB$age))

RMSE_SVRM_loocv<- sqrt(mean((IBB_SVRM_loocv$predict_SVRM_loocv-IBB$age)^2))

cat("MAE:", MAE_SVRM_loocv, "\nMed AE:", MedianAE_SVRM_loocv, "\nRMSE:", RMSE_SVRM_loocv, "\n")

MAE: 1.756718

Med AE: 1.438754

RMSE: 2.25164

Support vector regression (SLC12A5-2, -4)

set.seed(1)

tuneResult<-

tune(svm,age~SLC12A5_2_methylation_rate_ave+SLC12A5_4_methylation_rate_ave,data=IBBS,

ranges=list(cost=10^(seq(-2,4,0.1)),gamma=10^(seq(-5,1,0.1))),

tunecontrol = tune.control(sampling = "cross", cross = 10), scale = FALSE)

tunedModel <- tuneResult$best.model

tunedModel

Call:

best.tune(METHOD = svm, train.x = age ~ SLC12A5_2_methylation_rate_ave +

SLC12A5_4_methylation_rate_ave, data = IBBS, ranges = list(cost = 10^(seq(-2,

4, 0.1)), gamma = 10^(seq(-5, 1, 0.1))), tunecontrol = tune.control(sampling = "cross",

cross = 10), scale = FALSE)

Parameters:

SVM-Type: eps-regression

SVM-Kernel: radial

cost: 10

gamma: 0.1258925

epsilon: 0.1

Number of Support Vectors: 77

best.cost <- tunedModel$cost

best.gamma <- tunedModel$gamma

cat("Cost: ", best.cost, "\nGamma: ", best.gamma, "\n")

Cost: 10

Gamma: 0.1258925

tune_results <- as.data.frame(tuneResult$performances)

tune_results$cost <- log10(tune_results$cost)

tune_results$gamma <- log10(tune_results$gamma)

ggplot(tune_results, aes(x = cost, y = gamma, fill = error)) +

geom_tile() +

geom_point(aes(x = log10(best.cost), y = log10(best.gamma)), color = "blue", size = 1, shape = 21, fill = "blue") +

scale_fill_gradient(low = "white", high = "red") +

labs(title = "SVM Parameter Tuning Results",

x = "Log10(Cost)",

y = "Log10(Gamma)",

fill = "Error") +

theme_minimal()


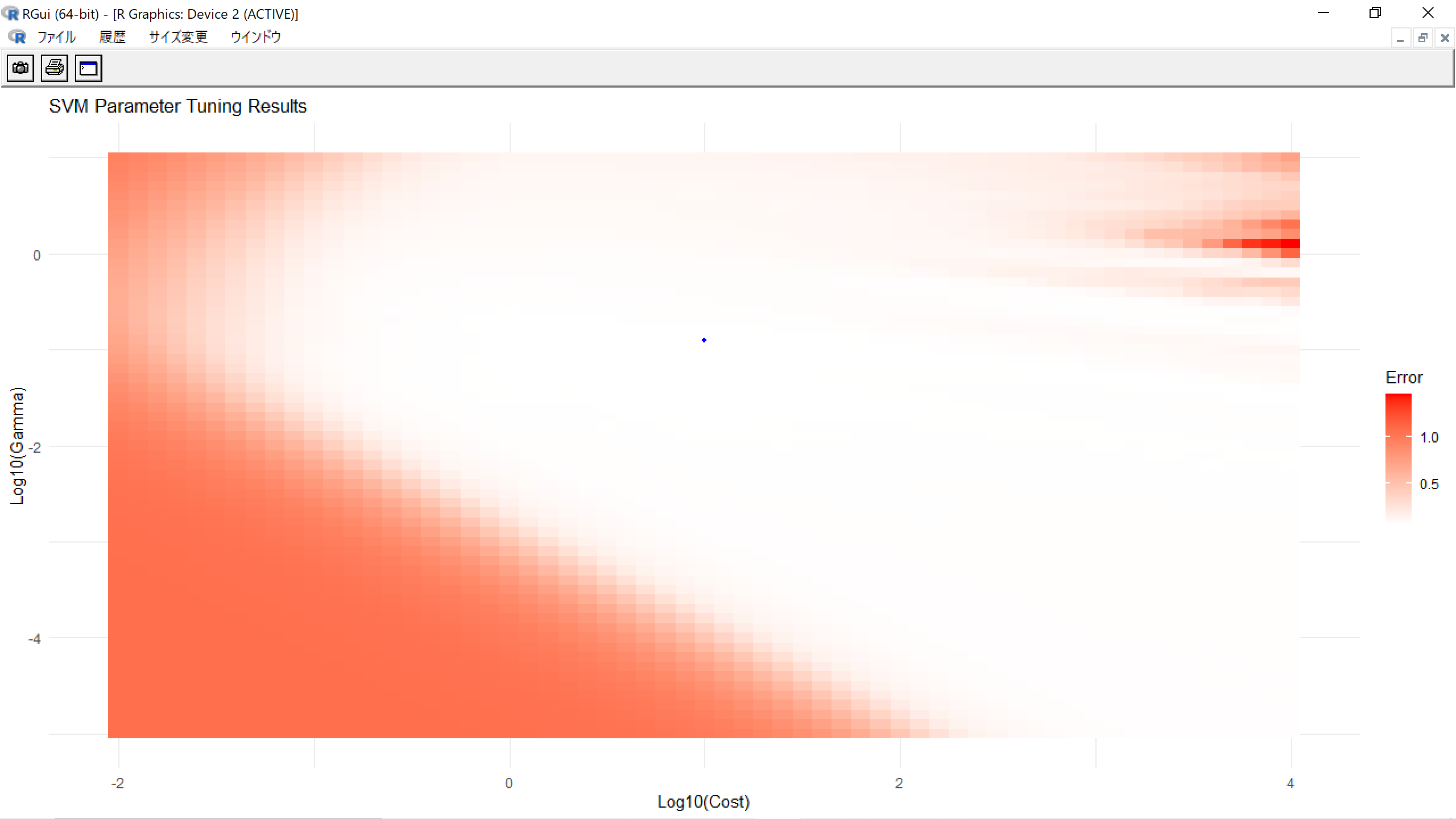


SVRM<-

svm(age~SLC12A5_2_methylation_rate_ave+SLC12A5_4_methylation_rate_ave,　data=IBBS,

cost=best.cost, gamma=best.gamma, epsilon=0.1, scale = FALSE)

#LOOCV

nSamples<-nrow(IBBS)

predict_SVRM_loocv<-numeric(nSamples)

for (z in 1:nSamples){

indices<-removeOne(nSamples,z)

dr<-data.frame(IBBS$age[indices],IBBS$SLC12A5_2_methylation_rate_ave[indices],IBBS$SLC12A5_4_methylation_rate_ave[indices])

colnames(dr)<-c("age","methylslc_2","methylslc_4")

bestmodel_SVRM<-svm(age~methylslc_2+methylslc_4, data=dr,

cost=best.cost, gamma= best.gamma, epsilon=0.1, scale = FALSE)

newdata<-data.frame(methylslc_2=IBBS$SLC12A5_2_methylation_rate_ave[z],methylslc_4=IBBS$SLC12A5_4_methylation_rate_ave[z])

p<-predict(bestmodel_SVRM,newdata)*sd(AGE)+mean(AGE)

if (p<0){p=0}

predict_SVRM_loocv[z]<-p}

IBB_SVRM_loocv<-cbind(IBB,predict_SVRM_loocv)

MAE_SVRM_loocv<-mean(abs(IBB_SVRM_loocv$predict_SVRM_loocv-IBB$age))

MedianAE_SVRM_loocv<-median(abs(IBB_SVRM_loocv$predict_SVRM_loocv-IBB$age))

RMSE_SVRM_loocv<- sqrt(mean((IBB_SVRM_loocv$predict_SVRM_loocv-IBB$age)^2))

cat("MAE:", MAE_SVRM_loocv, "\nMed AE:", MedianAE_SVRM_loocv, "\nRMSE:", RMSE_SVRM_loocv, "\n")

MAE: 1.478064

Med AE: 1.18985

RMSE: 1.915936

Support vector regression (SLC12A5-3, -4)

set.seed(1)

tuneResult<-

tune(svm,age~SLC12A5_3_methylation_rate_ave+SLC12A5_4_methylation_rate_ave,data=IBBS,

ranges=list(cost=10^(seq(-2,4,0.1)),gamma=10^(seq(-5,1,0.1))),

tunecontrol = tune.control(sampling = "cross", cross = 10), scale = FALSE)

tunedModel <- tuneResult$best.model

tunedModel

Call:

best.tune(METHOD = svm, train.x = age ~ SLC12A5_3_methylation_rate_ave +

SLC12A5_4_methylation_rate_ave, data = IBBS, ranges = list(cost = 10^(seq(-2,

4, 0.1)), gamma = 10^(seq(-5, 1, 0.1))), tunecontrol = tune.control(sampling = "cross",

cross = 10), scale = FALSE)

Parameters:

SVM-Type: eps-regression

SVM-Kernel: radial

cost: 10000

gamma: 0.01584893

epsilon: 0.1

Number of Support Vectors: 85

best.cost <- tunedModel$cost

best.gamma <- tunedModel$gamma

cat("Cost: ", best.cost, "\nGamma: ", best.gamma, "\n")

Cost: 10000

Gamma: 0.01584893

tune_results <- as.data.frame(tuneResult$performances)

tune_results$cost <- log10(tune_results$cost)

tune_results$gamma <- log10(tune_results$gamma)

ggplot(tune_results, aes(x = cost, y = gamma, fill = error)) +

geom_tile() +

geom_point(aes(x = log10(best.cost), y = log10(best.gamma)), color = "blue", size = 1, shape = 21, fill = "blue") +

scale_fill_gradient(low = "white", high = "red") +

labs(title = "SVM Parameter Tuning Results",

x = "Log10(Cost)",

y = "Log10(Gamma)",

fill = "Error") +

theme_minimal()


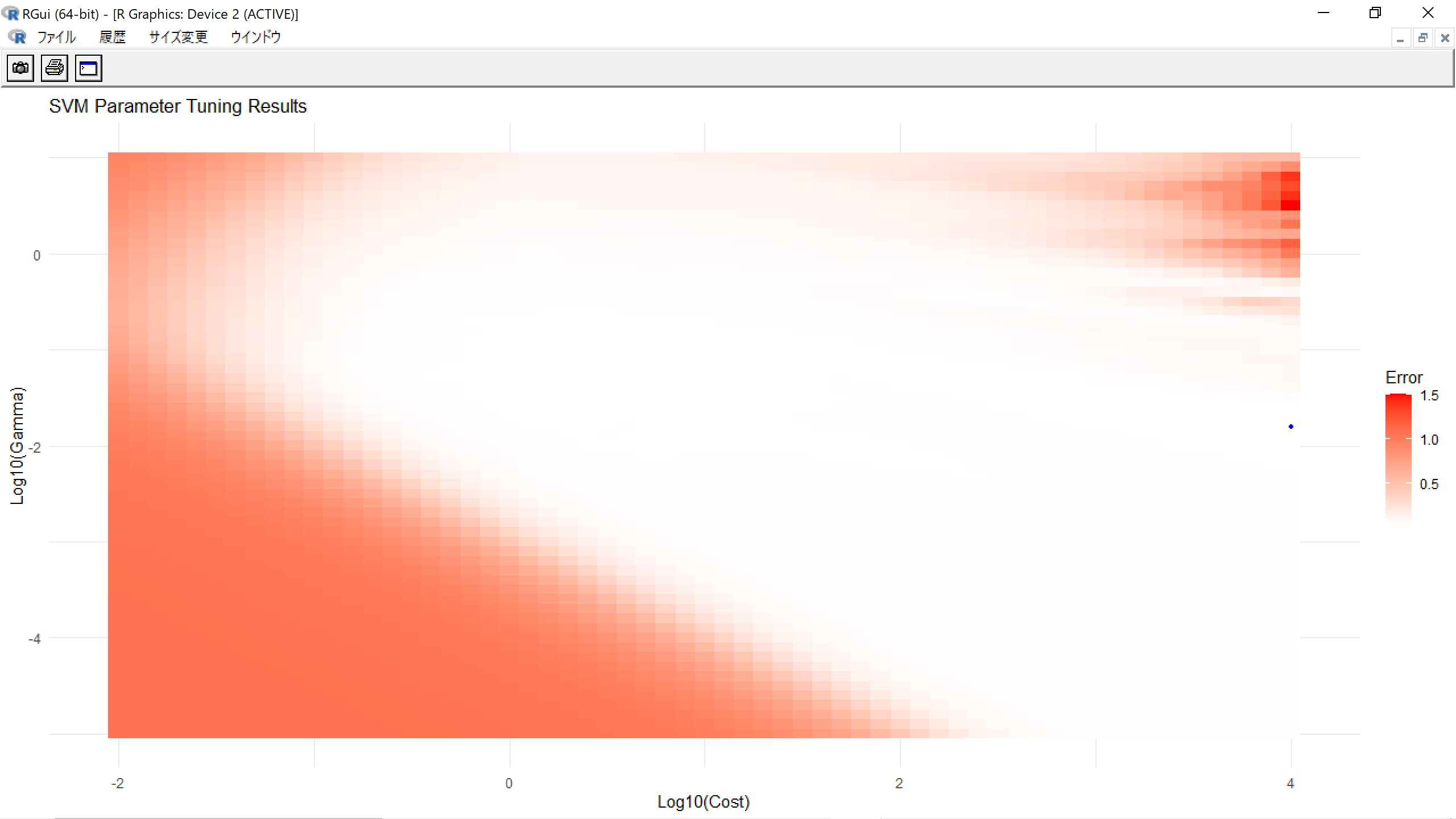


SVRM<-

svm(age~SLC12A5_3_methylation_rate_ave+SLC12A5_4_methylation_rate_ave,　data=IBBS,

cost=best.cost, gamma=best.gamma, epsilon=0.1, scale = FALSE)

#LOOCV

nSamples<-nrow(IBBS)

predict_SVRM_loocv<-numeric(nSamples)

for (z in 1:nSamples){

indices<-removeOne(nSamples,z)

dr<-data.frame(IBBS$age[indices],IBBS$SLC12A5_3_methylation_rate_ave[indices],IBBS$SLC12A5_4_methylation_rate_ave[indices])

colnames(dr)<-c("age","methylslc_3","methylslc_4")

bestmodel_SVRM<-svm(age~ methylslc_3+methylslc_4, data=dr,

cost=best.cost, gamma= best.gamma, epsilon=0.1, scale = FALSE)

newdata<-data.frame(methylslc_3=IBBS$SLC12A5_3_methylation_rate_ave[z],methylslc_4=IBBS$SLC12A5_4_methylation_rate_ave[z])

p<-predict(bestmodel_SVRM,newdata)*sd(AGE)+mean(AGE)

if (p<0){p=0}

predict_SVRM_loocv[z]<-p}

IBB_SVRM_loocv<-cbind(IBB,predict_SVRM_loocv)

MAE_SVRM_loocv<-mean(abs(IBB_SVRM_loocv$predict_SVRM_loocv-IBB$age))

MedianAE_SVRM_loocv<-median(abs(IBB_SVRM_loocv$predict_SVRM_loocv-IBB$age))

RMSE_SVRM_loocv<- sqrt(mean((IBB_SVRM_loocv$predict_SVRM_loocv-IBB$age)^2))

cat("MAE:", MAE_SVRM_loocv, "\nMed AE:", MedianAE_SVRM_loocv, "\nRMSE:", RMSE_SVRM_loocv, "\n")

MAE: 1.515522

Med AE: 1.193768

RMSE: 1.918024

Influences of interaction among age, sex, and growth environment

Single regression (SLC12A5-4)

SRM_SLC12A5_4<-lm(formula=YS~slc4S,data=IBBS)

predicted_age_SRM_SLC12A5_4_s <- predict(SRM_SLC12A5_4)

#Δage

deltaage_SRM_SLC12A5_4<- predicted_age_SRM_SLC12A5_4_s-IBBS$age

modeldelta_SRM_SLC12A5_4<-lm(formula=deltaage_SRM_SLC12A5_4~IBBS$species*IBBS$age*IBBS$sex*IBBS$environment)

options(na.action="na.fail")

modellist_SRM_SLC12A5_4<-dredge(modeldelta_SRM_SLC12A5_4,rank="AIC")

bestmodel_SRM_SLC12A5_4<-get.models(dredge(modeldelta_SRM_SLC12A5_4,rank="AIC"),subset=1)

bestmodel_SRM_SLC12A5_4

$`74`

Call:

lm(formula = deltaage_SRM_SLC12A5_4 ~ IBBS$age + IBBS$species +

IBBS$age:IBBS$species + 1)

Coefficients:

(Intercept) IBBS$age IBBS$speciesBB

-0.02024 -0.02749 -0.01612

IBBS$speciesPB IBBS$speciesSB IBBS$age:IBBS$speciesBB

0.15765 0.38184 -0.00389

IBBS$age:IBBS$speciesPB IBBS$age:IBBS$speciesSB

-0.19940 -0.18248

attr(,"rank")

function (x)

do.call("rank", list(x))

<environment: 0x0000025bb347eef8>

attr(,"call")

AIC(x)

attr(,"class")

[1] "function" "rankFunction"

attr(,"beta")

[1] "none"

summary(lm(formula = deltaage_SRM_SLC12A5_4 ~ IBBS$age + IBBS$species +

IBBS$age:IBBS$species + 1))

Call:

lm(formula = deltaage_SRM_SLC12A5_4 ~ IBBS$age + IBBS$species +

IBBS$age:IBBS$species + 1)

Residuals:

Min 1Q Median 3Q Max

-0.42082 -0.13800 -0.01428 0.14542 0.76814

Coefficients:

Estimate Std. Error t value Pr(>|t|)

(Intercept) -0.02024 0.03105 -0.652 0.515743

IBBS$age -0.02749 0.03871 -0.710 0.479013

IBBS$speciesBB -0.01612 0.04290 -0.376 0.707798

IBBS$speciesPB 0.15765 0.05888 2.677 0.008440 **

IBBS$speciesSB 0.38184 0.10076 3.790 0.000236 ***

IBBS$age:IBBS$speciesBB -0.00389 0.04700 -0.083 0.934175

IBBS$age:IBBS$speciesPB -0.19939 0.05787 -3.446 0.000782 ***

IBBS$age:IBBS$speciesSB -0.18248 0.11190 -1.631 0.105523

---

Signif. codes: 0 ‘***’ 0.001 ‘**’ 0.01 ‘*’ 0.05 ‘.’ 0.1 ‘ ’ 1

Residual standard error: 0.2072 on 122 degrees of freedom

Multiple R-squared: 0.275, Adjusted R-squared: 0.2334

F-statistic: 6.609 on 7 and 122 DF, p-value: 1.257e-06

#|Δage|

absdeltaage_SRM_SLC12A5_4<- abs(predicted_age_SRM_SLC12A5_4_s-IBBS$age)

modelabsdelta_SRM_SLC12A5_4<-

lm(formula =absdeltaage_SRM_SLC12A5_4~IBBS$species*IBBS$age*IBBS$sex*IBBS$environment)

options(na.action = "na.fail")

modellist_absSRM_SLC12A5_4<-dredge(modelabsdelta_SRM_SLC12A5_4,rank="AIC")

bestmodel_absSRM_SLC12A5_4<- get.models(dredge(modelabsdelta_SRM_SLC12A5_4,rank="AIC"),subset=1)

bestmodel_absSRM_SLC12A5_4

$`590`

Call:

lm(formula = absdeltaage_SRM_SLC12A5_4 ~ IBBS$age + IBBS$sex +

IBBS$species + IBBS$age:IBBS$species + IBBS$sex:IBBS$species +

1)

Coefficients:

(Intercept) IBBS$age IBBS$sexM

0.153844 0.002465 0.003628

IBBS$speciesBB IBBS$speciesPB IBBS$speciesSB

0.043853 0.161963 0.156891

IBBS$age:IBBS$speciesBB IBBS$age:IBBS$speciesPB IBBS$age:IBBS$speciesSB

0.032716 0.013042 -0.232063

IBBS$sexM:IBBS$speciesBB IBBS$sexM:IBBS$speciesPB IBBS$sexM:IBBS$speciesSB

-0.074119 -0.171725 0.121209

attr(,"rank")

function (x)

do.call("rank", list(x))

<environment: 0x0000025bb3dba3d8>

attr(,"call")

AIC(x)

attr(,"class")

[1] "function" "rankFunction"

attr(,"beta")

[1] "none"

summary(lm(formula = absdeltaage_SRM_SLC12A5_4 ~ IBBS$age + IBBS$sex +

IBBS$species + IBBS$age:IBBS$species + IBBS$sex:IBBS$species +

1))

Call:

lm(formula = absdeltaage_SRM_SLC12A5_4 ~ IBBS$age + IBBS$sex +

IBBS$species + IBBS$age:IBBS$species + IBBS$sex:IBBS$species +

1)

Residuals:

Min 1Q Median 3Q Max

-0.25437 -0.09709 -0.00685 0.07135 0.38811

Coefficients:

Estimate Std. Error t value Pr(>|t|)

(Intercept) 0.153844 0.024415 6.301 5.28e-09 ***

IBBS$age 0.002465 0.025571 0.096 0.923352

IBBS$sexM 0.003628 0.038427 0.094 0.924932

IBBS$speciesBB 0.043853 0.034486 1.272 0.206007

IBBS$speciesPB 0.161963 0.044540 3.636 0.000411 ***

IBBS$speciesSB 0.156891 0.076585 2.049 0.042719 *

IBBS$age:IBBS$speciesBB 0.032716 0.031304 1.045 0.298101

IBBS$age:IBBS$speciesPB 0.013042 0.037616 0.347 0.729418

IBBS$age:IBBS$speciesSB -0.232063 0.073295 -3.166 0.001966 **

IBBS$sexM:IBBS$speciesBB -0.074119 0.056636 -1.309 0.193181

IBBS$sexM:IBBS$speciesPB -0.171725 0.072537 -2.367 0.019540 *

IBBS$sexM:IBBS$speciesSB 0.121209 0.103291 1.173 0.242971

---

Signif. codes: 0 ‘***’ 0.001 ‘**’ 0.01 ‘*’ 0.05 ‘.’ 0.1 ‘ ’ 1

Residual standard error: 0.1323 on 118 degrees of freedom

Multiple R-squared: 0.2612, Adjusted R-squared: 0.1924

F-statistic: 3.794 on 11 and 118 DF, p-value: 0.0001132

Principal component regression (PC1)

IBBS$SLC12A5_PC1 <- pca_slc$x[,1]

PCRM_SLC <- lm(formula = age ~ SLC12A5_PC1, data = IBBS)

predicted_age_PCRM_SLC_s <- predict(PCRM_SLC)

#Δage

deltaage_PCRM_SLC<- predicted_age_PCRM_SLC_s-IBBS$age

modeldelta_PCRM_SLC<-lm(formula=deltaage_PCRM_SLC~IBBS$age*IBBS$sex*IBBS$environment)

options(na.action="na.fail")

modellist_PCRM_SLC<-dredge(modeldelta_PCRM_SLC,rank="AIC")

bestmodel_PCRM_SLC<-get.models(dredge(modeldelta_PCRM_SLC,rank="AIC"),subset=1)

bestmodel_PCRM_SLC

$`8`

Call:

lm(formula = deltaage_PCRM_SLC ~ IBBS$age + IBBS$environment +

IBBS$sex + 1)

Coefficients:

(Intercept) IBBS$age IBBS$environmentWild

0.06702 -0.08047 -0.09655

IBBS$sexM

-0.07429

attr(,"rank")

function (x)

do.call("rank", list(x))

<environment: 0x000001af94edf2e0>

attr(,"call")

AIC(x)

attr(,"class")

[1] "function" "rankFunction"

attr(,"beta")

[1] "none"

summary(lm(formula = deltaage_PCRM_SLC ~ IBBS$age + IBBS$environment +

IBBS$sex + 1))

Call:

lm(formula = deltaage_PCRM_SLC ~ IBBS$age + IBBS$environment +

IBBS$sex + 1)

Residuals:

Min 1Q Median 3Q Max

-0.47959 -0.15272 -0.02631 0.13285 0.80019

Coefficients:

Estimate Std. Error t value Pr(>|t|)

(Intercept) 0.06702 0.03289 2.038 0.04365 *

IBBS$age -0.08047 0.02217 -3.630 0.00041 ***

IBBS$environmentWild -0.09655 0.04452 -2.169 0.03197 *

IBBS$sexM -0.07429 0.04222 -1.760 0.08088 .

---

Signif. codes: 0 ‘***’ 0.001 ‘**’ 0.01 ‘*’ 0.05 ‘.’ 0.1 ‘ ’ 1

Residual standard error: 0.2261 on 126 degrees of freedom

Multiple R-squared: 0.1026, Adjusted R-squared: 0.08128

F-statistic: 4.804 on 3 and 126 DF, p-value: 0.003343

#|Δage|

absdeltaage_PCRM_SLC<- abs(predicted_age_PCRM_SLC_s-IBBS$age)

modelabsdelta_PCRM_SLC<-lm(formula=absdeltaage_PCRM_SLC~IBBS$age*IBBS$sex*IBBS$environment)

options(na.action = "na.fail")

modellist_absPCRM_SLC<-dredge(modelabsdelta_PCRM_SLC,rank="AIC")

bestmodel_absPCRM_SLC<- get.models(dredge(modelabsdelta_PCRM_SLC,rank="AIC"),subset=1)

bestmodel_absPCRM_SLC

$`22`

Call:

lm(formula = absdeltaage_PCRM_SLC ~ IBBS$age + IBBS$sex + IBBS$age:IBBS$sex +

1)

Coefficients:

(Intercept) IBBS$age IBBS$sexM IBBS$age:IBBS$sexM

0.19470 0.04875 -0.03281 -0.04873

attr(,"rank")

function (x)

do.call("rank", list(x))

<environment: 0x000001af974e5e48>

attr(,"call")

AIC(x)

attr(,"class")

[1] "function" "rankFunction"

attr(,"beta")

[1] "none"

summary(lm(formula = absdeltaage_PCRM_SLC ~ IBBS$age + IBBS$sex + IBBS$age:IBBS$sex +

1))

Call:

lm(formula = absdeltaage_PCRM_SLC ~ IBBS$age + IBBS$sex + IBBS$age:IBBS$sex +

1)

Residuals:

Min 1Q Median 3Q Max

-0.27765 -0.10510 -0.02190 0.09243 0.40838

Coefficients:

Estimate Std. Error t value Pr(>|t|)

(Intercept) 0.19470 0.01565 12.441 < 2e-16 ***

IBBS$age 0.04875 0.01435 3.396 0.000914 ***

IBBS$sexM -0.03281 0.02555 -1.284 0.201467

IBBS$age:IBBS$sexM -0.04873 0.02827 -1.724 0.087193 .

---

Signif. codes: 0 ‘***’ 0.001 ‘**’ 0.01 ‘*’ 0.05 ‘.’ 0.1 ‘ ’ 1

Residual standard error: 0.1376 on 126 degrees of freedom

Multiple R-squared: 0.1015, Adjusted R-squared: 0.08015

F-statistic: 4.747 on 3 and 126 DF, p-value: 0.003596

Elastic net regression (SLC12A5-1, -2, -3, -4)

X <- cbind(slc1S,slc2S,slc3S,slc4S)

ENM4 <- glmnet(x = cbind(IBBS$SLC12A5_1_methylation_rate_ave,IBBS$SLC12A5_2_methylation_rate_ave,IBBS$SLC12A5_3_methylation_rate_ave,IBBS$SLC12A5_4_methylation_rate_ave),

y = IBBS$age, family = "gaussian", lambda = 0.00842406, alpha = 0.02, standardize = FALSE)

predicted_age_s <- c(predict(ENM4, newx = X, s= 0.00842406))

#Δage

deltaage_ENM4<- predicted_age_s-IBBS$age

modeldelta_ENM4<-

lm(formula =deltaage_ENM4~IBBS$species*IBBS$age*IBBS$sex*IBBS$environment)

options(na.action = "na.fail")

modellist_ENM3<-dredge(modeldelta_ENM4,rank="AIC")

bestmodel_ENM4<- get.models(dredge(modeldelta_ENM4,rank="AIC"),subset=1)

bestmodel_ENM4

$`92`

Call:

lm(formula = deltaage_ENM4 ~ IBBS$age + IBBS$environment + IBBS$species +

IBBS$age:IBBS$environment + IBBS$age:IBBS$species + 1)

Coefficients:

(Intercept) IBBS$age

-0.02814 0.03934

IBBS$environmentWild IBBS$speciesBB

-0.03302 0.03063

IBBS$speciesPB IBBS$speciesSB

0.11924 0.39202

IBBS$age:IBBS$environmentWild IBBS$age:IBBS$speciesBB

-0.09694 -0.05645

IBBS$age:IBBS$speciesPB IBBS$age:IBBS$speciesSB

-0.25111 -0.29887

attr(,"rank")

function (x)

do.call("rank", list(x))

<environment: 0x0000024dafd6ae68>

attr(,"call")

AIC(x)

attr(,"class")

[1] "function" "rankFunction"

attr(,"beta")

[1] "none"

summary(lm(formula = deltaage_ENM4 ~ IBBS$age + IBBS$environment + IBBS$species +

IBBS$age:IBBS$environment + IBBS$age:IBBS$species + 1))

Call:

lm(formula = deltaage_ENM4 ~ IBBS$age + IBBS$environment + IBBS$species +

IBBS$age:IBBS$environment + IBBS$age:IBBS$species + 1)

Residuals:

Min 1Q Median 3Q Max

-0.4130 -0.1365 0.0020 0.1039 0.7217

Coefficients:

Estimate Std. Error t value Pr(>|t|)

(Intercept) -0.02814 0.03929 -0.716 0.4753

IBBS$age 0.03934 0.04234 0.929 0.3547

IBBS$environmentWild -0.03302 0.04511 -0.732 0.4656

IBBS$speciesBB 0.03063 0.04176 0.734 0.4647

IBBS$speciesPB 0.11924 0.06043 1.973 0.0508 .

IBBS$speciesSB 0.39202 0.09634 4.069 8.48e-05 ***

IBBS$age:IBBS$environmentWild -0.09694 0.04948 -1.959 0.0524 .

IBBS$age:IBBS$speciesBB -0.05645 0.04493 -1.257 0.2114

IBBS$age:IBBS$speciesPB -0.25111 0.05789 -4.338 3.02e-05 ***

IBBS$age:IBBS$speciesSB -0.29887 0.10524 -2.840 0.0053 **

---

Signif. codes: 0 ‘***’ 0.001 ‘**’ 0.01 ‘*’ 0.05 ‘.’ 0.1 ‘ ’ 1

Residual standard error: 0.1902 on 120 degrees of freedom

Multiple R-squared: 0.3076, Adjusted R-squared: 0.2557

F-statistic: 5.924 on 9 and 120 DF, p-value: 7.93e-07

#|Δage|

absdeltaage_ENM4<- abs(predicted_age_s-IBBS$age)

modeldelta_absENM4<-

lm(formula =absdeltaage_ENM4~IBBS$species*IBBS$age*IBBS$sex*IBBS$environment)

options(na.action = "na.fail")

modellist_absENM4<-dredge(modeldelta_absENM4,rank="AIC")

bestmodel_absENM4<- get.models(dredge(modeldelta_absENM4,rank="AIC"),subset=1)

bestmodel_absENM4

$`464`

Call:

lm(formula = absdeltaage_ENM4 ~ IBBS$age + IBBS$environment +

IBBS$sex + IBBS$species + IBBS$age:IBBS$species + IBBS$environment:IBBS$sex +

IBBS$environment:IBBS$species + 1)

Coefficients:

(Intercept) IBBS$age

0.15476 0.01208

IBBS$environmentWild IBBS$sexM

-0.03095 -0.06791

IBBS$speciesBB IBBS$speciesPB

0.01397 0.09305

IBBS$speciesSB IBBS$age:IBBS$speciesBB

0.22938 0.03039

IBBS$age:IBBS$speciesPB IBBS$age:IBBS$speciesSB

0.03174 -0.20635

IBBS$environmentWild:IBBS$sexM IBBS$environmentWild:IBBS$speciesBB

0.12534 0.07712

IBBS$environmentWild:IBBS$speciesPB IBBS$environmentWild:IBBS$speciesSB

NA NA

attr(,"rank")

function (x)

do.call("rank", list(x))

<environment: 0x0000024db0c2b248>

attr(,"call")

AIC(x)

attr(,"class")

[1] "function" "rankFunction"

attr(,"beta")

[1] "none"

summary(lm(formula = absdeltaage_ENM4 ~ IBBS$age + IBBS$environment +

IBBS$sex + IBBS$species + IBBS$age:IBBS$species + IBBS$environment:IBBS$sex +

IBBS$environment:IBBS$species + 1))

Call:

lm(formula = absdeltaage_ENM4 ~ IBBS$age + IBBS$environment +

IBBS$sex + IBBS$species + IBBS$age:IBBS$species + IBBS$environment:IBBS$sex +

IBBS$environment:IBBS$species + 1)

Residuals:

Min 1Q Median 3Q Max

-0.24235 -0.08577 -0.01636 0.07319 0.42476

Coefficients: (2 not defined because of singularities)

Estimate Std. Error t value Pr(>|t|)

(Intercept) 0.15476 0.03247 4.767 5.4e-06 ***

IBBS$age 0.01208 0.02464 0.490 0.624831

IBBS$environmentWild -0.03095 0.04334 -0.714 0.476553

IBBS$sexM -0.06791 0.02982 -2.277 0.024578 *

IBBS$speciesBB 0.01397 0.03731 0.374 0.708739

IBBS$speciesPB 0.09305 0.04230 2.200 0.029757 *

IBBS$speciesSB 0.22938 0.06388 3.591 0.000481 ***

IBBS$age:IBBS$speciesBB 0.03039 0.02954 1.029 0.305616

IBBS$age:IBBS$speciesPB 0.03174 0.03535 0.898 0.371089

IBBS$age:IBBS$speciesSB -0.20635 0.06658 -3.099 0.002426 **

IBBS$environmentWild:IBBS$sexM 0.12534 0.04877 2.570 0.011416 *

IBBS$environmentWild:IBBS$speciesBB 0.07712 0.05689 1.356 0.177832

IBBS$environmentWild:IBBS$speciesPB NA NA NA NA

IBBS$environmentWild:IBBS$speciesSB NA NA NA NA

---

Signif. codes: 0 ‘***’ 0.001 ‘**’ 0.01 ‘*’ 0.05 ‘.’ 0.1 ‘ ’ 1

Residual standard error: 0.1214 on 118 degrees of freedom

Multiple R-squared: 0.296, Adjusted R-squared: 0.2304

F-statistic: 4.511 on 11 and 118 DF, p-value: 1.114e-05

Support vector regression (SLC12A5-1, -2, -4)

SVRM3<-

svm(age~SLC12A5_1_methylation_rate_ave+SLC12A5_2_methylation_rate_ave +SLC12A5_4_methylation_rate_ave,　data=IBBS,

cost=10^0.2,gamma=10^-1.5,epsilon=0.1, scale = FALSE)

predict_SVRM3_s <- predict(SVRM3)

#Δage

deltaage_SVRM3<- predict_SVRM3_s-IBBS$age

modeldelta_SVRM3<-

lm(formula =deltaage_SVRM3~IBBS$species*IBBS$age*IBBS$sex*IBBS$environment)

options(na.action = "na.fail")

modellist_SVRM3<-dredge(modeldelta_SVRM3,rank="AIC")

bestmodel_SVRM3<- get.models(dredge(modeldelta_SVRM3,rank="AIC"),subset=1)

bestmodel_SVRM3

$`348`

Call:

lm(formula = deltaage_SVRM3 ~ IBBS$age + IBBS$environment + IBBS$species +

IBBS$age:IBBS$environment + IBBS$age:IBBS$species + IBBS$environment:IBBS$species +

1)

Coefficients:

(Intercept) IBBS$age

0.004403 0.016749

IBBS$environmentWild IBBS$speciesBB

-0.141713 -0.046268

IBBS$speciesPB IBBS$speciesSB

0.074166 0.396967

IBBS$age:IBBS$environmentWild IBBS$age:IBBS$speciesBB

-0.146505 -0.038533

IBBS$age:IBBS$speciesPB IBBS$age:IBBS$speciesSB

-0.171689 -0.216494

IBBS$environmentWild:IBBS$speciesBB IBBS$environmentWild:IBBS$speciesPB

0.170254 NA

IBBS$environmentWild:IBBS$speciesSB

NA

attr(,"rank")

function (x)

do.call("rank", list(x))

<environment: 0x0000024dae385438>

attr(,"call")

AIC(x)

attr(,"class")

[1] "function" "rankFunction"

attr(,"beta")

[1] "none"

summary(lm(formula = deltaage_SVRM3 ~ IBBS$age + IBBS$environment + IBBS$species +

IBBS$age:IBBS$environment + IBBS$age:IBBS$species + IBBS$environment:IBBS$species +

1))

Call:

lm(formula = deltaage_SVRM3 ~ IBBS$age + IBBS$environment + IBBS$species +

IBBS$age:IBBS$environment + IBBS$age:IBBS$species + IBBS$environment:IBBS$species +

1)

Residuals:

Min 1Q Median 3Q Max

-0.33405 -0.09561 -0.01532 0.09993 0.41934

Coefficients: (2 not defined because of singularities)

Estimate Std. Error t value Pr(>|t|)

(Intercept) 0.004403 0.040561 0.109 0.913732

IBBS$age 0.016749 0.037028 0.452 0.651844

IBBS$environmentWild -0.141713 0.052705 -2.689 0.008200 **

IBBS$speciesBB -0.046268 0.049323 -0.938 0.350111

IBBS$speciesPB 0.074166 0.056314 1.317 0.190365

IBBS$speciesSB 0.396967 0.085128 4.663 8.21e-06 ***

IBBS$age:IBBS$environmentWild -0.146505 0.042138 -3.477 0.000709 ***

IBBS$age:IBBS$speciesBB -0.038533 0.040125 -0.960 0.338841

IBBS$age:IBBS$speciesPB -0.171689 0.049993 -3.434 0.000819 ***

IBBS$age:IBBS$speciesSB -0.216494 0.089952 -2.407 0.017631 *

IBBS$environmentWild:IBBS$speciesBB 0.170254 0.073402 2.319 0.022076 *

IBBS$environmentWild:IBBS$speciesPB NA NA NA NA

IBBS$environmentWild:IBBS$speciesSB NA NA NA NA

---

Signif. codes: 0 ‘***’ 0.001 ‘**’ 0.01 ‘*’ 0.05 ‘.’ 0.1 ‘ ’ 1

Residual standard error: 0.1618 on 119 degrees of freedom

Multiple R-squared: 0.4123, Adjusted R-squared: 0.363

F-statistic: 8.35 on 10 and 119 DF, p-value: 3.587e-10

#|Δage|

absdeltaage_SVRM3<- abs(predict_SVRM3_s-IBBS$age)

modeldelta_absSVRM3<-

lm(formula =absdeltaage_SVRM3~IBBS$species*IBBS$age*IBBS$sex*IBBS$environment)

options(na.action = "na.fail")

modellist_absSVRM3<-dredge(modeldelta_absSVRM3,rank="AIC")

bestmodel_absSVRM3<- get.models(dredge(modeldelta_absSVRM3,rank="AIC"),subset=1)

bestmodel_absSVRM3

$`2528`

Call:

lm(formula = absdeltaage_SVRM3 ~ IBBS$age + IBBS$environment +

IBBS$sex + IBBS$species + IBBS$age:IBBS$environment + IBBS$age:IBBS$species +

IBBS$environment:IBBS$sex + IBBS$environment:IBBS$species +

IBBS$age:IBBS$environment:IBBS$species + 1)

Coefficients:

(Intercept)

0.135591

IBBS$age

-0.006746

IBBS$environmentWild

0.034019

IBBS$sexM

-0.039507

IBBS$speciesBB

-0.013054

IBBS$speciesPB

0.079457

IBBS$speciesSB

0.281875

IBBS$age:IBBS$environmentWild

0.110365

IBBS$age:IBBS$speciesBB

0.045447

IBBS$age:IBBS$speciesPB

0.042954

IBBS$age:IBBS$speciesSB

-0.186786

IBBS$environmentWild:IBBS$sexM

0.089349

IBBS$environmentWild:IBBS$speciesBB

0.036892

IBBS$environmentWild:IBBS$speciesPB

NA

IBBS$environmentWild:IBBS$speciesSB

NA

IBBS$age:IBBS$environmentWild:IBBS$speciesBB

-0.120189

IBBS$age:IBBS$environmentWild:IBBS$speciesPB

NA

IBBS$age:IBBS$environmentWild:IBBS$speciesSB

NA

attr(,"rank")

function (x)

do.call("rank", list(x))

<environment: 0x0000024db0ef8de8>

attr(,"call")

AIC(x)

attr(,"class")

[1] "function" "rankFunction"

attr(,"beta")

[1] "none"

summary(lm(formula = absdeltaage_SVRM3 ~ IBBS$age + IBBS$environment +

IBBS$sex + IBBS$species + IBBS$age:IBBS$environment + IBBS$age:IBBS$species +

IBBS$environment:IBBS$sex + IBBS$environment:IBBS$species +

IBBS$age:IBBS$environment:IBBS$species + 1))

Call:

lm(formula = absdeltaage_SVRM3 ~ IBBS$age + IBBS$environment +

IBBS$sex + IBBS$species + IBBS$age:IBBS$environment + IBBS$age:IBBS$species +

IBBS$environment:IBBS$sex + IBBS$environment:IBBS$species +

IBBS$age:IBBS$environment:IBBS$species + 1)

Residuals:

Min 1Q Median 3Q Max

-0.20857 -0.06085 -0.01870 0.05820 0.25998

Coefficients: (4 not defined because of singularities)

Estimate Std. Error t value

(Intercept) 0.135591 0.028716 4.722

IBBS$age -0.006746 0.028449 -0.237

IBBS$environmentWild 0.034019 0.038835 0.876

IBBS$sexM -0.039507 0.026700 -1.480

IBBS$speciesBB -0.013054 0.032939 -0.396

IBBS$speciesPB 0.079457 0.037270 2.132

IBBS$speciesSB 0.281875 0.056253 5.011

IBBS$age:IBBS$environmentWild 0.110365 0.043958 2.511

IBBS$age:IBBS$speciesBB 0.045447 0.032531 1.397

IBBS$age:IBBS$speciesPB 0.042954 0.036200 1.187

IBBS$age:IBBS$speciesSB -0.186786 0.061535 -3.035

IBBS$environmentWild:IBBS$sexM 0.089349 0.044454 2.010

IBBS$environmentWild:IBBS$speciesBB 0.036892 0.051939 0.710

IBBS$environmentWild:IBBS$speciesPB NA NA NA

IBBS$environmentWild:IBBS$speciesSB NA NA NA

IBBS$age:IBBS$environmentWild:IBBS$speciesBB -0.120189 0.056822 -2.115

IBBS$age:IBBS$environmentWild:IBBS$speciesPB NA NA NA

IBBS$age:IBBS$environmentWild:IBBS$speciesSB NA NA NA

Pr(>|t|)

(Intercept) 6.60e-06 ***

IBBS$age 0.81299

IBBS$environmentWild 0.38284

IBBS$sexM 0.14168

IBBS$speciesBB 0.69260

IBBS$speciesPB 0.03512 *

IBBS$speciesSB 1.96e-06 ***

IBBS$age:IBBS$environmentWild 0.01343 *

IBBS$age:IBBS$speciesBB 0.16507

IBBS$age:IBBS$speciesPB 0.23782

IBBS$age:IBBS$speciesSB 0.00297 **

IBBS$environmentWild:IBBS$sexM 0.04676 *

IBBS$environmentWild:IBBS$speciesBB 0.47895

IBBS$environmentWild:IBBS$speciesPB NA

IBBS$environmentWild:IBBS$speciesSB NA

IBBS$age:IBBS$environmentWild:IBBS$speciesBB 0.03655 *

IBBS$age:IBBS$environmentWild:IBBS$speciesPB NA

IBBS$age:IBBS$environmentWild:IBBS$speciesSB NA

---

Signif. codes: 0 ‘***’ 0.001 ‘**’ 0.01 ‘*’ 0.05 ‘.’ 0.1 ‘ ’ 1

Residual standard error: 0.1069 on 116 degrees of freedom

Multiple R-squared: 0.364, Adjusted R-squared: 0.2927

F-statistic: 5.106 on 13 and 116 DF, p-value: 4.272e-07

Support vector regression (SLC12A5-1, -3, -4)

SVRM3_2<-

svm(age~+SLC12A5_1_methylation_rate_ave+SLC12A5_3_methylation_rate_ave+SLC12A5_4_methylation_rate_ave,　data=IBBS,

cost=10^3.7,gamma=10^-1.9,epsilon=0.1, scale = FALSE)

predict_SVRM3_2_s <- predict(SVRM3_2)

#Δage

deltaage_SVRM3_2<- predict_SVRM3_2_s-IBBS$age

modeldelta_SVRM3_2<-

lm(formula =deltaage_SVRM3_2~IBBS$species*IBBS$age*IBBS$sex*IBBS$environment)

options(na.action = "na.fail")

modellist_SVRM3_2<-dredge(modeldelta_SVRM3_2,rank="AIC")

bestmodel_SVRM3_2<- get.models(dredge(modeldelta_SVRM3_2,rank="AIC"),subset=1)

bestmodel_SVRM3_2

$`348`

Call:

lm(formula = deltaage_SVRM3_2 ~ IBBS$age + IBBS$environment +

IBBS$species + IBBS$age:IBBS$environment + IBBS$age:IBBS$species +

IBBS$environment:IBBS$species + 1)

Coefficients:

(Intercept) IBBS$age

0.03215 0.03051

IBBS$environmentWild IBBS$speciesBB

-0.13671 -0.05355

IBBS$speciesPB IBBS$speciesSB

0.06217 0.26361

IBBS$age:IBBS$environmentWild IBBS$age:IBBS$speciesBB

-0.17393 -0.01602

IBBS$age:IBBS$speciesPB IBBS$age:IBBS$speciesSB

-0.17326 -0.15708

IBBS$environmentWild:IBBS$speciesBB IBBS$environmentWild:IBBS$speciesPB

0.12308 NA

IBBS$environmentWild:IBBS$speciesSB

NA

attr(,"rank")

function (x)

do.call("rank", list(x))

<environment: 0x0000024dabd36d18>

attr(,"call")

AIC(x)

attr(,"class")

[1] "function" "rankFunction"

attr(,"beta")

[1] "none"

summary(lm(formula = deltaage_SVRM3_2 ~ IBBS$age + IBBS$environment +

IBBS$species + IBBS$age:IBBS$environment + IBBS$age:IBBS$species +

IBBS$environment:IBBS$species + 1))

Call:

lm(formula = deltaage_SVRM3_2 ~ IBBS$age + IBBS$environment +

IBBS$species + IBBS$age:IBBS$environment + IBBS$age:IBBS$species +

IBBS$environment:IBBS$species + 1)

Residuals:

Min 1Q Median 3Q Max

-0.36016 -0.10067 -0.02156 0.09922 0.45466

Coefficients: (2 not defined because of singularities)

Estimate Std. Error t value Pr(>|t|)

(Intercept) 0.03215 0.03995 0.805 0.422544

IBBS$age 0.03051 0.03647 0.837 0.404503

IBBS$environmentWild -0.13671 0.05191 -2.634 0.009566 **

IBBS$speciesBB -0.05355 0.04858 -1.102 0.272531

IBBS$speciesPB 0.06217 0.05546 1.121 0.264554

IBBS$speciesSB 0.26361 0.08384 3.144 0.002103 **

IBBS$age:IBBS$environmentWild -0.17393 0.04150 -4.191 5.36e-05 ***

IBBS$age:IBBS$speciesBB -0.01602 0.03952 -0.405 0.686005

IBBS$age:IBBS$speciesPB -0.17326 0.04924 -3.519 0.000615 ***

IBBS$age:IBBS$speciesSB -0.15708 0.08859 -1.773 0.078762 .

IBBS$environmentWild:IBBS$speciesBB 0.12308 0.07229 1.703 0.091253 .

IBBS$environmentWild:IBBS$speciesPB NA NA NA NA

IBBS$environmentWild:IBBS$speciesSB NA NA NA NA

---

Signif. codes: 0 ‘***’ 0.001 ‘**’ 0.01 ‘*’ 0.05 ‘.’ 0.1 ‘ ’ 1

Residual standard error: 0.1594 on 119 degrees of freedom

Multiple R-squared: 0.3367, Adjusted R-squared: 0.281

F-statistic: 6.04 on 10 and 119 DF, p-value: 2.23e-07

#|Δage|

absdeltaage_SVRM3_2<- abs(predict_SVRM3_2_s-IBBS$age)

modeldelta_absSVRM3_2<-

lm(formula =absdeltaage_SVRM3_2~IBBS$species*IBBS$age*IBBS$sex*IBBS$environment)

options(na.action = "na.fail")

modellist_absSVRM3_2<-dredge(modeldelta_absSVRM3_2,rank="AIC")

bestmodel_absSVRM3_2<- get.models(dredge(modeldelta_absSVRM3_2,rank="AIC"),subset=1)

bestmodel_absSVRM3_2

$`80`

Call:

lm(formula = absdeltaage_SVRM3_2 ~ IBBS$age + IBBS$environment +

IBBS$sex + IBBS$species + IBBS$age:IBBS$species + 1)

Coefficients:

(Intercept) IBBS$age IBBS$environmentWild

0.105520 0.026488 0.052389

IBBS$sexM IBBS$speciesBB IBBS$speciesPB

-0.029754 0.021434 0.121199

IBBS$speciesSB IBBS$age:IBBS$speciesBB IBBS$age:IBBS$speciesPB

0.202362 -0.013360 -0.008683

IBBS$age:IBBS$speciesSB

-0.148385

attr(,"rank")

function (x)

do.call("rank", list(x))

<environment: 0x0000024db47a13a8>

attr(,"call")

AIC(x)

attr(,"class")

[1] "function" "rankFunction"

attr(,"beta")

[1] "none"

summary(lm(formula = absdeltaage_SVRM3_2 ~ IBBS$age + IBBS$environment +

IBBS$sex + IBBS$species + IBBS$age:IBBS$species + 1))

Call:

lm(formula = absdeltaage_SVRM3_2 ~ IBBS$age + IBBS$environment +

IBBS$sex + IBBS$species + IBBS$age:IBBS$species + 1)

Residuals:

Min 1Q Median 3Q Max

-0.19251 -0.06266 -0.01726 0.05409 0.34479

Coefficients:

Estimate Std. Error t value Pr(>|t|)

(Intercept) 0.105520 0.024268 4.348 2.9e-05 ***

IBBS$age 0.026488 0.020661 1.282 0.202307

IBBS$environmentWild 0.052389 0.024357 2.151 0.033491 *

IBBS$sexM -0.029754 0.020138 -1.477 0.142164

IBBS$speciesBB 0.021434 0.022947 0.934 0.352164

IBBS$speciesPB 0.121199 0.033482 3.620 0.000433 ***

IBBS$speciesSB 0.202362 0.052888 3.826 0.000208 ***

IBBS$age:IBBS$speciesBB -0.013360 0.023800 -0.561 0.575601

IBBS$age:IBBS$speciesPB -0.008683 0.030068 -0.289 0.773247

IBBS$age:IBBS$speciesSB -0.148385 0.057058 -2.601 0.010476 *

---

Signif. codes: 0 ‘***’ 0.001 ‘**’ 0.01 ‘*’ 0.05 ‘.’ 0.1 ‘ ’ 1

Residual standard error: 0.1042 on 120 degrees of freedom

Multiple R-squared: 0.2369, Adjusted R-squared: 0.1797

F-statistic: 4.14 on 9 and 120 DF, p-value: 0.0001153

How to apply to the models

MD <- read.csv("measurement_data.csv")

MD[,2] <- (MD[,2] - 42.572) / 14.209

MD[,3] <- (MD[,3] - 38.703) / 15.331

MD[,4] <- (MD[,4] - 28.888) / 12.219

MD[,5] <- (MD[,5] - 36.016) / 14.994

write.csv(MD, "measurement_data_standardized.csv", row.names = FALSE)

MDS <- read.csv("measurement_data_standardized.csv")

IBBS <- read.csv("integrate_bear_blood_standardized.csv")

SD <- 9.0109

MEAN <- 12.433

The file “measurement_data.csv” is a template file, available in Dryad (DOI: 10.5061/dryad.b5mkkwhqt). Write the sample IDs and measured methylation levels on it.

The “measurement_data_standardized.csv” file contains values of standardized methylation levels of “measurement_data.csv”.

The standardized values are calculated by the following equation.

“standardized value” = (“original value” − “mean of training data”) ÷ “standard deviation of training data”)

Single regression (SLC12A5-4)

SRM <-lm(formula=age~SLC12A5_4_methylation_rate_ave,data=IBBS)

predicted_age_SRM <- predict(SRM,MDS)*SD+MEAN

predicted_age_SRM[predicted_age_SRM < 0] <- 0

data.frame(Sample_ID = MDS$Sample_ID, Predicted_Age = predicted_age_SRM)

Principal component regression (PC1)

pca_slc <- prcomp(IBBS[, c("SLC12A5_1_methylation_rate_ave",

"SLC12A5_2_methylation_rate_ave",

"SLC12A5_3_methylation_rate_ave",

"SLC12A5_4_methylation_rate_ave")],

scale = FALSE)

IBBS$SLC12A5_PC1 <- pca_slc$x[,1]

PCRM <- lm(formula = age ~ SLC12A5_PC1, data = IBBS)

MDS$SLC12A5_PC1 <- as.numeric(as.matrix(MDS[,c("SLC12A5_1_methylation_rate_ave", "SLC12A5_2_methylation_rate_ave", "SLC12A5_3_methylation_rate_ave", "SLC12A5_4_methylation_rate_ave")]) %*% pca_slc$rotation[,1])

predicted_age_PCRM <- predict(PCRM,MDS)*SD+MEAN

predicted_age_PCRM[predicted_age_PCRM < 0] <- 0

data.frame(Sample_ID = MDS$Sample_ID, Predicted_Age = predicted_age_PCRM)

Elastic net regression (SLC12A5-1, -2, -3, -4)

library(glmnet)

ENM <- glmnet(x = cbind(IBBS$SLC12A5_1_methylation_rate_ave,IBBS$SLC12A5_2_methylation_rate_ave,IBBS$SLC12A5_3_methylation_rate_ave,IBBS$SLC12A5_4_methylation_rate_ave),

y = IBBS$age, family = "gaussian", lambda = 0.00842406, alpha = 0.02, standardize = FALSE)

MDS_ENM <- cbind(MDS$SLC12A5_1_methylation_rate_ave, MDS$SLC12A5_2_methylation_rate_ave, MDS$SLC12A5_3_methylation_rate_ave, MDS$SLC12A5_4_methylation_rate_ave)

predicted_age_ENM <- c(predict(ENM,MDS_ENM,s=0.00842406)*SD+MEAN)

predicted_age_ENM[predicted_age_ENM < 0] <- 0

data.frame(Sample_ID = MDS$Sample_ID, Predicted_Age = predicted_age_ENM)

Support vector regression (SLC12A5-1, -3, -4)

library(e1071)

SVRM<-

svm(age~SLC12A5_1_methylation_rate_ave+SLC12A5_3_methylation_rate_ave+SLC12A5_4_methylation_rate_ave,data=IBBS, cost=10^3.7, gamma=10^-1.9, epsilon=0.1, scale = FALSE)

predicted_age_SVRM<-predict(SVRM,MDS)*SD+MEAN

predicted_age_SVRM[predicted_age_SVRM < 0] <- 0

data.frame(Sample_ID = MDS$Sample_ID, Predicted_Age = predicted_age_SVRM)

Output to a csv file

sample_ID <- MDS[, 1, drop = FALSE]

predicted_age<-cbind(sample_ID,predicted_age_SRM,predicted_age_PCRM,predicted_age_ENM,predicted_age_SVRM)

write.csv(predicted_age, "predicted_age_result.csv", row.names = FALSE)

At this point, two files, “measurement_data_standardized.csv” and “predicted_age_result.csv”, should have been generated. Leaving them as they are may cause errors in subsequent runs, so please either rename the files or move them to a different folder.
