# Supplementary material for: Epigenetic Clock in Bears: A Simple Cost‐Effective Blood DNA Methylation‐Based Age Estimation Method Applicable to Multiple Bear Species
Source: Ecol Evol. 2025 May 6;15(5):e71424. doi: 10.1002/ece3.71424 (PMC12055220; doi:10.1002/ece3.71424)
Supplement: Supplementary file 3 — Appendix S3. [file ECE3-15-e71424-s002.pdf]

Supplemental Information for:

Title of Manuscript: Epigenetic clock in bears: A simple cost-effective blood DNA methylation-based age estimation method applicable to multiple bear species

Michito Shimozuru, Shiori Nakamura, Jumpei Yamazaki, Yojiro Yanagawa, Hiroo Tamatani, Misako Kuroe, Koji Yamazaki, Shinsuke Koike, Yusuke Goto, Tomoko Naganuma, Kahoko Tochigi, Akino Inagaki, Naoki Takekoshi, Seungyun Baek, Nobutaka Sato, Yusuke Honda, Toshio Tsubota, Hideyuki Ito

Table of Contents:

**Supplementary Table S_M1**	Page 2
**Supplementary Table S_R1**	Page 5
**Supplementary Table S_R2**	Page 6
**Supplementary Table S_R3**	Page 7
**Supplementary Table S_R4**	Page 8
**Supplementary Figure S_M1**	Page 9
**Supplementary Figure S_M2**	Page 10
**Supplementary Figure S_R1**	Page 11

Supplementary Table S_M1. List of samples used in this study.

Bear ID	Species	Captive/ Wild	Area (wild)	Sex	Date of birth	Date of 1st capture	Age at 1st capture	Date of sampling	Age (Years)	Model construction*
#1	ABB_JBB	C	–	F	1994/2/2	–	–	2012/6/15	18.4	included
#5	ABB_JBB	C	–	F	1994/1/28	–	–	2017/7/14	23.5	included
#6	ABB_JBB	C	–	F	1994/2/7	–	–	2010/11/4	16.7	included
#7	ABB_JBB	C	–	F	2012/2/1	–	–	2021/3/5	9.1	included
#8	ABB_JBB	C	–	F	2012/2/1	–	–	2017/2/23	5.1	included
#13	ABB_JBB	C	–	M	2013/1/27	–	–	2020/11/28	7.8	included
#17	ABB_JBB	C	–	M	2013/2/6	–	–	2017/9/11	4.6	included
#18	ABB_JBB	C	–	M	2015/1/31	–	–	2017/9/9	2.6	included
#184	ABB_JBB	C	–	M	2008/2/1	–	–	2021/9/6	13.6	included
#19	ABB_JBB	C	–	F	2015/1/31	–	–	2017/9/9	2.6	included
#14	ABB_JBB	C	–	F	1994/2/1	–	–	2012/6/14	18.4	included
#77	ABB_JBB	C	–	F	1995/2/1	–	–	2018/7/11	23.4	included
Natsu	ABB_JBB	C	–	M	2016/1/26	–	–	2023/11/22	7.8	included
Miharu	ABB_JBB	C	–	F	2020/2/8	–	–	2023/10/31	3.7	included
Hachi	ABB_HBB	C	–	M	1995/12/28	–	–	2023/3/11	27.2	included
Tomo	ABB_HBB	C	–	F	1998/1/30	–	–	2023/11/30	25.8	included
AF35	ABB_JBB	W	Tochigi	F	2009/2/1	2010/6/13	1.4	2017/5/31	8.3	included
AM85	ABB_JBB	W	Tochigi	M	2016/2/1	2017/7/6	1.4	2017/7/6	1.4	included
AM21	ABB_JBB	W	Tochigi	M	2003/2/1	2008/7/15	5.4	2017/7/13	14.4	included
AF18	ABB_JBB	W	Tochigi	F	2006/2/1	2008/5/26	2.3	2015/6/7	9.3	included
AM15	ABB_JBB	W	Tochigi	M	2004/2/1	2007/8/10	3.5	2016/5/24	12.3	included
AF07	ABB_JBB	W	Tochigi	F	2001/2/1	2006/10/19	5.7	2018/7/15	17.4	included
AF19	ABB_JBB	W	Tochigi	F	2007/2/1	2008/6/8	1.3	2018/6/15	11.4	included
AM36	ABB_JBB	W	Tochigi	M	2009/2/1	2010/7/4	1.4	2018/7/26	9.5	included
AF55	ABB_JBB	W	Tochigi	F	2010/2/1	2012/8/12	2.5	2019/6/17	9.4	included
AF62	ABB_JBB	W	Tochigi	F	2009/2/1	2012/8/12	3.5	2019/6/27	10.4	included
AM68	ABB_JBB	W	Tochigi	M	2010/2/1	2014/5/29	4.3	2020/8/17	10.5	included
AM87	ABB_JBB	W	Tochigi	M	2016/2/1	2017/7/20	1.5	2020/8/17	4.5	included
AF16	ABB_JBB	W	Tochigi	F	2006/2/1	2008/4/28	2.2	2021/6/26	15.4	included
AF45	ABB_JBB	W	Tochigi	F	2008/2/1	2010/11/12	2.8	2021/6/15	13.4	included
AM101	ABB_JBB	W	Tochigi	M	2019/2/1	2020/7/6	1.4	2021/7/8	2.4	included
AF81	ABB_JBB	W	Tochigi	F	2016/2/1	2017/6/14	1.4	2017/6/14	1.4	included
AF82	ABB_JBB	W	Tochigi	F	2016/2/1	2017/6/14	1.4	2017/6/14	1.4	included
AM100	ABB_JBB	W	Tochigi	M	2018/2/1	2019/7/22	1.5	2019/7/22	1.5	included
AF46	ABB_JBB	W	Tochigi	F	2006/2/1	2011/5/30	5.3	2021/6/12	15.4	included
AM58	ABB_JBB	W	Tochigi	M	2010/2/1	2012/6/17	2.4	2018/7/26	8.5	included
NG_101	ABB_JBB	W	Nagano	M	2012/2/1	2013/8/19	1.5	2020/6/3	8.3	included
NG18B58	ABB_JBB	W	Nagano	M	2016/2/1	2018/8/11	2.5	2020/8/5	4.5	included

NG18B38	ABB_JBB	W	Nagano	F	2017/2/1	2018/7/9	1.4	2020/8/16	3.5	included
NG_223	ABB_JBB	W	Nagano	F	2019/2/1	2019/11/23	0.8	2020/8/20	1.5	included
NG17B01	ABB_JBB	W	Nagano	M	2014/2/1	2017/5/22	3.3	2019/5/11	5.3	included
NG_54	ABB_JBB	W	Nagano	F	2004/2/1	2009/6/6	5.3	2018/5/25	14.3	included
NG_33	ABB_JBB	W	Nagano	M	2004/2/1	2006/6/18	2.4	2017/7/14	13.4	included
NG_64	ABB_JBB	W	Nagano	F	2005/2/1	2009/8/24	4.6	2017/7/14	12.4	included
NG_171	ABB_JBB	W	Nagano	M	2011/2/1	2014/6/4	3.3	2017/8/19	6.5	included
NG_76	ABB_JBB	W	Nagano	F	2008/2/1	2011/5/27	3.3	2017/8/29	9.6	included
NG_354	ABB_JBB	W	Nagano	F	2012/2/1	2015/10/14	3.7	2017/8/30	5.6	included
NG17B46	ABB_JBB	W	Nagano	M	2017/2/1	2017/8/31	0.6	2017/8/31	0.6	included
NG_158	ABB_JBB	W	Nagano	M	2016/2/1	2016/11/12	0.8	2017/9/3	1.6	included
NG_49	ABB_JBB	W	Nagano	F	2004/2/1	2008/10/28	4.7	2020/6/4	16.3	included
NG_107	ABB_JBB	W	Nagano	F	2013/2/1	2014/7/16	1.5	2020/7/3	7.4	included
NG_189	ABB_JBB	W	Nagano	F	2016/2/1	2019/6/3	3.3	2020/9/8	4.6	included
Hokuto	PB	C	—	M	2000/12/8	—	—	2022/11/7	21.9	included
Cookie	PB	C	—	F	1993/1/21	—	—	2023/2/21	30.1	excluded*
Rara	PB	C	—	F	1994/11/20	—	—	2022/12/27	28.1	included
Denali	PB	C	—	M	1993/11/9	—	—	2022/12/27	29.1	included
Light	PB	C	—	M	2013/11/21	—	—	2023/3/8	9.3	included
Icchan	PB	C	—	F	2013/12/11	—	—	2022/12/26	9.0	excluded*
Houchan	PB	C	—	F	2020/11/25	—	—	2022/12/1	2.0	excluded*
Peace	PB	C	—	F	1999/12/2	—	—	2023/4/9	23.4	included
Candy	PB	C	—	F	1992/11/2	—	—	2023/4/20	30.5	included
Cookie	PB	C	—	F	1993/1/21	—	—	2023/4/20	30.2	included
Ikoro	PB	C	—	M	2008/12/9	—	—	2023/5/22	14.4	included
Kai	PB	C	—	M	2004/12/2	—	—	2023/6/21	18.5	included
Pola	PB	C	—	F	2004/12/5	—	—	2023/6/21	18.5	included
Pola	PB	C	—	F	2004/12/5	—	—	2023/7/3	18.6	excluded*
Maruru	PB	C	—	F	2012/12/8	—	—	2023/4/28	10.4	excluded*
Maruru	PB	C	—	F	2012/12/8	—	—	2023/7/21	10.6	included
Lila	PB	C	—	F	2014/12/21	—	—	2023/10/28	8.9	excluded*
Yume	PB	C	—	F	2021/12/10	—	—	2023/12/1	2.0	included
Ruru	PB	C	—	F	1994/11/20	—	—	2022/6/12	27.6	included
Iwan	PB	C	—	M	2000/11/20	—	—	2019/9/28	18.9	included
Deah	PB	C	—	F	2008/12/2	—	—	2024/1/16	15.1	included
Momo	PB	C	—	F	2014/11/25	—	—	2016/6/13	1.5	included
Bufin	PB	C	—	F	1991/12/9	—	—	2016/6/13	24.5	included
Icchan	PB	C	—	F	2013/12/11	—	—	2017/7/7	3.6	included
Houchan	PB	C	—	F	2020/11/25	—	—	2023/10/21	2.9	included
Kiroru	PB	C	—	M	2008/12/9	—	—	2024/3/19	15.3	included
Lila	PB	C	—	F	2014/12/21	—	—	2024/3/19	9.2	included
Umekichi	SB	C	—	M	2009/10/11	—	—	2023/3/1	13.4	included
Masa	SB	C	—	F	2008/6/26	—	—	2023/1/30	14.6	included

Mars	SB	C	–	M	2003/4/29	–	–	2023/3/22	19.9	included
Shine	SB	C	–	M	2009/9/25	–	–	2023/3/11	13.5	excluded*
Taochii	SB	C	–	F	1994/7/2#	–	–	2019/12/2	25.4	included
Taochii	SB	C	–	F	1994/7/2#	–	–	2021/10/16	27.3	excluded*
Wanpii	SB	C	–	M	1994/7/2#	–	–	2023/4/5	28.8	excluded*
Wanpii	SB	C	–	M	1994/7/2#	–	–	2023/4/14	28.8	included
Ma-ne	SB	C	–	F	2015/8/27	–	–	2023/11/18	8.2	included
Happy	SB	C	–	F	2006/10/3	–	–	2023/12/8	17.2	included
Shine	SB	C	–	M	2009/9/25	–	–	2024/2/19	14.4	included
Daisuke	ADB	C	–	M	1990/12/4	–	–	2023/12/4	33.0	excluded

ABB_JBB: Asian black bears, Japanese black bears; ABB_HBB: Asian black bears, Himarayan black bears; PB: polar bears; SB: sun bears; ADB: Andean bears; C: captive; W: Wild; F: female; M: male
#The date of birth for two sun bears, born in 1994, were unavailable. It was assumed that both were born in the middle of the year (i.e., July 2).
*Some of the duplicated samples were excluded from the construction of the pan-bear age estimation model.

Supplementary Table S_R1. Correlation between DNA methylation level and chronological age. This table includes correlation coefficient (cor) and *p*-values for each CpG for each bear species.

Species	SL-1		SL-2		SL-3		SL-4	
	cor	*p*-value	cor	*p*-value	cor	*p*-value	cor	*p*-value
Asian black bears	0.943	< 0.0001	0.958	< 0.0001	0.943	< 0.0001	0.967	< 0.0001
Polar bears	0.973	< 0.0001	0.967	< 0.0001	0.967	< 0.0001	0.964	< 0.0001
Sun bears	0.941	< 0.0001	0.903	< 0.001	0.906	< 0.001	0.907	< 0.001

Supplementary Table S_R2. Coefficient values and p-values for the linear regression of Δage or |Δage| in the best Asian black bears specific age estimation model (the SVR model using SL-1, -2 and -4) .

	Estimate	*p*-value
Δage		
(Intercept)	0.0001824	−
|Δage|		
(Intercept)	0.153	−

Supplementary Table S_R3. Optimized parameters for each Asian black bears specific age estimation model.

model	CpG sites	alpha	lambda	log_{10}(cost)	log_{10}(gamma)	epsilon
Elastic net regression	SL-1, -2, -3, -4	0.01	0.0094842			
Support vector regression	SL-1, -2, -3, -4			5.0	-3.6	0.1
	SL-1, -2, -3			0.4	-0.8	0.1
	SL-1, -2, -4			4.9	-3.1	0.1
	SL-1, -3, -4			2.4	-2.2	0.1
	SL-2, -3, -4			2.1	-1.8	0.1
	SL-1, -2			1.7	-2.6	0.1
	SL-1, -3			0.4	-0.6	0.1
	SL-1, -4			1.5	-2.2	0.1
	SL-2, -3			4.8	-2.4	0.1
	SL-2, -4			4.5	-1.9	0.1
	SL-3, -4			1.9	-1.5	0.1

Supplementary Table S_R4. Optimized parameters for each pan-bear age estimation model.

model	CpG sites	alpha	lambda	log10(cost)	log10(gamma)	epsilon
Elastic net regression	SL-1, -2, -3, -4	0.02	0.0084241			
Support vector regression	SL-1, -2, -3, -4			0.2	-1.2	0.1
	SL-1, -2, -3			0.1	-1.0	0.1
	SL-1, -2, -4			0.2	-1.5	0.1
	SL-1, -3, -4			3.7	-1.9	0.1
	SL-2, -3, -4			0.4	-1.0	0.1
	SL-1, -2			1.1	-1.1	0.1
	SL-1, -3			0.3	-0.9	0.1
	SL-1, -4			3.9	-1.9	0.1
	SL-2, -3			3.2	-1.6	0.1
	SL-2, -4			1.0	-0.9	0.1
	SL-3, -4			4.0	-1.8	0.1

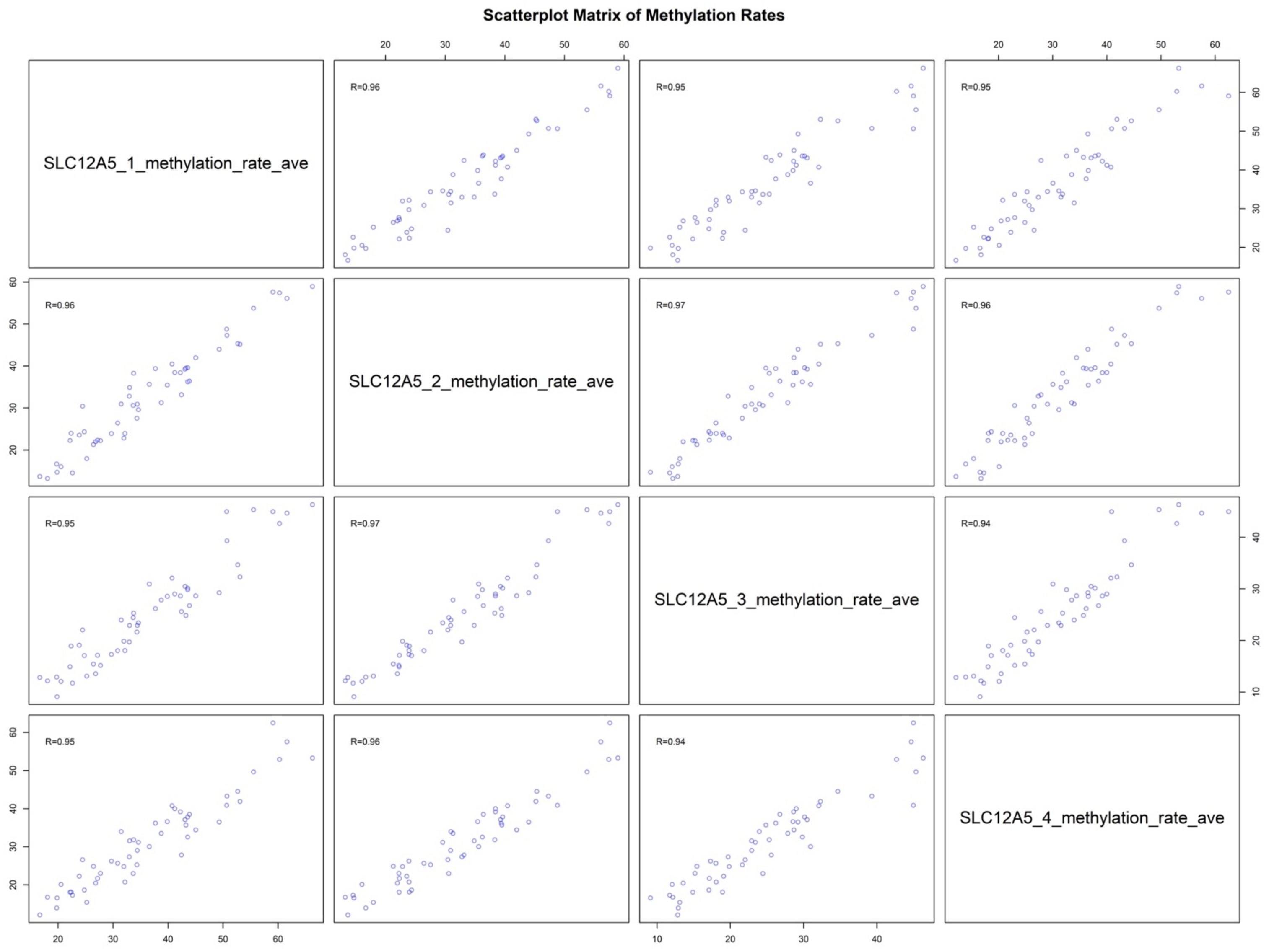

Supplementary Figure S_M1. Pairwise scatter plots of methylation levels (%) among the four CpGs adjacent to *SLC12A5* for Asian black bears.

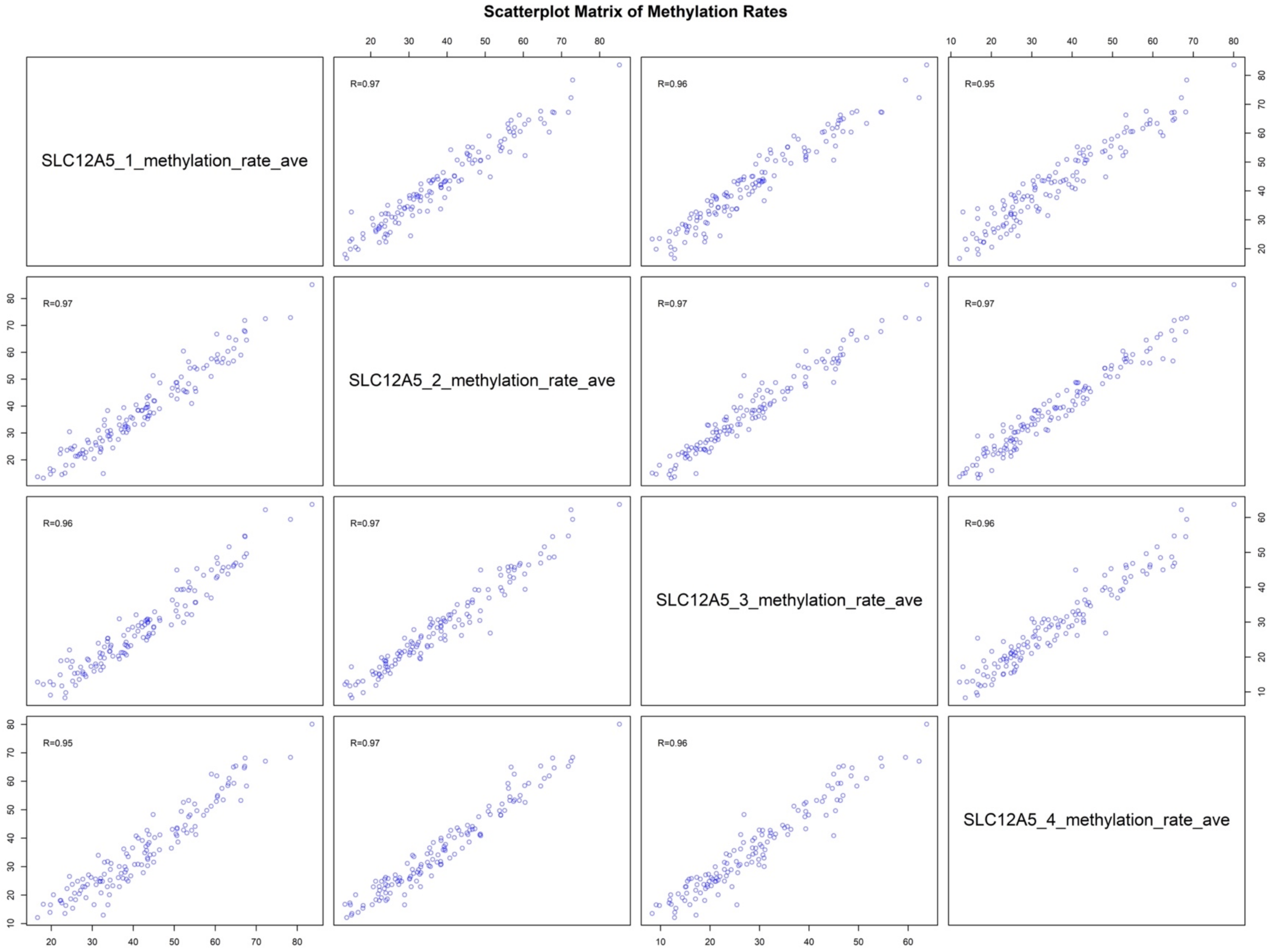

Supplementary Figure S_M2. Pairwise scatter plots of methylation levels (%) among the four CpGs adjacent to *SLC12A5* for bears included in the pan-bear model (i.e., brown bears, Asian black bears, polar bears, and sun bears).

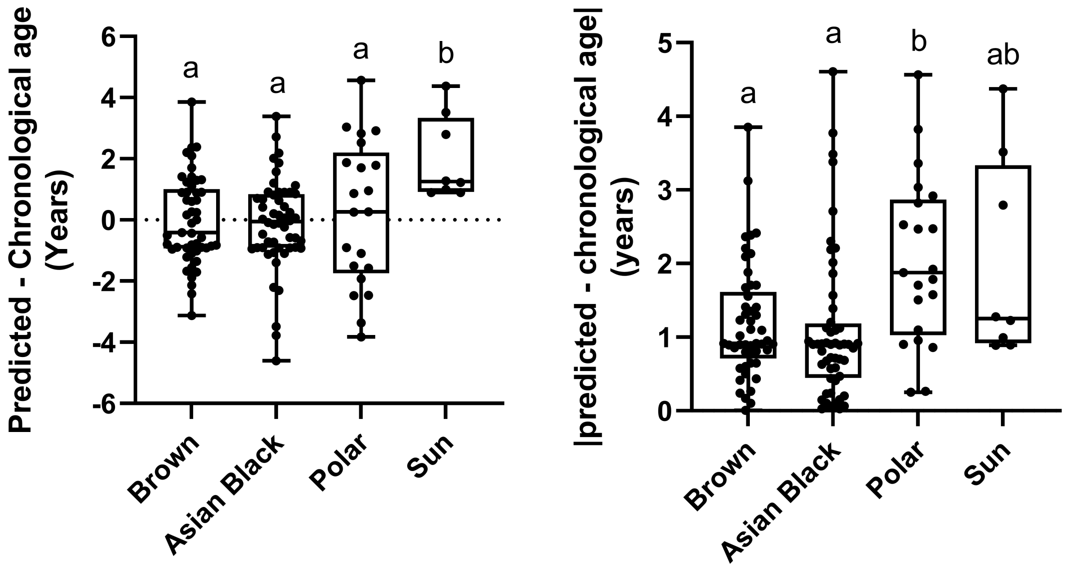

Supplementary Figure S_R1. Species differences in Δage (left) and |Δage| values (right) in the pan-bear age estimation model. Different letters indicate significant differences between bear species (Tukey's multiple comparison test, $P < 0.05$).
